# Divergent Reactivity of an Isolable Nickelacyclobutane

**DOI:** 10.1002/anie.202111389

**Published:** 2021-11-08

**Authors:** María L. G. Sansores‐Paredes, Storm van der Voort, Martin Lutz, Marc‐Etienne Moret

**Affiliations:** ^1^ Organic Chemistry and Catalysis Debye Institute for Nanomaterials Science Faculty of Science Utrecht University Universiteitsweg 99 3584 CG Utrecht The Netherlands; ^2^ Structural Biochemistry Bijvoet Centre for Biomolecular Research Faculty of Science Utrecht University Universiteitsweg 99 3584 CG Utrecht The Netherlands

**Keywords:** cyclopropanation, metallacycles, metathesis, nickel, pincer compounds

## Abstract

Nickelacyclobutanes are mostly invoked as reactive intermediates in the reaction of nickel carbenes and olefins to yield cyclopropanes. Nevertheless, early work suggested that other decomposition routes such as β‐hydride elimination and even metathesis could be accessible. Herein, we report the isolation and characterization of a stable pentacoordinated nickelacyclobutane incorporated in a pincer complex. The coordination of different coligands to the nickelacyclobutane determines its selective decomposition along cyclopropanation, metathesis or apparent β‐hydride elimination pathways. DFT calculations shed light on the mechanism of these different pathways.

## Graphical abstract

A pincer scaffold allows the isolation of a nickelacyclobutane and a detailed study of its reactivity. The cycle slowly undergoes an apparent beta‐elimination reaction in non‐polar solvents; exposure to additional ligands leads to selective ring cleavage along either the cyclopropanation or the olefin metathesis pathway. These results give insight into the divergent reactivity of metallacyclobutane intermediates.

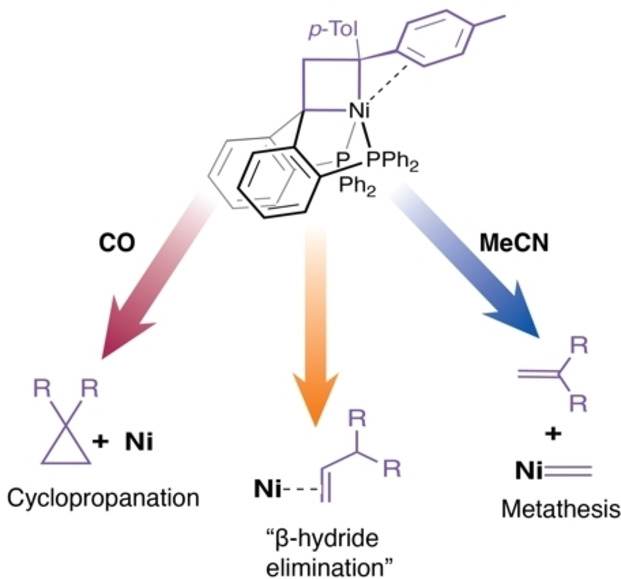

Metallacyclobutanes are key reactive intermediates in catalytic cycles such as olefin metathesis and cyclopropanation.[^1^, ^2^, ^3^, ^4^, ^5^, ^6^, ^7^, ^8^, ^9^, ^10^] They can undergo several distinct decomposition reactions including i) reductive elimination to yield cyclopropanes, ii) [2+2] cycloreversion to form an alkene and a metal carbene and iii) β‐hydride elimination followed by reductive elimination to form an alkene complex (Scheme 1).[^3^, ^11^] While many highly selective catalytic cyclopropanation or metathesis reactions are known, our understanding of the factors that determine the selectivity of metallacyclobutane decomposition remains limited.^[12]^

**Scheme 1:**
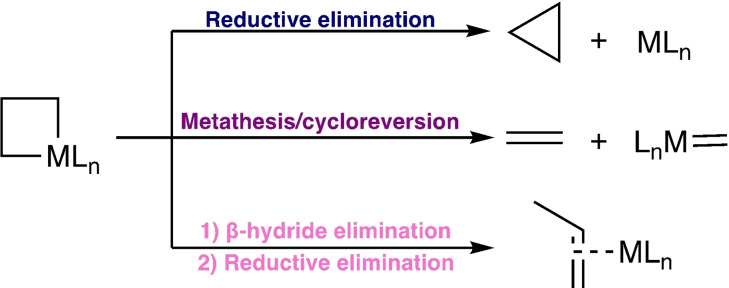
Metallacyclobutane decomposition.

More specifically, nickelacyclobutanes formed by [2+2] cycloaddition of a nickel carbene intermediate and an olefin are commonly proposed intermediates in catalytic cyclopropanation.[^6^, ^7^, ^8^, ^9^, ^10^, ^11^, ^13^, ^14^, ^15^, ^16^, ^17^] In particular, mechanistic studies using nitrogen ylides as the carbene source have shown that olefin coordination prior to carbene formation enhances nickelacyclobutane formation over unproductive decomposition of the nickel carbene intermediate.[^9^, ^10^] Direct observations of such [2+2] cycloadditions are limited to reactions of nickel fluorocarbenes with fluorinated olefins. Interestingly, these reactions also yield olefin metathesis products, but the isolated perfluorinated nickelacyclobutane is not an intermediate in this process.[^18^, ^19^, ^20^] Further indication for the feasibility of Ni‐based olefin metathesis comes from early work by Miyashita and Grubbs, in which they identified carbene‐derived products from the thermal decomposition of phosphine‐supported nickelacyclobutanes.[^11^, ^13^, ^14^] The preferred decomposition pathway strongly depended on the coordination environment of nickel: conditions favoring mono‐, di‐ and triligated species afforded different product distributions, the latter favoring C−C bond cleavage reactions akin to metathesis.[^1^, ^11^, ^13^, ^14^, ^21^, ^22^] These observations suggest that the reactivity of these species could be controlled by ligand design.

Herein we show that a pincer ligand framework incorporating a precoordinated olefin allows trapping of a transient nickel carbene to form a stable nickelacyclobutane. This architecture favors pentacoordinate geometries, which are unusual amongst predominantly square planar group 10 metallacycles.[^3^, ^23^, ^24^, ^25^, ^26^, ^27^] Depending on the presence and nature of an additional ligand, the nickelacyclobutane selectively undergoes cyclopropanation, [2+2] cycloreversion, or apparent β‐hydride elimination. Experiments and computations provide insight into the mechanistic pathways.

The ligand 1,1‐bis[2‐(diphenylphosphino)phenyl]ethene (^Ph^bppe^H,H^, **1**) was accessed from the corresponding ketone^[28]^ via Wittig reaction. Complexation with Ni(cod)_2_ and *p*‐fluorostyrene afforded the tetrahedral Ni^0^ complex **2** (Scheme 2) as confirmed by an X‐ray crystal structure and multinuclear NMR (see SI, section 4).

**Scheme 2:**
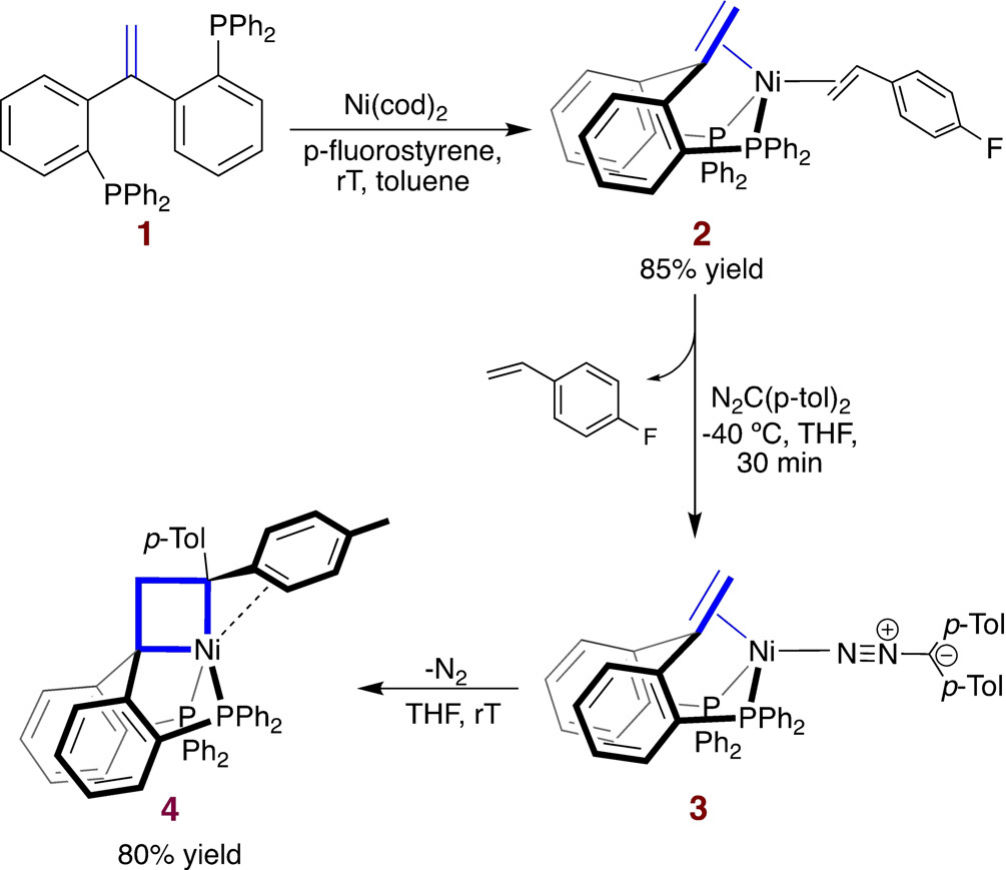
^Ph^bppe^H,H^ complexation and nickelacyclobutane formation.

Exposing complex **2** to bis(*p*‐tolyl)diazomethane led to release of *p*‐fluorostyrene and formation of adduct **3**, which readily releases N_2_ at room temperature (also in the dark) to form nickelacyclobutane **4** and could not be isolated in bulk (Scheme 2, see SI section 2.1). The IR spectrum of a mixture of **3** and **4** showed a characteristic ν(N=C) absorption band at 2044 cm^−1^. Gratifyingly, crystals of **3** suitable for X‐ray diffraction could also be obtained from such a mixture. The resulting crystal structure confirms the η^1^(N) binding mode of the diazo ligand and the tridentate P‐η^2^(C,C)‐P pincer coordination (see SI, section 4). The C−N distance of 1.312(3) Å indicates a relatively weak activation of the diazoalkane in comparison with previously known examples (C−N: 1.327(2)–1.349(4) Å).[^17^, ^29^, ^30^]

An X‐ray crystal structure of nickelacyclobutane **4** (Figure 1) reveals a nickel(II) center that can be described as pentacoordinated with four ligands from the pincer framework (P1, P2, C7, C39) and an additional π‐interaction with one of the tolyl groups (C40, C41). A transannular Ni−C38 distance of 2.577(2) Å and an angle between the planes C7‐C38‐C39 and C7‐Ni1‐C39 of 27.69(19)° shows a high degree of puckering in the four‐membered ring.^[30]^

**Figure 1:**
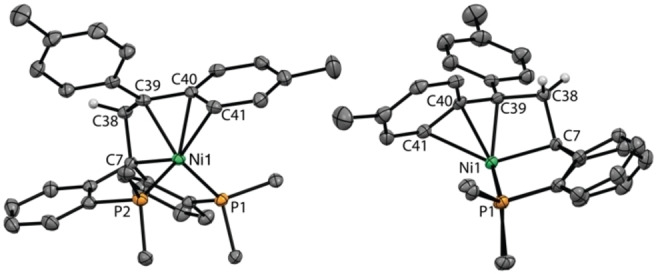
Two views of the molecular structure of **4**. Displacement ellipsoids are drawn at the 50 % probability level. Solvent molecules, most H atoms and phenyl rings from the phosphines are omitted for clarity.^[30]^ Selected bond lengths (Å) and angles (deg): Ni1–C7 2.011(2), C7–C38 1.541(3), C38–C39 1.524(3), C39–C40 1.465(3), C40–C41 1.428(3), Ni1–C39 2.058(2), Ni1–C40 2.049(2), Ni1–C41 2.240(2), Ni–C38 2.577(2), C7‐Ni1‐C39 70.41(8), C7‐C38‐C39 99.88(16), Ni1‐C7‐C38 92.08(12).

NMR analysis of **4** in [D_8_]toluene solution at −40 °C is consistent with the solid‐state structure. The methylene group affords diastereotopic ^1^H signals at *δ*=4.35 and 4.40 ppm. One aromatic ^1^H signal is shifted upfield at 5.35 ppm and the two CH_3_ groups are diasterotopic, which we attribute to π‐coordination of one of the *p*‐tolyl group. Accordingly, the ^31^P NMR spectrum at −40 °C presents two doublets at 21.9 and 44.9 ppm (*J*_P,P_=77 Hz). Upon warming to 25 °C, the ^31^P NMR signals coalesce to one broad signal in the 20–40 ppm range while the methylene and C*H*_3_^1^H signals coalesce to 4.40 ppm and 1.76 ppm, respectively. These observations are consistent with exchange of the bound and unbound *p*‐tolyl groups on the NMR timescale, for which a Gibbs free energy of activation of 12.0±0.2 kcal mol^−1^ at −20 °C can be estimated (see SI, section 2.2).

Upon exposure of compound **4** to CO (1 atm) as a π‐acceptor coligand, a new complex was formed that displays symmetrical ^1^H and ^31^P NMR spectra and a strong IR absorption at ν(C=O)=1984 cm^−1^ (see SI, section 2.3). The spectroscopic data are consistent with the trigonal bipyramidal (TBP) nickelacyclobutane structure **4‐CO** (Scheme 3), in which a CO ligand has displaced the π‐interaction and occupies an axial position. **4‐CO** is unstable in solution and fully converts over 16 h under CO to compound **5**, which contains two CO ligands as shown by IR absorptions at 1944 and 2002 cm^−1^. An X‐ray crystal structure (Figure 2) identified complex **5** as a tetrahedral nickel(0) complex of a diphosphine ligand containing a cyclopropane unit that results from reductive elimination of the metallacycle.^[30]^ The orientation of the cyclopropane ring in **5** is not that expected from direct reductive elimination: the CH_2_ group instead of the Ctol_2_ group points towards the nickel center, which likely reduces steric repulsion. In the absence of any indication of ring rearrangements in the nickelacyclobutane,[^31^, ^32^, ^33^] we propose transient phosphine decoordination after cyclopropane formation as the most likely isomerization pathway, but inversion of the chelate cycle cannot be excluded.

**Figure 2:**
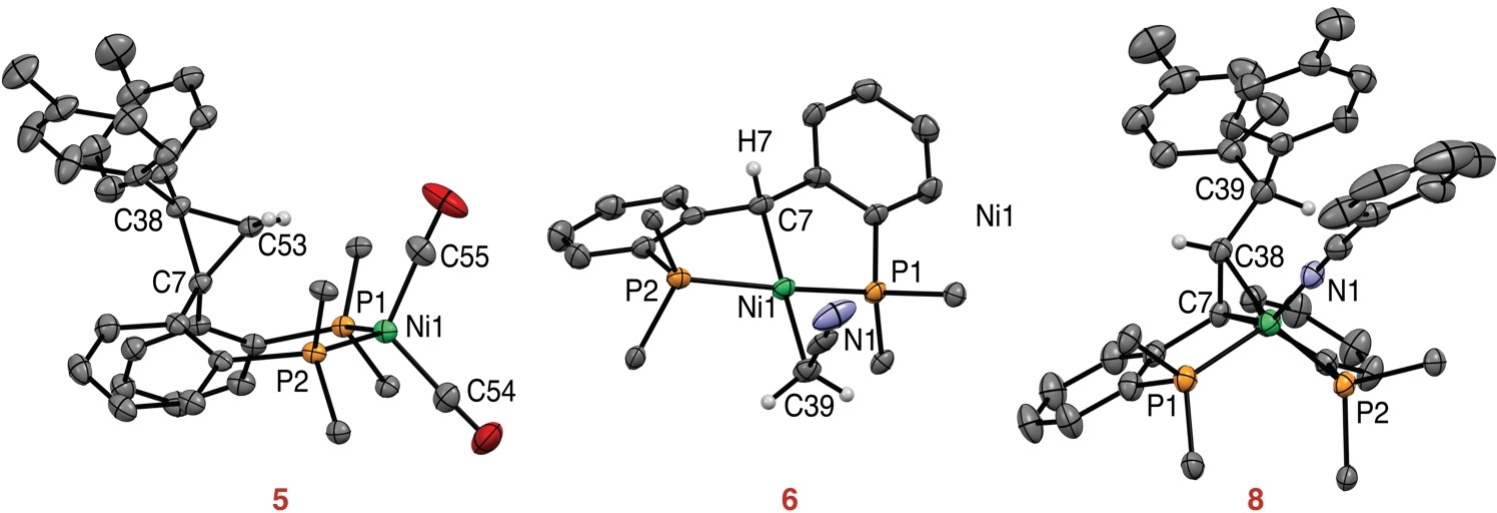
Molecular structures of complex **5**, **6** and **8**. Displacement ellipsoids are drawn at the 50 % probability level. Solvent molecules, most H atoms and phenyl rings from the phosphines are omitted for clarity.^[30]^ Selected bond lengths (Å) and angles (deg), for complex **5**: Ni1–C55 1.7728(14), Ni1–C54 1.7852(14), Ni1–P1 2.2203(3), Ni1–P2 2.2298(3). For complex **6**: Ni1–C7 2.015(5), Ni1–C39 1.997(5), Ni1–P1 2.1356(14), Ni1–P2 2.1859(14). For complex **8**: Ni1–P1 2.1845(9), Ni1–P2 2.1718(9), Ni1–N1 1.878(3), Ni1–C7 2.027(3), Ni1–C38 2.039(3).

**Scheme 3:**
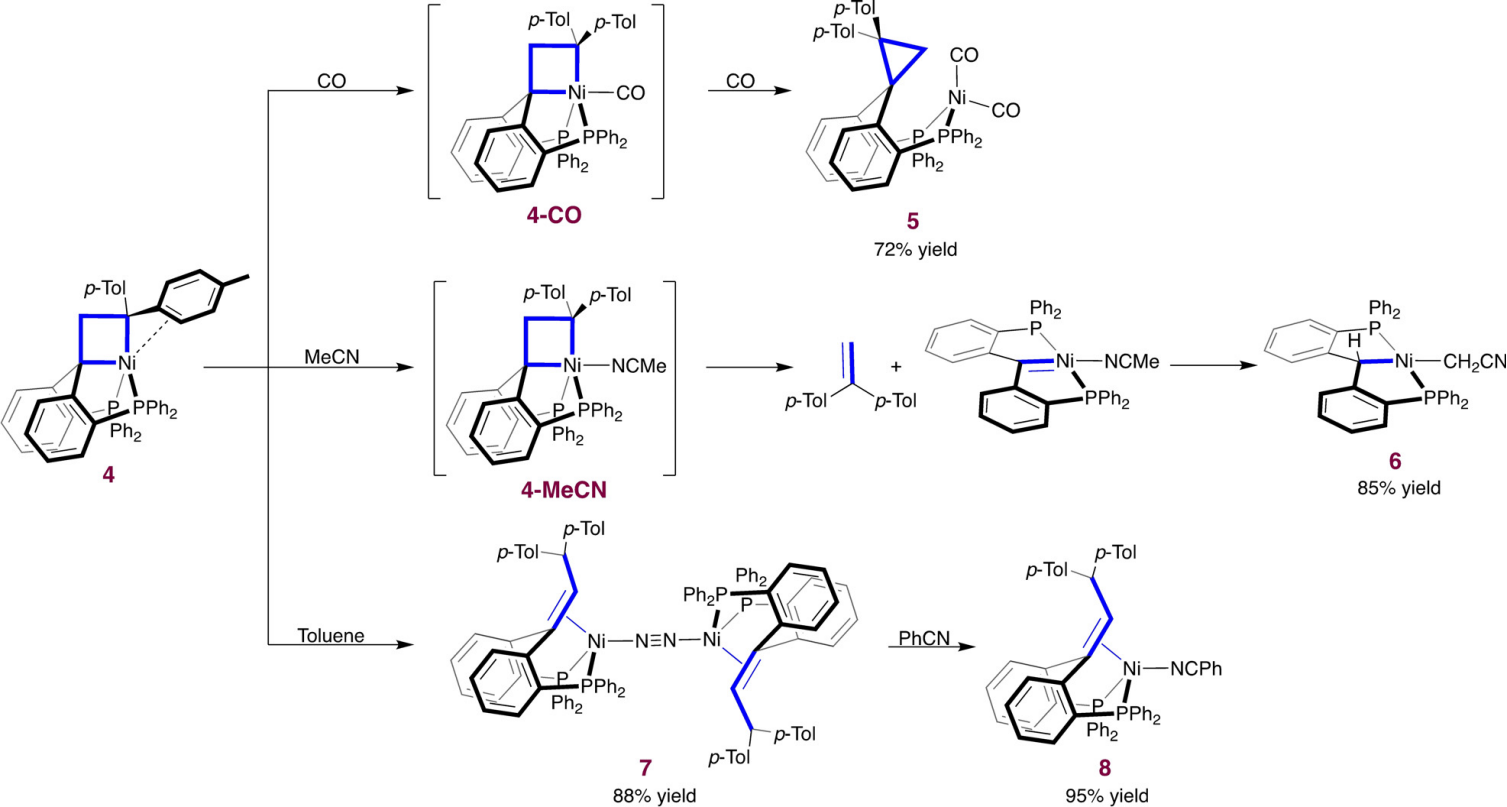
Reactivity of nickelacyclobutane **4**.

To study the influence of a σ‐donor coligand, the reactivity of nickelacyclobutane **4** with MeCN was explored (Scheme 3). In CD_3_CN/C_6_D_6_, ^1^H NMR and ^31^P NMR indicate a symmetrical species at −30 °C (see SI, section 2.4), consistent with the formation of TBP complex **4‐MeCN** featuring an MeCN ligand in axial position. Over 1 h at room temperature in MeCN, **4‐MeCN** decayed to a symmetrical complex devoid of tolyl fragments (**6**) with release of 1,1‐di(*p*‐tolyl)ethylene. Complex **6** was crystallographically and spectroscopically identified as a square planar nickel(II) complex bearing a P(CH)P pincer ligand and a CH_2_CN group (Figure 2).^[30]^ Repeating the experiment with a CD_2_ group instead of CH_2_ in nickelacyclobutane **4** yielded d^2^‐1,1‐di(p‐tolyl)ethylene, confirming the origin of the methylene group. The formation of these products can be explained by [2+2] cycloreversion of the nickelacyclobutane to yield the alkene and the carbene pincer complex (PC_carbene_P)Ni(MeCN), which subsequently activates a C−H bond from MeCN. Related nickel and palladium carbene pincer compounds are known, with similar MeCN activation being reported for palladium.[^34^, ^35^, ^36^, ^37^] To our knowledge, this is the first observation of a nickelacyclobutane selectively undergoing a metathesis‐like ring opening.

In non‐polar solvents benzene and toluene, nickelacyclobutane **4** slowly decomposed to a new compound (**7**); full conversion requires more than 24 h at room temperature and can be accelerated by heating. An X‐ray crystal structure revealed **7** as a dinuclear nickel(0) complex featuring a bridging dinitrogen molecule (Scheme 3, SI section 4).^[30]^ The ligand backbone contains a new olefin formally resulting from the insertion of a di‐*p*‐tolylcarbene fragment into one of the olefinic C−H bonds of ligand **1**. Reaction of **7** with benzonitrile yielded the monomeric complex **8** (Figure 2). While complex **7** could be seen as the result of β‐hydride elimination from nickelacyclobutane **4**,[^11^, ^13^, ^14^, ^21^, ^22^] the syn coplanar geometry required for such a concerted reaction would likely be strained. This intramolecular pathway was indeed ruled out by a crossover experiment with equal amounts of nickelacyclobutane and d^2^‐nickelacyclobutane in toluene. After reaction, the two C‐H positions originating from the ligand CH_2_/CD_2_ group exhibited 23 % and 77 % deuterium incorporation, respectively, whereas twice 50 % would be expected for an intramolecular process. Therefore, a more intricate intermolecular mechanism is at play (see SI sections 2.5 and 5.2.2).

To gain insight into the mechanism and selectivity of nickelacyclobutane transformations, DFT calculations were performed (Figure 3). First, a nickel carbene pathway accounts for nickelacyclobutane formation from the η^1^(N) diazoalkane adduct **3**. Endergonic (15.4 kcal mol^−1^) isomerization to the η^1^(C) binding mode (**9**) is followed by facile nitrogen release (**TS1**) to form nickel carbene **10** with an overall barrier of 22.6 kcal mol^−1^, in accord with N_2_ extrusion being accessible at room‐temperature. This is somewhat surprising given that this process required UV light irradiation or Lewis acid additives for related Ni carbenes,[^6^, ^38^] which may suggest a cooperative role of the alkene ligand. The double bond can further decoordinate via a low‐lying **TS2** (1.7 kcal mol^−1^) to yield three‐coordinated nickel carbene **11**, which is more stable by 3 kcal mol^−1^. From structure **10**, **TS3** (18.3 kcal mol^−1^) yields nickelacyclobutane **4** via the Chauvin mechanism^[39]^ with an overall free energy gain of 30.3 kcal mol^−1^ from diazoadduct **3**. For comparison, a non‐carbene pathway for nickelacyclobutane formation via nucleophilic attack of the diazoalkane on the alkene backbone^[17]^ was predicted to have a total energy barrier of 54.6 kcal mol^−1^ with respect to diazoadduct **3** and is therefore implausible (see SI section 5.2.1).

**Figure 3:**
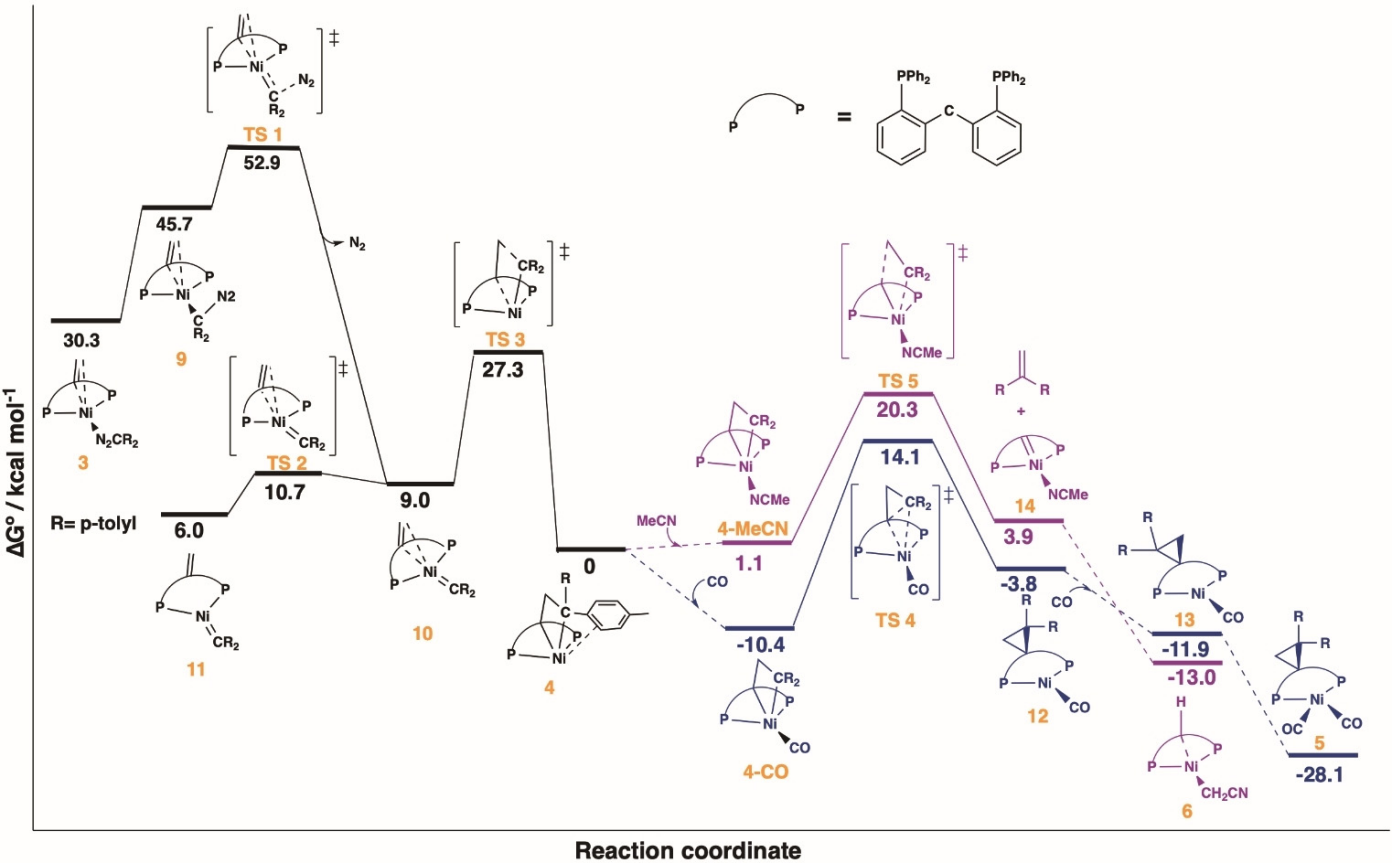
Gibbs free energy profiles for the formation of nickelacyclobutane 4 and further reactivity with CO and MeCN computed at B3LYP‐GD3BJ/def2TZVP//B3LYP‐GD3BJ/6‐31(d,p) level of theory. In blue: cyclopropanation pathway, in purple: metathesis pathway. Dashed lines connect intermediates between which no transition state was optimized.

The cyclopropanation pathway (blue) starts with the coordination of CO yielding TBP complex **4‐CO** that undergoes a concerted reductive elimination (**TS4**) with a barrier of 24.5 kcal mol^−1^. The resulting tricoordinate nickel complex **12** shows no nickel‐cyclopropane interaction and accepts another CO ligand to form tetracoordinated complex **13** with an exergonicity of 8.1 kcal mol^−1^. Isomerization to the less sterically encumbered cyclopropane complex **5** is favored by 16.2 kcal mol^−1^. For comparison, the metathesis pathway from **4‐CO** was computed. The corresponding TS is associated with a similar energy barrier of 24.4 kcal mol^−1^ and leads to a more energetic (PC_carbene_P)Ni(CO) product in comparison with the cyclopropane complex (**5**) by 19.8 kcal mol^−1^. Hence, cyclopropanation is predicted to be thermodynamically favored in the presence of an excess of CO, but metathesis might become competitive with limited CO reactant (see SI, sections 2.2 and 5.2.4).

For the metathesis pathway in MeCN (purple), the formation of TBP nickelacyclobutane compound **4‐MeCN** is nearly ergoneutral (+1.1 kcal mol^−1^), consistent with coordination occurring in the presence of a large excess of MeCN. The subsequent transition state for cycloreversion (**TS5**) yields a (PC_carbene_P)Ni(MeCN) complex (**14**) and 1,1‐di(*p*‐tolyl)ethylene with a barrier of 19.9 kcal mol^−1^. Afterwards, C‐H activation to produce complex **6** provides an overall driving force of −13 kcal mol^−1^ for this pathway. For comparison, the transition state for cyclopropane formation from **4‐MeCN** is significantly higher in energy (28.5 kcal mol^−1^) than for cycloreversion (20.3 kcal mol^−1^), disfavoring this pathway in accord with experimental observations (see SI, section 5.2.3).

Additionally, cyclopropanation and cycloreversion routes from nickelacyclobutane **4** were computed in the absence of exogenous ligands. Cyclopropanation is endergonic by 25.2 kcal mol^−1^ and associated with a barrier of 30.5 kcal mol^−1^, and therefore inaccessible at room temperature (see SI, section 5.2.3). In contrast, calculations associated cycloreversion from nickelacyclobutane (**4**) with a barrier of 24.4 kcal mol^−1^, but the product (PC_carbene_P)Ni(1,1‐di(p‐tolyl)ethylene) is thermodynamically disfavored by 13.5 kcal mol^−1^.

In summary, we presented the synthesis and characterization of a nickelacyclobutane via a nickel carbene pathway. The cycle is stabilized by a diphosphine pincer ligand that favors an unusual pentacoordinated geometry. Selective decomposition to a cyclopropane derivative can be induced by introducing a π‐acceptor coligand such as CO in axial position, whereas selective [2+2] cycloreversion/metathesis is observed with a σ‐donor coligand such as MeCN. Additionally, an olefin product that would be consistent with a β‐hydride elimination/reductive elimination sequence from the nickelacyclobutane in the absence of coligands was shown to instead originate from an intermolecular pathway. The accessibility of these different pathways owes to the ability of the pincer scaffold to accommodate both Ni^0^ and Ni^II^ species and to sample different coordination modes. Our findings underline the influence of the coordination environment of nickelacyclobutane intermediates on their selective reactivity and suggests that controlled Ni‐catalyzed olefin metathesis may be accessible. This possibility is currently under investigation in our laboratory.

## Conflict of interest

The authors declare no conflict of interest.

## Supporting information

As a service to our authors and readers, this journal provides supporting information supplied by the authors. Such materials are peer reviewed and may be re‐organized for online delivery, but are not copy‐edited or typeset. Technical support issues arising from supporting information (other than missing files) should be addressed to the authors.

Supporting Information

## References

[1] R. H. Grubbs , A. Miyashita , in Fundam. Res. Homog. Catal., Springer, Boston, MA, 1978, pp. 207–220.

[2] P. W. Jennings , L. L. Johnson , Chem. Rev. 1994, 94, 2241–2290.

[3] J. Cámpora , P. Palma , E. Carmona , Coord. Chem. Rev. 1999, 193–195, 207–281.

[4] O. M. Ogba , N. C. Warner , D. J. O'Leary , R. H. Grubbs , Chem. Soc. Rev. 2018, 47, 4510–4544. 29714397 10.1039/c8cs00027a PMC6107346

[5] T. P. Montgomery , A. M. Johns , R. H. Grubbs , Catalysts 2017, 7, 87.

[6] D. J. Mindiola , G. L. Hillhouse , J. Am. Chem. Soc. 2002, 124, 9976–9977. 12188647 10.1021/ja0269183

[7] R. Waterman , G. L. Hillhouse , J. Am. Chem. Soc. 2003, 125, 13350–13351. 14583018 10.1021/ja0381914

[8] K. D. Kitiachvili , D. J. Mindiola , G. L. Hillhouse , J. Am. Chem. Soc. 2004, 126, 10554–10555. 15327309 10.1021/ja047052z

[9] S. A. Künzi , J. M. Sarria Toro , T. Den Hartog , P. Chen , Angew. Chem. Int. Ed. 2015, 54, 10670–10674; 10.1002/anie.201505482 26223478

[10] S. A. Künzi , R. Gershoni-Poranne , P. Chen , Organometallics 2019, 38, 1928–1938.

[11] R. H. Grubbs , A. Miyashita , J. Am. Chem. Soc. 1978, 100, 7418–7420.

[12] C. P. Gordon , K. Yamamoto , W. C. Liao , F. Allouche , R. A. Andersen , C. Copéret , C. Raynaud , O. Eisenstein , ACS Cent. Sci. 2017, 3, 759–768. 28776018 10.1021/acscentsci.7b00174 PMC5532720

[13] A. Miyashita , R. H. Grubbs , Tetrahedron Lett. 1981, 22, 1255–1256.

[14] A. Miyashita , M. Ohyoshi , H. Shitara , H. Nohira , J. Organomet. Chem. 1980, 338, 103–111.

[15] E. S. Kline , R. H. Hauge , Z. H. Kafafi , J. L. Margrave , Organometallics 1988, 7, 1512–1516.

[16] D. B. Jacobson , B. S. Freiser , Organometallics 1984, 3, 513–519.

[17] N. D. Harrold , A. R. Corcos , G. L. Hillhouse , J. Organomet. Chem. 2016, 813, 46–54.

[18] D. J. Harrison , A. L. Daniels , I. Korobkov , R. T. Baker , Organometallics 2015, 34, 5683–5686.

[19] D. J. Harrison , A. L. Daniels , J. Guan , B. M. Gabidullin , M. B. Hall , R. T. Baker , Angew. Chem. Int. Ed. 2018, 57, 5772–5776; 10.1002/anie.201802090 29575521

[20] A. Rochon , M. R. Elsby , R. T. Baker , Can. J. Chem. 2021, 99, 209–215.

[21] R. H. Grubbs , A. Miyashita , M.-I. M. Liu , P. L. Burk , J. Am. Chem. Soc. 1977, 99, 3863–3864.

[22] R. H. Grubbs , A. Miyashita , M. Liu , P. Burk , J. Am. Chem. Soc. 1978, 100, 2418–2425.

[23] R. P. Hughes , R. T. Carl , D. E. Samkoff , R. E. Davis , K. D. Holland , Organometallics 1986, 5, 1053–1055.

[24] H. M. R. Hoffmann , A. R. Otte , A. Wilde , S. Menzer , D. J. Williams , Angew. Chem. Int. Ed. Engl. 1995, 34, 100–102;

[25] R. J. Klingler , J. C. Huffman , J. K. Kochi , J. Am. Chem. Soc. 1982, 104, 2147–2157.

[26] D. J. Yarrow , J. A. Ibers , M. Lenarda , M. Graziani , J. Organomet. Chem. 1974, 70, 133–145.

[27] J. A. Ibers , R. DiCosimo , G. M. Whitesides , Organometallics 1982, 1, 13–20.

[28] B. W. H. Saes , D. G. A. Verhoeven , M. Lutz , R. J. M. Klein Gebbink , M. E. Moret , Organometallics 2015, 34, 2710–2713.

[29] D. E. Herbert , N. C. Lara , T. Agapie , Chem. Eur. J. 2013, 19, 16453–16460. 24127196 10.1002/chem.201302539 PMC3897266

[30] Deposition Numbers 2100133, 2100134, 2100135, 2100136, 2100137, 2100138 and 2100139 contain the supplementary crystallographic data for this paper. These data are provided free of charge by the joint Cambridge Crystallographic Data Centre and Fachinformationszentrum Karlsruhe Access Structures service.

[31] C. N. Wilker , R. Hoffmann , J. Am. Chem. Soc. 1983, 105, 5285–5290.

[32] R. J. Al-Essa , R. J. Puddephatt , M. A. Quyser , C. F. H. Tipper , J. Am. Chem. Soc. 1979, 101, 364–370.

[33] R. J. Puddephatt , Can. J. Chem. 2021, 99, 165–172.

[34] C. C. Comanescu , V. M. Iluc , Organometallics 2014, 33, 6059–6064.

[35] D. V. Gutsulyak , W. E. Piers , J. Borau-Garcia , M. Parvez , J. Am. Chem. Soc. 2013, 135, 11776–11779. 23906261 10.1021/ja406742n

[36] C. C. Comanescu , V. M. Iluc , Polyhedron 2018, 143, 176–183.

[37] C. C. Comanescu , V. M. Iluc , Organometallics 2015, 34, 4684–4692.

[38] V. M. Iluc , G. L. Hillhouse , J. Am. Chem. Soc. 2014, 136, 6479–6488. 24716462 10.1021/ja501900j

[39] D. Astruc , New J. Chem. 2005, 29, 42–56.

