# Supplementary material for: Divergent Reactivity of an Isolable Nickelacyclobutane
Source: Angew Chem Int Ed Engl. 2021 Nov 8;60(51):26518–22. doi: 10.1002/anie.202111389 (PMC9298726; doi:10.1002/anie.202111389)
Supplement: Supplementary file 1 — Supporting Information [file ANIE-60-26518-s001.pdf]

Angewandte Chemie
Eine Zeitschrift der Gesellschaft Deutscher Chemiker

Supporting Information

Divergent Reactivity of an Isolable Nickelacyclobutane

*María L. G. Sansores-Paredes, Storm van der Voort, Martin Lutz, and Marc-Etienne Moret**

anie_202111389_sm_miscellaneous_information.pdf

Table of contents

1. Experimental section ... 1

1.1 General information ... 1

1.2 Physical methods ... 1

1.3 Synthesis and characterization ... 1

2. Additional experiments ... 8

2.1 Conversion of diazo adduct 3 to Nickelacyclobutane 4 ... 8

2.2 VT-NMR of Nickelacyclobutane 4 ... 9

2.3 IR analysis of intermediate 4-CO and reactivity under an N_2 atmosphere ... 12

2.4 VT NMR of Nickelacyclobutane (4) in presence of d^3-MeCN. Analysis of intermediate 4-MeCN. ... 14

2.5 Bimolecular experiment in the formation of $[(^{Ph}bppe^{H,CHptol2})Ni]_2N_2$ (7) ... 24

3. Spectra of new compound ... 28

4. X-ray crystal structure determinations ... 62

5. DFT studies ... 67

5.1 General information ... 67

5.2 Additional comments per pathway ... 67

5.2.1) Nickelacyclobutane formation ... 67

5.2.2) β-elimination ... 69

5.2.3) Cyclopropanation ... 69

5.2.4) Cycloreversion ... 71

5.2.5) Additional comments ... 72

5.3 Cartesian coordinates ... 73

6. Literature references ... 137

7. Author Contributions ... 137

1. Experimental section

1.1 General information

All reactants were purchased from commercial sources and used as received without further purification. Additionally, $Ni(cod)_2$, bis(4-methylphenyl)diazomethane and benzonitrile were stored in the glovebox. Benzonitrile was degassed by freeze pump procedure before used.
All the reactions were performed under an N_2(g) atmosphere using standard Schlenk line or glovebox techniques. Deuterated solvents were purchased from Cambridge Isotope Laboratory Incorporation (Cambridge, USA), degassed by freeze pump procedure, and stored over molecular sieves before use. Common solvents were dried using a MBRAUN MB SPS-80 purification system and/or distillation technique. Bis(4-methylphenyl)diazomethane[1,2] and 2,2'-bis(diphenylphosphino)benzophenone[3] were synthetized according to literature procedures.

1.2 Physical methods

^{1}H, ^{13}C, ^{19}F and ^{31}P NMR spectra (400, 100, 376 and 161 MHz respectively) were recorded on an Agilent MR400 or a Varian AS400 spectrometer at 297 K unless it is stated differently. ^{1}H and ^{13}C NMR chemical shifts relative to tetramethylsilane are referenced to the residual solvent resonance. ^{19}F NMR chemical shifts were referenced to $CFCl_3$ and ^{31}P NMR chemical shifts were referenced to 85% aqueous H_3PO_4 solution; both externally. Infrared spectra were recorded using a Perking Elmer Spectrum One FT-IR spectrometer under N_2 flow. GC-MS measurements were performed on a Perkin Elmer Clarus 680 GC (column PE, Elite 5MS, 15 m x 0.25 mm ID x 0.25 µm) equipped with Clarus SQ8T MS and analyzed with TurboMass software. Elemental analysis was conducted by Medac Ltd, Surret, United Kingdom.

1.3 Synthesis and characterization

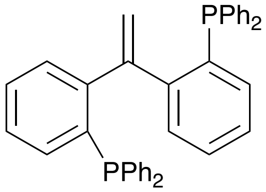

^{Ph}bppe^{H,H} Ligand (1): Methyltriphenylphosphonium bromide (MeTPPBr) (3.43 g, 9.6 mmol) was suspended in THF (20 mL) in a Schlenk tube equipped with a stirring bar. Subsequently, n-BuLi (6 mL, 1.6 M) was added dropwise to the tube leading to a red solution. After 30 minutes, a solution of 2,2'-bis(diphenylphosphino)benzophenone (2.3 g, 4.2 mmol) in THF (30 mL) was transferred dropwise during 15 min to the reaction mixture via syringe and the mixture was left stirring for 16 h. The reaction mixture was quenched with a saturated $NaHCO_3$ solution (50 mL), and the organic phase was recovered. Then, the organic phase was washed with brine (50 mL). The aqueous phase was extracted three times with Et_2O (3 x 50 mL). All the organic fractions were combined and dried over Na_2SO_4. The solvent was evaporated, and the obtained pale-yellow powder was washed with cold MeOH until the solvent was colorless. The product was dried overnight in the vacuum line. It was obtained 2.1 g of a white powder with 92 % yield.
^{1}H NMR (400 MHz, C_6D_6, 25 ºC) δ(ppm) 7.76–7.59 (m, 1H, Ar-*H*), 7.35 (tt, *J*= 6.5, 2.5 Hz, 9H, Ar-*H*), 7.26 (dd, *J*= 7.9, 2.2 Hz, 2H, Ar-*H*), 6.97 (dd, *J*= 5.4, 1.8 Hz, 14H, Ar-*H*), 6.87 (td, *J*= 7.5, 1.4 Hz, 2H, Ar-*H*), 5.35 (s, 2H, =CH_2).
$^{13}C\{^{1}H\}$ NMR (101 MHz, C_6D_6): δ(ppm) 150.0–148.9 (m, Ar), 148.2 (t, *J*= 6.9 Hz, *C*=CH_2), 139.5–138.8 (m, Ar), 136.7 (dd, *J*= 8.8, 5.2 Hz, Ar), 135.9 (s, Ar), 134.9–133.5 (m, Ar), 130.8 (s, Ar), 128.8 (s, Ar), 128.6 (d, *J*= 6.3 Hz, Ar), 128.4 (Ar), 127.6 (Ar), 123.4 (t, *J*= 5.5 Hz, C=CH_2).
$^{31}P\{^{1}H\}$ NMR (162 MHz, C_6D_6, 25 ºC) δ(ppm) -13.64 (s, 2P).
IR (cm^{-1}): 3070, 3045, 1584, 1468, 1431, 1089, 916, 741, 694, 502, 467, 417.
Elemental analysis: $C_{38}H_{30}P_2$, calculated: C, 83.20; H, 5.51. Found: C, 82.23; 5.49.

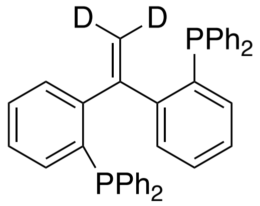

^{Ph}bppe^{D,D} Ligand (1b): Same procedure than ligand ^{Ph}bppe^{H,H} (**1**) described above using CD_3TPPBr. 1.73 g of product was isolated as a white powder with 75 % yield.
^{1}H NMR (400 MHz, C_6D_6, 25 ºC) δ(ppm) 7.34 (td, *J*= 6.8, 2.7 Hz, 9H, Ar-*H*), 7.28–7.23 (m, 2H, Ar-*H*), 7.01–6.91 (m, 14H, Ar-*H*), 6.86 (t, *J*= 7.5 Hz, 2H, Ar-*H*). Some signals are obscured by the residual deuterated solvent.
^{31}P{H^1} NMR (162 MHz, C_6D_6, 25 ºC) δ(ppm) -13.60 (s, 2P).

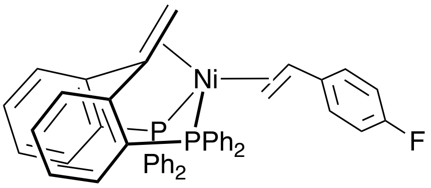

(^{Ph}bppe^{H,H})Ni(CH$_2$CHPhF) (2): Ligand ^{Ph}bppe^{H,H} (200 mg, 0.36 mmol) was placed in a vial and dissolved in toluene (10 mL). Ni(cod)$_2$ (100 mg, 0.36 mmol) dissolved in toluene (5 mL) was added in one portion. The reaction mixture was stirred for 5 min, then 4-fluorostyrene (44 µL, 0.36 mmol) was added into the solution with a micro-syringe. The mixture was stirred at room temperature for 4 hours. After this, the volume of solvent was reduced in half by evaporation and the product was precipitated using hexane (10 mL). The solid was filtered, washed with hexane (3 x 2 mL) and dried to yield 223 mg of an orange powder with 85 % yield. Crystals suitable for X-ray diffraction were obtained by vapor diffusion of hexane into a toluene solution of **2**.
^{1}H NMR (400 MHz, C_6D_6, 25 ºC): δ(ppm) 7.69 (dd, *J*= 7.7, 3.5 Hz, 1H, Ar-*H*), 7.50 (ddd, *J*= 7.8, 3.8, 1.2 Hz, 1H, Ar-*H*), 7.38 (ddt, *J*= 8.3, 5.5, 2.5 Hz, 2H, Ar-*H*), 7.35–7.25 (m, 5H, Ar-*H*), 7.26 –7.19 (m, 1H, Ar-*H*), 7.13–7.01 (m, 8H, Ar-*H*), 6.97 (dt, *J*= 10.0, 7.7 Hz, 2H, Ar-*H*), 6.93–6.83 (m, 3H, Ar-*H*), 6.83–6.73 (m, 2H, Ar-*H*), 6.64 (td, *J*= 7.6, 1.7 Hz, 2H, Ar-*H*), 6.58–6.47 (m, 4H, Ar-*H*), 4.39 (ddt, $J_{H,H}$= 12.2, 8.7, $J_{H,P}$= 3.0 Hz, 1H, CH$_2$=C*H*), 3.43 (t, $J_{H,P}$= 3.5 Hz, 1H, C*H$_2$*=C$_{ligand}$), 3.39 (ddd, $J_{H,H}$= 8.2, $J_{H,P}$=5.5, 2.0 Hz, 1H, *CH$_2$*=CH), 3.26 (ddd, $J_{H,H}$= 12.9, $J_{H,P}$= 8.2, 4.5 Hz, 1H, *CH$_2$*=CH), 3.04 (d, $J_{H,P}$= 6.2 Hz, 1H, C*H$_2$*=C$_{ligand}$).
^{13}C{H^1} NMR (101 MHz, C_6D_6, 25 ºC): δ(ppm) 161.3 (s, Ar), 158.8 (s, Ar), 156.0 (d, *J*= 34.9 Hz, Ar), 154.5 (d, *J*= 39.5 Hz, Ar), 144.3 (d, *J*= 30.0 Hz, Ar), 143.3 (s, Ar), 139.2–137.8 (m, Ar), 137.1–135.9 (m, Ar), 134.5 (d, *J*= 14.7 Hz, Ar), 133.3 (s, Ar), 133.1 (d, *J*= 13.9 Hz, Ar), 132.7 (s, Ar), 132.2 (d, *J*= 10.8 Hz, Ar), 129.7 (d, *J*= 14.4 Hz, Ar), 129.0 (d, *J*= 1.7 Hz, Ar), 128.7 (s, Ar), 128.4 (s, Ar), 127.2 (d, *J*= 3.9 Hz, Ar), 126.7 (d, *J*= 17.2 Hz, Ar), 126.0 (d, *J*= 3.4 Hz, Ar), 114.9 (d, *J*= 21.2 Hz, Ar), 103.9 (m, CH$_2$=*C$_{ligand}$*), 69.0 (d, *J*= 3.3 Hz, CH$_2$=*C*H), 67.9 (m, *C*H$_2$=C$_{ligand}$), 53.3 (s, *C*H$_2$=CH).
^{31}P{H^1} NMR (162 MHz, C_6D_6, 25 ºC): δ(ppm) 26.2 (dd, $J_{P,P}$= 33 Hz, $J_{P,F}$= 2.2 Hz, 1P), 24.8 (d, $J_{P,P}$= 33 Hz, 1P).
^{19}F NMR (376 MHz, C_6D_6, 25 ºC) δ(ppm) -120.37 (m).
IR (cm^{-1}): 3051, 2900, 2853, 1508, 1433, 1216, 1066, 909, 802, 742, 694, 540, 514, 488.
Elemental analysis calculated: $C_{46}H_{37}FNiP_2$. C, 75.74; H, 5.11. Found: C, 75.18; H, 5.15.

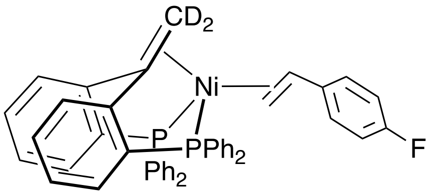

(^{Ph}bppe^{D,D})Ni(CH$_2$CHPhF) (2b): Same procedure as for (^{Ph}bppe^{H,H})Ni(CH$_2$CHPhF) described above using ligand ^{Ph}bppe^{D,D} (1b). The product was isolated as 211 mg of an orange powder with 80 % yield.
1H NMR (400 MHz, C_6D_6, 25 ºC): δ(ppm) 7.72–7.60 (m, 1H, Ar-H), 7.45 (dd, J= 7.7, 3.8 Hz, 1H, Ar-H), 7.37–7.21 (m, 6H, Ar-H), 7.17 (t, J= 7.0 Hz, 1H, Ar-H), 7.07–6.88 (m, 10H, Ar-H), 6.83 (dt, J= 15.0, 7.5 Hz, 2H, Ar-H), 6.78–6.68 (m, 3H, Ar-H), 6.59 (t, J= 7.5 Hz, 2H, Ar-H), 6.52–6.42 (m, 3H, Ar-H), 4.33 (ddd, JH,H= 9.19, 12.5 Hz, JH,P= 2.8, 1H, CH2=CH), 3.39–3.28 (m, 1H, CH2=CH), 3.21 (ddd, JH,P= 12.9, JH,H= 8.4, 4.6 Hz, 1H, CH2=CH). Some signals are obscured by the residual deuterated solvent.

31P{H1} NMR (162 MHz, C_6D_6, 25 ºC): δ(ppm) 26.27 (dd, JP,P= 32.9, JP,F= 2.3 Hz), 24.92 (d, JP-P= 32.8 Hz).
19F NMR (376 MHz, C_6D_6, 25 ºC): δ(ppm) -120.31 (m).

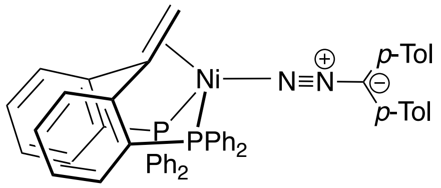

(^{Ph}bppe^{H,H})Ni(N_2C*p*-Tol$_2$) (3): In the glovebox, (^{Ph}bppe^{H,H})Ni(CH_2CHPhF) (20 mg, 0.027 mmol) was placed in a vial and dissolved in THF (3 mL). In a separate vial, bis(4-methylphenyl)diazomethane (12.2 mg, 0.054 mmol) was dissolved in THF (1 mL). Both vials were cooled down in the cold well at -78 ºC with an acetone/dry ice bath for 15 min. Afterwards, the diazoalkane solution was added dropwise to the complex solution in over 5 min. The mixture was stirred over 30 min in the cold well, after which the solvent was evaporated. This crude product was identified by ^{1}H NMR as a mixture of (^{Ph}bppe^{H,H})Ni(N_2C*p*-Tol$_2$) **3,** nickelacyclobutane **4** and diazoalkane. For NMR measurement, the mixture is dissolved in toluene and hexane is introduced by slow vapor diffusion of hexane at -35 ºC overnight. The supernatant was removed, and the solids were dissolved carefully in C_6D_6 allowing the identification of the methylene protons.
For IR, workup was performed to eliminate diazoalkane residues that could interfere with the measurement. The solvent of the reaction mixture was reduced to a quarter of the initial volume and cold hexane was added, causing precipitation. The precipitate was filtered and dissolved in C_6D_6. ^{1}H NMR was recorded corroborating no diazoalkane residues were present and showing a mixture of (^{Ph}bppe^{H,H})Ni(N_2Cp-tol$_2$) and nickelacyclobutane. Giving that the Nickelacyclobutane does not present a peaks in the diazo alkane area (2033 cm^{-1}), the peak was assigned to (^{Ph}bppe^{H,H})Ni(N_2Cp-tol$_2$). Crystals were grown by slow vapor diffusion of hexane into a toluene solution at -35 ºC.
^{1}H NMR (400 MHz, C_6D_6, 25 ºC): 4.14 (s, 2H, C*H$_2$*=C).
^{31}P{H^1} NMR (162 MHz, C_6D_6, 25 ºC): 19.39 (s, 2P).
IR [mixture of Nickelacyclobutane and (^{Ph}bppe^{H,H})Ni(N_2Cp-tol$_2$)] (cm^{-1}): 3052, 2967, 2924, 2855, 2033, 1512, 1435, 1260, 1197, 1108, 1066, 909, 812, 744, 694, 539.

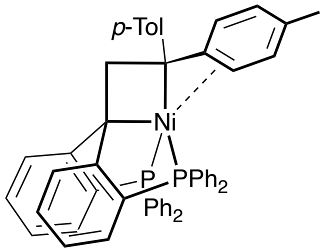

Nickelacyclobutane (4): (^{Ph}bppe^{H,H})Ni(CH_2CHPhF) (30 mg, 0.038 mmol) and bis(4-methylphenyl)diazomethane (12.2 mg, 0.054 mmol) were weighted in separated vials, dissolved in THF (3 mL) and cooled down to -78 ºC with an acetone/dry ice bath in the cold well of the glovebox. After 15 min, the diazoalkane solution was added dropwise to the stirred solution of complex **1** over 5 min. The mixture was stirred at low temperature for 30 min and then allowed to warm up to room temperature. The solvent was evaporated, and the residue was triturated with cold hexane in the cold well at -78 ºC. The dark red solid was filtered and washed with hexane until the solvent was colorless. Afterwards, the solid was washed with cold Et_2O (0.2 mL). The solid was then dissolved in THF at room temperature and transferred to a vial. The solvent is evaporated leading to 24 mg of a red-brown powder with 80 % yield. To obtain crystals suitable for X-ray diffraction, an open vial containing a saturated solution of **4** in Et_2O was placed in a sealed vial containing dry MeCN; upon standing at room temperature, slow concentration by vapor diffusion of Et_2O into MeCN yielded suitable crystals.
^{1}H NMR (400 MHz, C_6D_6, 25 ºC): δ(ppm) 8.41–8.27 (m, 2H, Ar-*H*), 7.75 (d, *J*= 7.2 Hz, 1H, Ar-*H*), 7.31 (t, *J*= 7.6 Hz, 2H, Ar-*H*), 7.25 (d, *J*= 6.9 Hz, 2H, Ar-*H*), 7.04 (s, 4H, Ar-*H*), 7.0–6.87 (m, 14H, Ar-*H*), 6.66 (t, *J*= 7.4 Hz, 2H, Ar-*H*), 6.53 (t, *J*= 7.6 Hz, 5H, Ar-*H*), 6.47–6.24 (m, 5H, Ar-*H*), 4.40 (s, 2H, C*H$_2$*=C), 1.76 (s, 6H, 2C*H$_3$*).
^{13}C{H^1} NMR (101 MHz, C_6D_6, 25 ºC): δ(ppm) 164.8 (b, Ar), 135.1 (s, Ar), 133.5 (s, Ar), 132.5 (d, *J*= 12.2 Hz, Ar), 132.1 (d, *J*= 12.3 Hz, Ar), 131.2 (s, Ar), 130.8 (s, Ar), 130.2 (s, Ar), 129.9 (d, *J*= 13.3 Hz, Ar), 129.0 (s, Ar), 128.5 (s, Ar), 127.6 (s, Ar), 127.1 (s, Ar), 125.8 (d, *J*= 4.1 Hz, Ar), 52.6 (s, C$H_{2,metallacycle}$), 28.9 (t, *J*= 9.3 Hz, $Q_{metallacycle}$), 21.4 (s, 2CH_3), 14.2 (t, *J*= 9.1 Hz, $Q_{metallacycle}$).
^{31}P{H^1} NMR (162 MHz, C_6D_6, 25 ºC): δ(ppm) 40-20 ppm (b, 2P).

^{1}H NMR (400 MHz, d^{8}-tol, -40 ºC): δ(ppm) 8.34 (s, 2H, Ar-*H*), 8.16 (s, 1H, Ar-*H*), 7.48–7.19 (m, 8H, Ar-*H*), 7.14 (s, 3H, Ar-*H*), 7.06 (s, 2H, Ar-*H*), 6.97 (s, 1H, Ar-*H*), 6.87 (s, 2H, Ar-*H*), 6.79 (s, 2H, Ar-*H*), 6.66 (s, 3H, Ar-*H*), 6.58 (d, *J*= 8.8 Hz, 4H, Ar-*H*), 6.45 (d, *J*= 7.9 Hz, 2H, Ar-*H*), 6.29 (s, 4H, Ar-*H*), 6.08 (s, 1H, Ar-*H*), 5.35 (s, 1H, coordinated tolyl-*H*), 4.41 (b, 1H, $CH_{2,metallacycle}$), 4.35 (b, 1H, $CH_{2,metallacycle}$), 1.88 (s, 3H, CH_3), 1.64 (s, 3H, CH_3).
^{13}C{^{1}H} NMR (101 MHz, d^{8}-tol, -40 ºC): δ(ppm) 166.2 (b, Ar), 163.2 (b, Ar), 144.8 (s, Ar), 142.9 (s, Ar), 140.6 (d, *J*= 54.1 Hz, Ar), 138.8 (s, Ar), 137.6 (s, Ar), 137.0 (s, Ar), 135.0 (d, *J*= 46.3 Hz, Ar), 133.2 (s, Ar), 132.8 (d, *J*= 13.2 Hz, Ar), 132.2 (s, Ar), 131.7 (s, Ar), 130.7 (s, Ar), 126.9 (s, Ar), 125.8 (s, Ar), 105.5 (s, coordinated tolyl-*H*), 85.6 (s, coordinated tolyl), 52.2 (s, $CH_{2,metallacycle}$), 29.1 (d, *J*= 18.2 Hz, $Q_{metallacycle}$), 14.31(d, *J*= 18.5 Hz, $Q_{metallacycle}$). Some signals are obscured by the solvent.
^{31}P{^{1}H} NMR (162 MHz, d^{8}-tol, -40 ºC): δ(ppm) 44.9 (d, $J_{P,P}$= 77 Hz), 21.9 (d, $J_{P,P}$= 77 Hz).
IR (cm^{-1}): 3050, 2962, 2920, 1432, 1259, 1066, 1026, 908, 813, 738, 691, 565, 519.
The high sensitivity of the compound did not allow to obtain elemental analysis data.

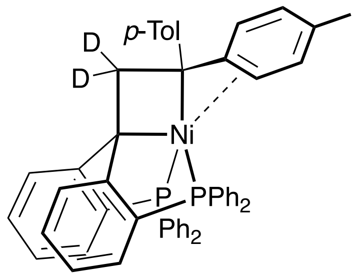

d^{2}-Nickelacyclobutane (4b): Same procedure as nickelacyclobutane complex **4** described above using the deuterated complex **2b** as precursor. 25 mg of product was isolated as a red-brown powder with 82 % yield.
^{1}H NMR (400 MHz, C_6D_6, 25 ºC): δ(ppm) 8.37–8.29 (m, 3H, Ar-*H*), 7.77 (t, *J*= 7.8 Hz, 2H, Ar-*H*), 7.69 (d, *J*= 4.1 Hz, 1H, Ar-*H*), 7.42 (s, 1H), 7.38–7.28 (m, 4H, Ar-*H*), 7.27–7.20 (m, 5H, Ar-*H*), 6.92 (d, *J*= 5.1 Hz, 10H, Ar-*H*), 6.88–6.62 (m, 9H, Ar-*H*), 6.52 (t, *J*= 7.5 Hz, 5H, Ar-*H*), 6.40 (s, 7H, Ar-*H*), 1.76 (s, 6H, 2CH_3). Some signals are obscured by the solvent.
^{31}P{^{1}H} NMR (162 MHz, C_6D_6, 25 ºC): δ(ppm) 40-20 ppm (b, 2P).

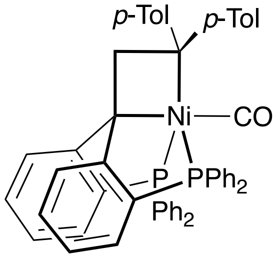

Generation of 4-CO: A solution of nickelacyclobutane complex **4** (10 mg) in toluene (0.5 mL) was placed in a Young NMR tube. The tube was connected to a gas setup and degassed by two freeze-pump-thaw cycles. With the toluene solution frozen, CO was introduced. Addition of CO to the tube leads to an immediate change of color from dark red to bright red associate to ligand exchange. The solution was transferred to a vial inside of the glovebox in approximately 10 min and dried under vacuum for 20 min. The red solid was washed with hexane (3 x 0.5 mL) and dried under vacuum. Giving the instability of the complex, no yield was estimated. A small quantity of cyclopropane $Ni(CO)_2$ (**5**) can be observed in IR (see SI section 3.2).
^{1}H NMR (400 MHz, C_6D_6, 25 ºC): δ(ppm) 7.82 (d, *J*= 8.0 Hz, 2H, Ar-*H*), 7.31 (d, *J*= 7.9 Hz, 4H, Ar-*H*), 7.06 (s, 3H, Ar-*H*), 6.98 (t, *J*= 7.3 Hz, 3H, Ar-*H*), 6.90 (dd, *J* = 15.4, 7.9 Hz, 8H, Ar-*H*), 6.81 (t, *J*= 7.3 Hz, 3H, Ar-*H*), 6.73 (d, *J*= 7.9 Hz, 6H, Ar-*H*), 6.58 (t, *J*= 7.6 Hz, 4H, Ar-*H*), 4.78 (s, 2H, CH_2), 2.07 (s, 6H, 2CH_3). Some signals are obscured by the residual solvent peak.
^{31}P{^{1}H} NMR (162 MHz, C_6D_6, 25 ºC): δ(ppm) 33.9 (s, 2P).
IR [mixture of **4-CO** and complex **5**) (cm^{-1}): 3053, 2933, 2855, 1984 (CO vibration of 4-CO), 1944 (CO vibration of 5), 1433, 1088, 809, 741, 692, 510.

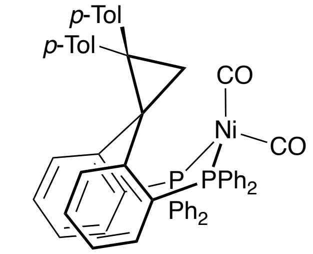

Cyclopropane Ni(CO)$_2$ (5): Nickelacyclobutane complex **4** (20 mg, 0.025 mmol) was dissolved in toluene and placed in a Young NMR tube. The tube was connected to a gas setup and degassed by two freeze-pump-thaw cycles. With the toluene solution frozen, CO was introduced. After 10 min, solution in the tube turned pink and after 16h yellow. The reaction mixture was transferred to a vial under N_2, filtered, and the solvent reduced to a quarter of the initial volume. Hexane was added to induce precipitation (1 mL), the solid was filtered and washed with hexane (3 x 0.5 mL). 15 mg of product was obtained as a pale-yellow powder with 72 % yield. Crystals suitable for X-ray diffraction were grown by vapor diffusion of hexane into a toluene solution at -35 ºC.
^{1}H NMR (400 MHz, C_6D_6, 25 ºC): δ(ppm) 7.85 (ddt, *J*= 9.7, 5.9, 1.6 Hz, 4H, Ar-*H*), 7.78 (ddd, *J*= 8.1, 4.7, 1.4 Hz, 2H, Ar-*H*), 7.15–7.07 (m, 6H, Ar-*H*), 6.93–6.83 (m, 12H, Ar-*H*), 6.82–6.74 (m, 4H, Ar-*H*), 6.69 (d, *J*= 8.0 Hz, 4H, Ar-*H*), 6.56 (ddd, *J* = 8.2, 7.2, 1.4 Hz, 2H, Ar-*H*), 3.35 (s, 2H, C*H$_2$*), 2.01 (s, 6H, 2C*H$_3$*).
^{13}C{H^1} NMR (101 MHz, C_6D_6, 25 ºC): δ(ppm) 201.2 (t, *J*= 1.9 Hz, *C*O), 196.3 (t, *J*= 6.4 Hz, *C*O), 149.2–147.9 (m, Ar), 142.5 (d, *J*= 31.7 Hz, Ar), 139.4 (s, Ar), 138.3 (d, *J*= 9.2 Hz, Ar), 138.0 (d, *J*= 9.4 Hz, Ar), 137.7 (s, Ar), 136.9 (s, Ar), 135.6 (d, *J*= 21.0 Hz, Ar), 134.7 (s, Ar), 133.8 (d, *J*= 15.3 Hz, Ar), 132.2 (d, *J*= 13.2 Hz, Ar), 131.1 (s, Ar), 129.3 (s, Ar), 128.7 (s, Ar), 128.6 (s, Ar), 128.3 (d, *J*= 2.1 Hz, Ar), 128.1 (d, *J*= 3.1 Hz, Ar), 127.9 (d, *J*= 8.1 Hz, Ar), 126.0 (s, Ar), 45.6 (d, *J*= 5.2 Hz, $Q_{cyclopropane}$), 40.9 (s, $Q_{cyclopropane}$), 36.1 (s, CH_2), 20.9 (s, CH_3).
^{31}P{H^1} NMR (162 MHz, C_6D_6, 25 ºC): δ(ppm) 26.2 (s, 2P)
IR (cm^{-1}): 3053, 2922, 2852, 2002, 1944, 1511, 1434, 743, 695, 518.
The high sensitivity of the compound did not allow to obtain elemental analysis data.

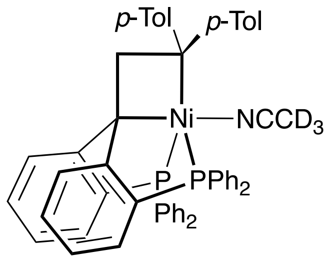

4-MeCN generated in situ with d^3-MeCN: In a young NMR tube was placed a solution of nickelacyclobutane complex **4** (10 mg) in d^8-tol (0.5 mL). A drop of d^3-MeCN was added on top of the solution and mixed carefully. Giving the instability of the complex, it could not be isolated.
^{1}H NMR (400 MHz, d^8-tol/d^3-MeCN, 25 ºC) δ 7.91 (b, 4H, Ar-*H*), 7.17 (b, 3H, Ar-*H*), 7.03–6.77 (m, 24H, Ar-*H*), 6.63 (b, 5H, Ar-*H*), 6.46 (b, 4H, Ar-*H*), 4.33 (s, 2H, C*H$_2$*), 1.93 (s, 6H, 2C*H$_3$*). Some signals are obscured by the residual solvent.
^{31}P{H^1} NMR (162 MHz, d^8-tol/d^3-MeCN, 25 ºC): δ(ppm) 20-15 (broad, 2P).
^{1}H NMR (400 MHz, d^8-tol/d^3-MeCN, -30 ºC) δ 7.71 (d, *J* = 8.1 Hz, 3H, Ar-*H*), 7.05–6.83 (m, 15H, Ar-*H*), 6.70 (d, *J* = 7.6 Hz, 11H, Ar-*H*), 6.64 (d, *J* = 7.7 Hz, 7H, Ar-*H*), 4.46 (s, 2H, C*H$_2$*), 2.21–1.94 (s, 6H, 2C*H$_3$*). Some signals are obscured by the residual solvent.
^{31}P{H^1} NMR (162 MHz, d^8-tol/d^3-MeCN, -30 ºC): δ(ppm) 15.7 (s, 2P).

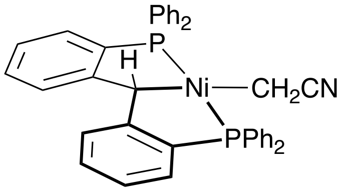

(^{Ph}PCHP^{Ph})Ni(CH$_2$CN) (6): Nickelacyclobutane complex **4** (20 mg, 0.025 mmol) was dissolved in MeCN (7 mL). The reaction mixture was stirred for 1 hour and a yellow precipitate was observed. The solvent was removed by decantation

and the solid was washed with hexane (3 x 1 mL). After drying, 13 mg of a yellow powder was obtained with 85 % yield. Crystals suitable for X-ray diffraction were obtained by evaporation of a saturated Et_2O solution.

^{1}H NMR (400 MHz, C_6D_6, 25 ºC): δ(ppm) 8.06 (q, *J*= 6.1 Hz, 4H, Ar-*H*), 7.37 (d, *J*= 6.3 Hz, 4H, Ar-*H*), 7.02 (t, *J*= 7.2 Hz, 5H, Ar-*H*), 6.94 (t, *J*= 7.6 Hz, 2H, Ar-*H*), 6.88 (dd, *J*= 10.6, 7.3 Hz, 5H, Ar-*H*), 6.74 (t, *J*= 7.6 Hz, 2H, Ar-*H*), 5.37 (s, 1H, C*H*), 0.70 (t, $J_{H,P}$= 9.0 Hz, 2H, CH_2CN). Some signals are obscured by the residual deuterated solvent peak.

^{13}C{^{1}H} NMR (101 MHz, C_6D_6, 25 ºC): δ(ppm) 158.3 (t, *J*= 20.2 Hz, Ar), 138.3 (t, *J*= 23.4 Hz, Ar), 134.3 (t, *J*= 6.8 Hz, Ar), 133.0 (t, *J*= 5.7 Hz, Ar), 132.2 (s, Ar), 132.1 (t, *J*= 21.0 Hz, Ar), 131.2 (t, *J*= 20.1 Hz, Ar), 129.9 (s, Ar), 129.2 (t, *J*= 5.0 Hz, Ar), 128.7 (t, *J*= 4.7 Hz, Ar, Ar), 127.4 (s, Ar), 125.3 (s, Ar), 49.7 (s, *C*H), -16.6 (*C*H_2CN). Some signals are obscured by the residual solvent peak.

^{31}P{^{1}H} NMR (162 MHz, C_6D_6, 25 ºC): δ(ppm) 39.0 (s, 2P).

IR (cm^{-1}): 3048, 2979, 2925, 2100, 2080, 2004, 1510, 1433, 1219, 1090, 825, 769, 743, 695, 540.

The high sensitivity of the compound did not allow to obtain elemental analysis data.

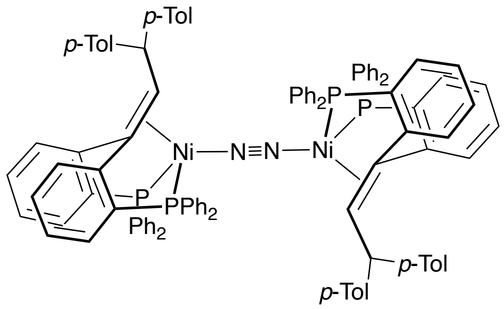

[(^{Ph}bppe^{H,CHptol2})Ni]$_2$N$_2$ (7): Nickelacyclobutane complex **4** (20 mg, 0.025 mmol) was dissolved in toluene and placed in a young NMR tube. The tube was heated at 70 ºC for 16 h. Afterwards, the solution was transfer to a vial inside of the glovebox and the solvent was evaporated. The resulting solid was washed with cold hexane (3 x 0.5 mL) and dried to obtain 18 mg of a yellow powder with 88 % yield. Additionally, the complex can be synthesized from nickelacyclobutane **4** in toluene at room temperature with a reaction time longer than 24 h.

Crystals suitable for X-ray diffraction were grown by vapor diffusion of hexane in a saturated toluene solution.

^{1}H NMR (400 MHz, C_6D_6, 25 ºC): δ(ppm) 7.76–7.67 (m, 8H, Ar-*H*), 7.64 (dd, *J*= 8.1, 3.7 Hz, 2H, Ar-*H*), 7.38 (dd, *J*= 7.7, 3.3 Hz, 2H, Ar-*H*), 7.23–7.13 (m, 12H, Ar-*H*), 7.09 (s, 4H, Ar-*H*), 6.99–6.85 (m, 25H, Ar-*H*), 6.85–6.75 (m, 11H, Ar-*H*), 6.72 (d, *J*= 7.8 Hz, 3H, Ar-*H*), 6.66 (tt, *J*= 7.6, 3.8 Hz, 5H, Ar-*H*), 4.69 (b, 2H, C*H*=C), 4.05 (d, $J_{H,H}$= 10.6 Hz, 2H, =C-C*H*), 2.07 (s, 6H, 2CH_3), 2.06 (s, 6H, 2CH_3).

^{13}C{^{1}H} NMR (101 MHz, C_6D_6, 25 ºC): δ(ppm) 156.3 (d, *J*= 38.8 Hz, Ar), 151.7 (d, *J*= 40.4 Hz, Ar), 145.8 (s, Ar), 138.7 (d, *J*= 16.5 Hz, Ar), 138.2–137.7 (m, Ar), 136.3 (d, *J*= 30.7 Hz, Ar), 134.8 (d, *J*= 15.3 Hz, Ar), 134.3 (s, Ar), 133.6 (d, *J*= 20.7 Hz, Ar), 133.2 (s, Ar), 132.6 (s, Ar), 126.5 (d, *J*= 13.0 Hz, Ar), 100.0 (s, *C*=CH), 81.5 (b, =*C*-CH), 55.35 (s, =C-*C*H), 21.1 (m, *C*H$_3$).

^{31}P{^{1}H} NMR (162 MHz, C_6D_6, 25 ºC): δ(ppm) 27.8 (d, $J_{P,P}$= 55 Hz), 12.3 (d, $J_{P,P}$= 55 Hz).

IR (cm^{-1}): 2956, 2923, 2853, 1509, 1468, 1435, 1260, 1095, 1023, 806, 745, 694, 543, 499.

The high sensitivity of the compound did not allow to obtain elemental analysis data.

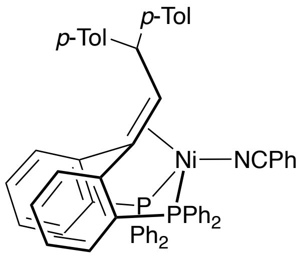

(^{Ph}bppe^{H,CHptol2})Ni(PhCN) (8): [(^{Ph}bppe^{H,CHptol2})Ni]$_2$N$_2$ complex (20 mg, 0.012 mmol) was dissolved in toluene and benzonitrile (3 mg, 0.03 mmol) was added to the solution. The reaction mixture turned red and was stirred for 20 min. The solvent was evaporated, the resulting solid was washed with cold hexane (2 x 0.5 mL) and dried. 20 mg of the product was obtained as a dark red powder with 95% yield. Crystals were grown by vapor diffusion of hexane in a saturated toluene solution at -35 ºC.

^{1}H NMR (400 MHz, C_6D_6, 25 ºC): δ(ppm) 8.06–7.97 (m, 3H, Ar-*H*), 7.86 (dd, *J*= 8.0, 3.7 Hz, 1H, Ar-*H*), 7.45 (dt, *J*= 5.8, 2.9 Hz, 1H, Ar-*H*), 7.39–7.25 (m, 6H, Ar-*H*), 7.21 (ddd, *J*= 14.2, 7.4, 5.7 Hz, 4H, Ar-*H*), 7.12 (d, *J*= 7.9 Hz, 2H, Ar-*H*), 7.06–6.97 (m, 5H, Ar-*H*), 6.94 (dd, *J*= 7.6, 5.8 Hz, 3H, Ar-*H*), 6.91–6.80 (m, 6H, Ar-*H*), 6.78 (dd, *J*= 7.9, 1.7 Hz, 2H, Ar-*H*), 6.76–6.69 (m, 2H, Ar-*H*), 6.67 (d, *J*= 7.7 Hz, 2H, Ar-*H*), 4.56 (dd, $J_{H,H}$= 10.9, $J_{H,P}$= 3.6 Hz, 1H, C*H*=C), 4.14 (d, $J_{H,H}$= 10.9 Hz, 1H, =C-C*H*), 2.13 (s, 3H, C*H*$_3$), 1.72 (s, 3H, C*H*$_3$). Some signals are obscured by the residual ^{1}H peak of the deuterated solvent.
$^{31}P\{H^1\}$ NMR (162 MHz, C_6D_6, 25 ºC) δ 25.91 (d, $J_{P,P}$= 69.5 Hz), 11.00 (d, $J_{P,P}$= 69.5 Hz).
$^{13}C\{H^1\}$ NMR (101 MHz, C_6D_6, 25 ºC): δ(ppm) 158.0 (d, *J*= 42.9 Hz, Ar), 153.1 (d, *J*= 43.4 Hz, Ar), 146.8 (s, Ar), 145.8 (s, Ar), 145.4 (s, Ar), 140.4 (d, *J*= 10.4 Hz, Ar), 139.2 (s, Ar), 137.5 (d, *J*= 29.2 Hz, Ar), 134.2 (d, *J*= 4.1 Hz, Ar), 134.0 (s, Ar), 133.7 (s, Ar), 132.7–132.3 (m, Ar), 132.0 (d, *J*= 13.4 Hz, Ar), 130.9 (t, *J*= 2.7 Hz, Ar), 130.6 (d, *J*= 15.0 Hz, Ar), 129.9 (s, Ar), 128.8 (d, *J*= 15.0 Hz, Ar), 128.4 (d, *J*= 4.0 Hz, Ar), 127.5 (s, Ar), 127.1 (dd, *J*= 11.5, 3.5 Hz, Ar), 126.3 (d, *J*= 13.1 Hz, Ar), 125.4 (d, *J*= 3.1 Hz, Ar), 123.5 (s, Ar), 114.8 (t, *J*= 2.4 Hz, *C*N), 93.9 (s, *C*=CH), 77.2–73.6 (m, =*C*-CH), 55.8 (d, *J*= 5.5 Hz, =C-*C*H), 21.1(s, CH_3), 20.7 (s, *C*H$_3$).
IR (cm^{-1}): 3055, 2956, 2922, 2855, 2195, 1435, 1260, 1094, 1020, 801, 717, 543.
The high sensitivity of the compound did not allow to obtain elemental analysis data.

2. Additional experiments

2.1 Conversion of diazo adduct 3 to Nickelacyclobutane 4

It is possible to follow the progression of diazo adduct 3 to nickelacyclobutane analyzing a sample during the isolation procedure. If the sample is analyzed immediately after filtration (see isolation procedure in section 1.3), the spectra reveal a mixture of complexes 3 and 4. Figure S1 and S2 show the conversion of complex 3 to yield nickelacyclobutane 4 in a solid sample after filtration dissolved in C_6D_6. Overtime in a period of 30 min, compound 3 is mostly consumed and the concentration of nickelacyclobutane 4 increases.

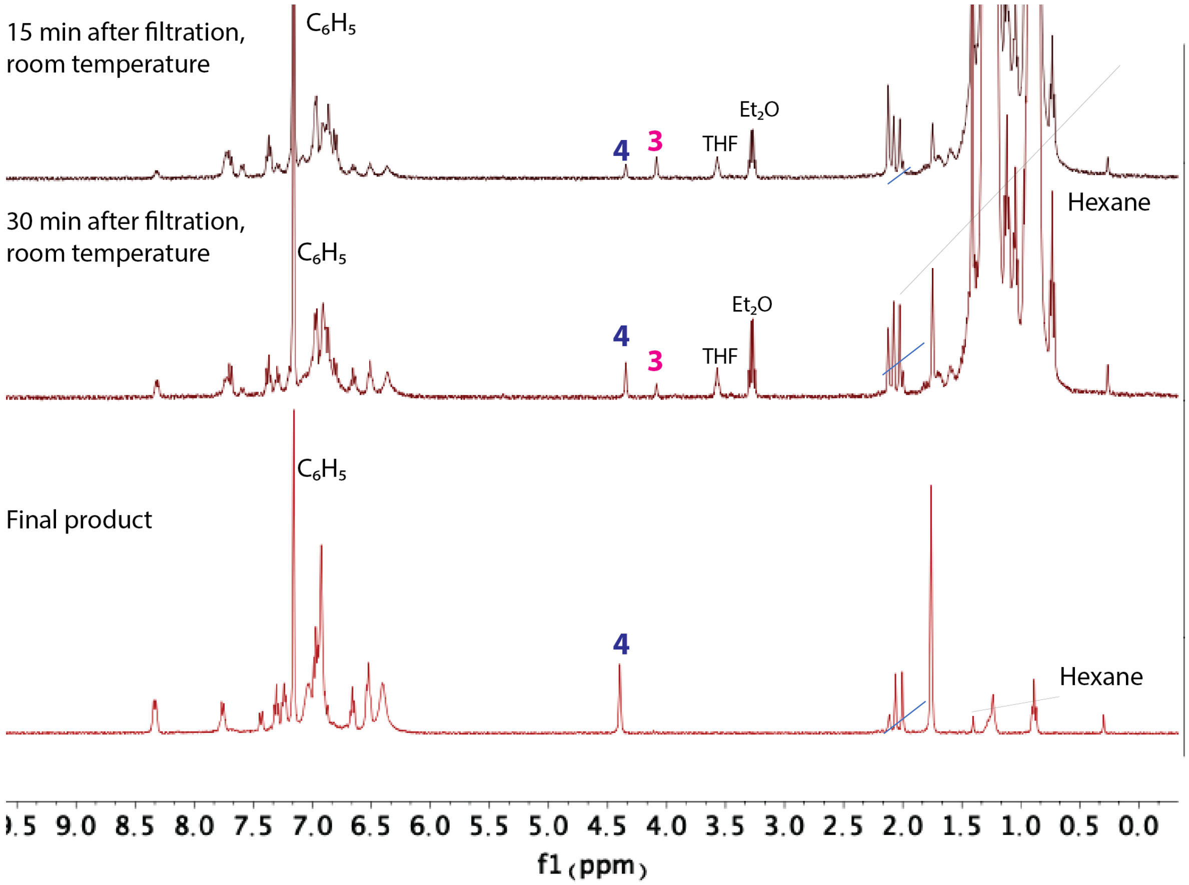

Figure S1. ^{1}H NMR of the conversion of intermediate 3 to nickelacyclobutane 4 overtime in C_6D_6. Solvents and a small impurity bis-(4,4'-dimethyl-benzhydrylidene)-hydrazine are crossed with a line.

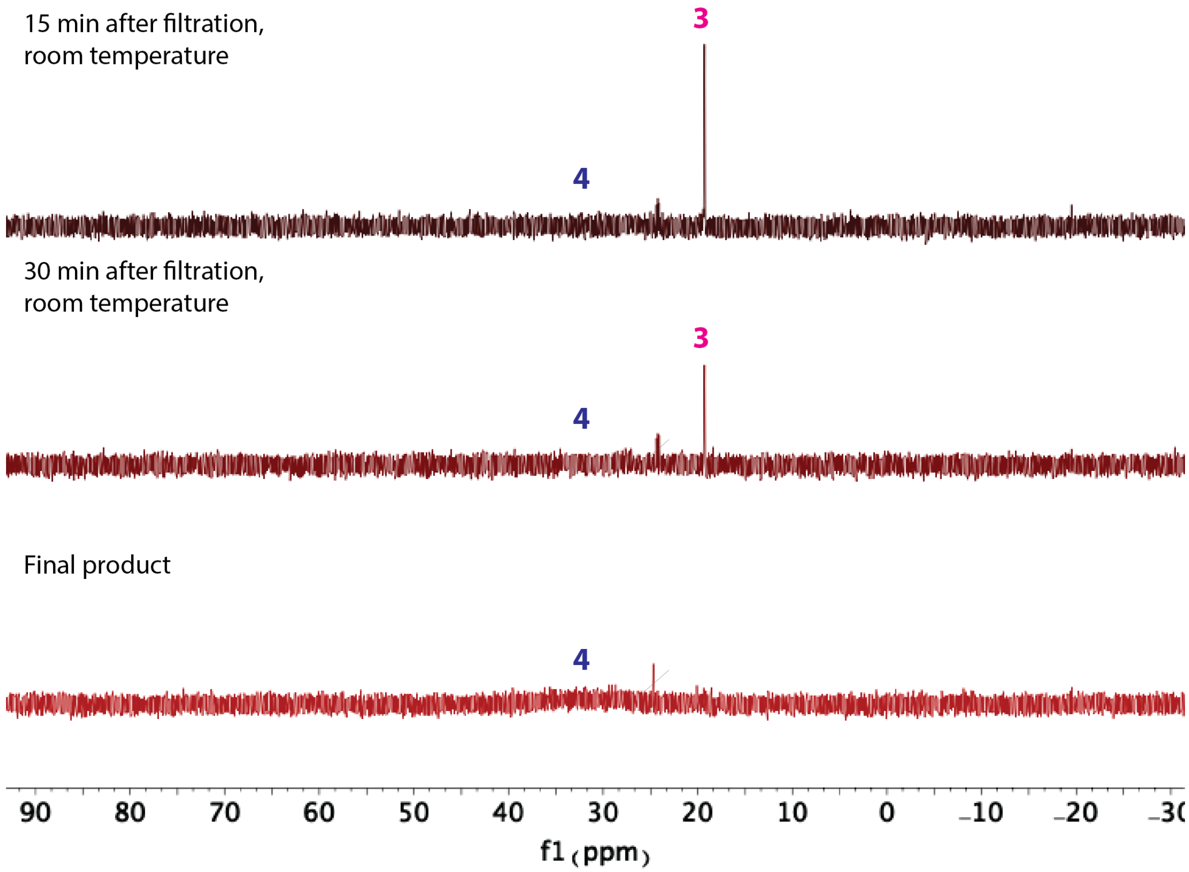

Figure S2. ^{31}P NMR of the conversion of intermediate 3 to nickelacyclobutane 4 overtime in C_6D_6.

2.2 VT-NMR of Nickelacyclobutane 4

Figure S3 depicts the variable temperature ^{1}H NMR analysis of complex 4 in d^{8}-toluene. At -40 ºC, the decrease of fluxionality allows to see the features expected for the unsymmetrical nickelacyclobutane structure observed in the solid state. The aromatic protons are separated as consequence of the loss of symmetry as well as the methyl groups from the tolyl fragments (found at ∂(ppm): 1.69 and 1.88). The proton in ortho position associated with the coordinated tolyl group is shifted upfield at ∂(ppm): 5.35. The methylene protons of the nickelacyclobutane are diasterotopic and result in broad overlapping signals at ∂(ppm): 4.37 and 4.41. A ^{31}P NMR spectrum recorded at -40 ºC (Figure S4) showed two doublets at ∂(ppm) 22.9 and 44.9 that are consistent with the unsymmetrical structure. Additionally, ^{1}H-EXSY (Hadamard) (Figure S5) displays the fluxional nature of complex 4 by the interchange of the methyl signals and aromatic signals at ∂(ppm) 8.3.

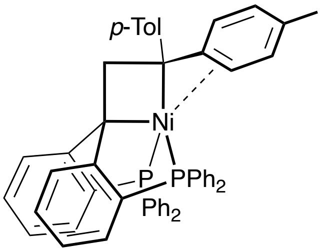

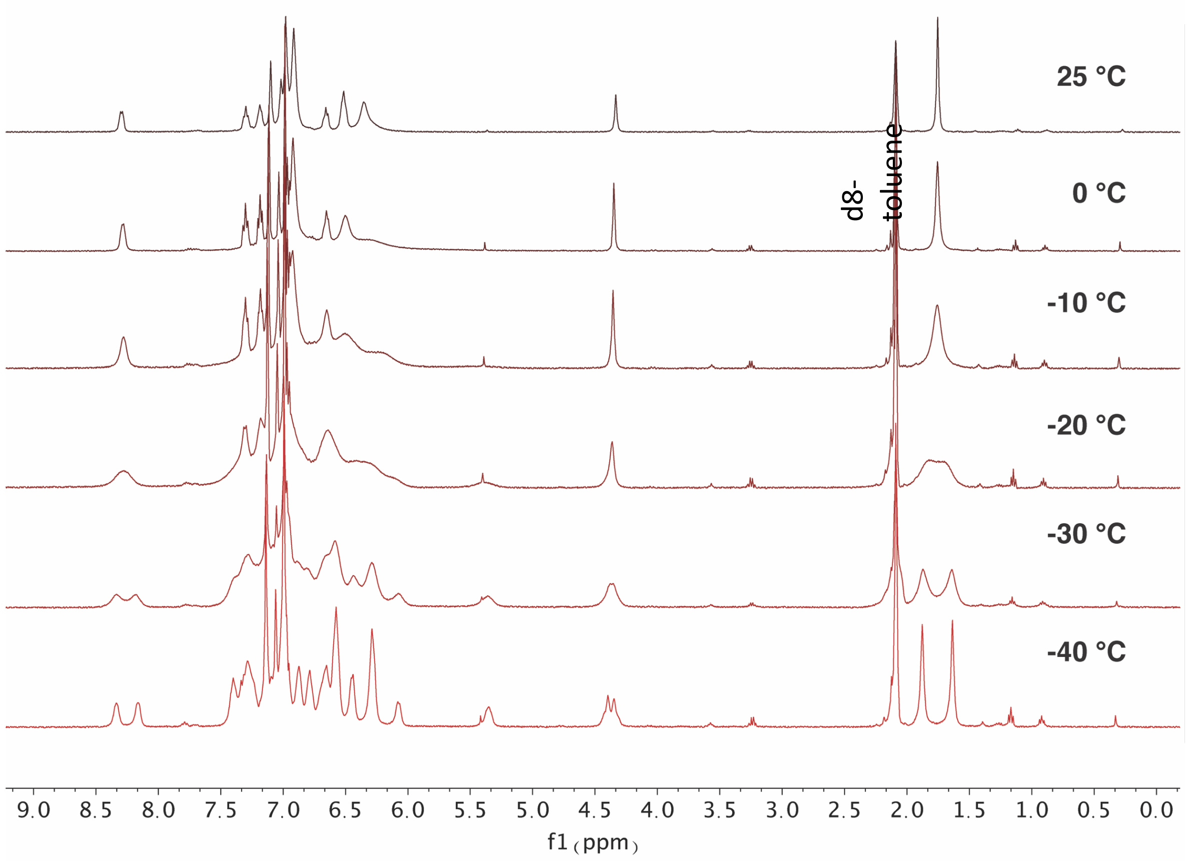

Figure S3. VT ^{1}H NMR Nickelacyclobutane in d^{8}-tol.

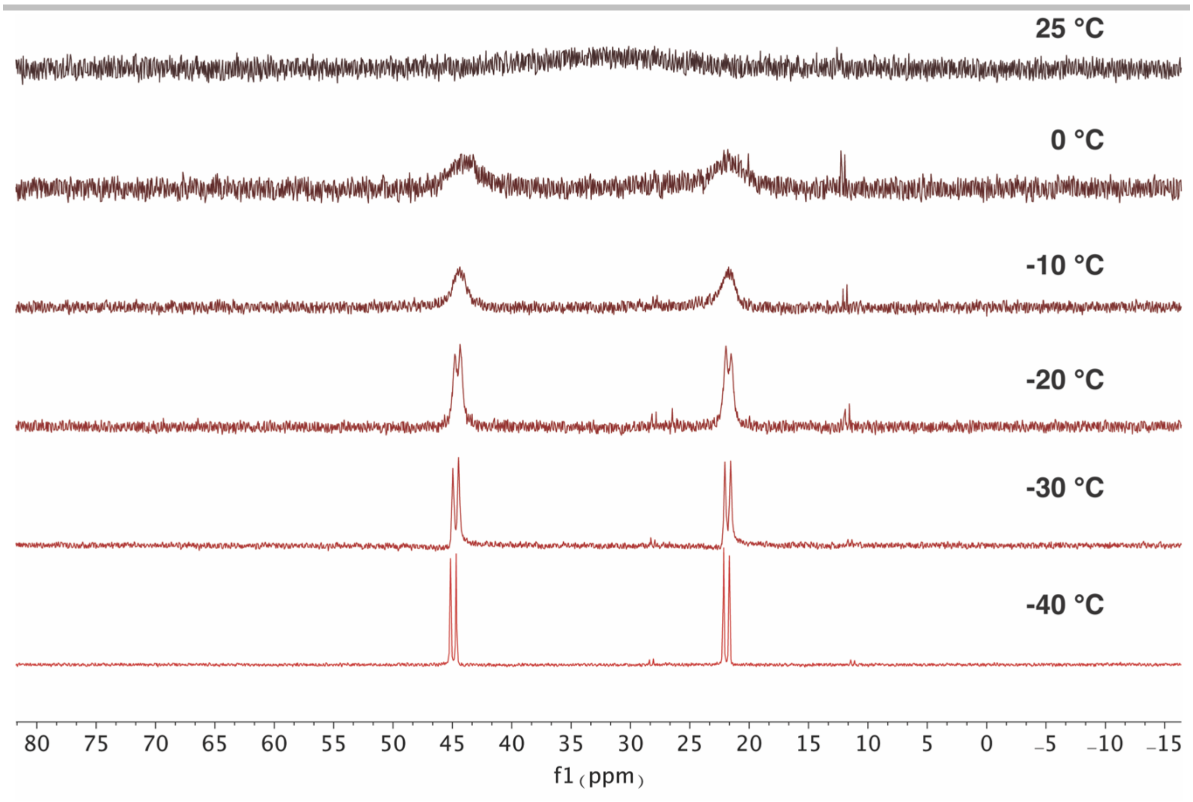

Figure S4. VT ^{31}P NMR Nickelacyclobutane in d^8-tol.

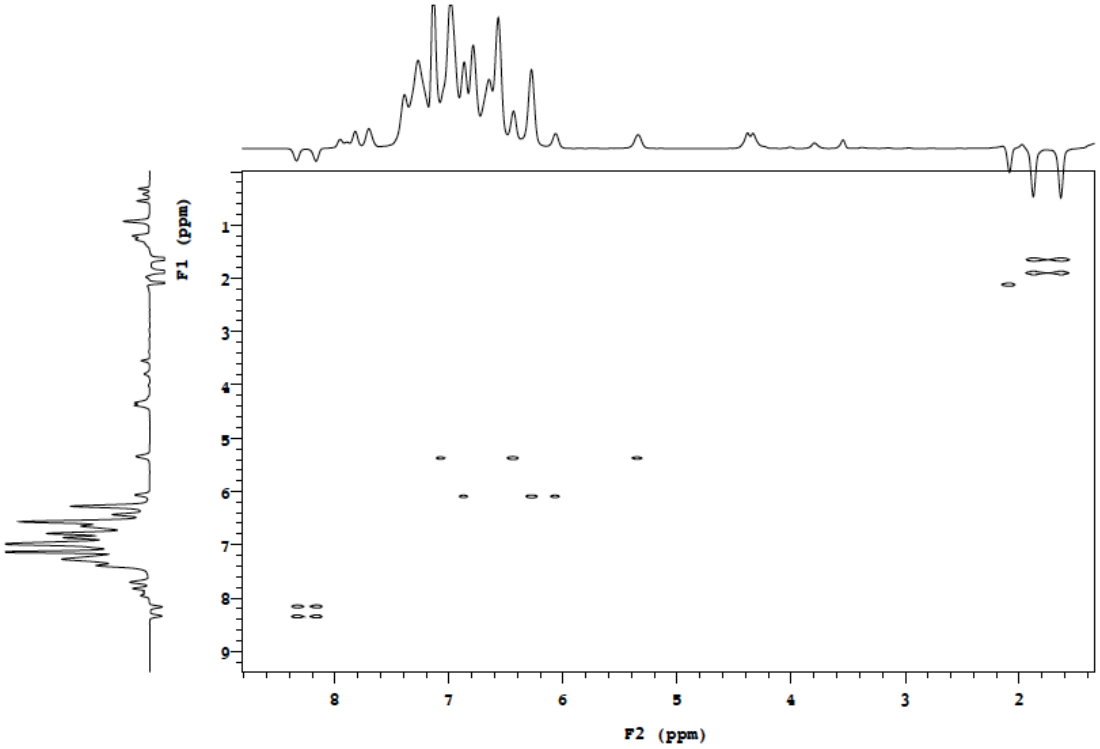

Figure S5. EXSY ^{1}H (Hadamard) of Nickelacyclobutane in d^8-tol.

Based on the Eyring equation, the transition state Gibbs energy of the fluxional movement can be defined as follows:[4]

$$\Delta G^{\neq} = (4.575x10^{-3})(T_c)(9.97 + log\frac{Tc}{\Delta v})$$

Where T_c is the coalescence temperature and Δv is the chemical shift difference in Hz.

$$\Delta G^{\neq} = (4.575x10^{-3})(253)(9.97 + log\frac{253}{97})$$

$$\Delta G^{\neq} = 12.0\ Kcal/mol$$

Where considering the estimated error calculated with 1 Hz and 5 K deviation on an error propagation calculator[5]:

$$\Delta G^{\neq} = 12.0 \pm 0.2\ Kcal/mol$$

2.3 IR analysis of intermediate 4-CO and reactivity under an N_2 atmosphere

Complex **4-CO** can be generated and isolated to obtain a clean ^{1}H and ^{31}P NMR (see SI section 1.3 for experimental procedure). However, the IR spectrum of the intermediate showed two peaks related with CO stretching. Comparison of the recorded IR spectrum with the IR spectrum of cyclopropane $Ni(CO)_2$ (**5**) suggested a small amount of complex **5** can be found already (Figures S6 and S7). The peak at 1984cm^{-1} is associated with the proposed Nickelacyclobutane(CO). Additionally, calculated C=O stretching frequencies at B3LYP-GDB3J/6-31g(d,p) level of theory are in good agreement with the experimental data (table 1).

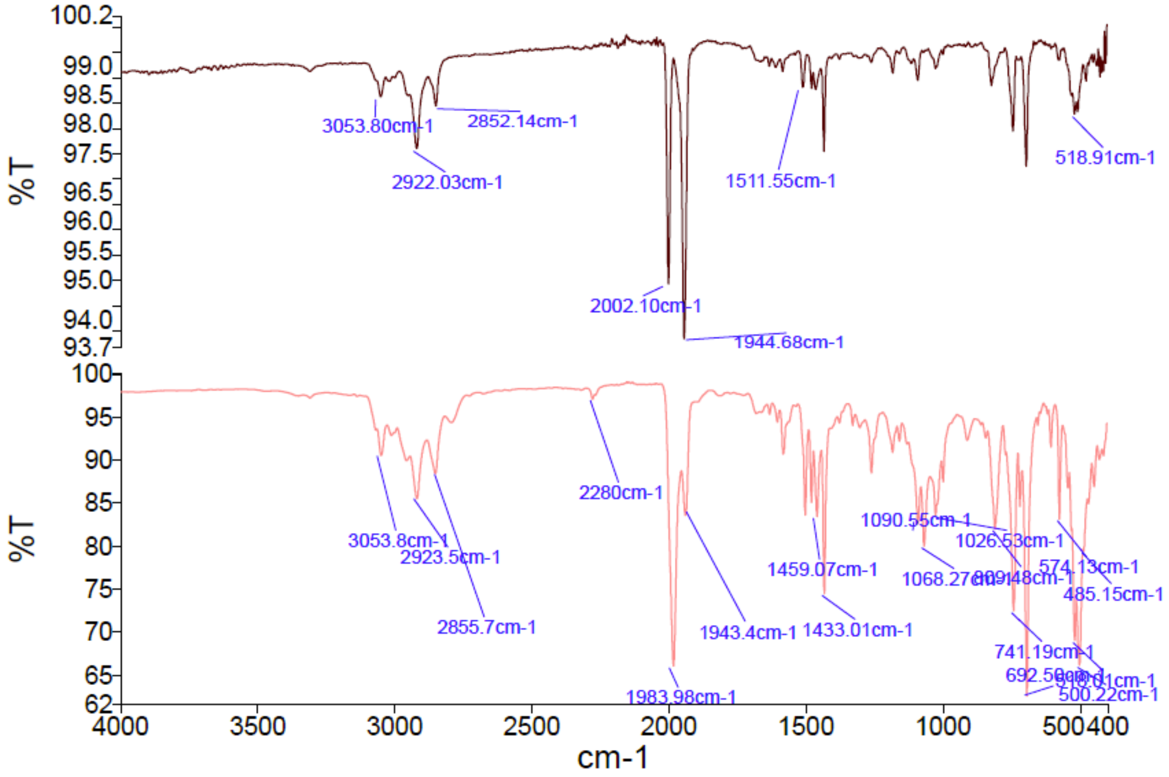

Figure S6. IR comparison of intermediate with CO (down) and complex **5** (on top).

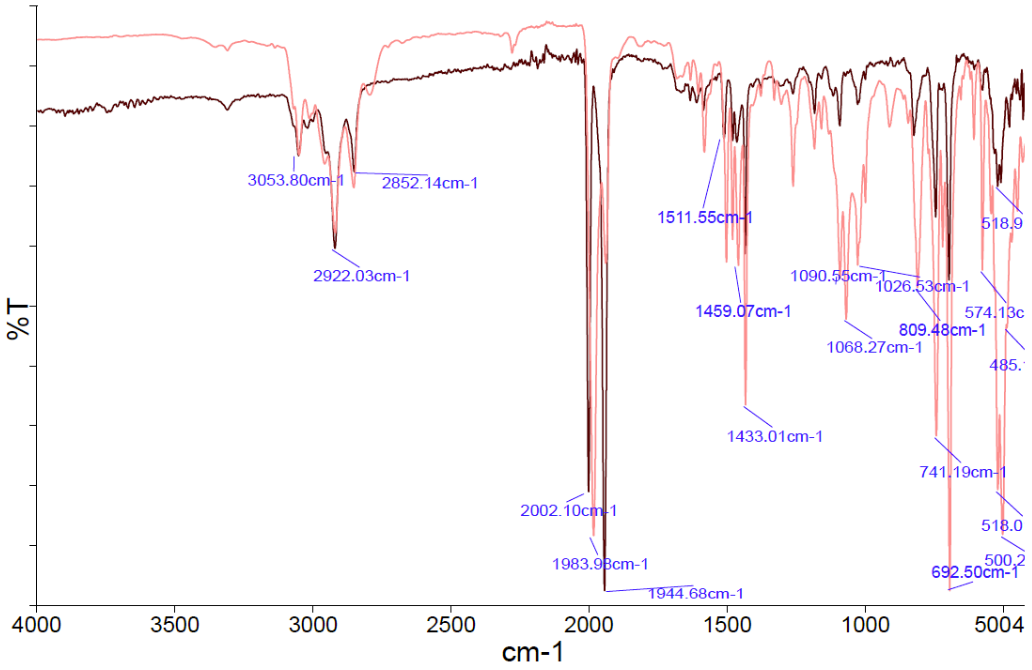

Figure S7. IR comparison (overlap) of intermediate with CO (pink) and complex **6** (red).

Table S1. Vibrational C=O stretch frequencies calculated at the B3LYP-GDB3J/6-31g(d,p) level of theory.

Complex	C=O stretch frequency (cm^{-1})
Nickelacyclobutane(CO)	2070
Cyclopropane $Ni(CO)_2$	2035 (asymmetrical)
Cyclopropane $Ni(CO)_2$	2082 (symmetrical)

The isolated complex **4-CO** was kept in a Young NMR tube under nitrogen atmosphere and analyzed after 16h. ^{1}H NMR (Figure S8) showed the presence of 1,1-di(p-tolyl)ethylene at ∂(ppm) 5.41. This correlates with the DFT calculations that suggest the metathesis pathway is thermodynamically available. Nevertheless, under a CO atmosphere cyclopropanation is preferred due to the formation of more stable species.

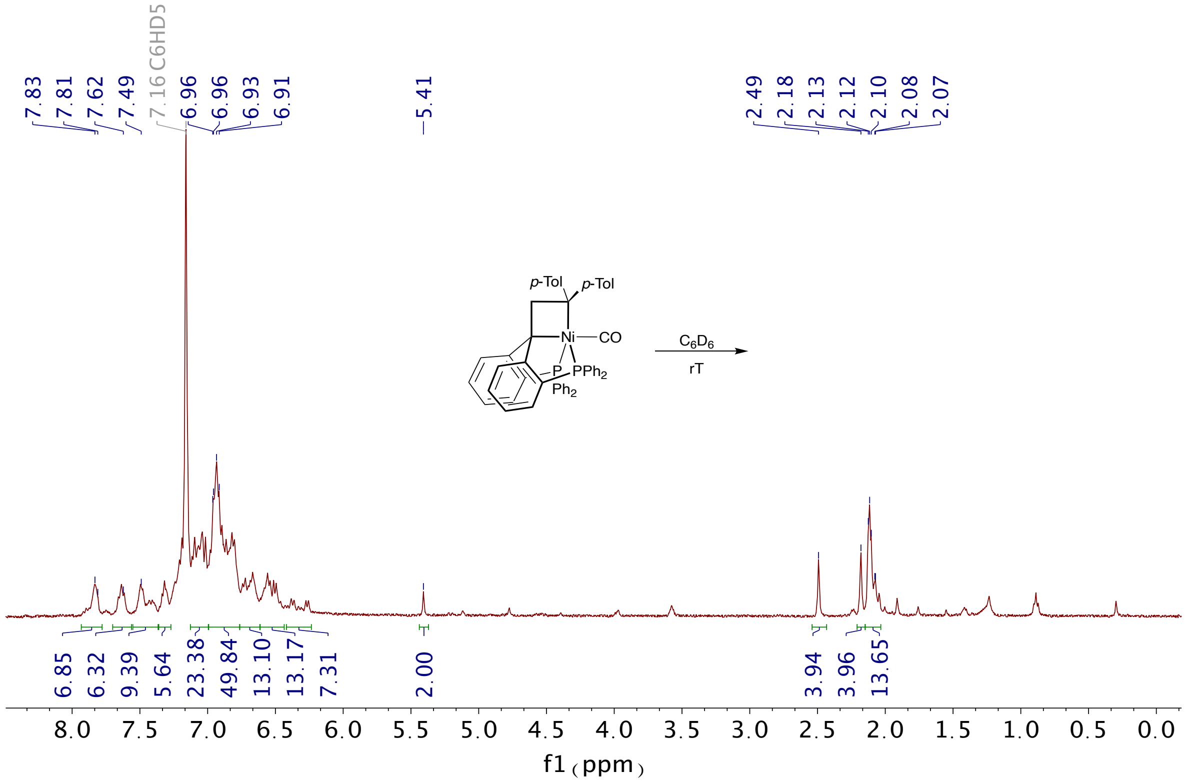

Figure S8. ^{1}H NMR of decomposition of the proposed Nickelacyclobutane(CO) in C_6D_6 at 25ºC after 16h. The peak at 5.41 ppm correspond to 1,1-di(p-tolyl)ethylene.

2.4 VT NMR of Nickelacyclobutane (4) in presence of d^3-MeCN. Analysis of intermediate 4-MeCN.

Addition of d^3-MeCN to a d^8-toluene solution of nickelacyclobutane leads to a change of coordination, where a molecule of d^3-MeCN displaces the C=C of the tolyl group. ^{1}H NMR (Figure S9) depicts broad signals, the methylene protons are presented at ∂(ppm) 4.33 and the methyl groups are shifted downfield at 1.93 ^{31}P NMR (figure S10) showed a broad signal between 12-18 ppm.

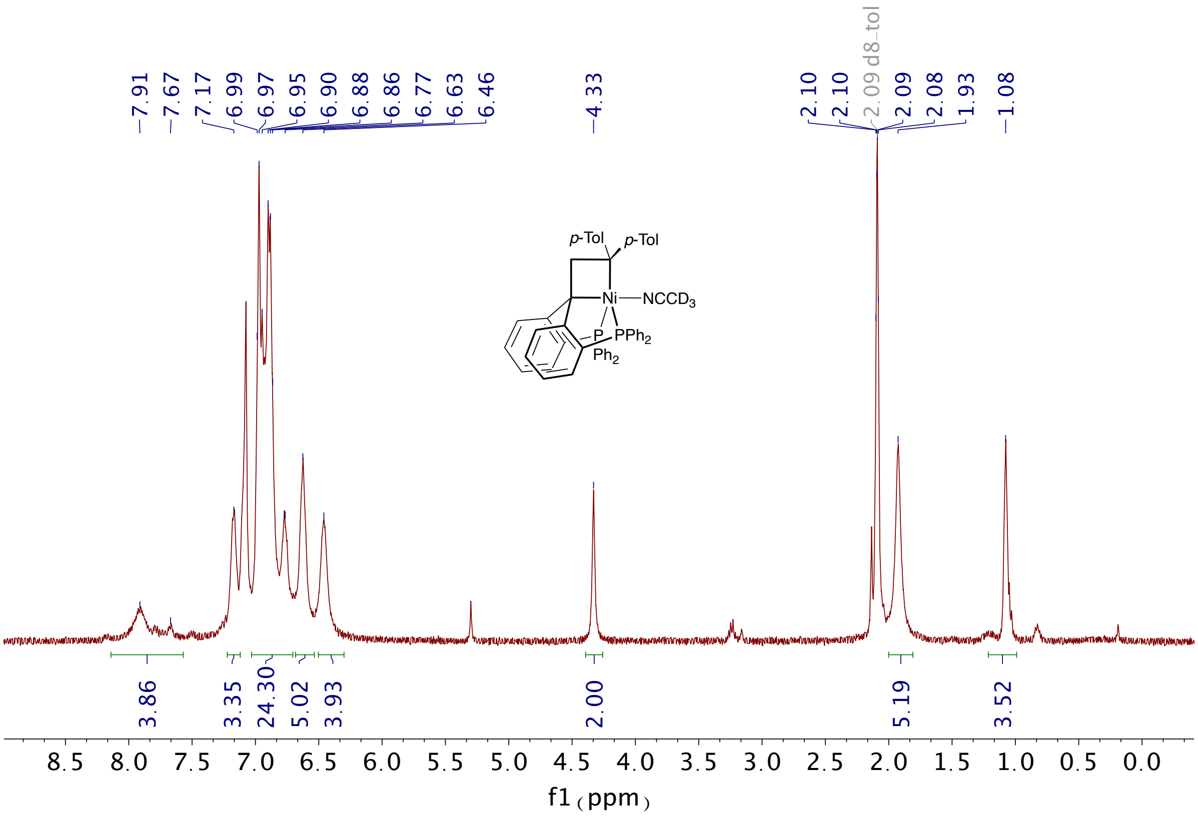

Figure S9. ^{1}H NMR Nickelacyclobutane with d^3-MeCN and d^8-tol at 25 ºC.

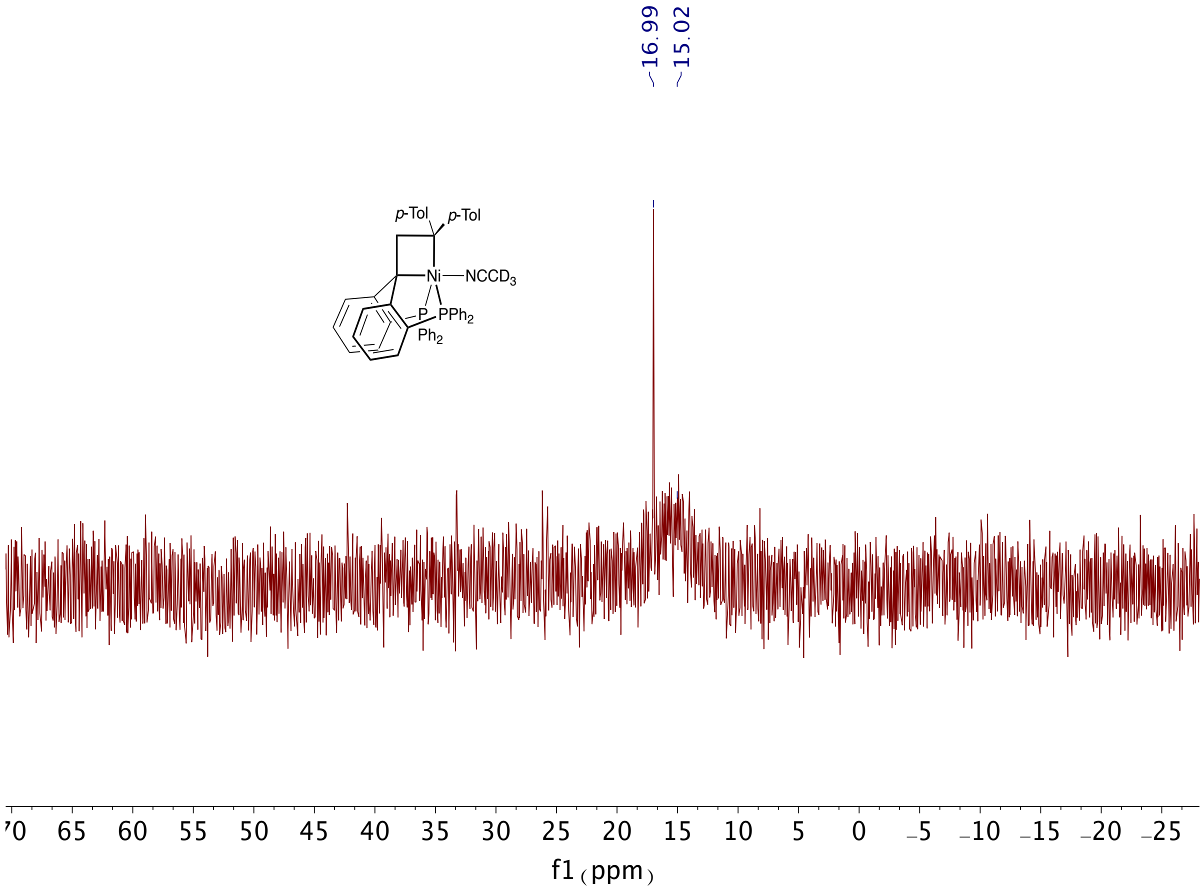

Figure S10. ^{31}P NMR Nickelacyclobutane with d^3-MeCN and d^8-tol at 25 ºC. The signal at 16.99 is an unknown impurity.

In Figure S11, VT ^1H NMR showed a better definition of the signals leading to a symmetrical structure of nickelacyclobutane(d^3-MeCN). Interestingly, the ^{31}P NMR peak sharpens at low temperatures (Figure S12). This could be explained by partial dissociation of the acetonitrile coligand at higher temperatures.

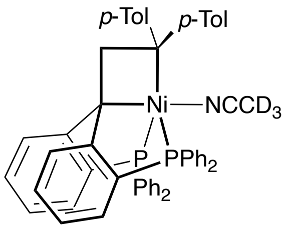

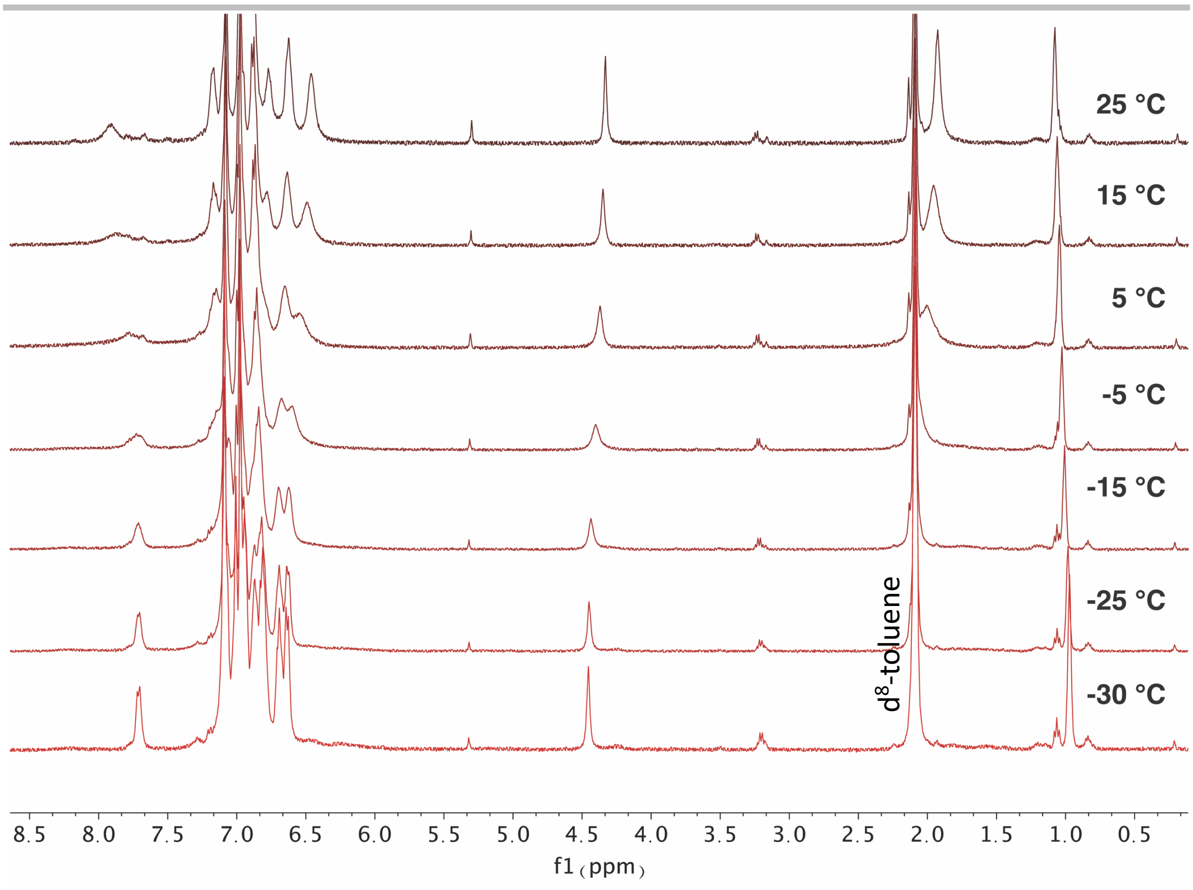

Figure S11. VT ^{1}H NMR Nickelacyclobutane with d^3-MeCN and d^8-tol. Peak at 5.4 ppm correspond to 1,1-di(p-tolyl)ethylene.

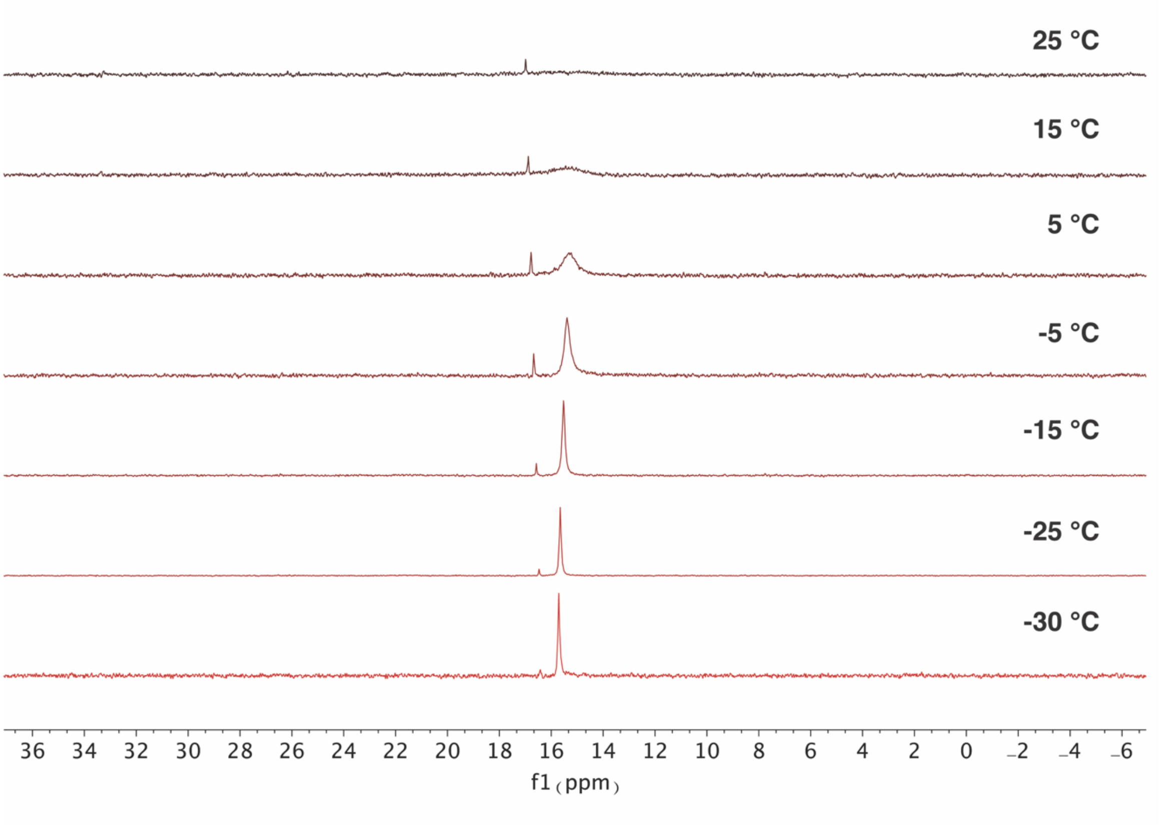

Figure S12. VT ^{31}P NMR Nickelacyclobutane with d^3-MeCN and d^8-tol.

The in situ generated Nickelacyclobutane(d^3-MeCN) species was kept in an Young NMR tube and analyzed after 16h. ^1H NMR and ^{31}P NMR (Figure S13 and S14) showed conversion to ($^{Ph}PCDP^{Ph}$)Ni(CD_2CN) and 1,1-di(p-tolyl)ethylene as main products, supporting this species as intermediate in the metathesis pathway. 1,1-di(p-tolyl)ethylene was identified by CG-MS ($[M]^+$= 208) and with ^1H NMR values reported in literature.[6]
Other minor product can be observed with peaks in ^1H NMR at ∂(ppm) 3.81 and 4.07. ^{31}P NMR signals at ∂(ppm) 8.5 and 26.1 present a similar pattern to [($^{Ph}bppe^{H,CHptol2}$)Ni]$_2N_2$.

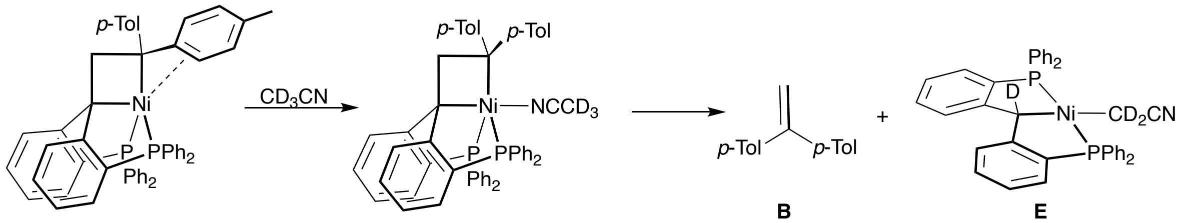

B E

Scheme S1. Formation and reactivity of 4-CD_3CN.

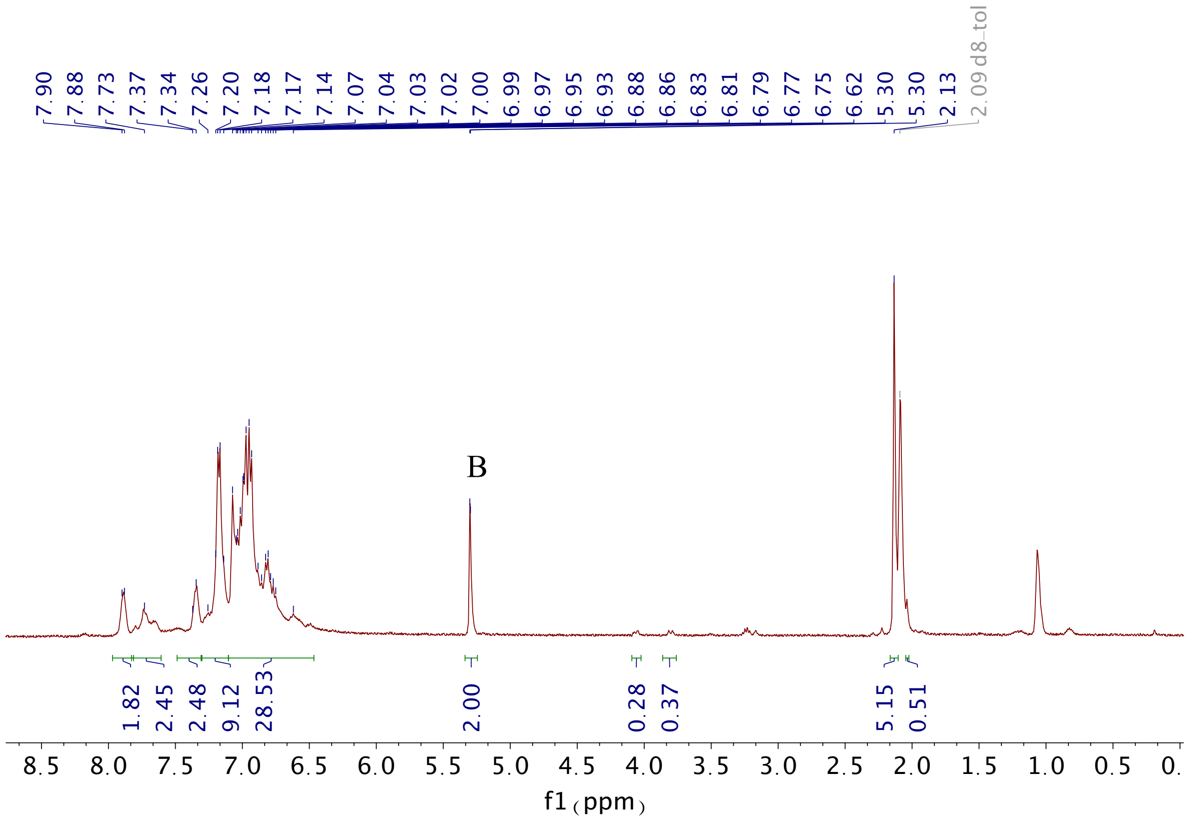

Figure S13. ^{1}H NMR Nickelacyclobutane with d^3-MeCN and d^8-tol at 25ºC after 16h. Peak at 5.4 ppm correspond to 1,1-di(p-tolyl)ethylene B.

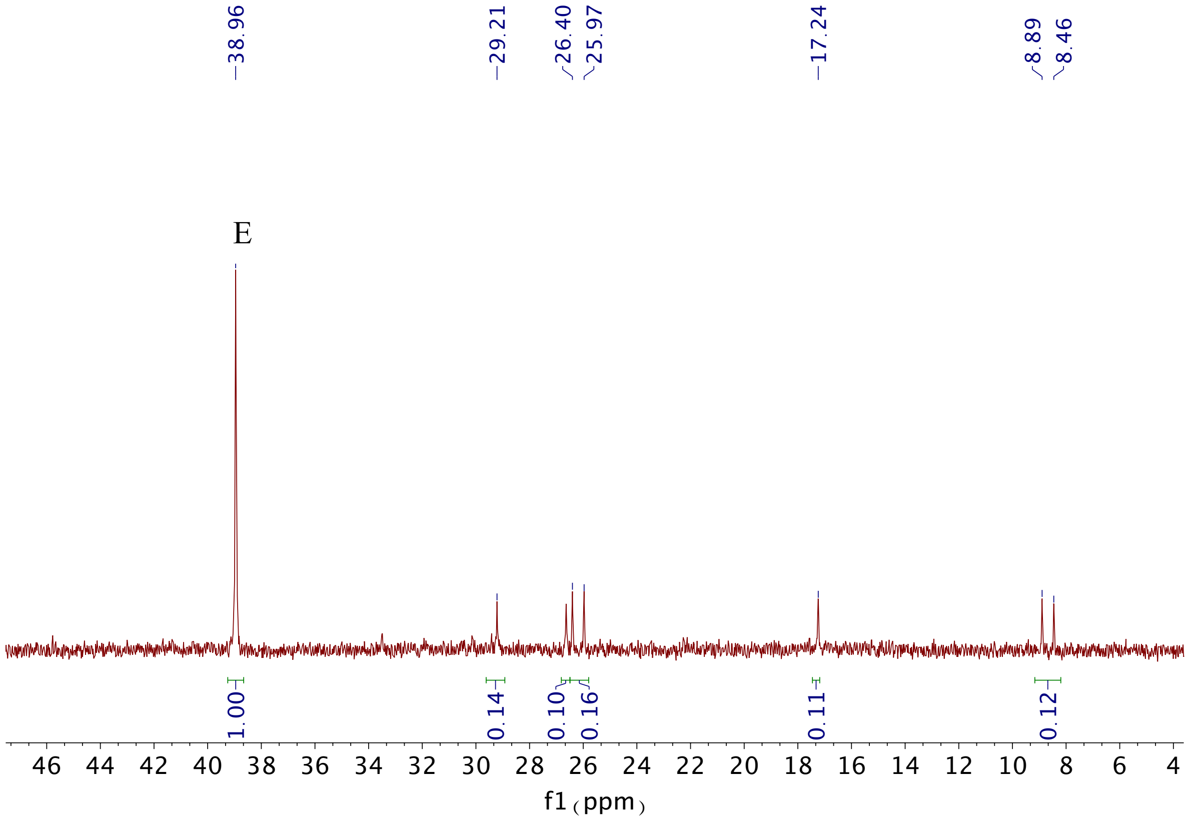

Figure S14. ^{31}P NMR of converted nickelacyclobutane with d^3-MeCN and d^8-tol at 25ºC after 16h. Peak at 38.9 ppm corresponds to (^{Ph}PCDP^{Ph})Ni(CD$_2$CN).

In order to corroborate that the methylene group from 1,1-di(p-tolyl)ethylene originates from the pincer ligand, the same experiment was repeated with a deuterated nickelacyclobutane **4b**. After 16 h, ^{1}H NMR (Figure S15) depicts at ∂(ppm): 2.12 the peak corresponding to the two methyl groups of 1,1-di(p-tolyl)ethylene. The methylene peak is not present at ∂(ppm): 5.41, being deuterated. Additionally, d^2-1,1-di(p-tolyl)ethylene was identified by CG-MS ($[M]^+$= 210). ^{31}P NMR showed conversion to (^{Ph}PCDP^{Ph})Ni(CD$_2$CN) (Figure S16).

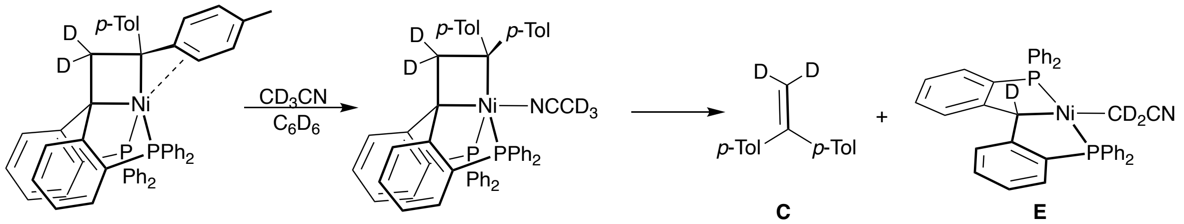

C **E**

Scheme S2. Formation and reactivity of 4b-CD$_3$CN.

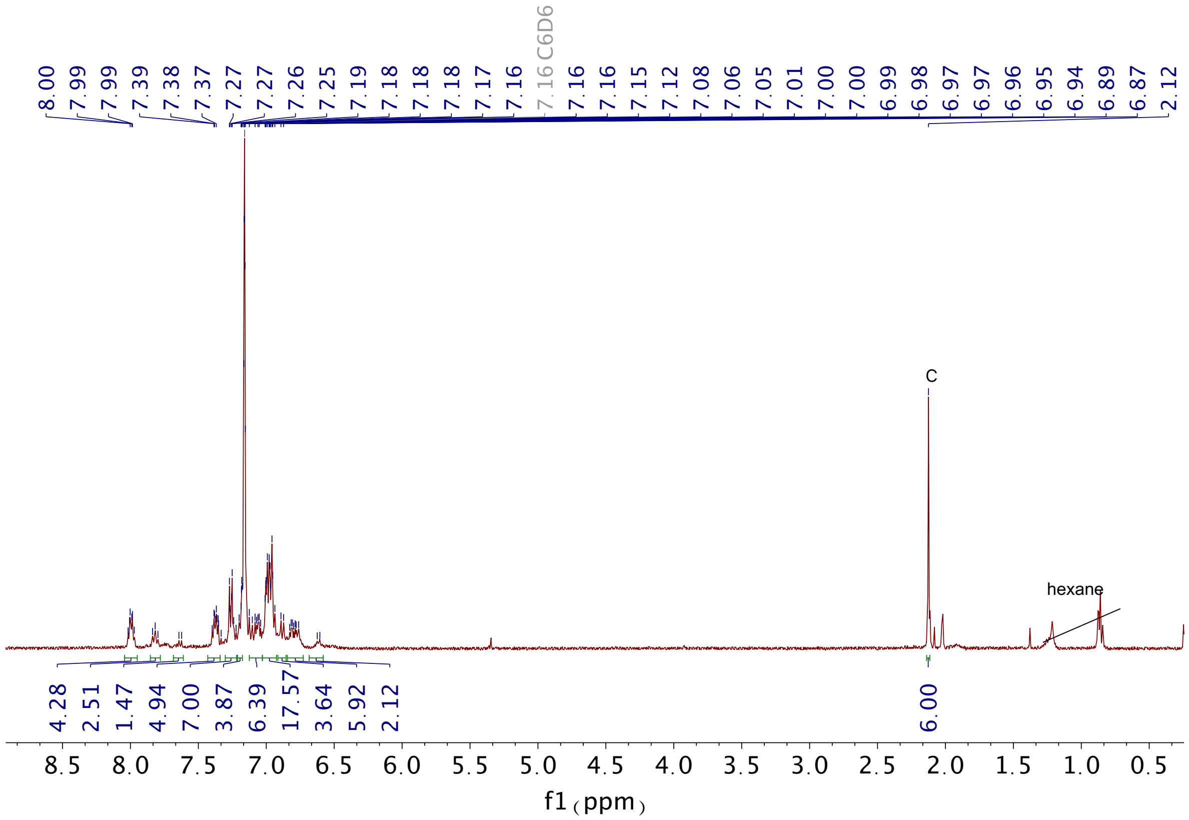

Figure S15. ^{1}H NMR d^{2}-Nickelacyclobutane **4b** with d^{3}-MeCN and d^{8}-tol at 25ºC after 16h. Peak at 2.12 ppm correspond to 1,1-di(p-tolyl)ethylene C.

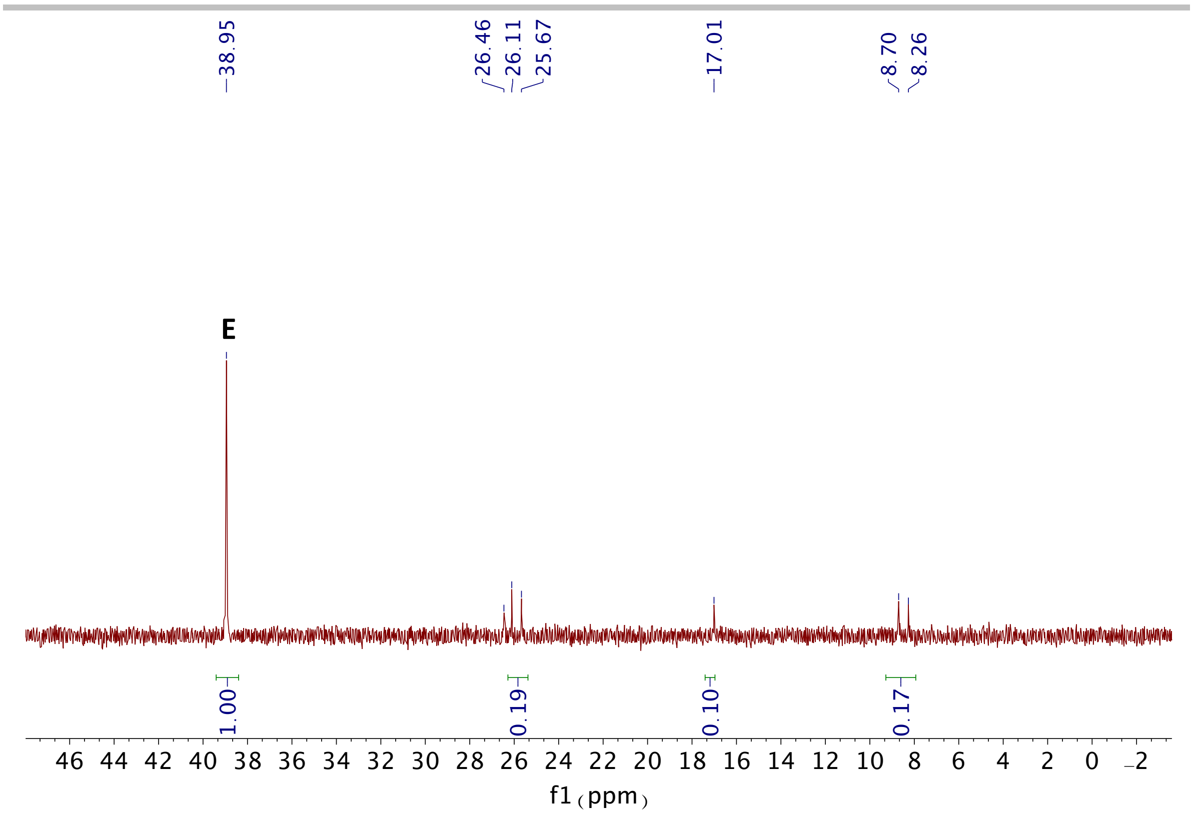

Figure S16. ^{31}P NMR spectrum of converted d^2-Nickelacyclobutane **4b** with d^3-MeCN and d^8-tol at 25ºC after 16h. The peak at 38.9 ppm corresponds to (^{Ph}PCDP^{Ph})Ni(CD_2CN).

An additional experiment was performed to corroborate the selective conversion of **4-MeCN** to the cycloreversion products. Nickelacyclobutane **4** was carefully dissolved in d^3-MeCN and a small amount of dioxane (approximately 2 µL) was added as internal standard. An ^1H NMR spectrum recorded after 5 min in solution (figure S17) revealed that slightly more than 50% conversion of the intermediate to the cycloreversion products has already taken place. Setting the dioxane integral to 1, the peak of the methylene protons from the intermediate displayed an integral of 0.35 at ∂(ppm): 4.12 and 1,1-di(p-tolyl)ethylene methylene peak had an integral of 0.43 at ∂(ppm): 5.39. After 20 min (figure S18), the intermediate was fully consumed and the 1,1-di(p-tolyl)ethylene methylene peak displayed an integral of 0.82, which compares well with the total integral of 0.78 observed in the previous measurement. This supports a selective conversion of the intermediate to cycloreversion products in acetonitrile.

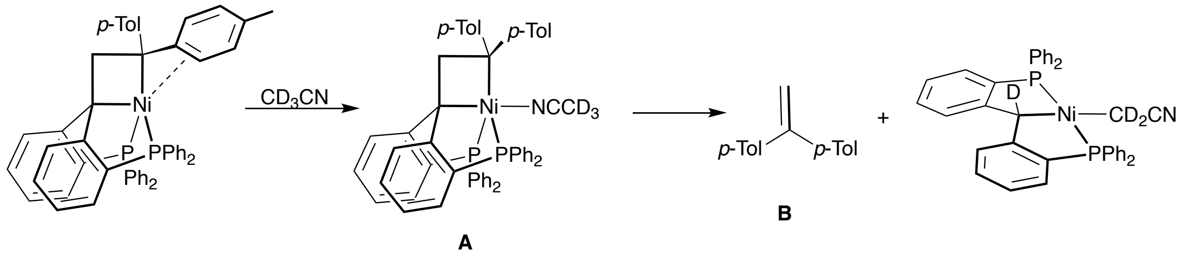

Scheme S3. Formation and reactivity of 4-CD_3CN.

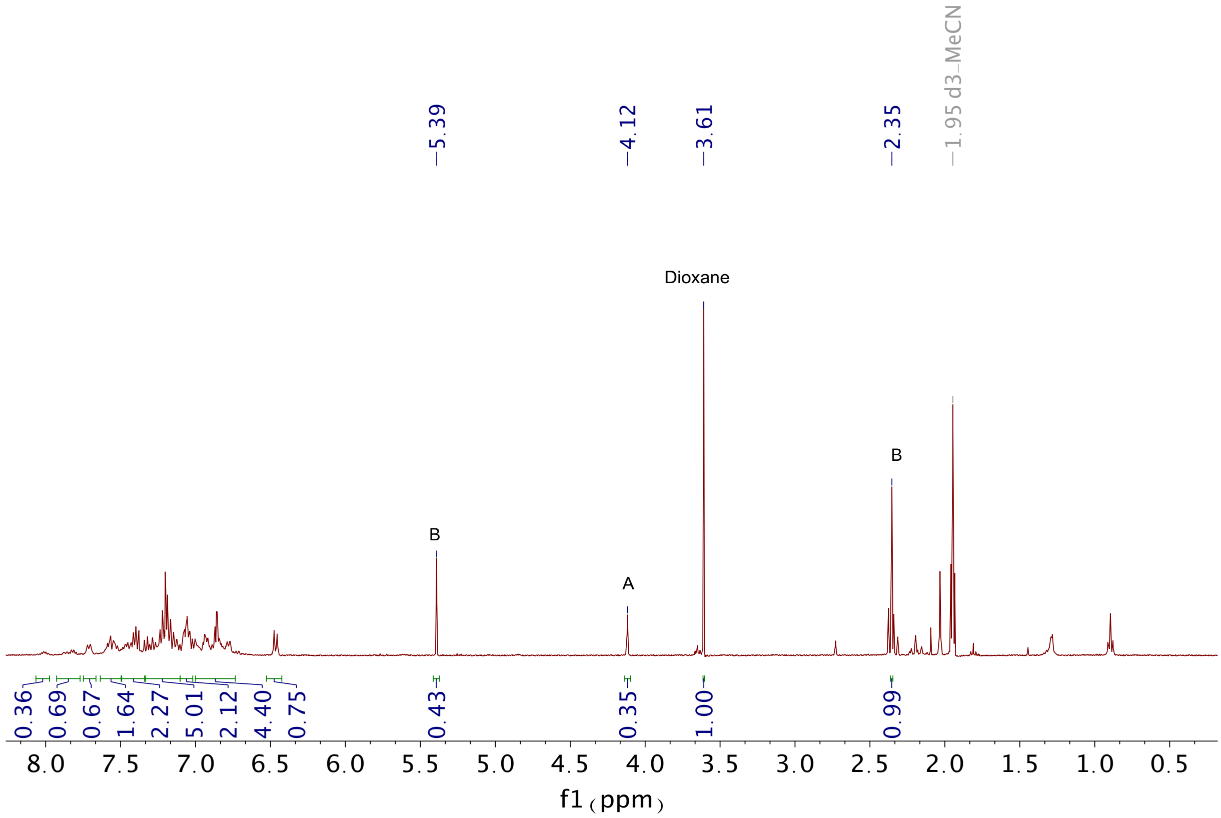

Figure S17. Solution of nickelacyclobutane **4** in d^3-MeCN and dioxane, time= 5 min.

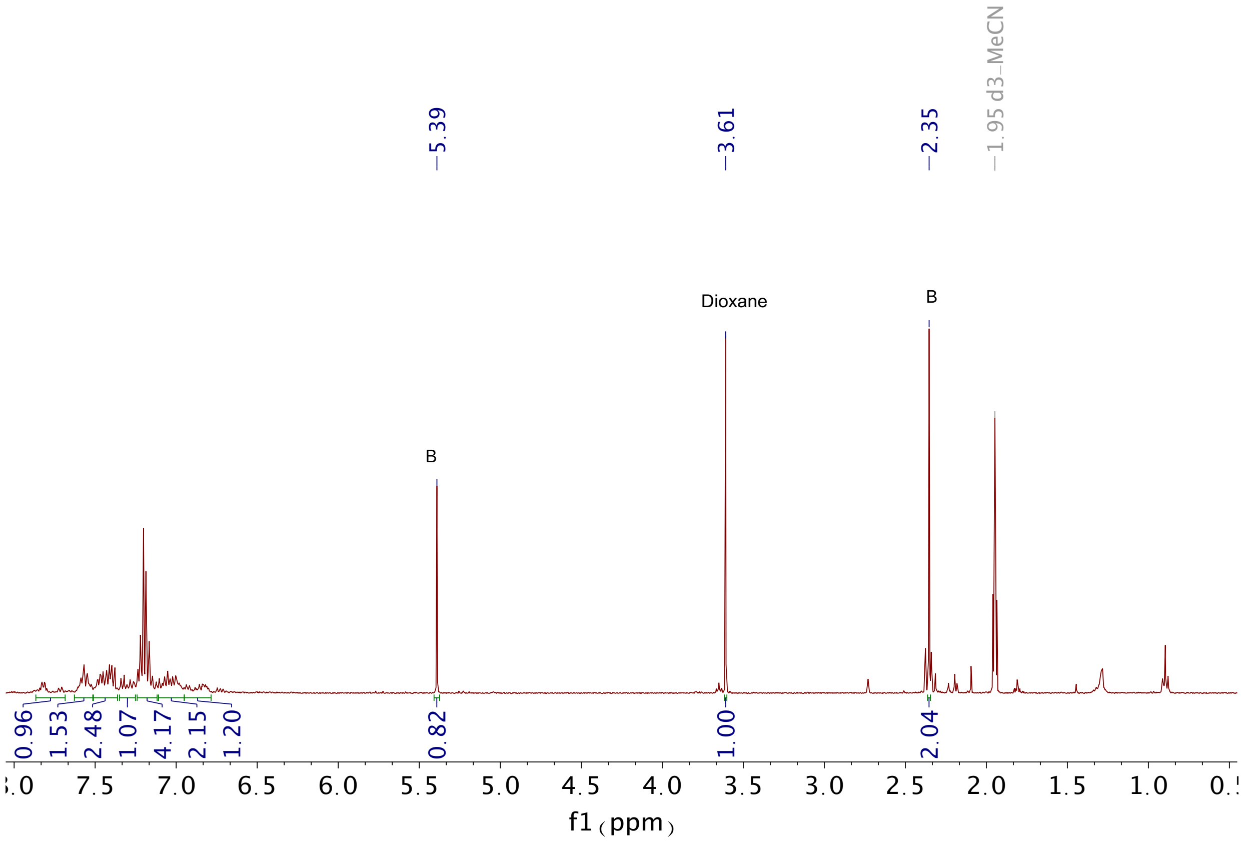

Figure S18. Solution of nickelacyclobutane **4** in d^3-MeCN and dioxane, time= 20 min.

2.5 Bimolecular experiment in the formation of [(^{Ph}bppe^{H,CHptol2})Ni]$_2$N$_2$ (7)

The bimolecular experiment started with a mixture of Nickelacyclobutane and d^2-Nickelacyclobutane in proportion 1:1 in a benzene solution. Deuterated solvents were avoided to discard exchange with the solvent. In Figure S19 is presented the initial ^{1}H NMR spectrum, where the methylene protons are located at ∂(ppm) 4.37 and the methyl groups at 1.74 following the expected proportion 2:12. ^{31}P NMR in Figure S20 exhibits the reported broad signal.
After 16h, an ^{1}H NMR spectrum (Figure S21) showed the reactants had not been completely consumed and maintained the initial proportion of 2:12 of the methylene and methyl groups. Interestingly, in the case of the product, the methine protons located at ∂(ppm): 4.07 and 4.69 did not present an equal proportion expected for an intramolecular reaction. The proportion of the methine protons were 2:0.6 (77 % : 23 %) respectively, suggesting a complex bimolecular process in the formation of [(^{Ph}bppe^{H,CHptol2})Ni]$_2$N$_2$. ^{31}P NMR in Figure S22 presented the products as two doublets on top of each other, giving the appearance of two quadruplets with small J coupling.

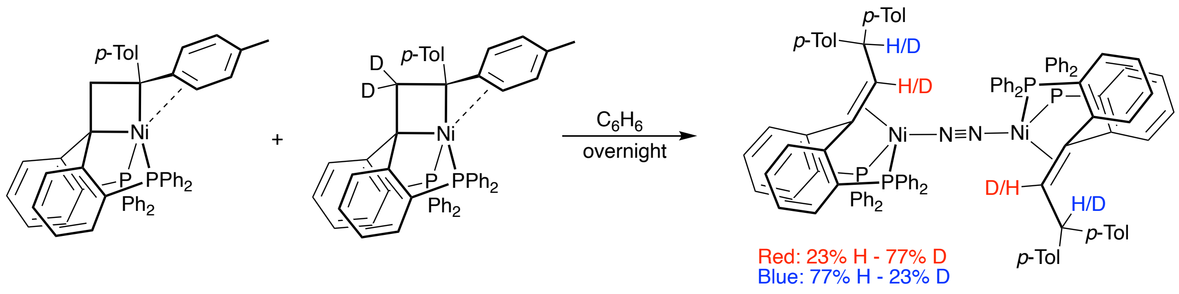

Scheme S4. Bimolecular experiment using nickelacyclobutane 4 and 4b (deuterated).

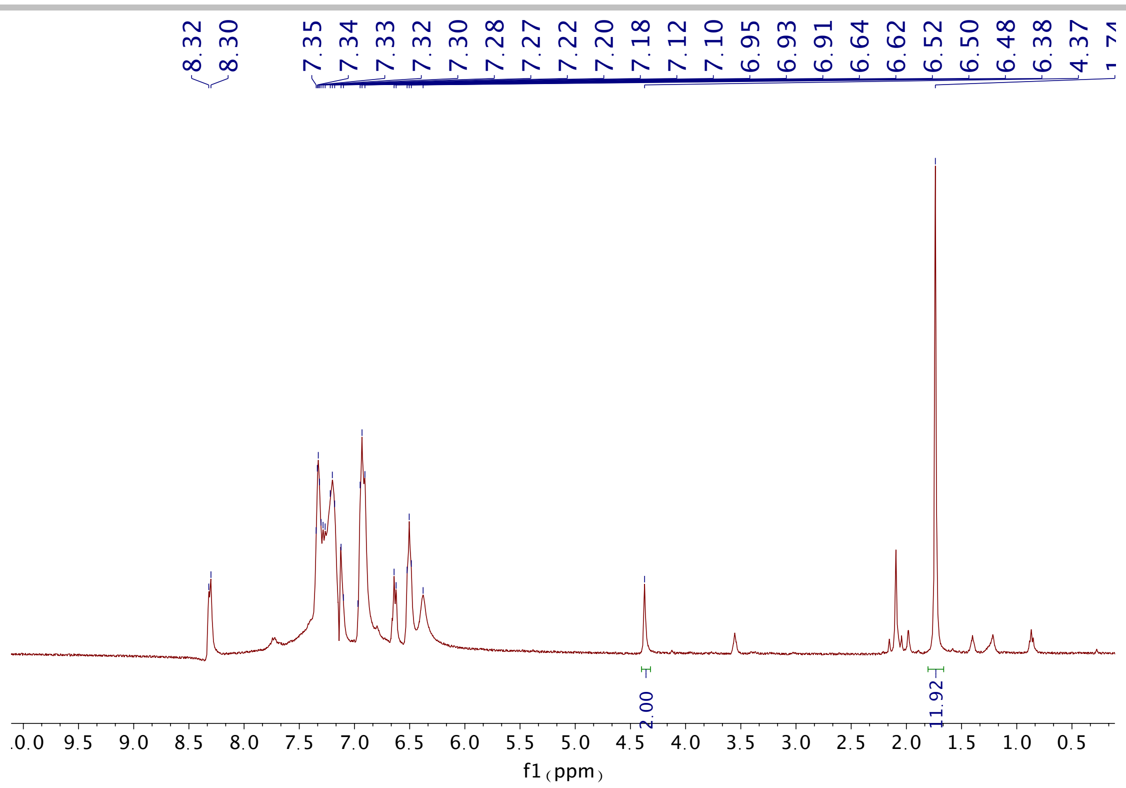

Figure S19. ^{1}H NMR of a 1:1 mixture of Nickelacyclobutane (**4**) and d^{2}-Nickelacyclobutane (**4b**) in benzene. Solvent suppression is applied.

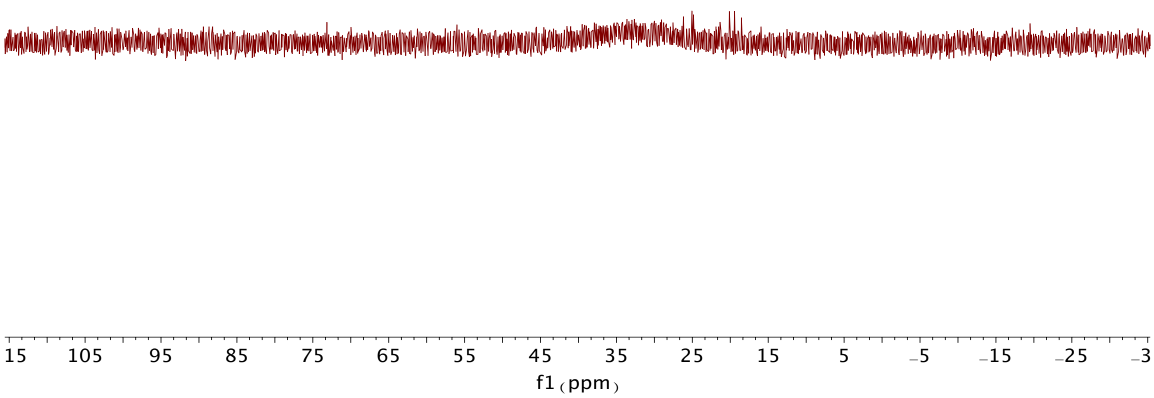

Figure S20. ^{31}P NMR in benzene of a 1:1 mixture of Nickelacyclobutane (**4**) and d^{2}-Nickelacyclobutane (**4b**).

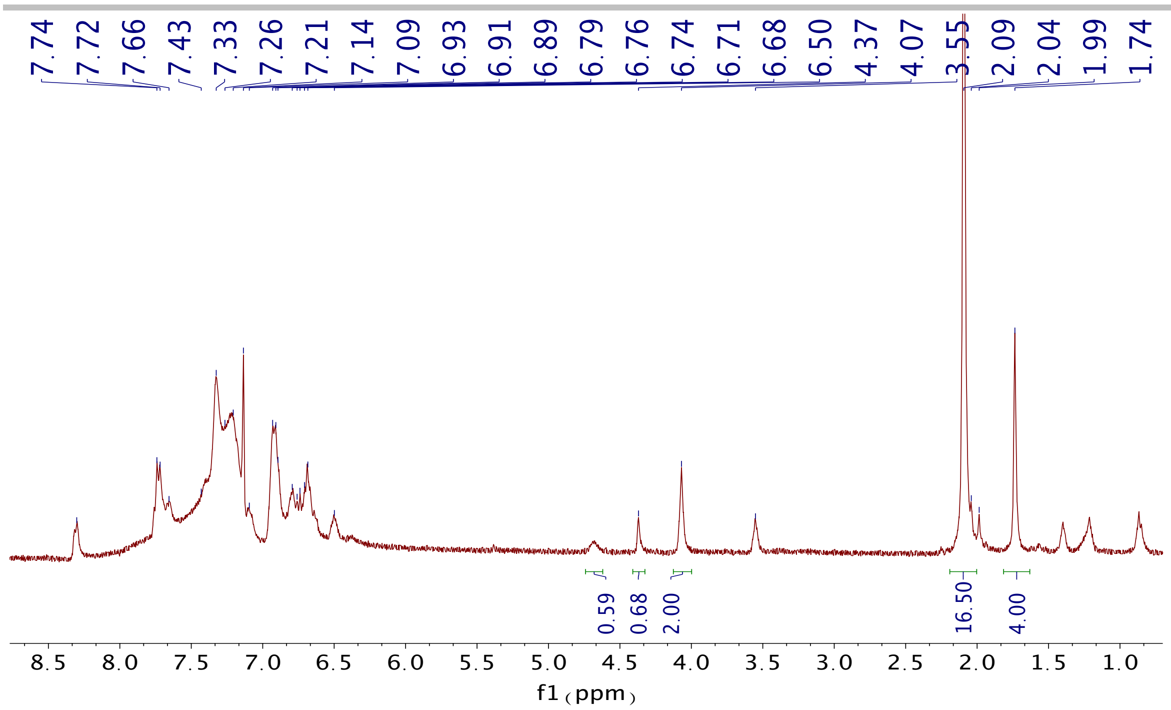

Figure S21. Solvent suppression ^{1}H NMR in benzene of a 1:1 mixture of Nickelacyclobutane (**4**) and d^{2}-Nickelacyclobutane (**4b**) after 16h at room temperature. Peaks at 4.37 and 1.74 ppm correspond not consumed reactant.

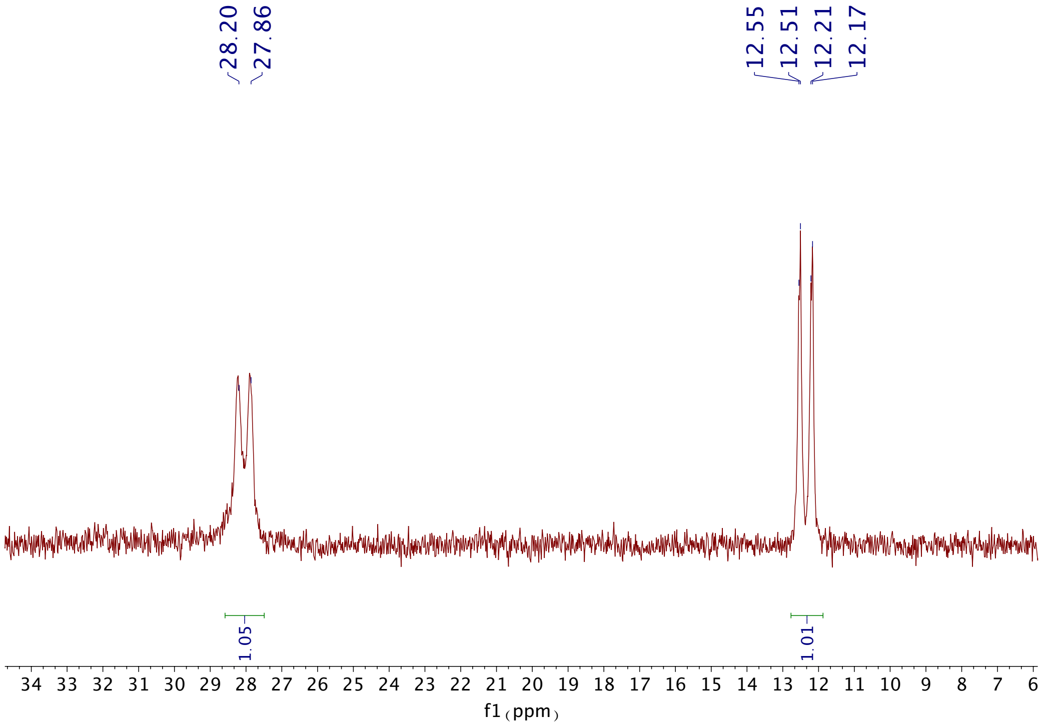

Figure S22. ^{31}P NMR in benzene of a 1:1 mixture of Nickelacyclobutane (**4**) and d^{2}-Nickelacyclobutane (**4b**) after 16h at room temperature.

Scrambling of hydrogen/deuterium atoms could be explained by a radical mechanism as described in Scheme S5. In this proposed mechanism, Ni-C(p-Tol)2 bond undergoes homolytic cleavage to yield a Ni(I) species where the C(p-Tol)$_2$ radical attacks a C-D bond of another nickelacyclobutane molecule, resulting in a nickelacyclobutyl radical on Cβ. This organic radical could abstract a hydrogen atom from the Ni(I) complex yielding a Ni(I) organic radical on Cβ that rearrange into the Ni(0)-olefin product. This process generates a partially deuterated nickelacyclobutane that can enter the reaction chain again. This allows for intermolecular redistribution of H and D.

In addition, the observation that the olefinic proton is predominately deuterated in the product can be explained by the different strengths of C-H and C-D bonds. The first H-atom abstraction step is likely to be faster for the weaker C-H bonds than for C-D bonds (primary kinetic isotope effect), resulting in a lower deuteration of the tertiary position and, consequently, a higher deuteration of the olefinic position.

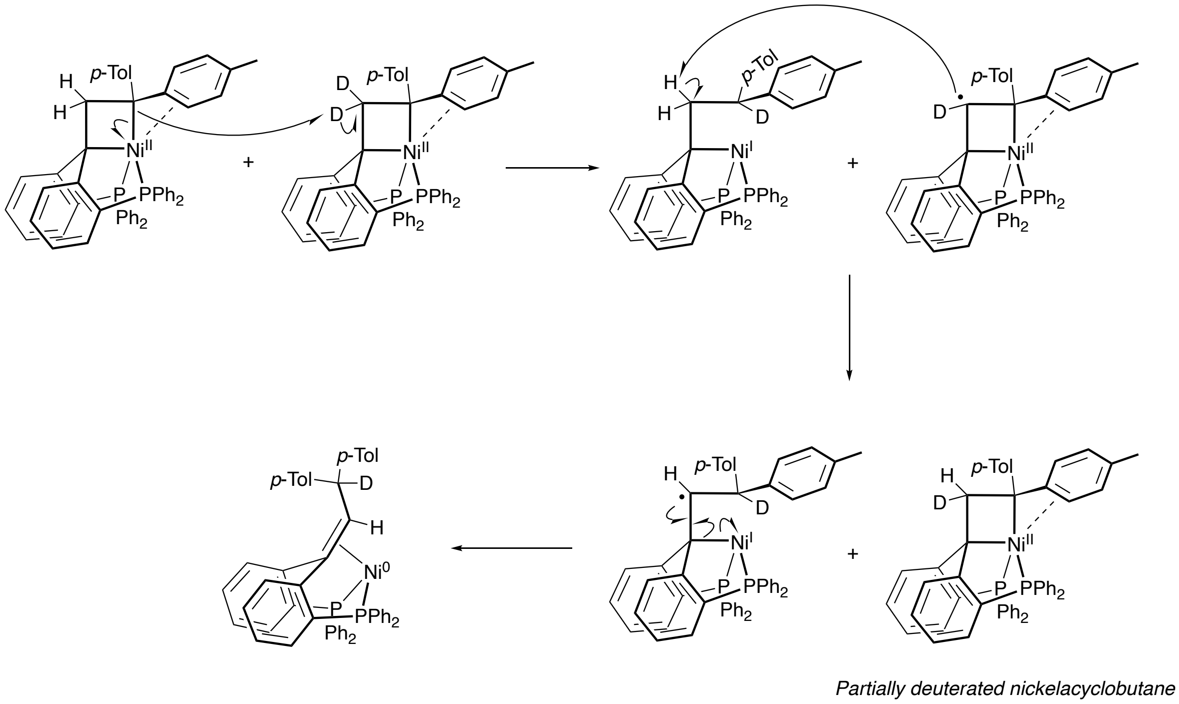

Scheme S5. Proposed mechanism for the bimolecular reactivity of nickelacyclobutane 4 and 4b (deuterated).

3. Spectra of new compound

^{Ph}bppe^{H,H} Ligand (1)

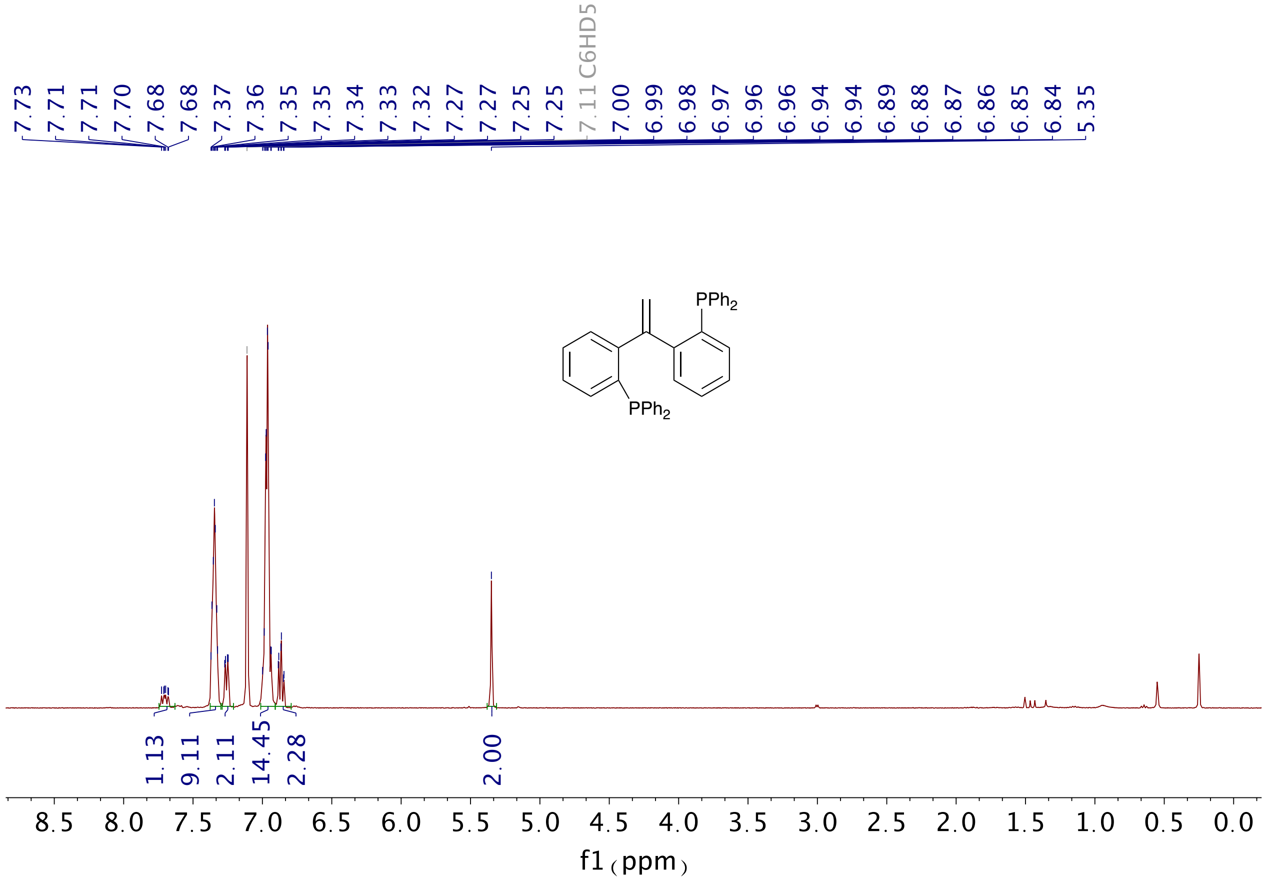

Figure S23. ^{1}H NMR, C_6D_6, 25 ºC, compound **1**.

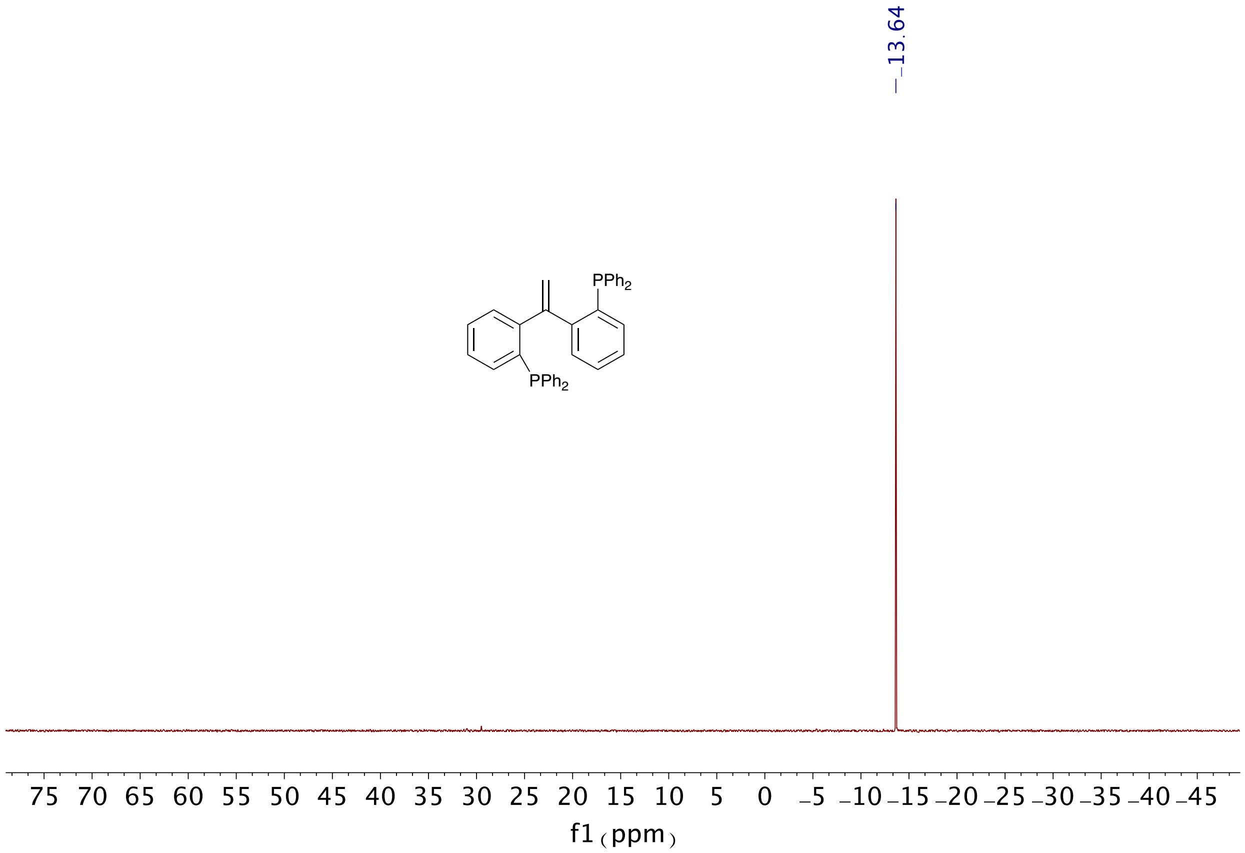

Figure S24. $^{31}P\{^{1}H\}$ NMR, C_6D_6, 25 ºC, compound **1**.

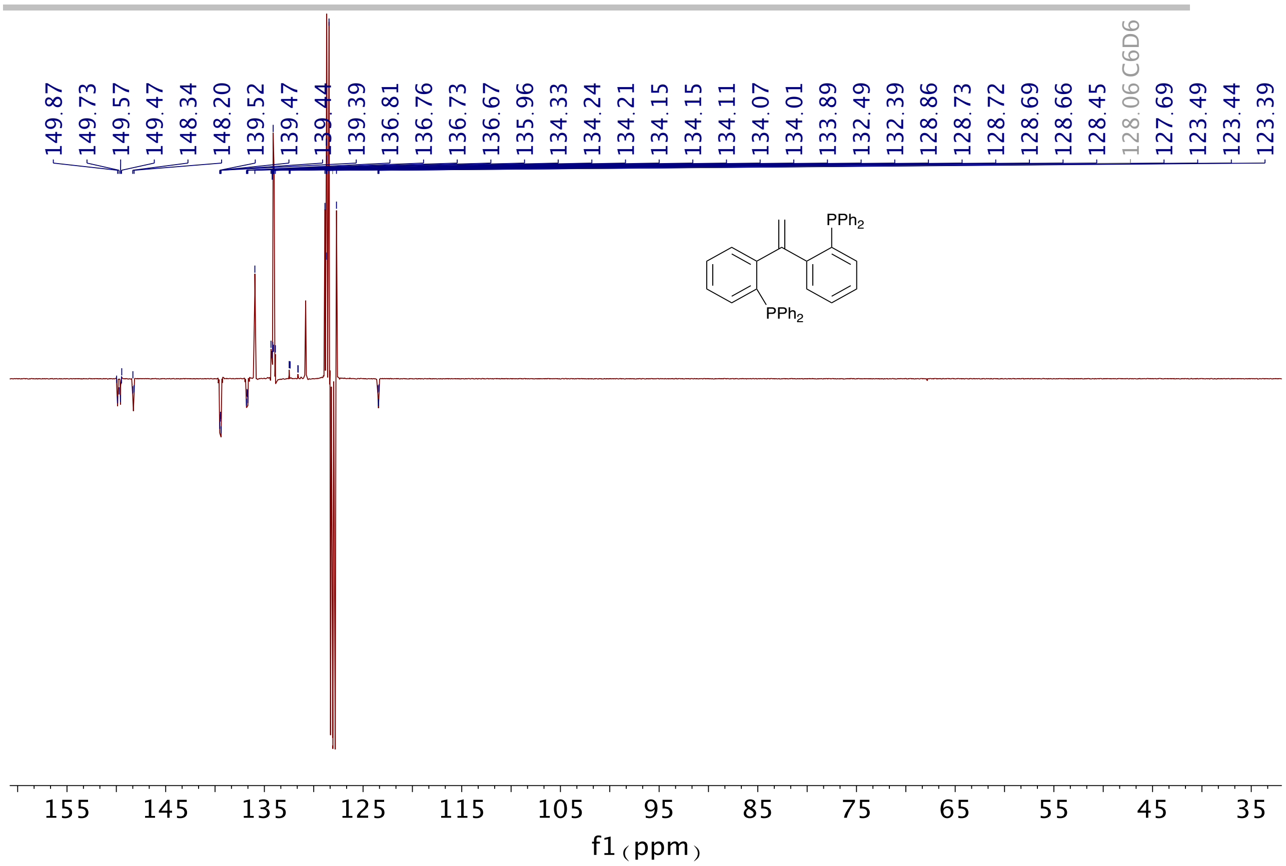

Figure S25. APT, C_6D_6, 25 ºC, compound **1**.

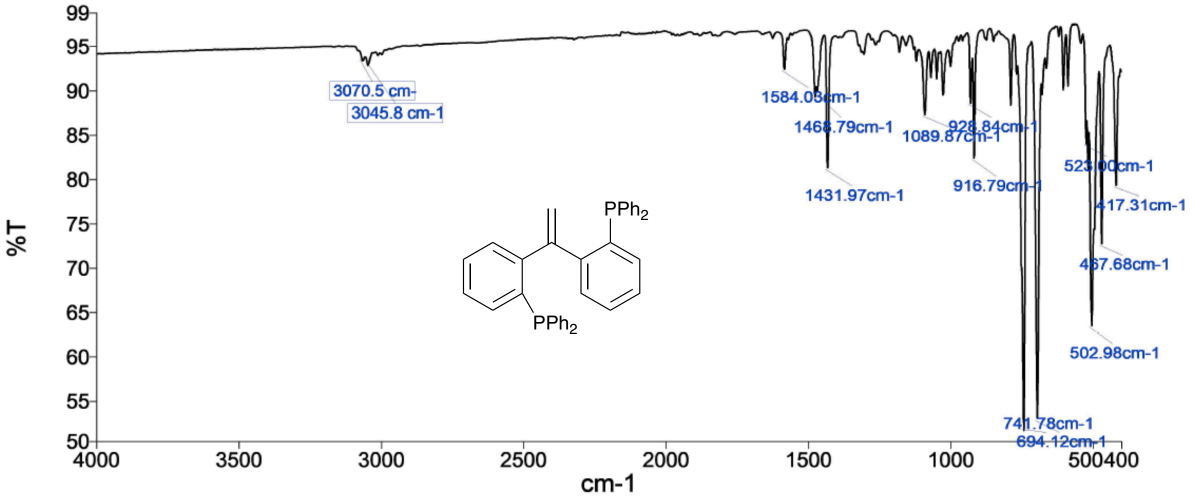

Figure S26. IR compound 1.

^{Ph}bppe^{D,D} Ligand (1b)

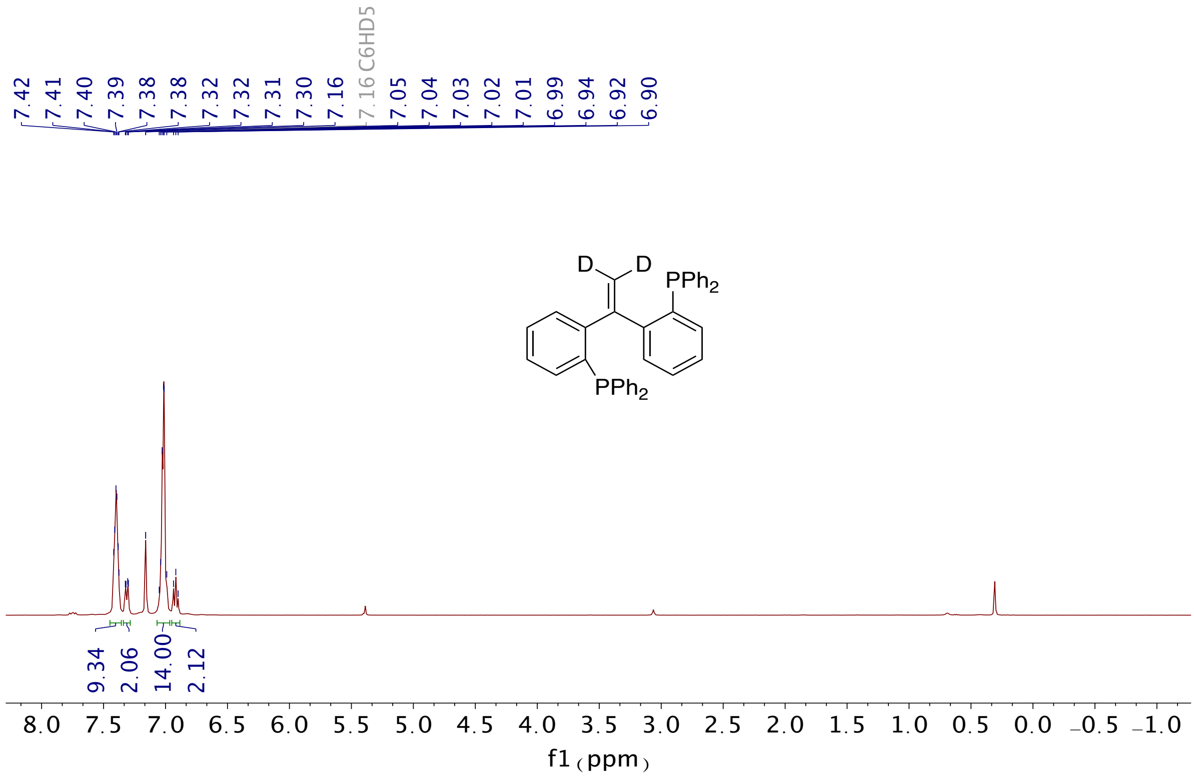

Figure S27. ^{1}H NMR, C_6D_6, 25 ºC, compound **1b**.

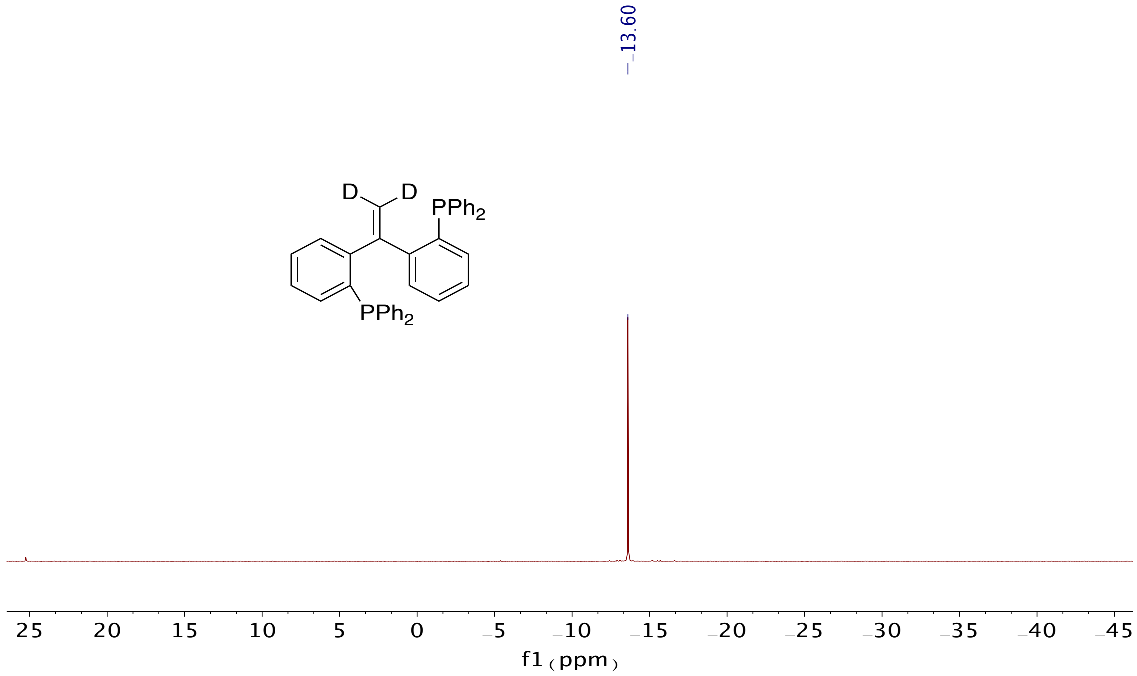

Figure S28. $^{31}P\{^{1}H\}$ NMR, C_6D_6, 25 ºC, compound **1b**

(^{Ph}bppe^{H,H})Ni(CH_2CHPhF) (2)

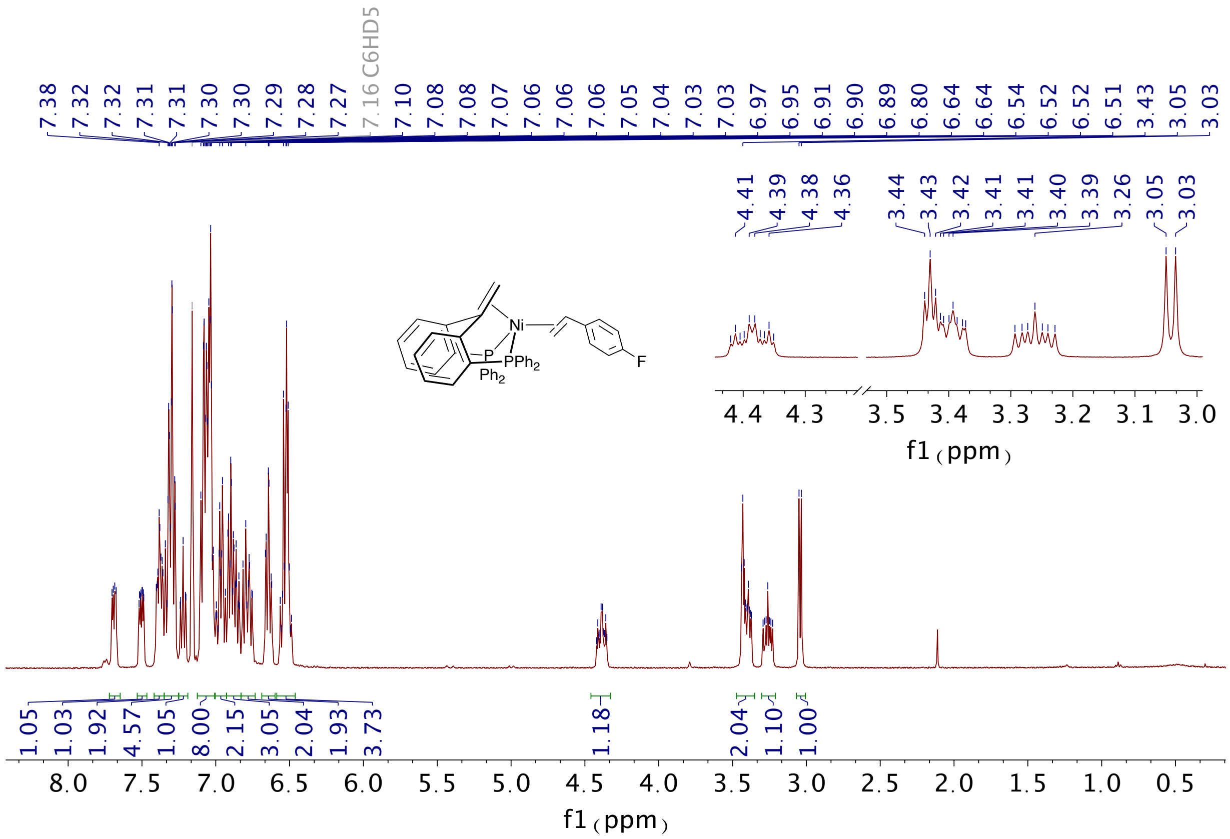

Figure S29. ^{1}H NMR, C_6D_6, 25 ºC, complex **2**.

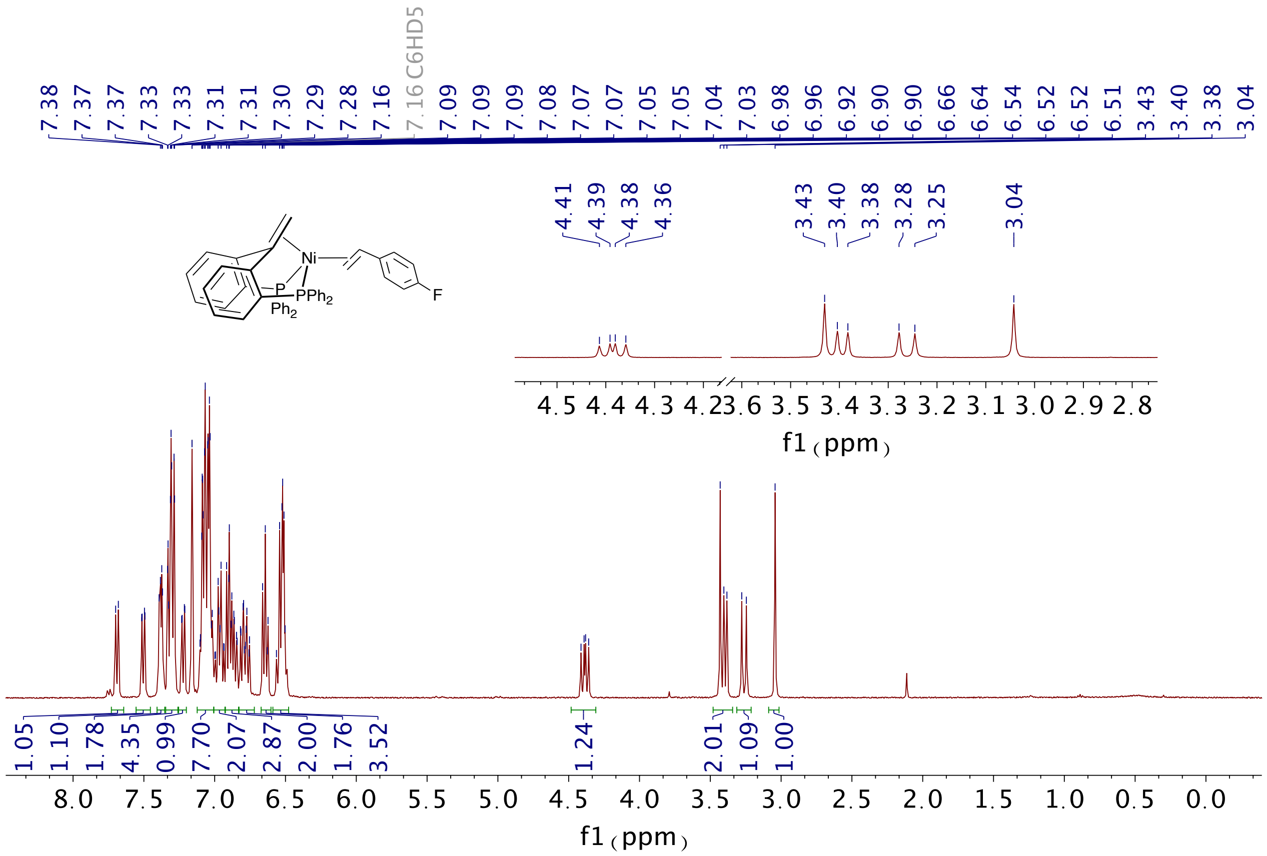

Figure S30. $^{1}H\{^{31}P\}$ NMR, C_6D_6, 25 ºC, complex **2**.

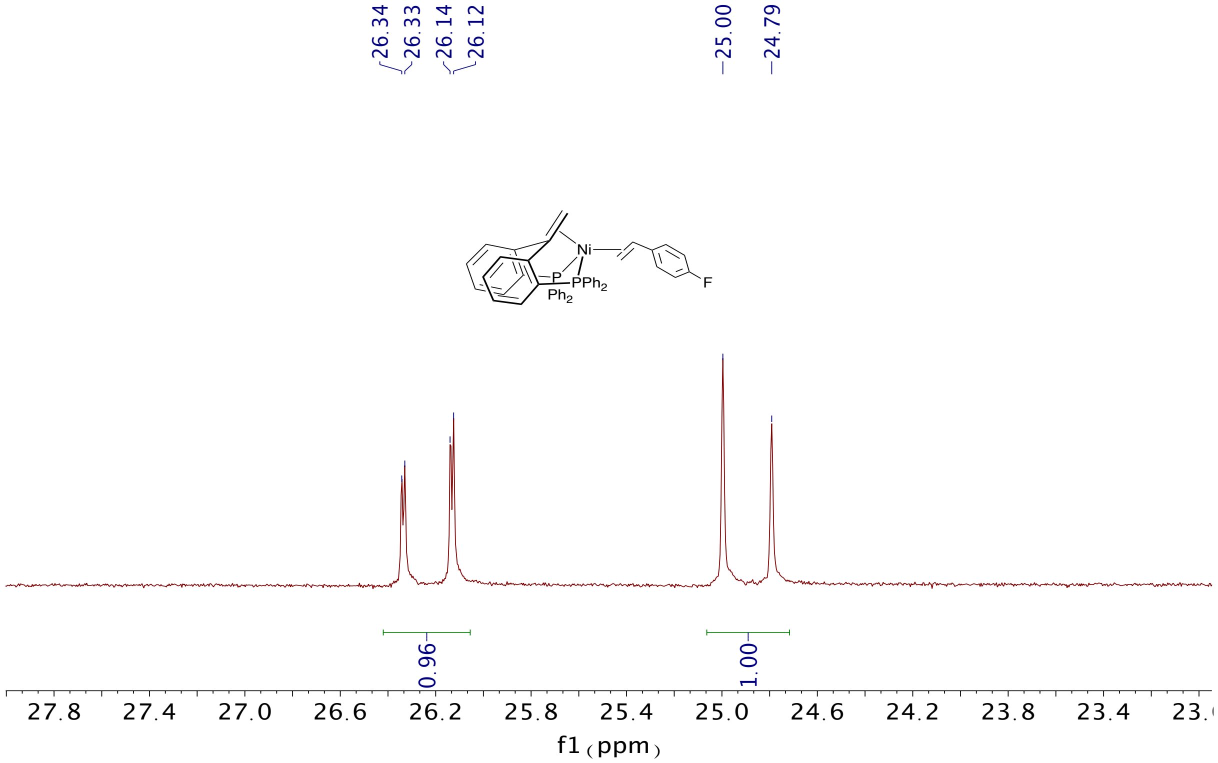

Figure S31. $^{31}P\{^{1}H\}$ NMR, C_6D_6, 25 ºC, complex **2**.

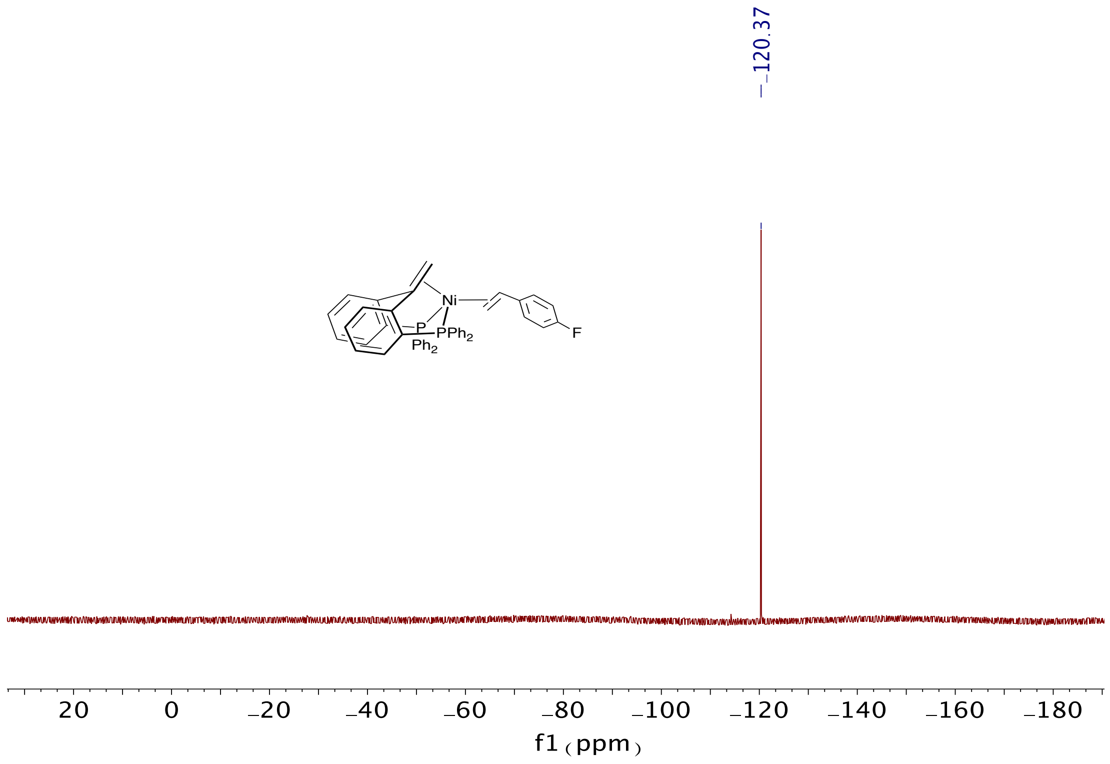

Figure S32. ^{19}F NMR, C_6D_6, 25 ºC, complex **2**.

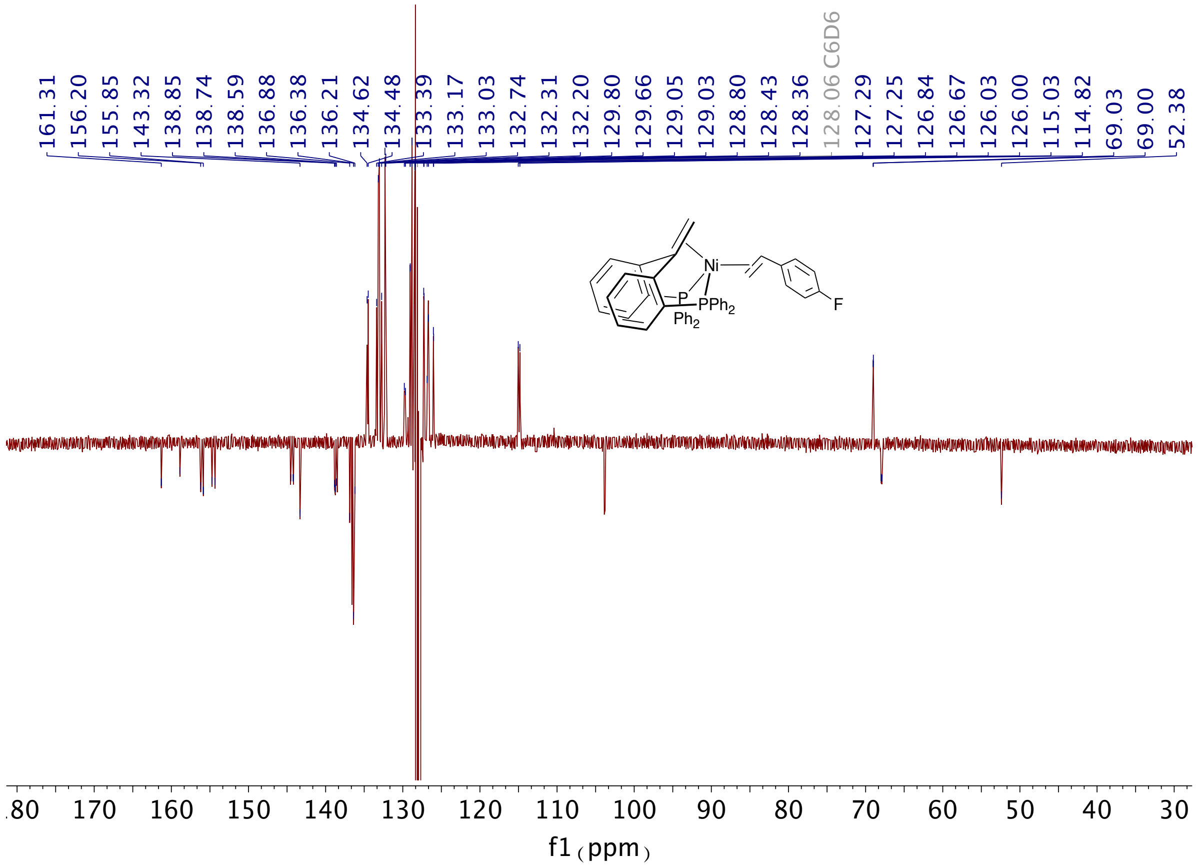

Figure S33. APT, C_6D_6, 25 ºC, complex **2**.

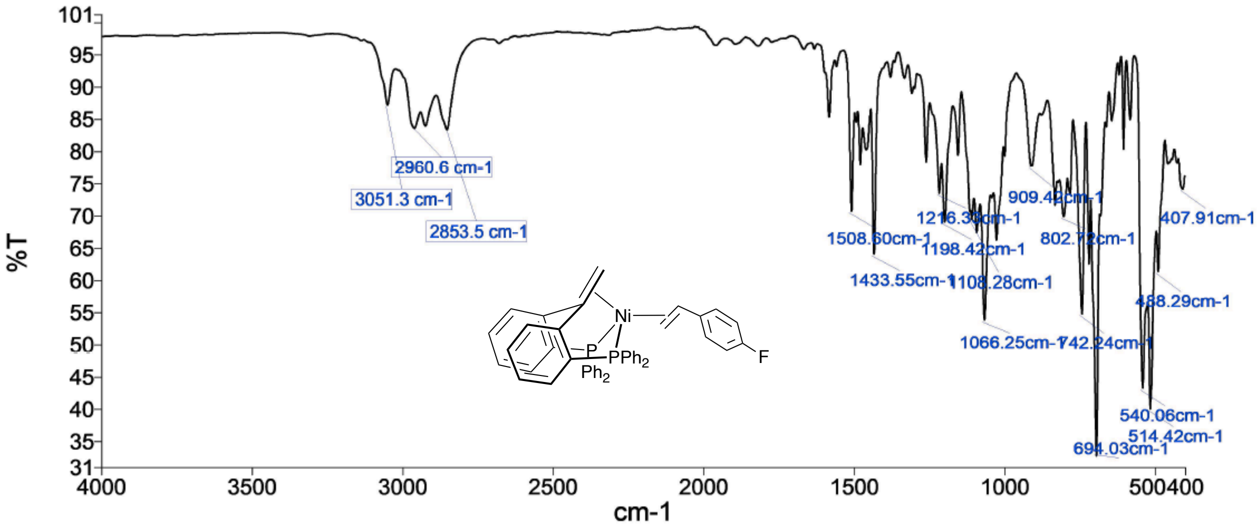

Figure S34. IR complex **2**.

(^{Ph}bppe^{D,D})Ni(CH$_2$CHPhF) (2b)

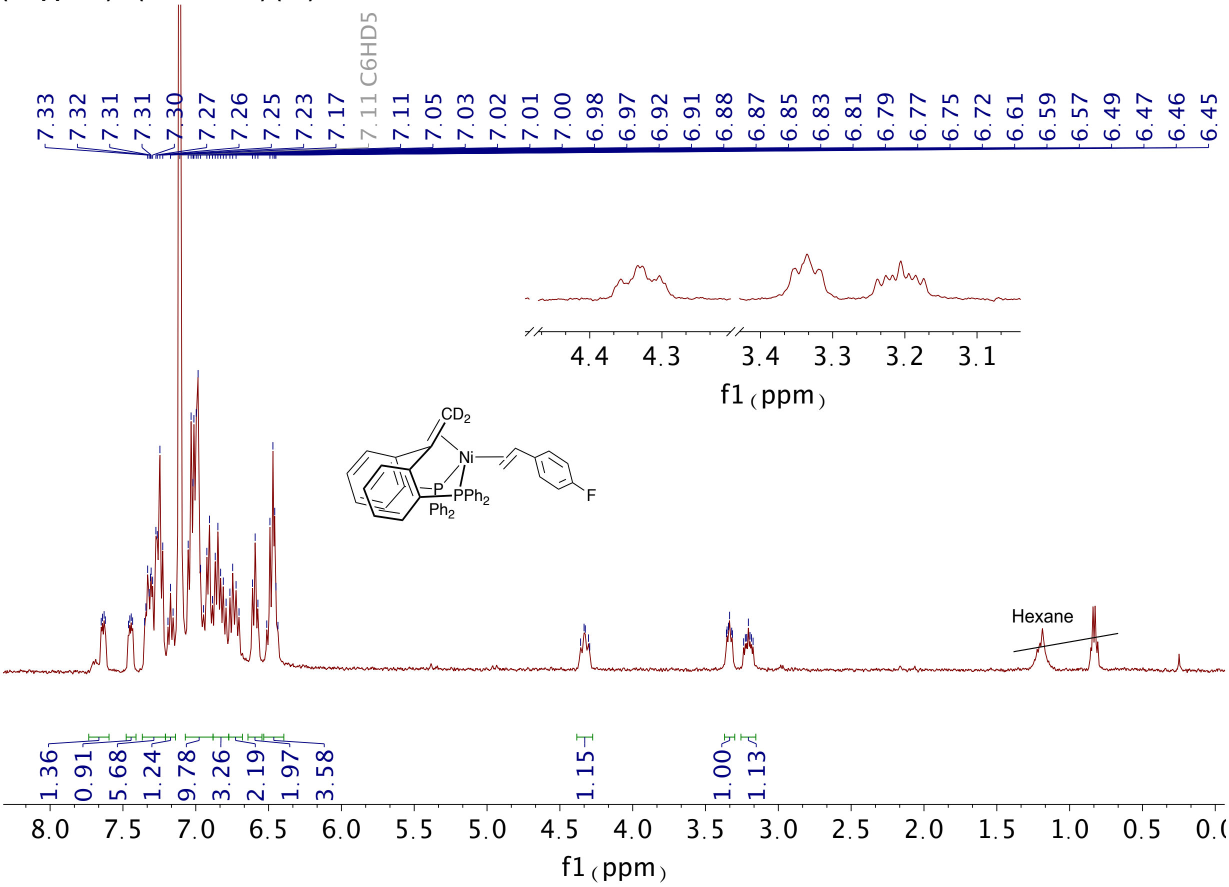

Figure S35. ^{1}H NMR, C_6D_6, 25 ºC, complex **2b**.

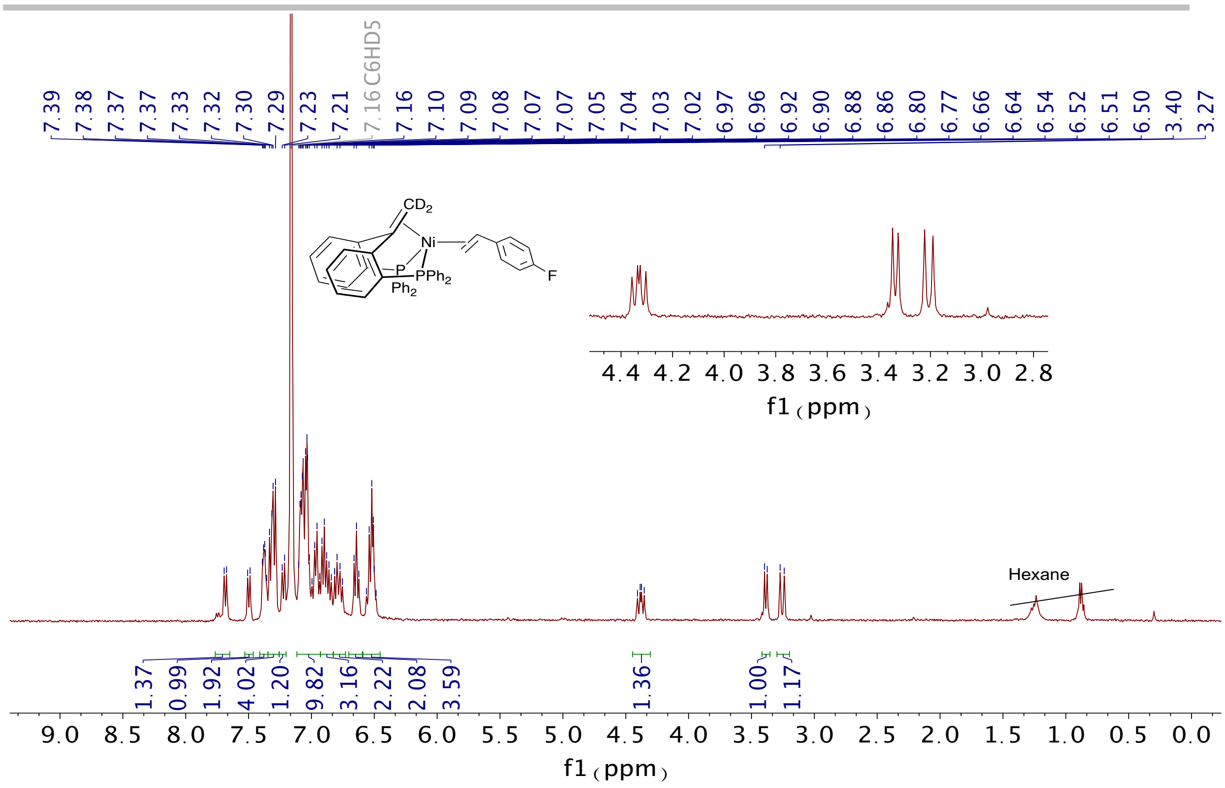

Figure S36. $^{1}H\{^{31}P\}$ NMR, C_6D_6, 25 ºC, complex **2b**.

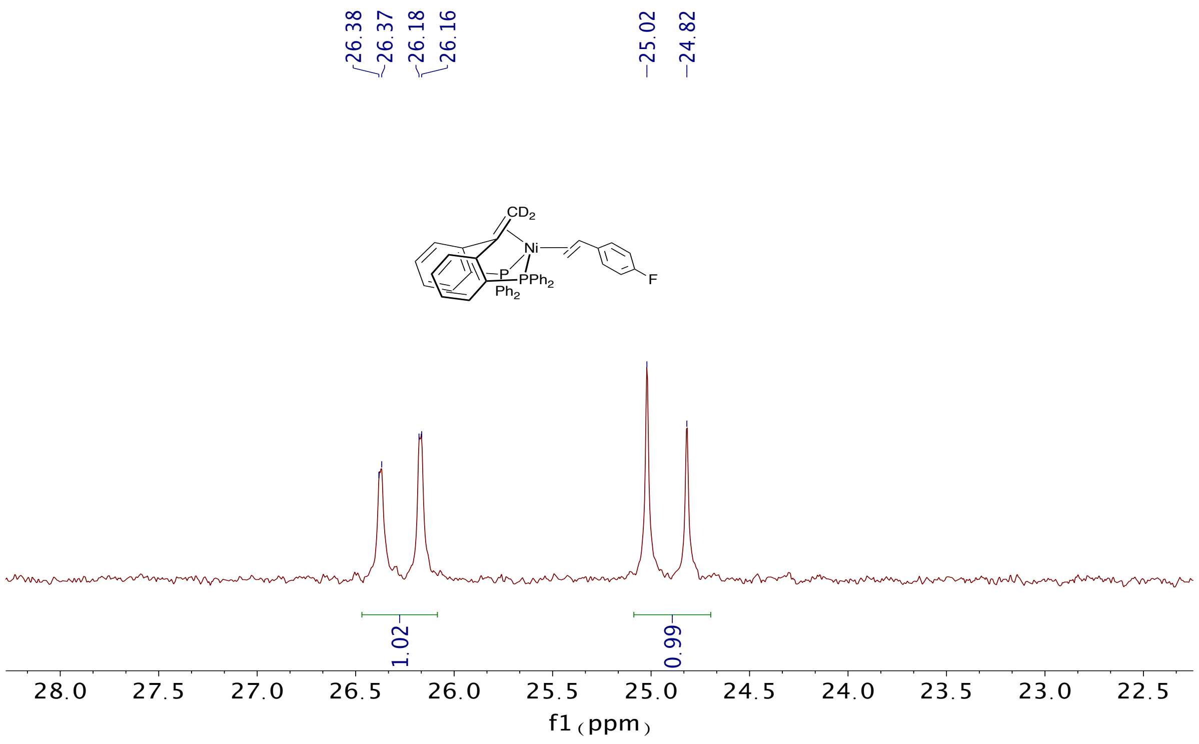

Figure S37. $^{31}P\{^{1}H\}$ NMR, C_6D_6, 25 ºC, complex **2b**.

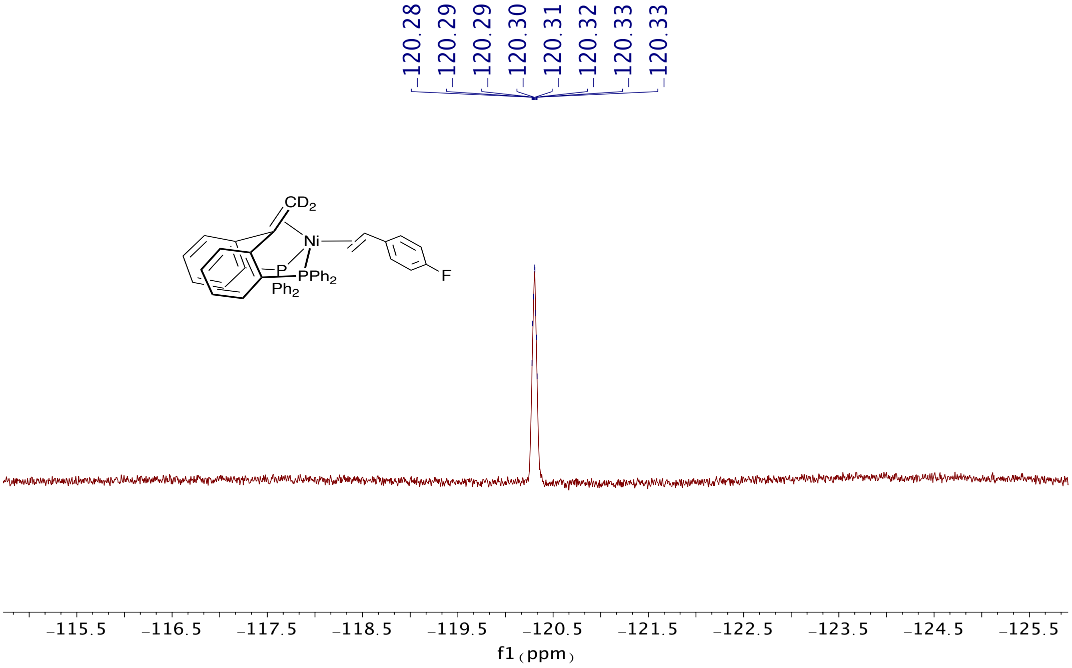

Figure S38. ^{19}F NMR, C_6D_6, 25 ºC, complex **2b**.

(^{Ph}bppe^{H,H})Ni(N_2Cp-tol$_2$) (3)

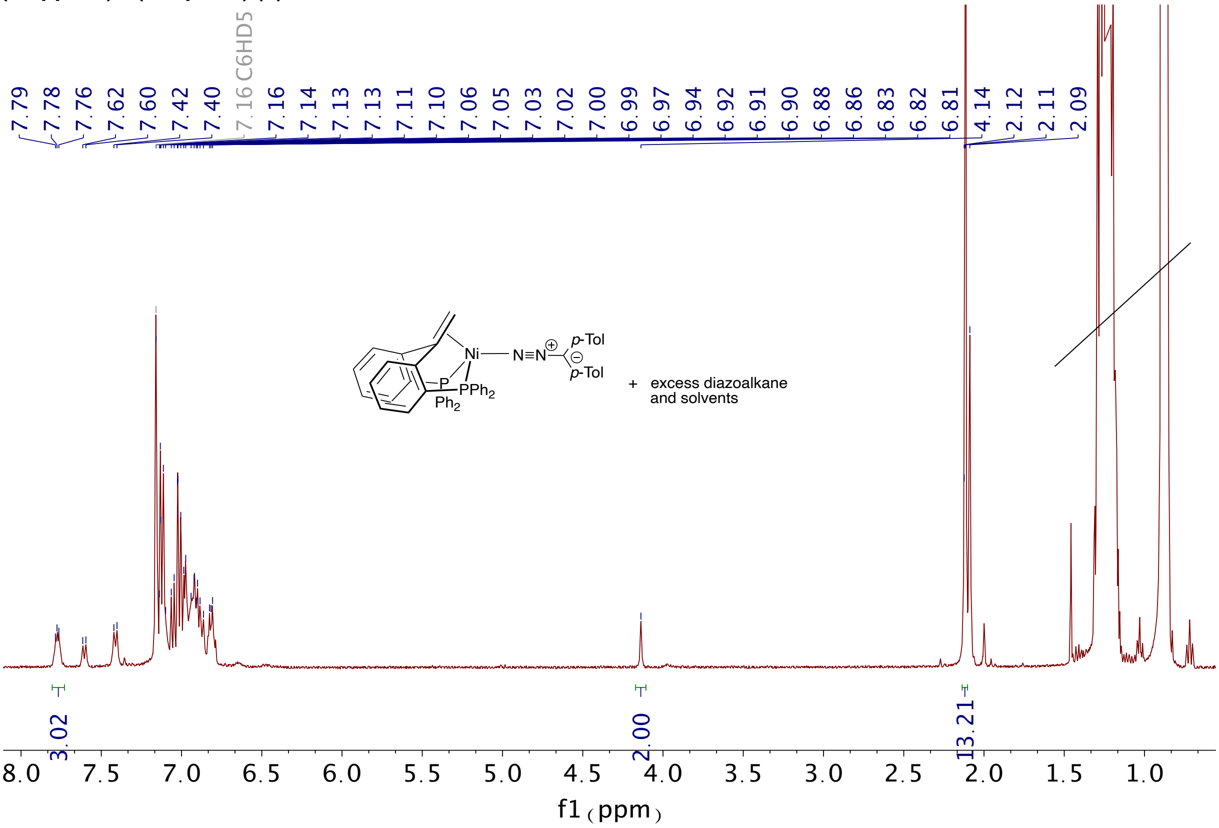

Figure S39. ^{1}H NMR, C_6D_6, 25 ºC, complex **3** non-isolated.

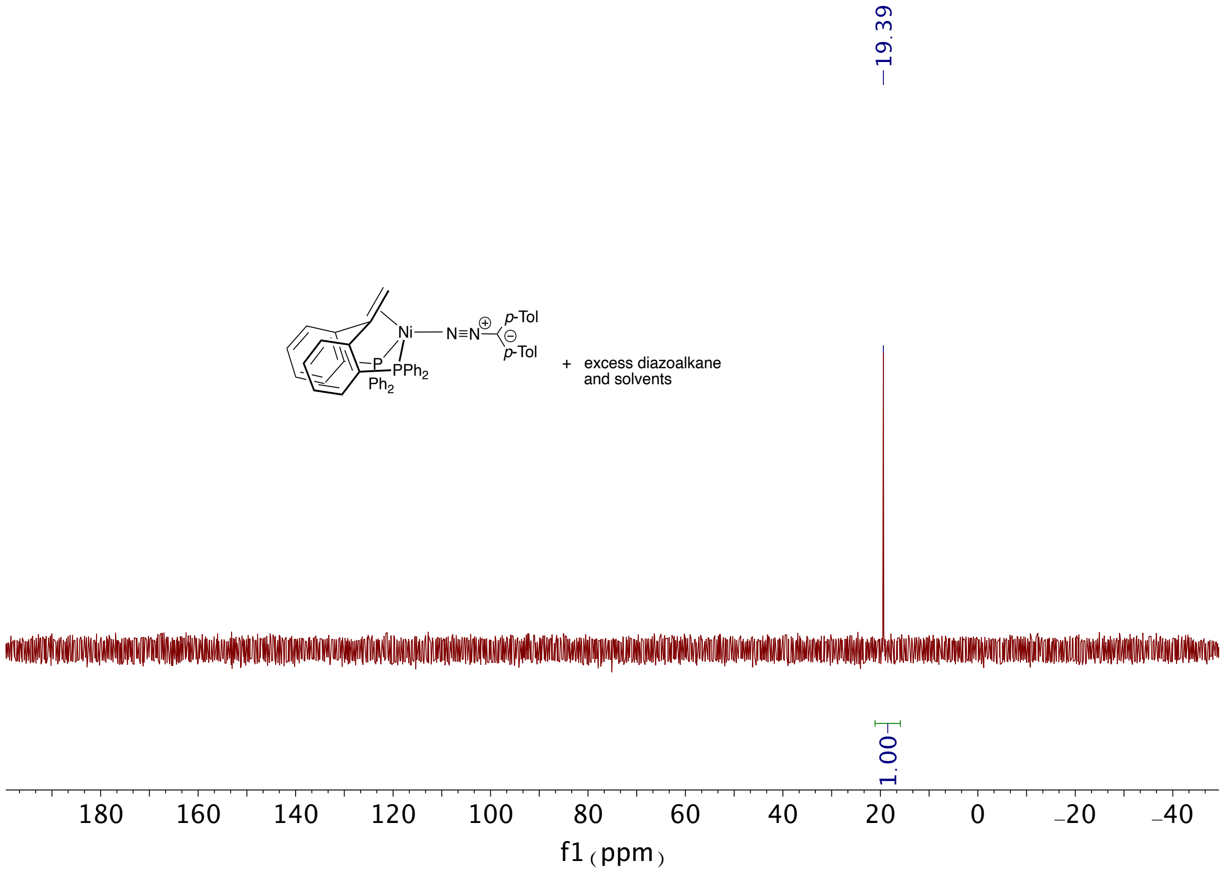

Figure S40. $^{31}P\{^{1}H\}$ NMR, C_6D_6, 25 ºC, complex **3** non-isolated.

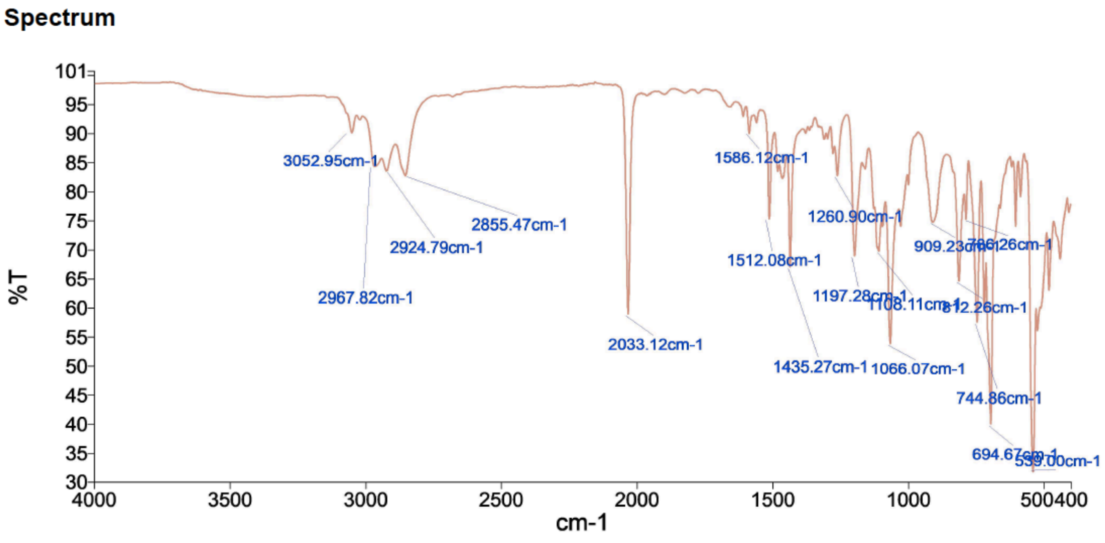

Figure S41. IR mixture of (^{Ph}bppe^{H,H})Ni(N_2Cp-tol$_2$), complex **3**; and Nickelacyclobutane, complex **4**.

Nickelacyclobutane (4)

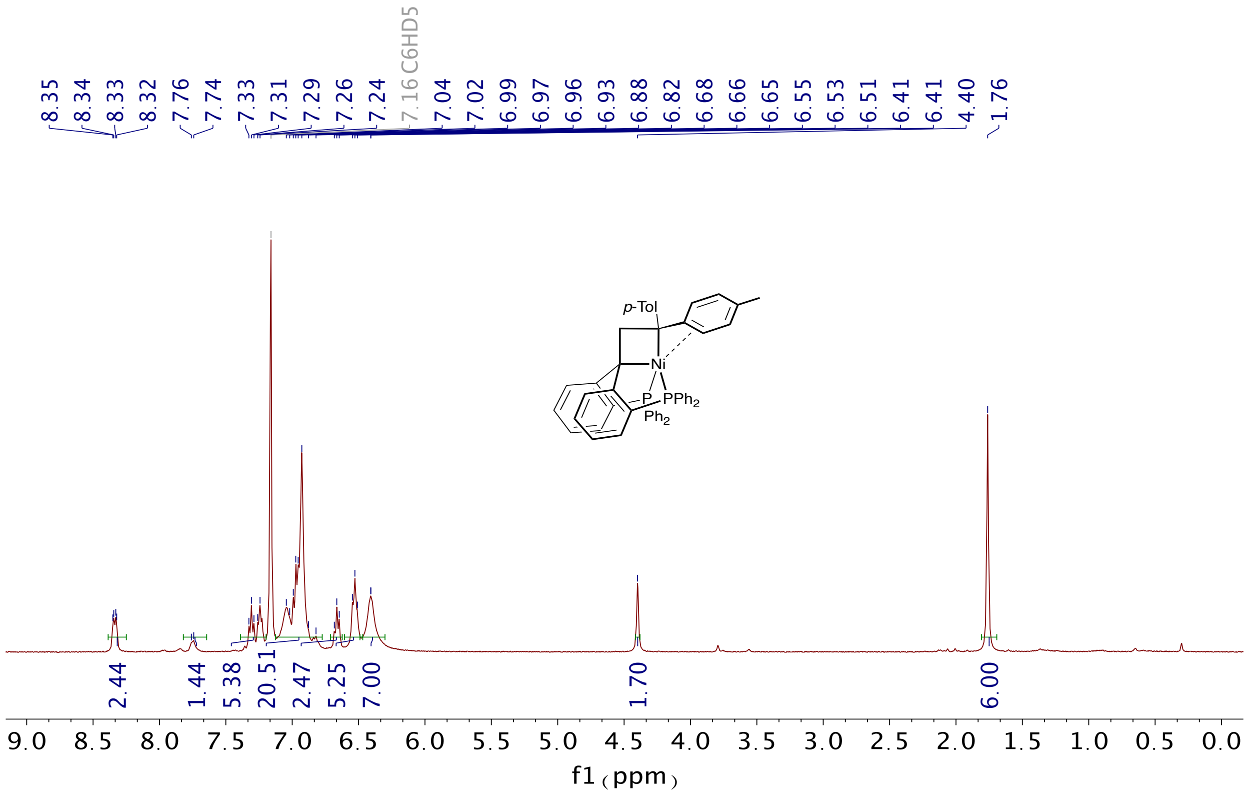

Figure S42. ^{1}H NMR, C_6D_6, 25 ºC, complex **4**.

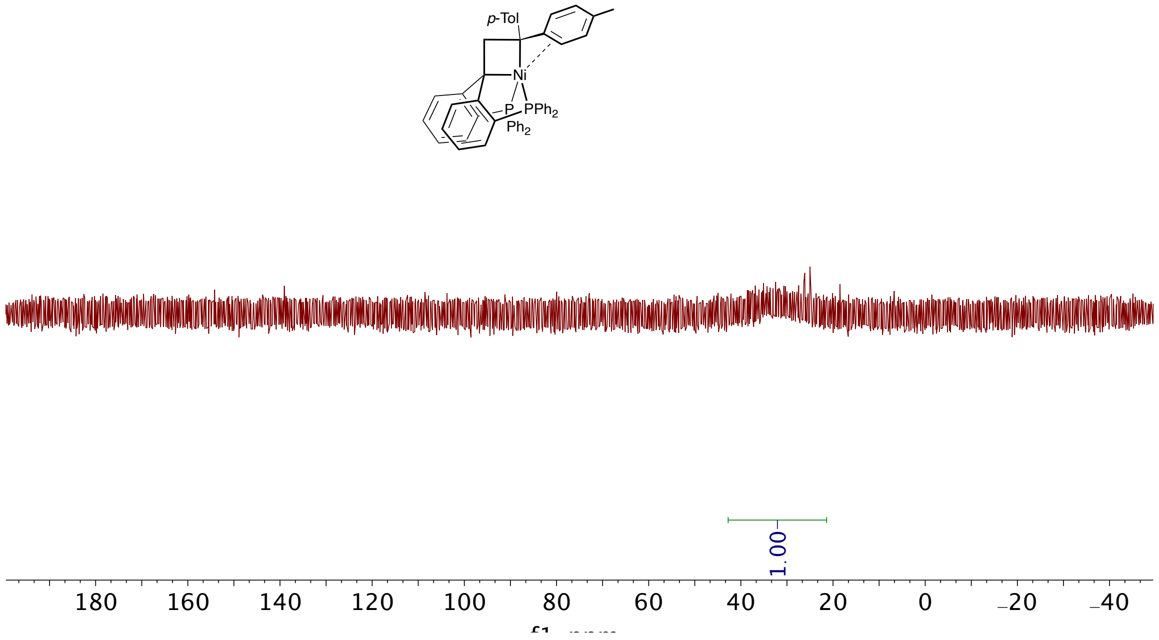

Figure S43. $^{31}P\{^{1}H\}$ NMR, C_6D_6, 25 ºC, complex **4**.

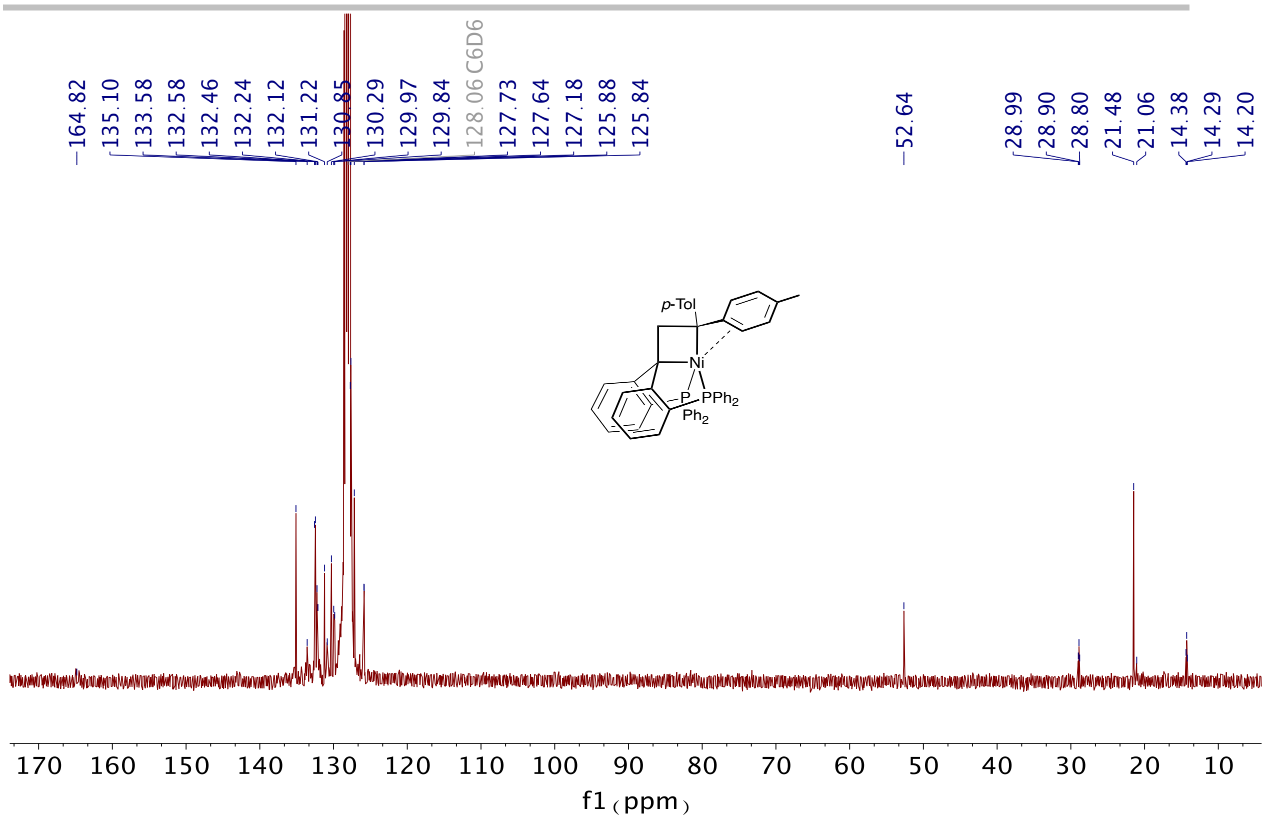

Figure S44. ^{13}C NMR, C_6D_6, 25 ºC, complex **4**.

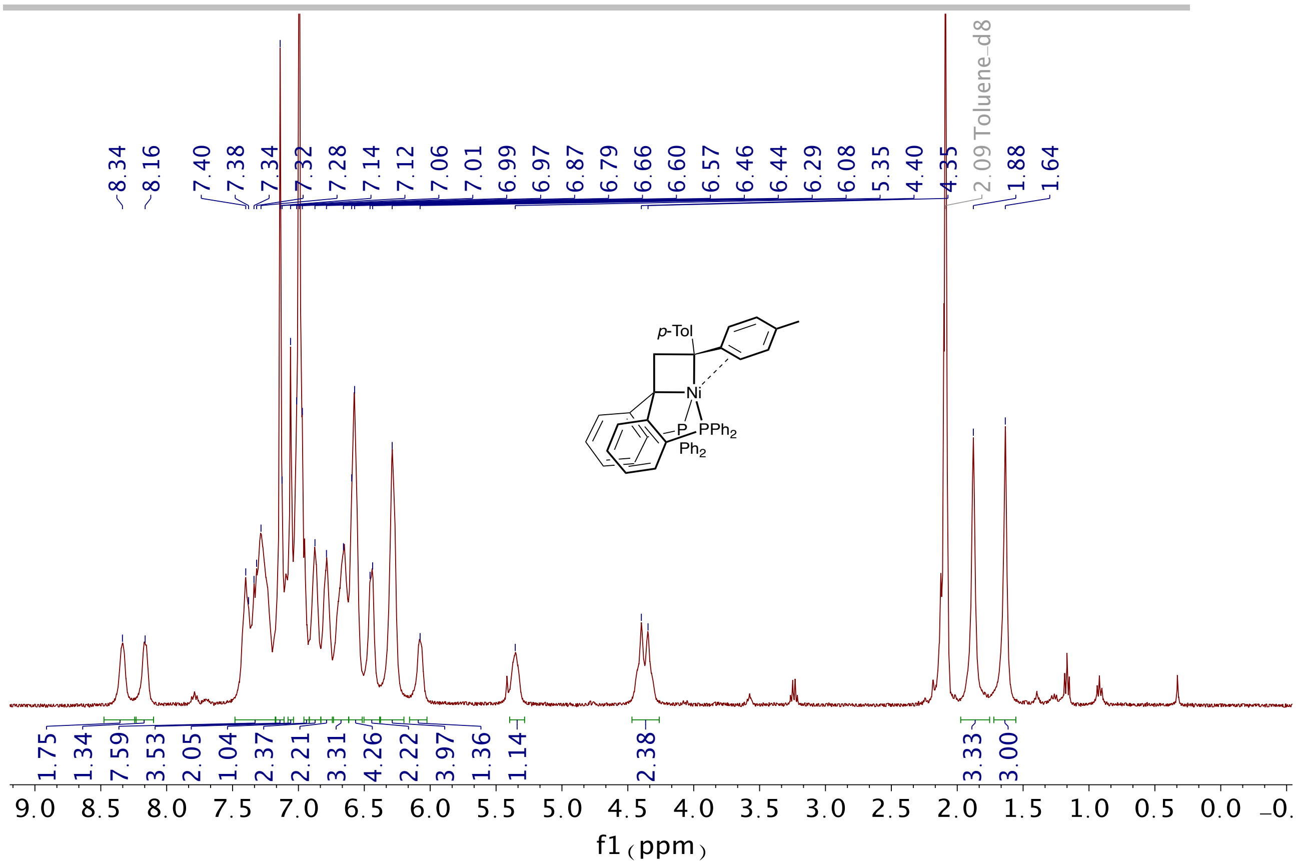

Figure S45. ^{1}H NMR, d^{8}-tol, -40 ºC, complex **4**.

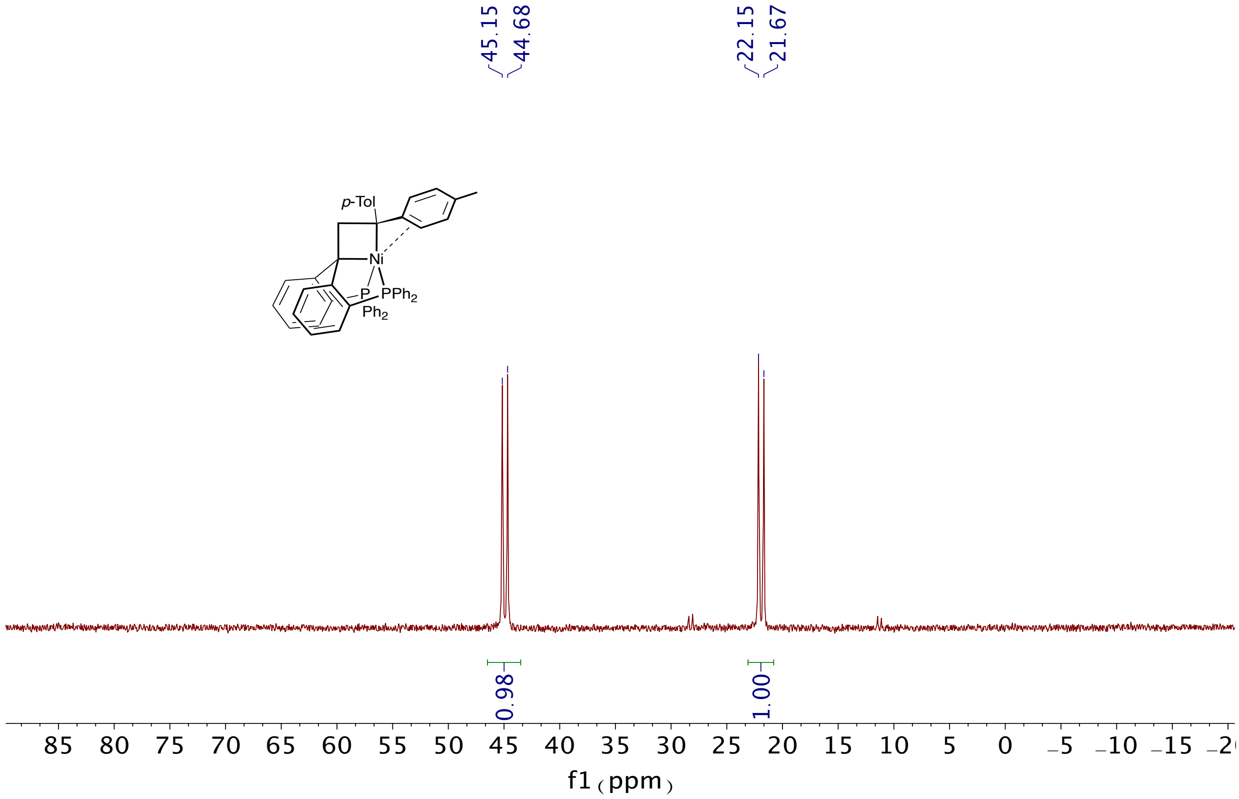

Figure S46. $^{31}P\{^1H\}$ NMR, d^8-tol, -40 ºC, complex **4**.

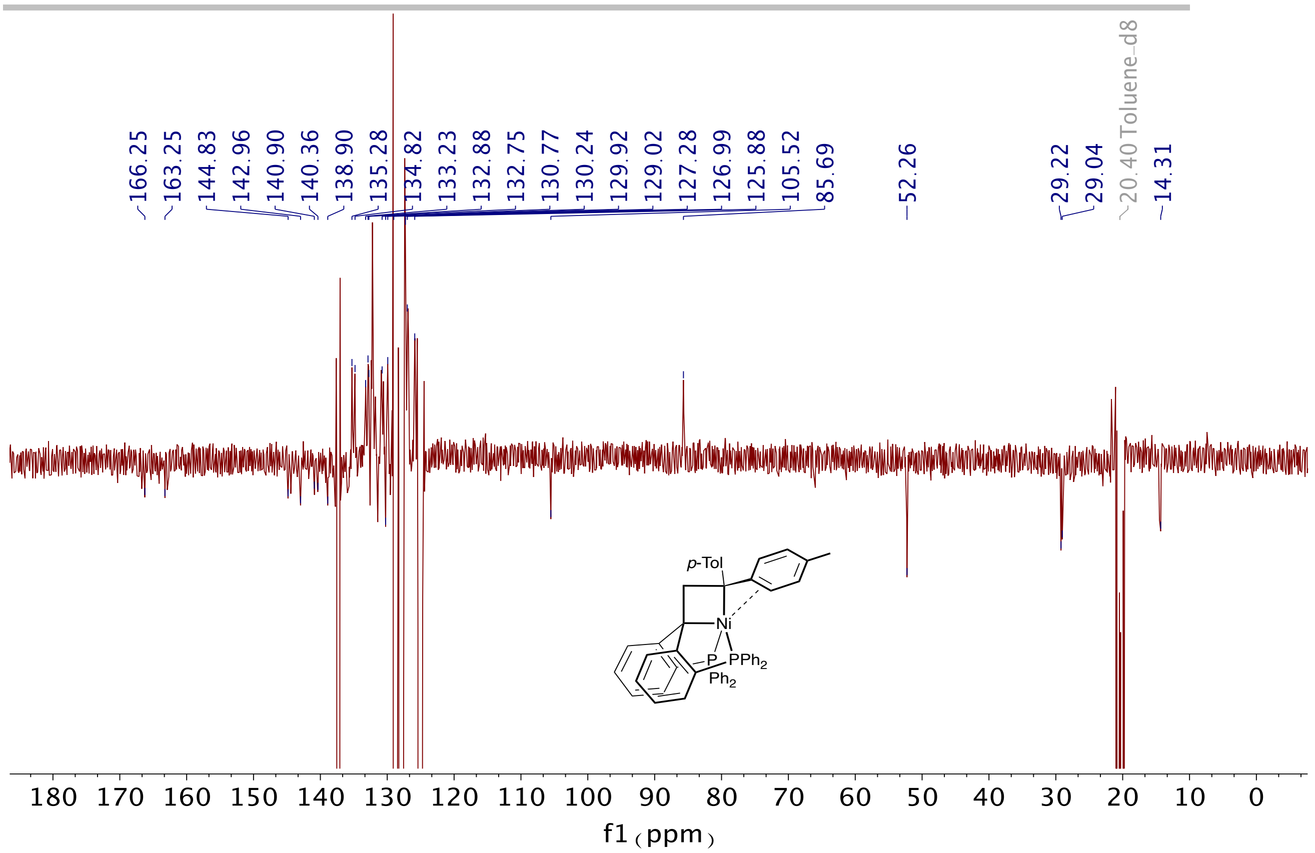

Figure S47. APT, d[8]-tol, -40 ºC, complex **4**.

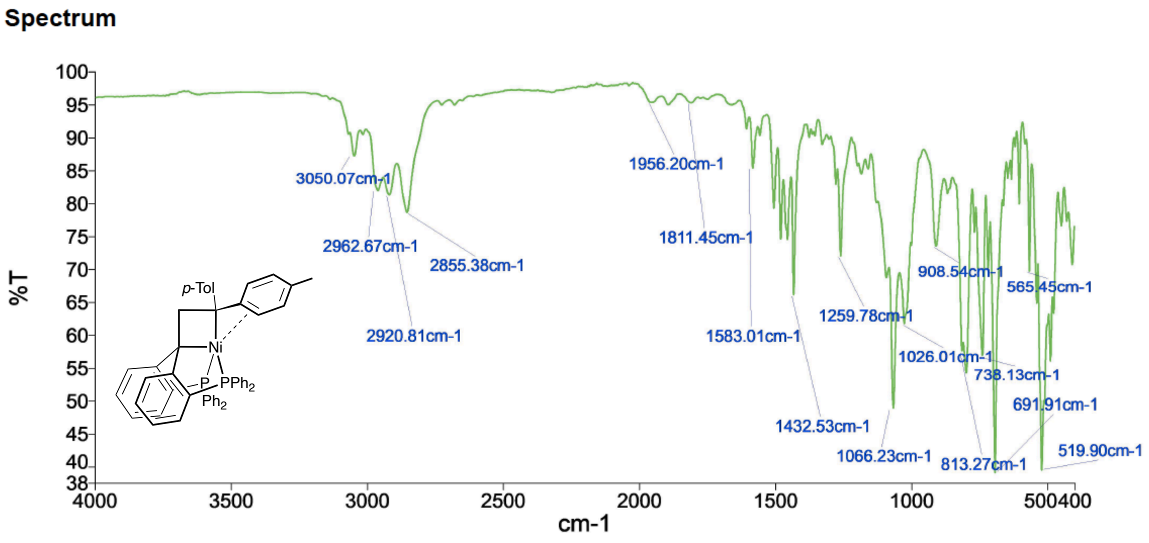

Figure S48. IR complex **4**.

d[2]-Nickelacyclobutane (4b)

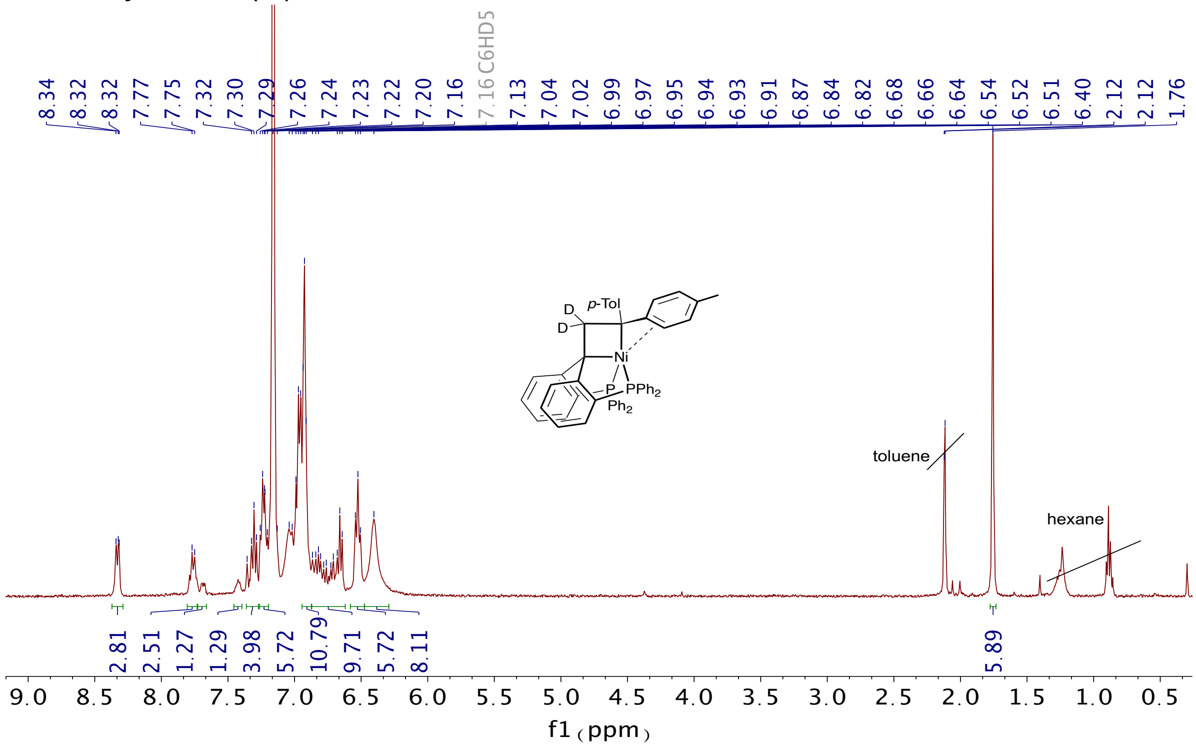

Figure S49. ^{1}H NMR, C_6D_6, 25 ºC, complex **4b**.

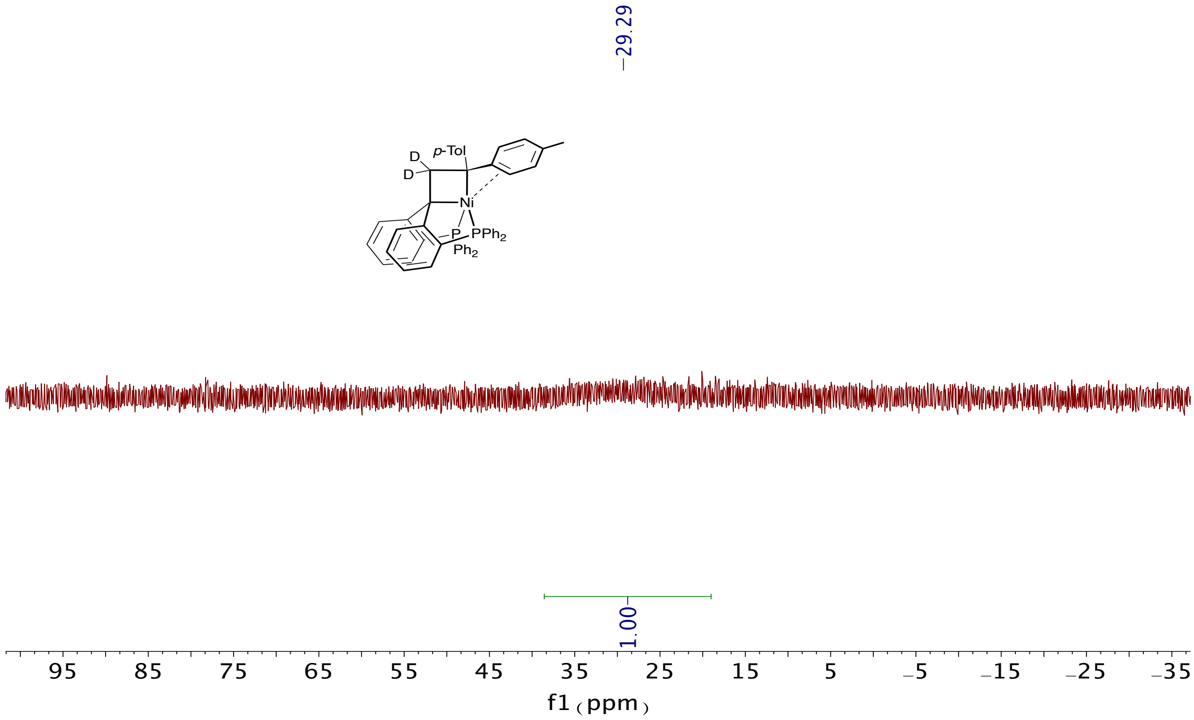

Figure S50. $^{31}P\{^{1}H\}$ NMR, C_6D_6, 25 ºC, complex **4b**.

4-CO

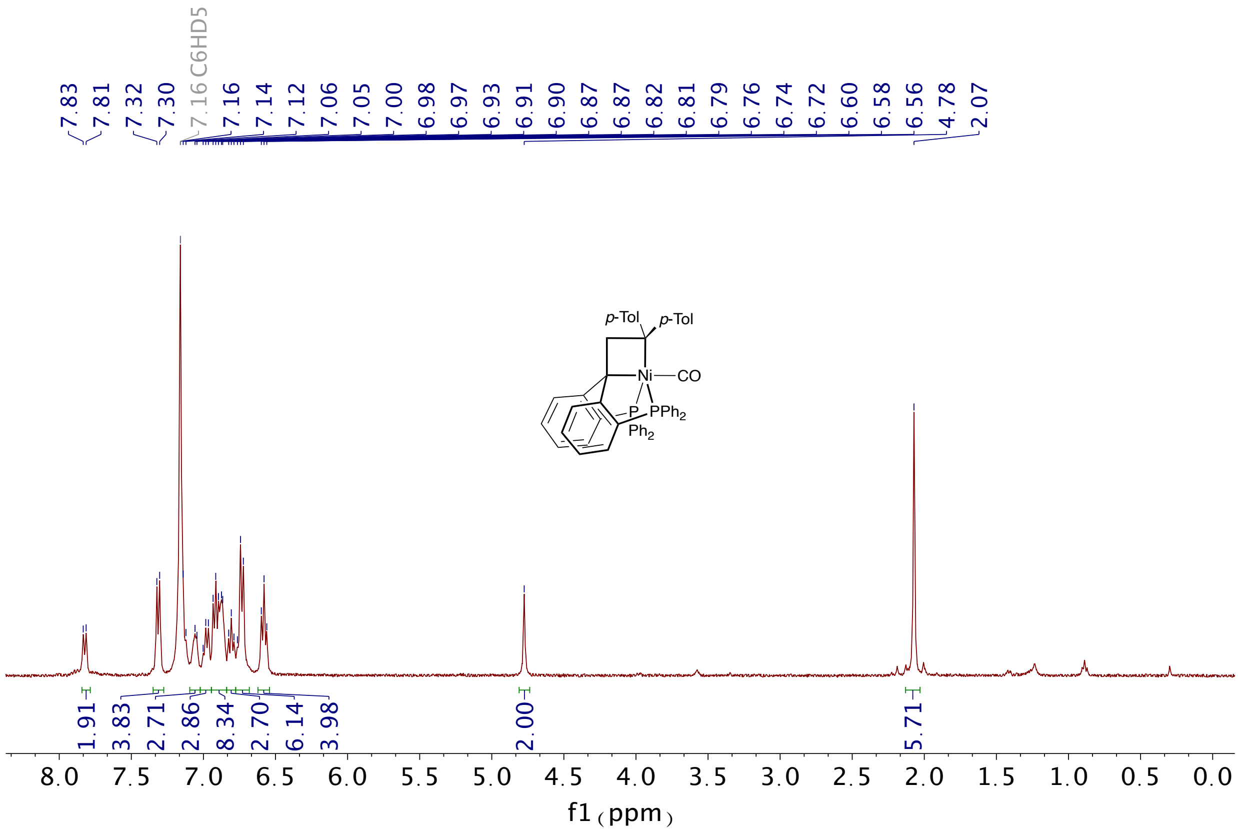

Figure S51. ^{1}H NMR, C_6D_6, 25 ºC, **4-CO**.

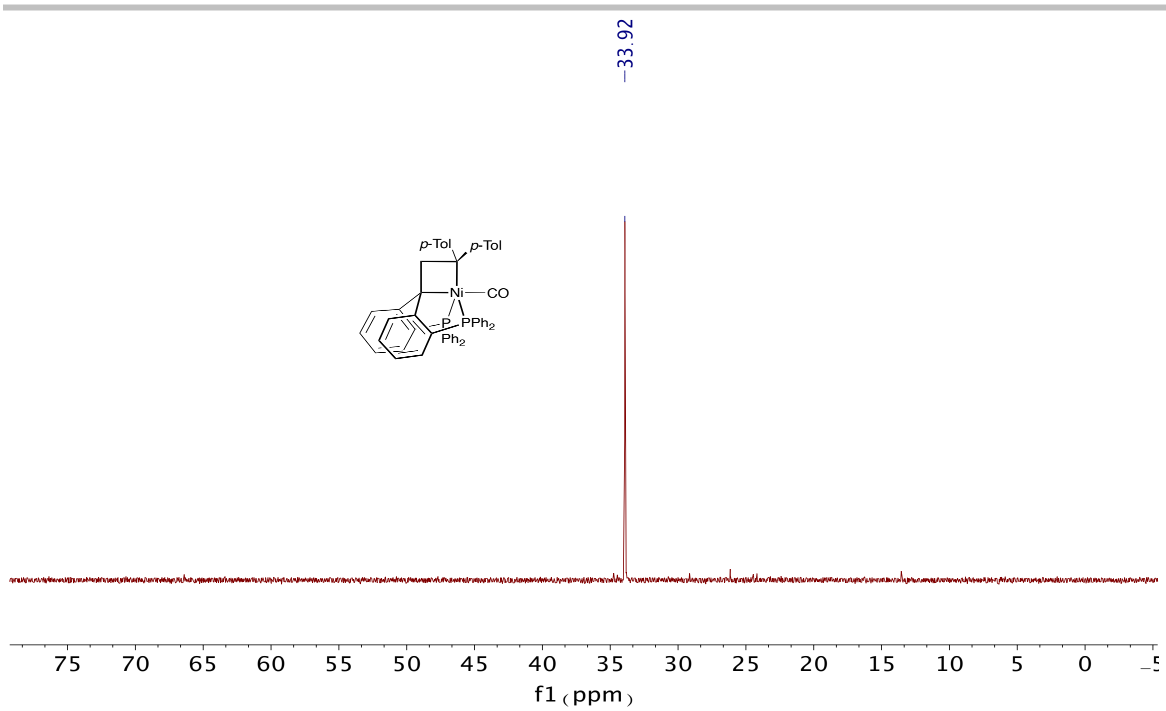

Figure S52. $^{31}P\{^{1}H\}$ NMR, C_6D_6, 25 ºC, complex **4-CO.**

4-CD$_3$CN

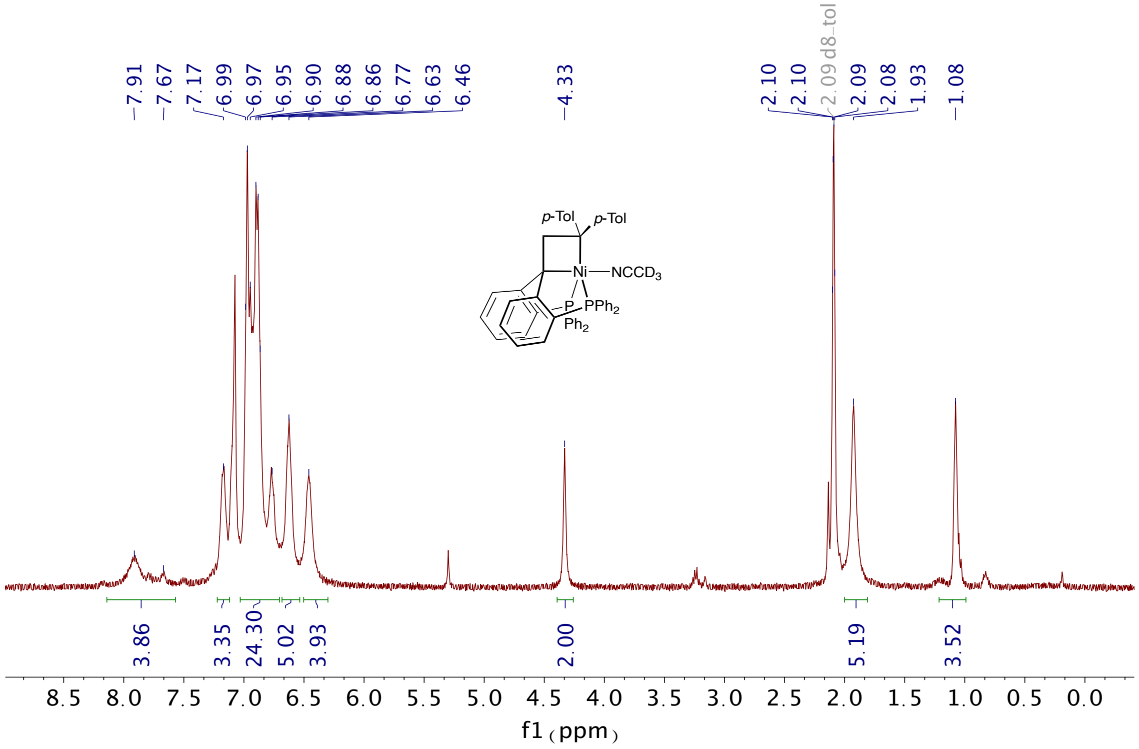

Figure S53. ^{1}H NMR, C_6D_6, 25 ºC, **4-CD$_3$CN**.

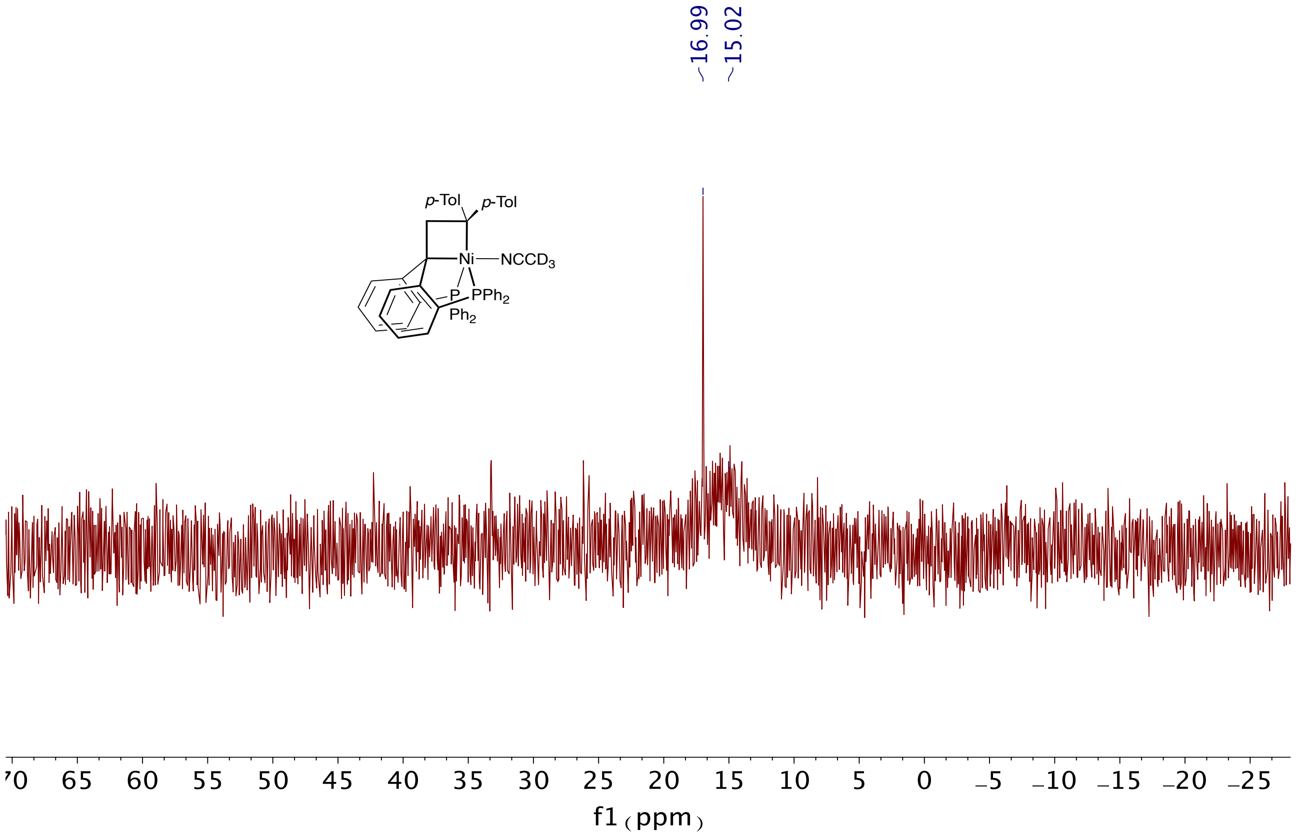

Figure S54. $^{31}P\{^{1}H\}$ NMR, C_6D_6, 25 ºC, complex **4-CD$_3$CN.**

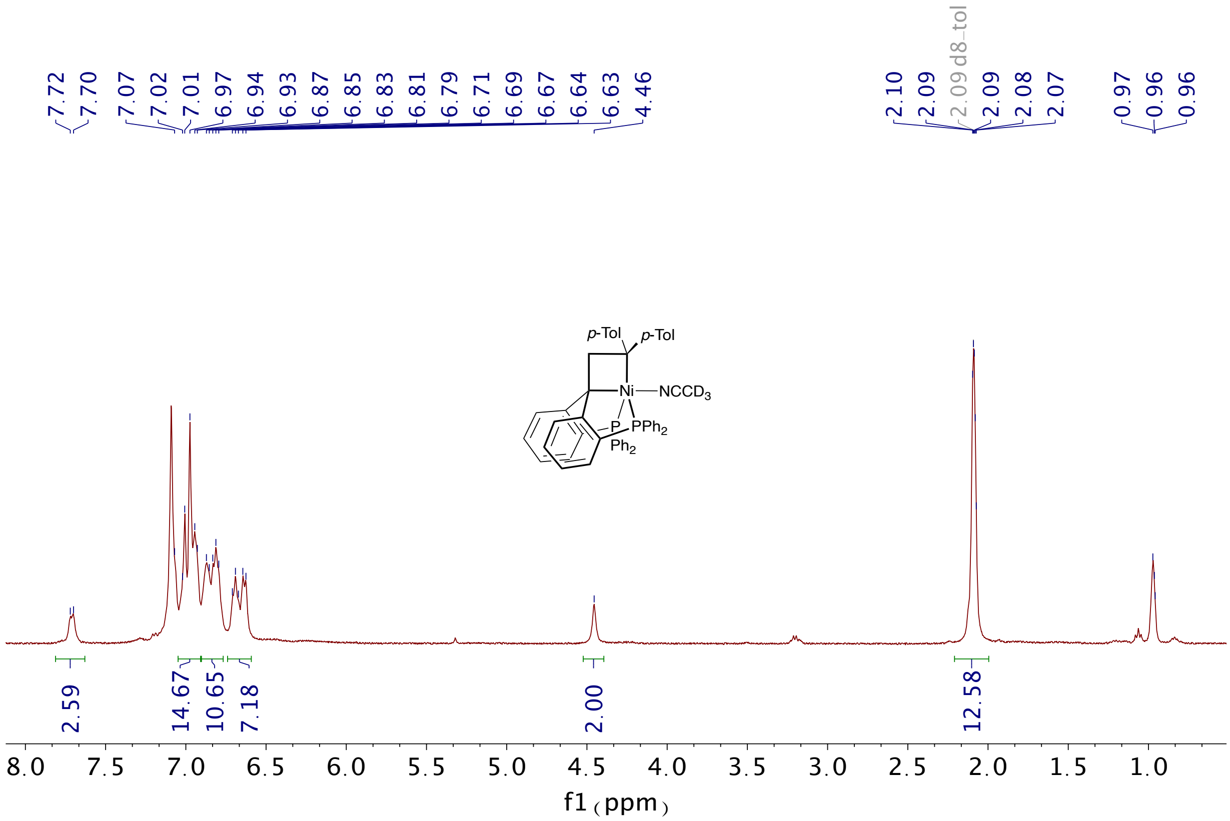

Figure S55. ^{1}H NMR, d^{8}-tol, -30 ºC, **4-CD$_3$CN**.

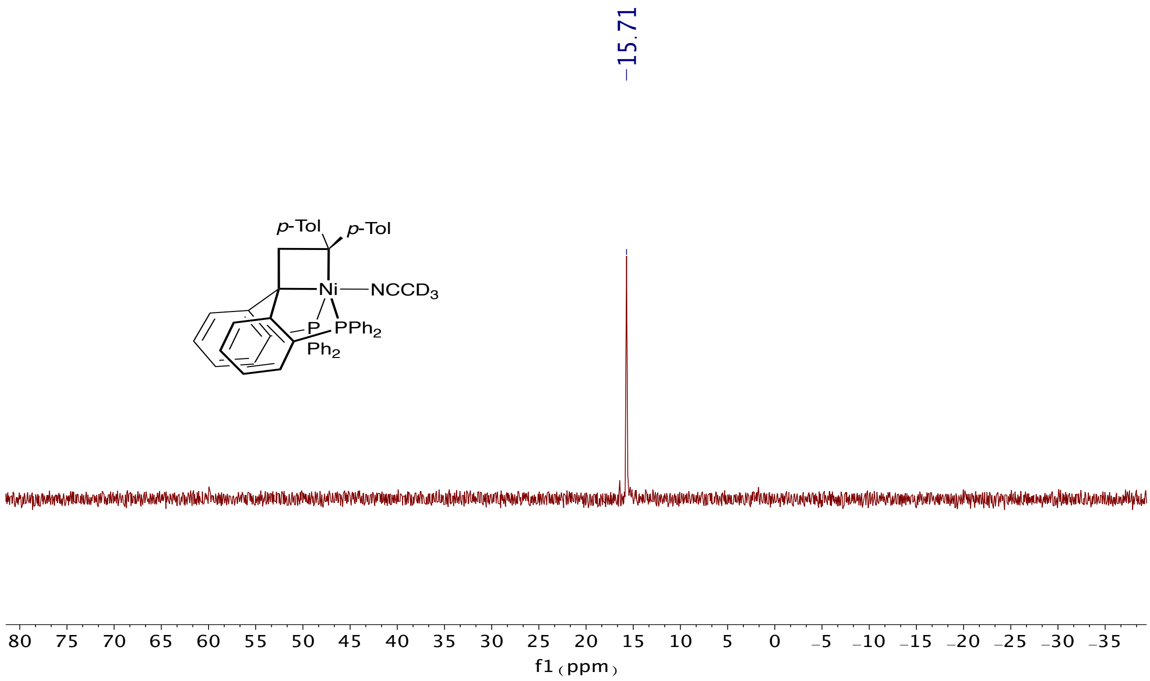

Figure S56. $^{31}P\{^{1}H\}$ NMR, d^{8}-tol, -30 ºC, complex **4-CD$_3$CN.**

Cyclopropane Ni(CO)$_2$ (5)

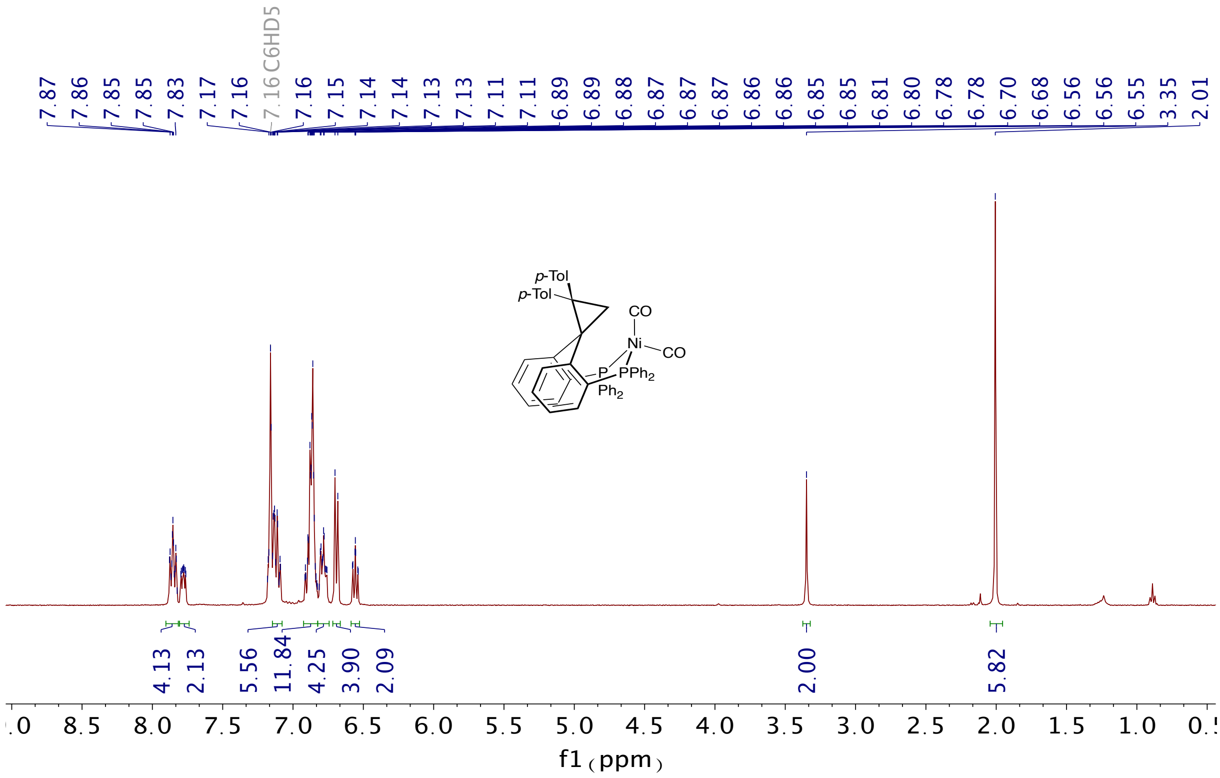

Figure S57. ^1H NMR, C_6D_6, 25 ºC, complex **5**.

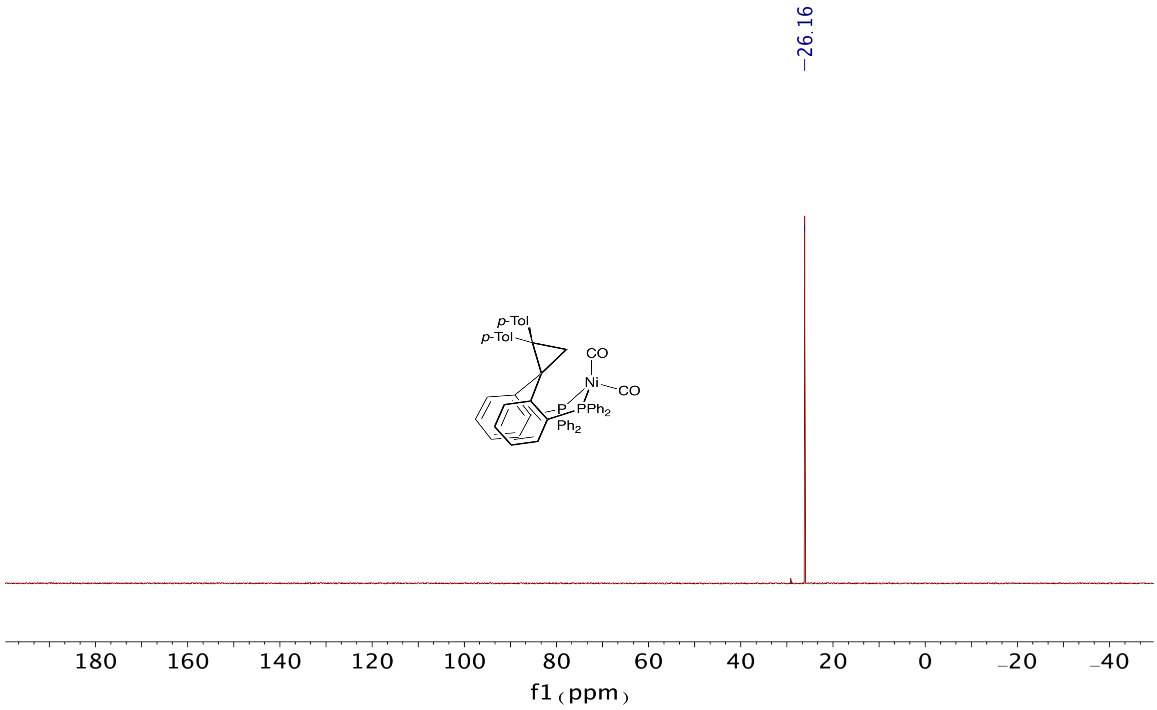

Figure S58. $^{31}P\{^1H\}$ NMR, C_6D_6, 25 ºC, complex **5**.

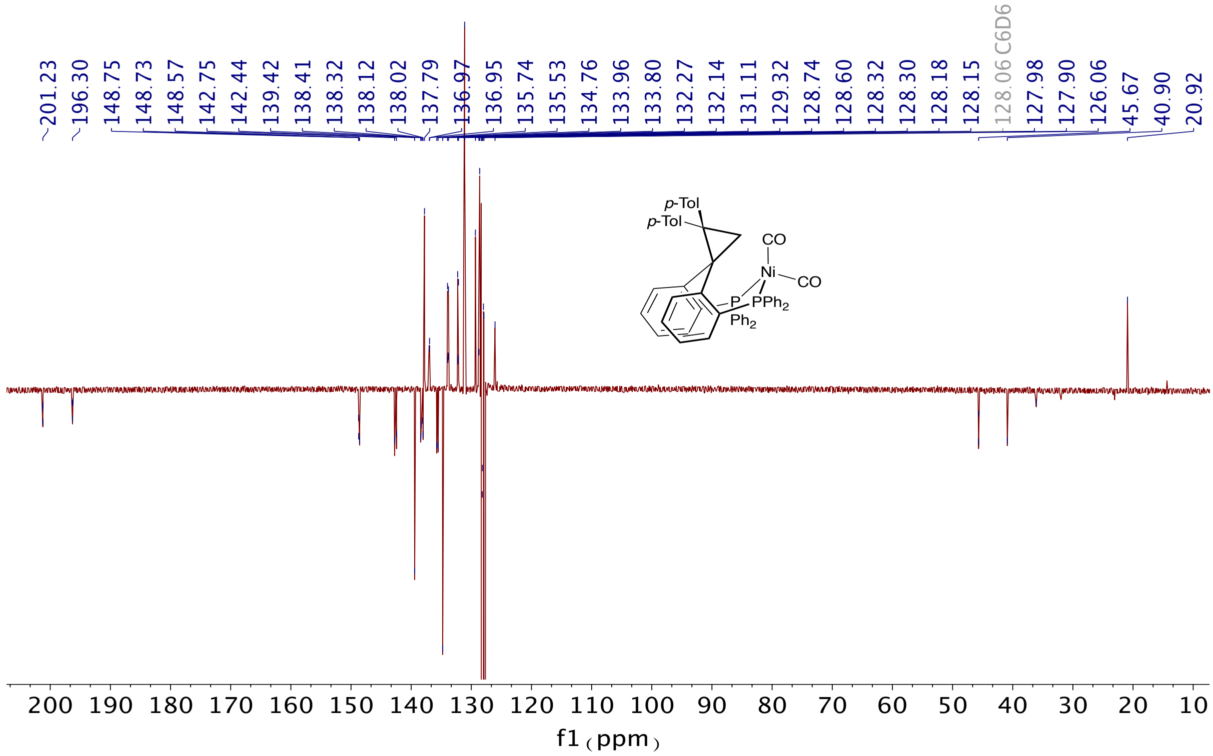

Figure S59. APT, C_6D_6, 25 ºC, complex **5**.

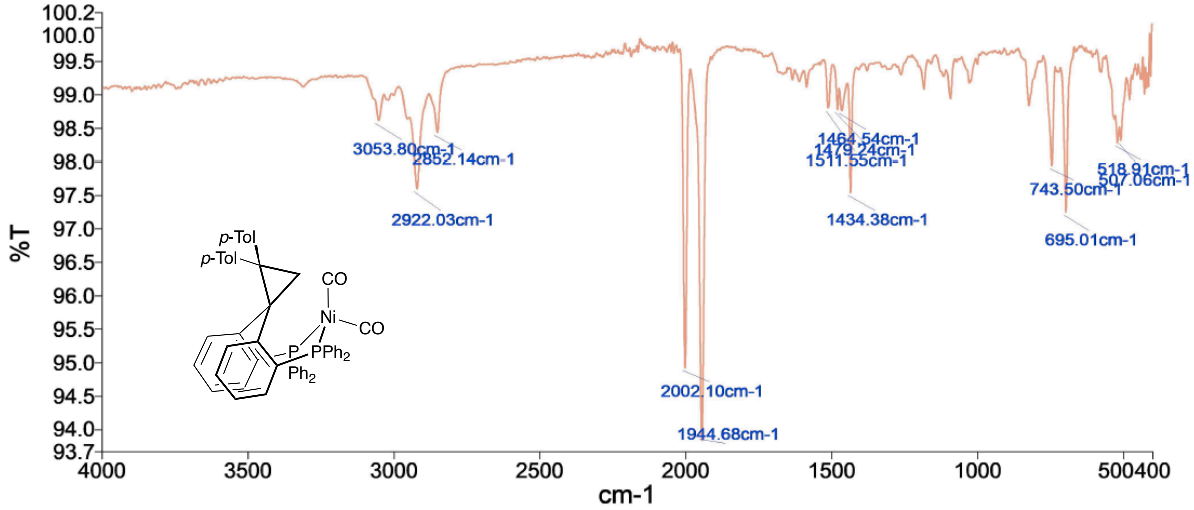

Figure S60. IR complex **5**.

(^{Ph}PCHP^{Ph})Ni(CH_2CN) (6)

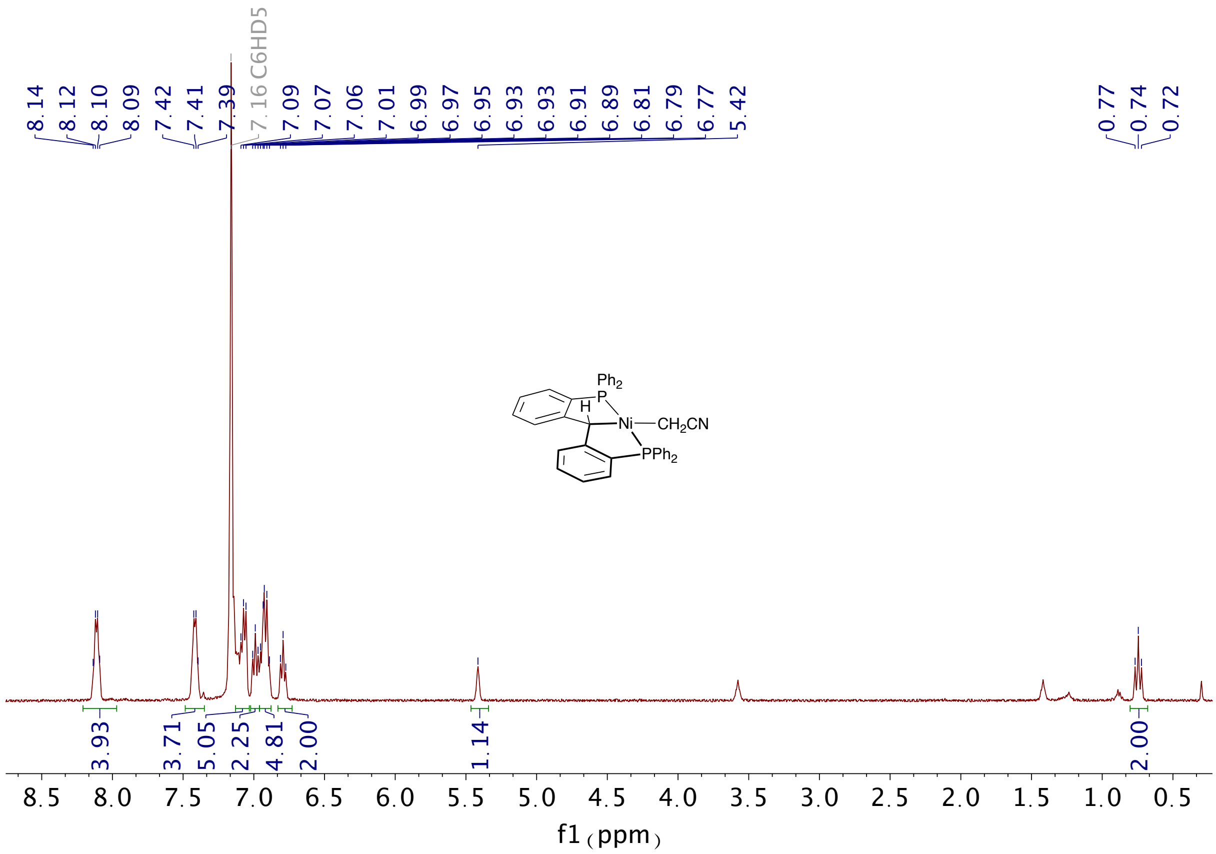

Figure S61. ^{1}H NMR, C_6D_6, 25 ºC, complex **6**.

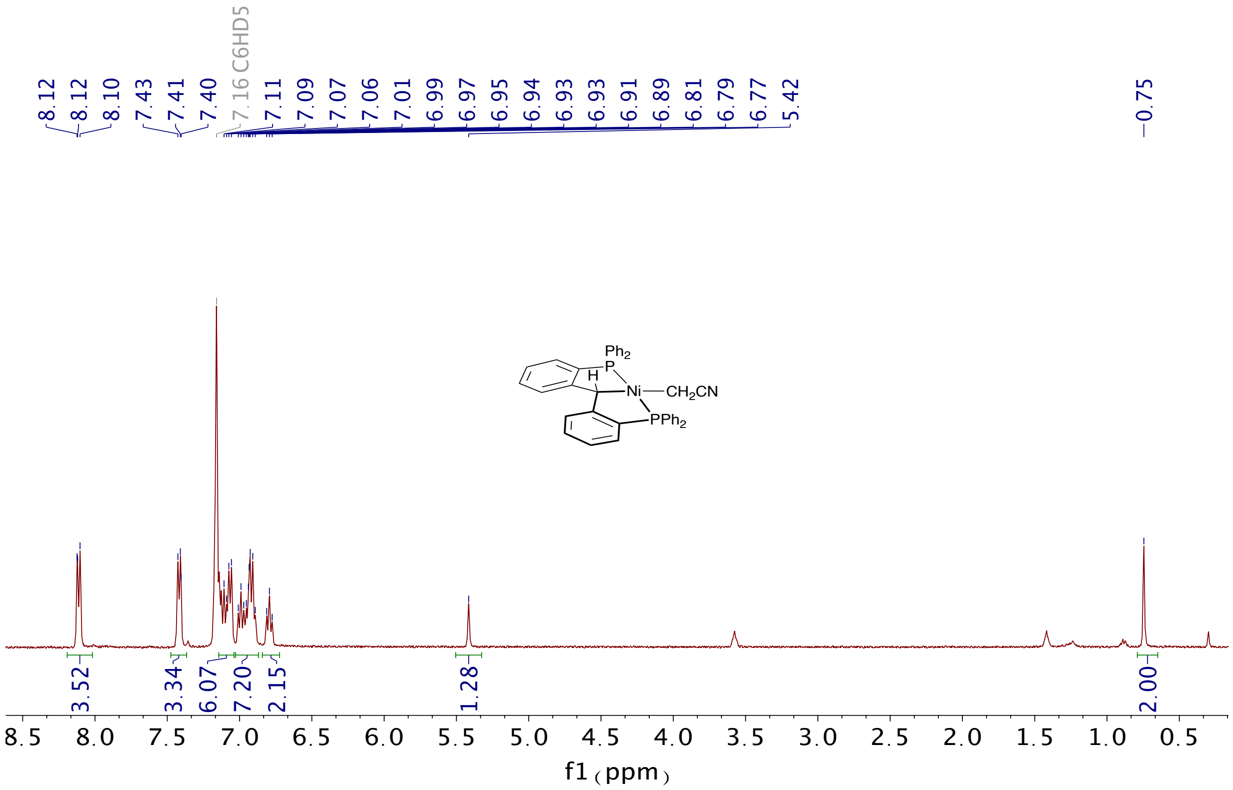

Figure S62. $^{1}H\{^{31}P\}$ NMR, C_6D_6, 25 ºC, complex **6**.

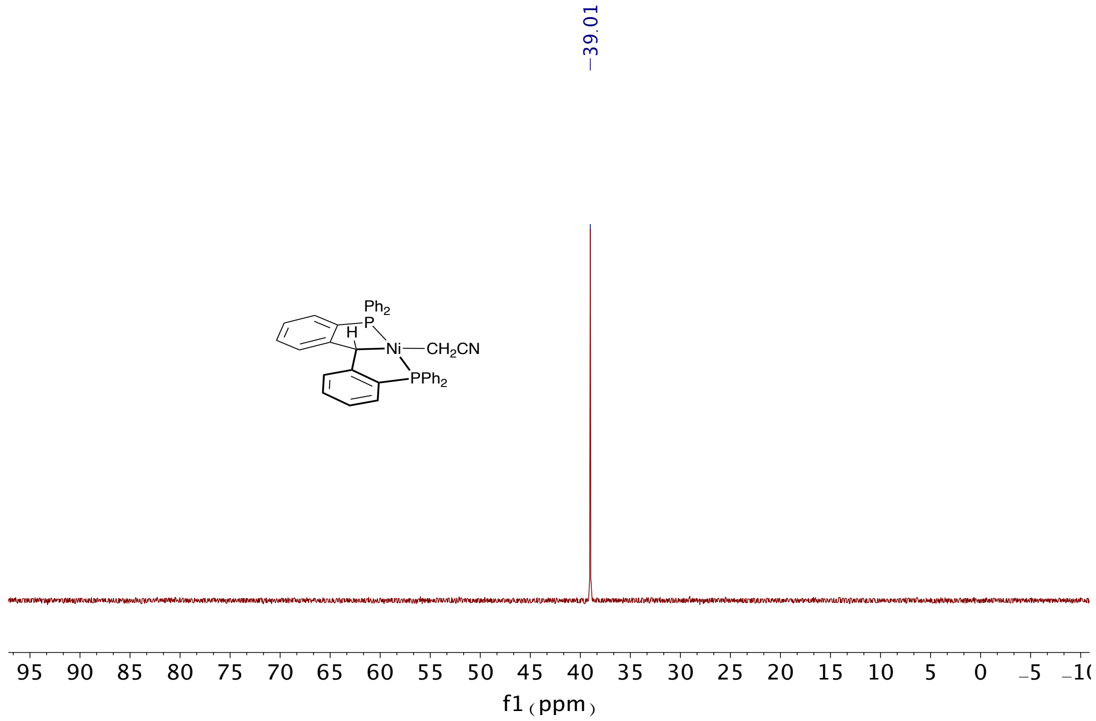

Figure S63. $^{31}P\{^{1}H\}$ NMR, C_6D_6, 25 ºC, complex **6**.

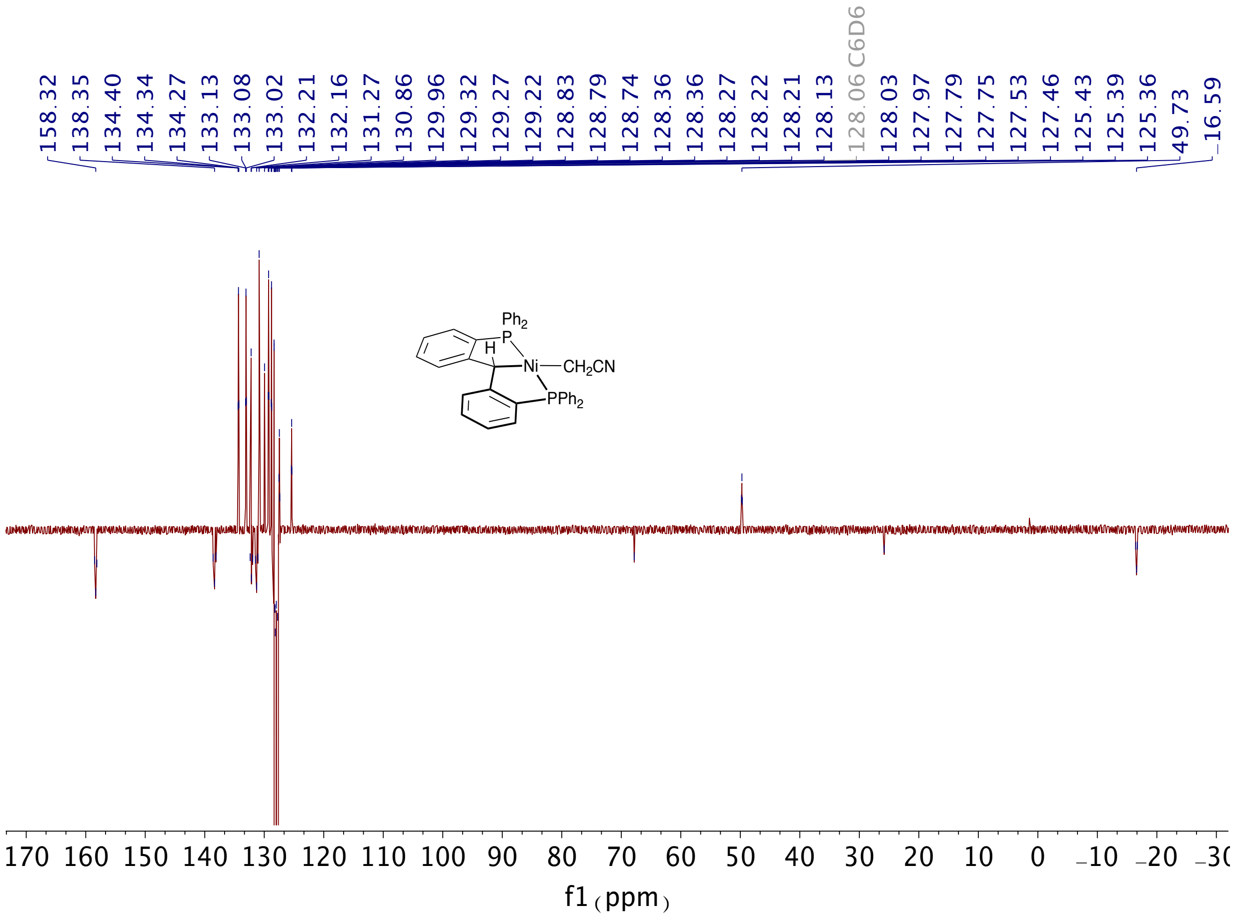

Figure S64. APT, C_6D_6, 25 ºC, complex **6**.

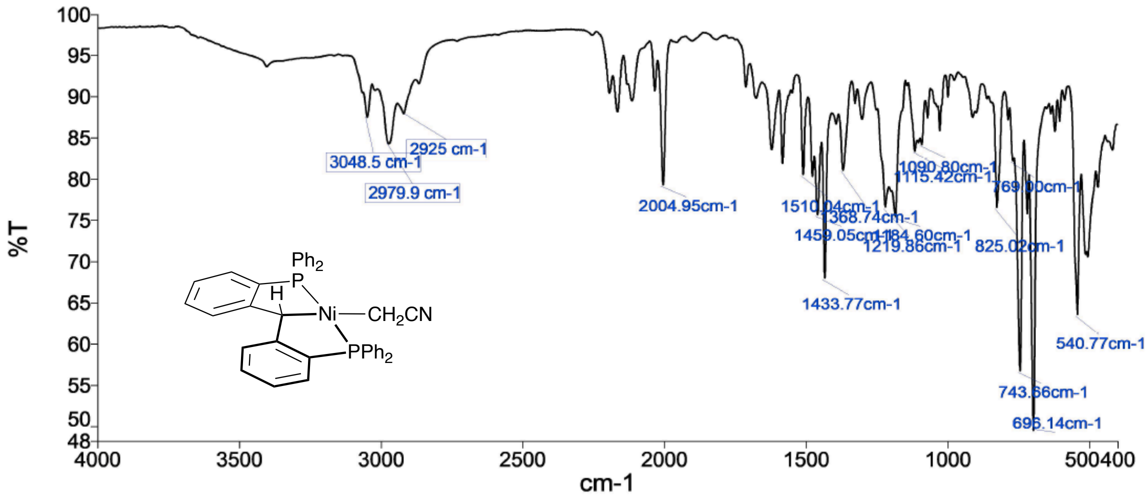

Figure S65. IR complex

[(^{Ph}bppe^{H,CHptol2})Ni]$_2$N$_2$ (7)

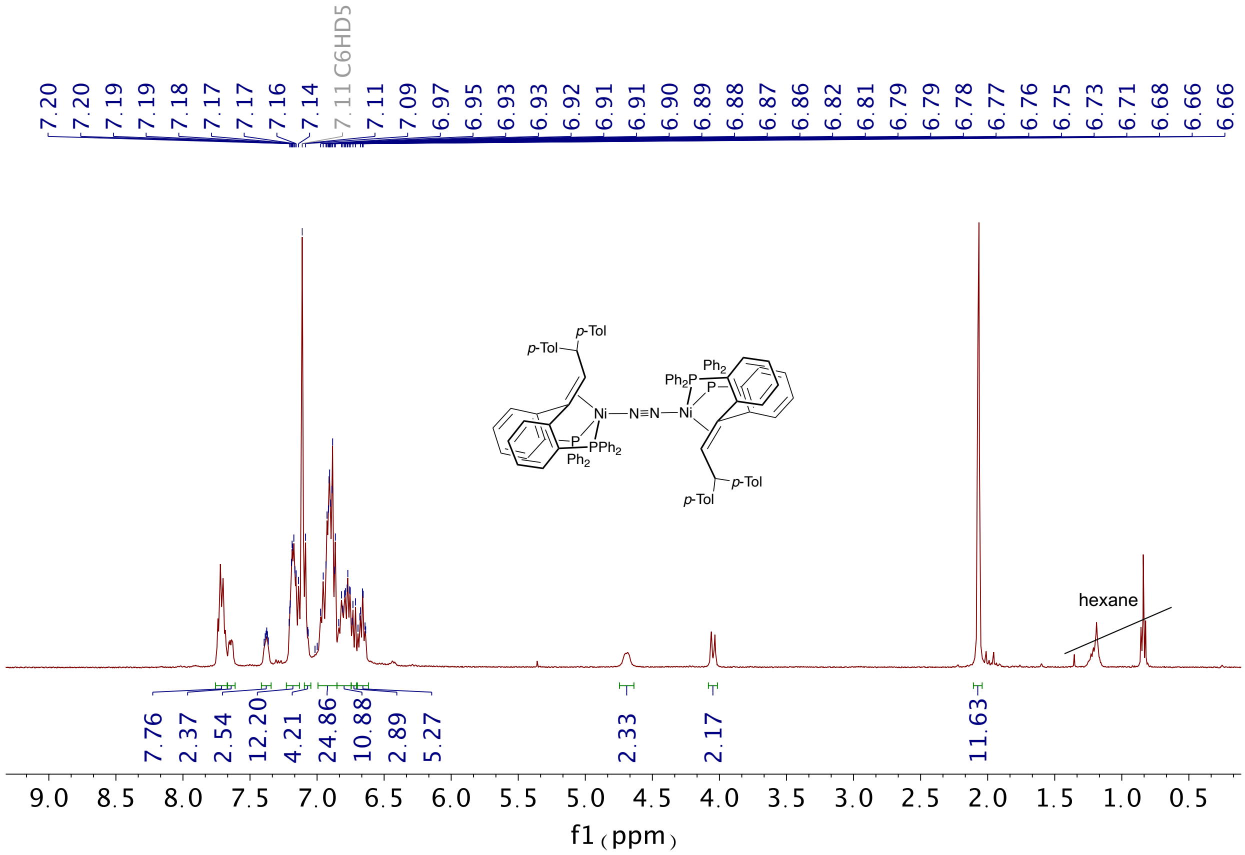

Figure S66. 1H NMR, C_6D_6, 25 ºC, complex 7.

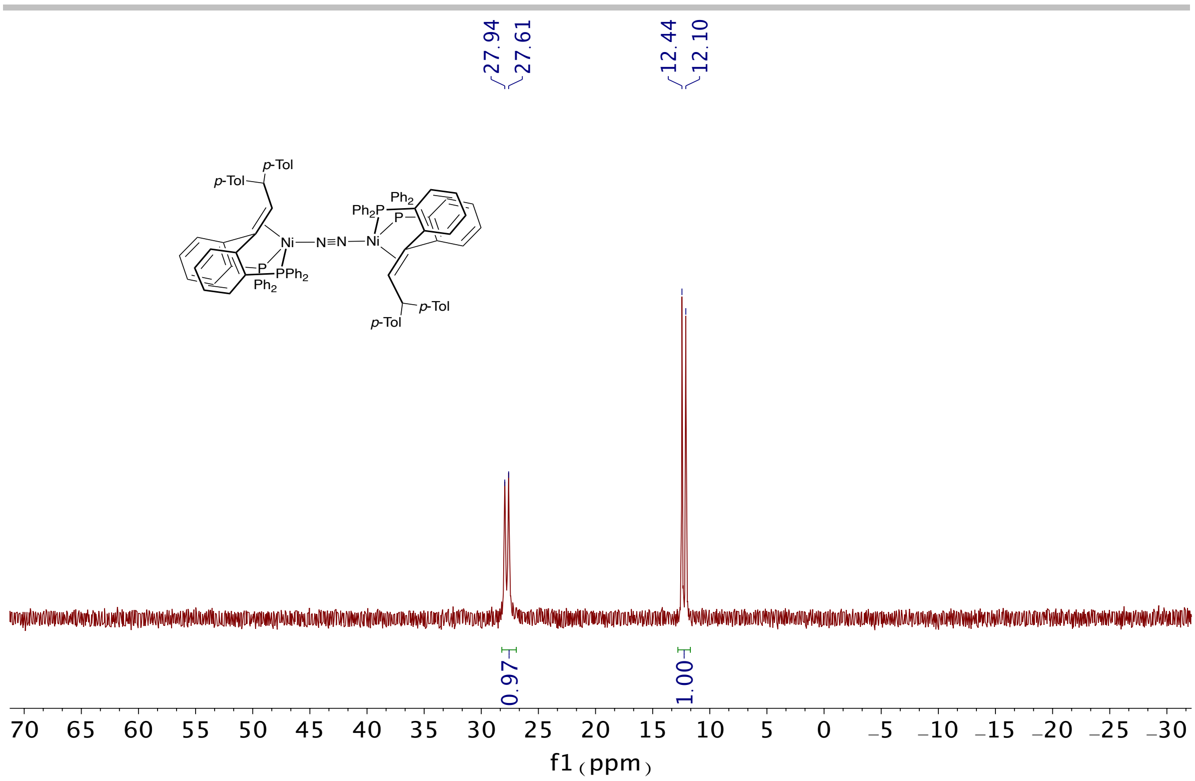

Figure S67. $^{31}P\{^{1}H\}$ NMR, C_6D_6, 25 ºC, complex **7**.

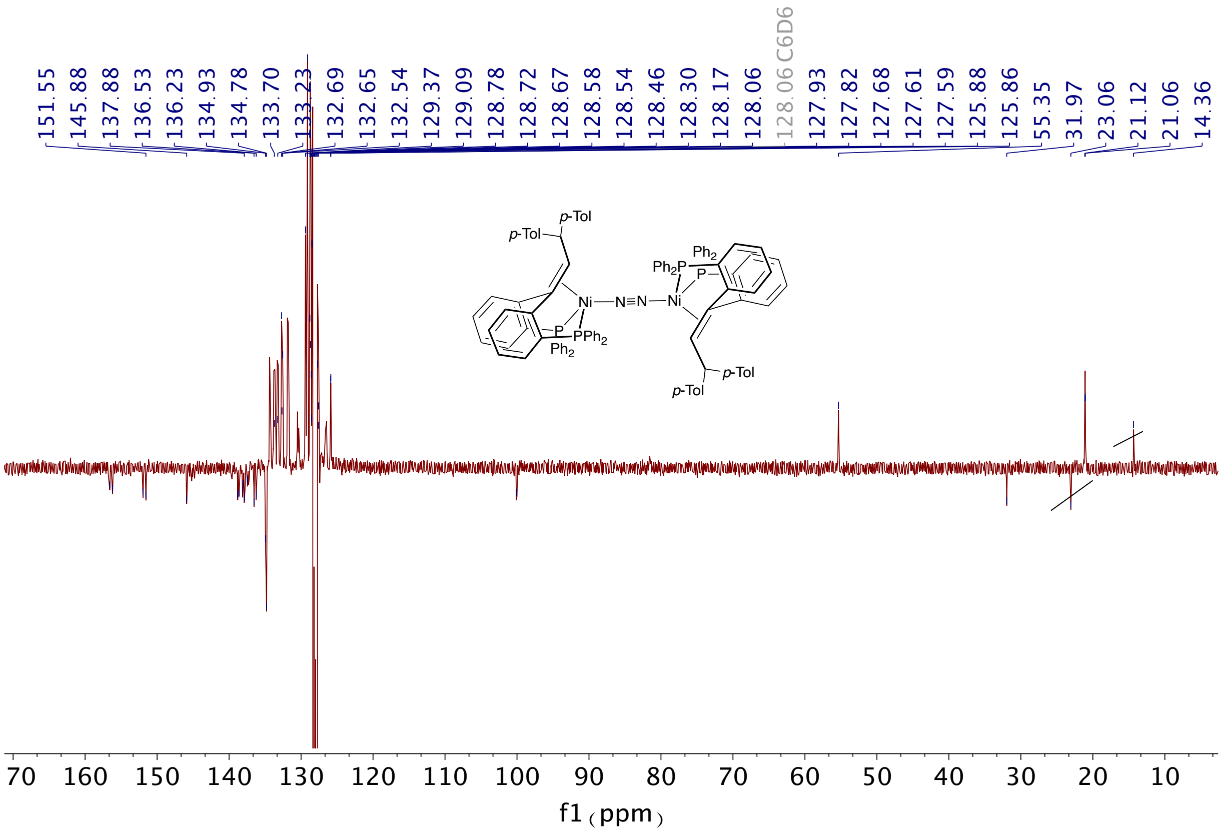

Figure S68. APT, C_6D_6, 25 ºC, complex **7**.

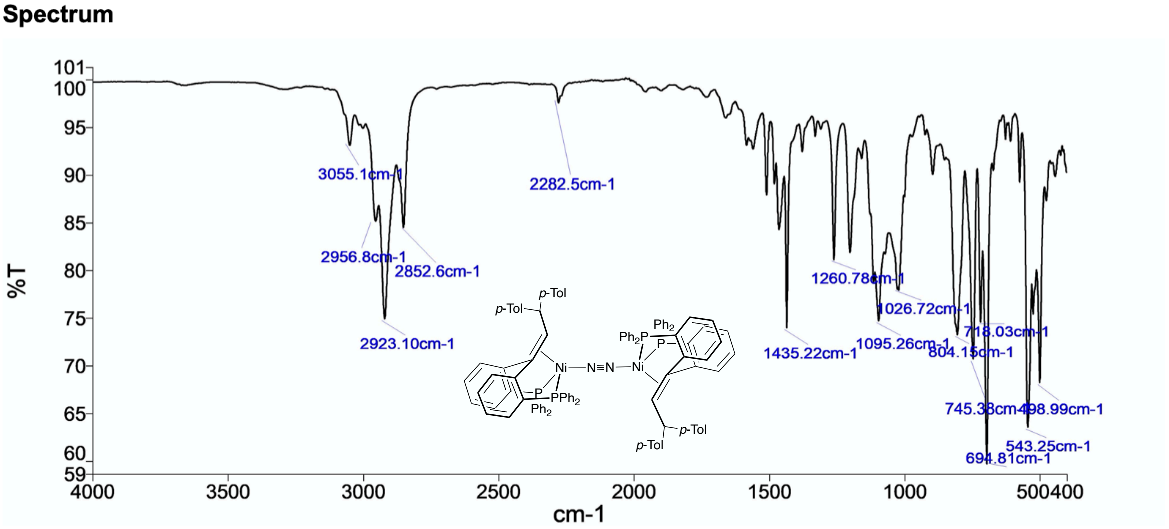

Figure S69. IR complex **7**.

(^{Ph}bppe^{H,CHptol2})Ni(PhCN) (8)

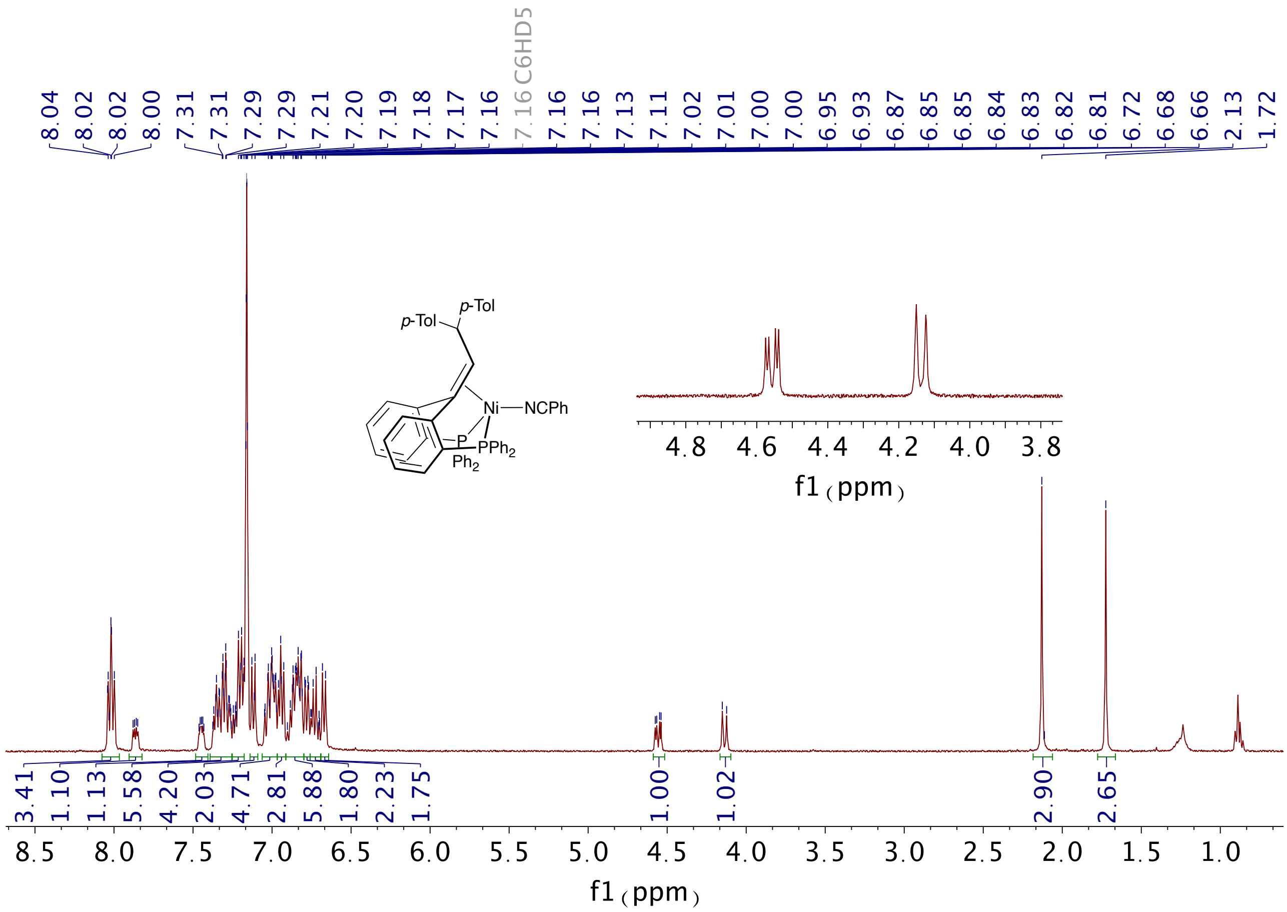

Figure S70. ^{1}H NMR, C_6D_6, 25 ºC, complex **8**.

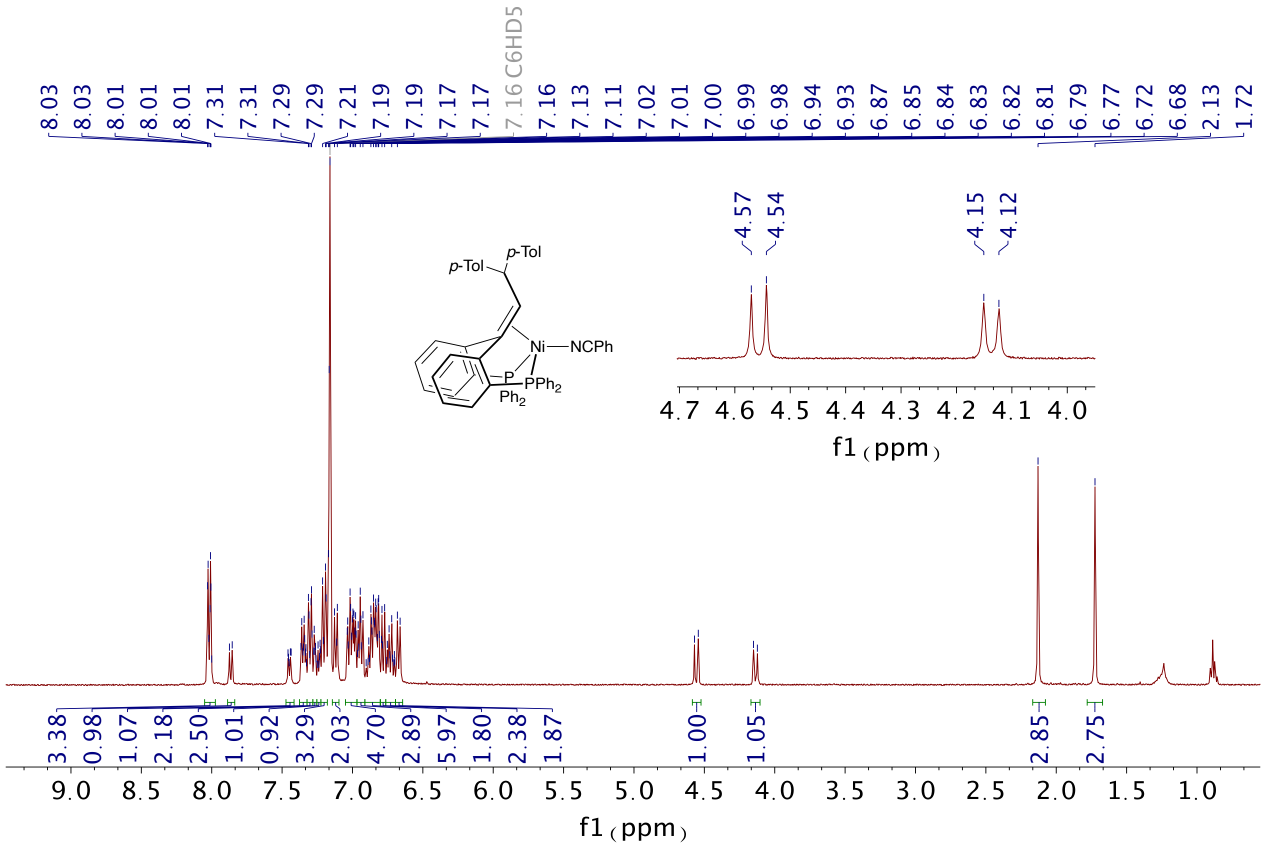

Figure S71. $^{1}H\{^{31}P\}$ NMR, 25 ºC, complex **8**.

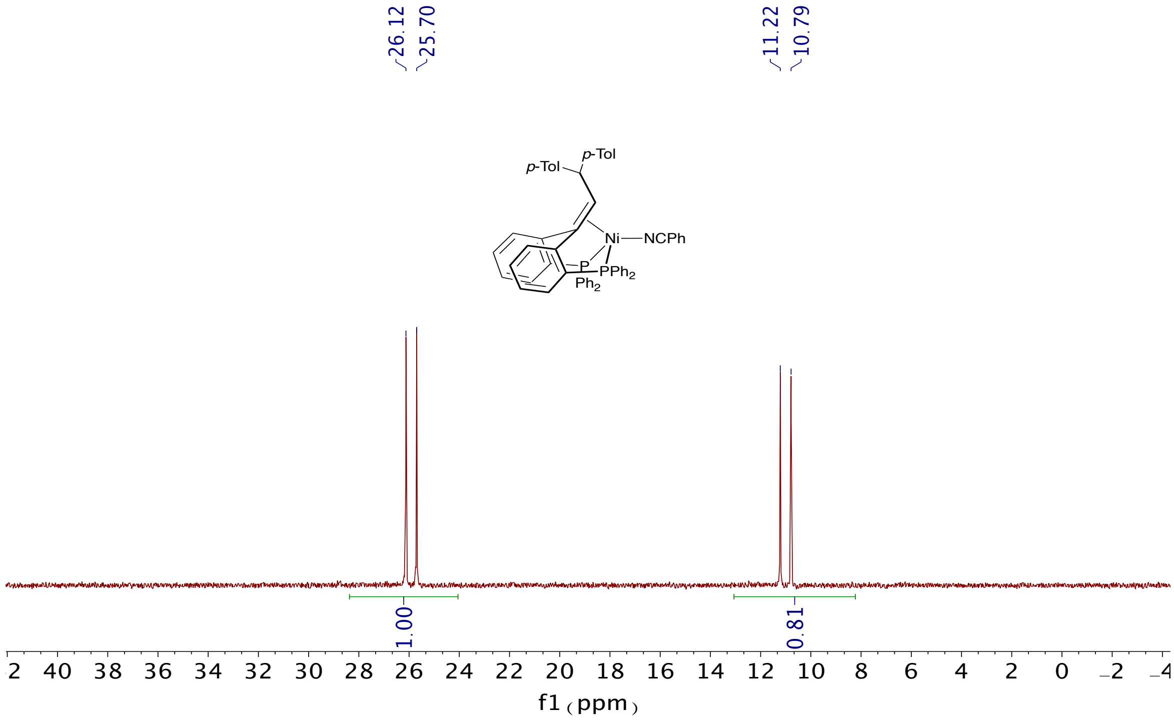

Figure S72. $^{31}P\{^{1}H\}$ NMR, C_6D_6, 25 ºC, complex **8**.

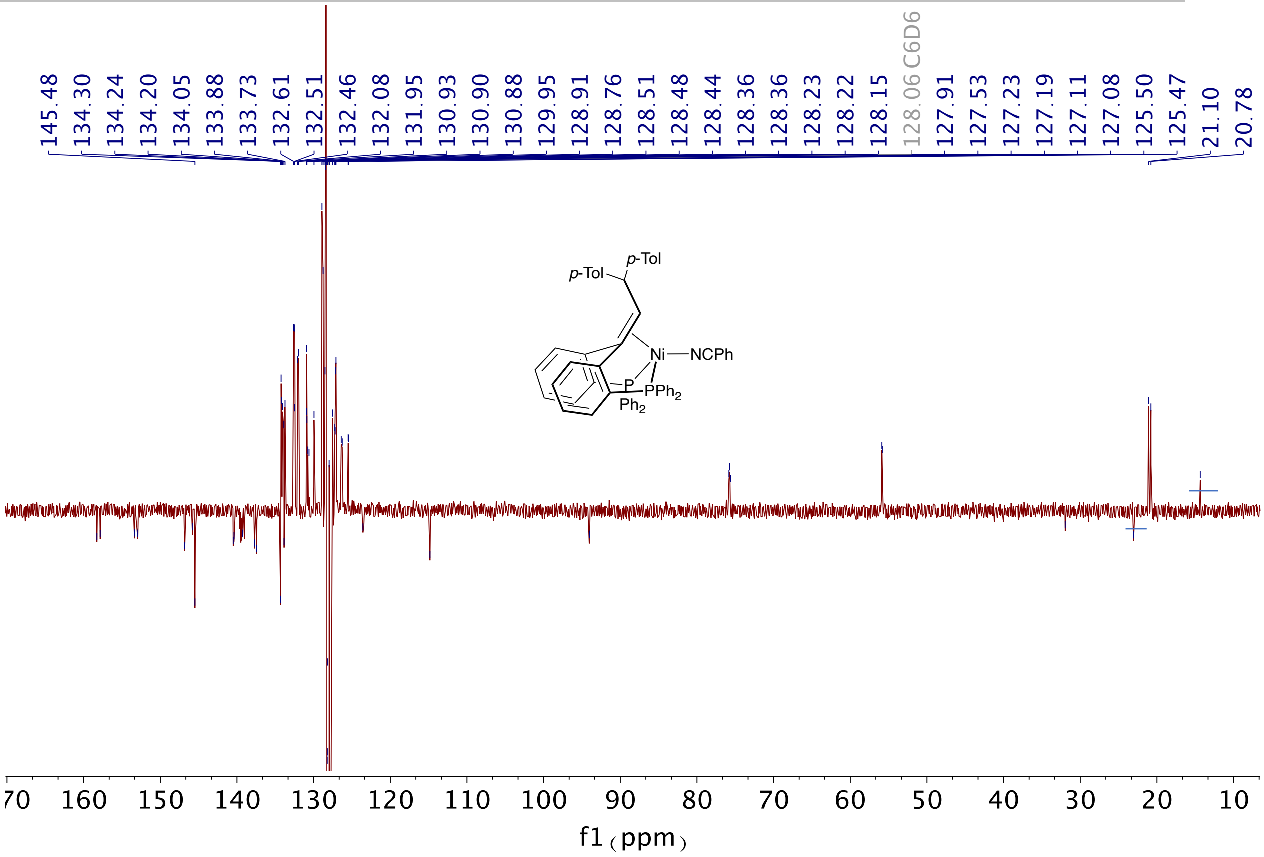

Figure S73. APT, C_6D_6, 25 ºC, complex **8**.

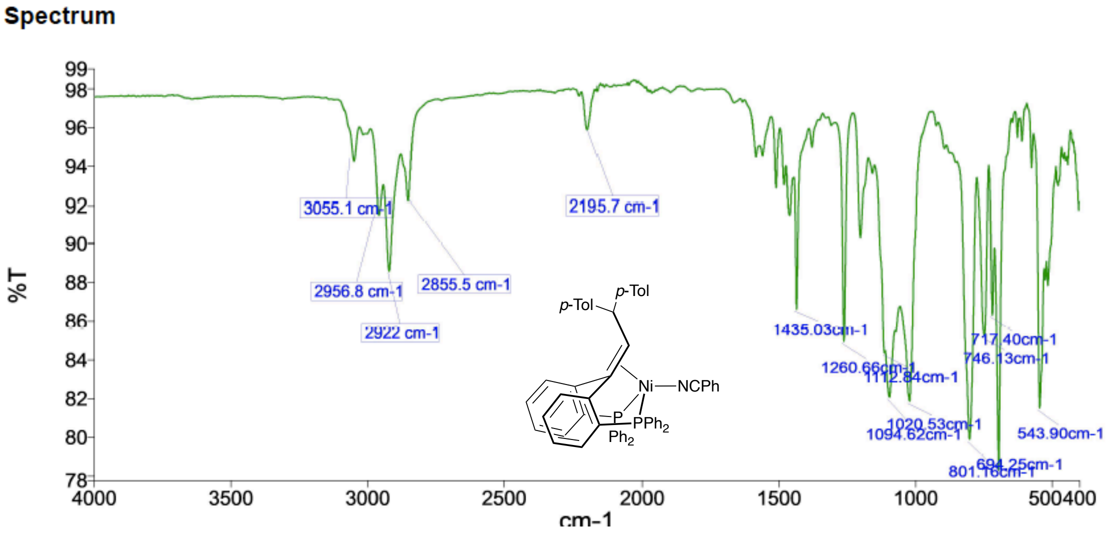

Figure S74. IR complex **8**.

4. X-ray crystal structure determinations

Deposition Numbers 2100133, 2100134, 2100135, 2100136, 2100137, 2100138 and 2100139 contain the supplementary crystallographic data for this paper. These data are provided free of charge by the joint Cambridge Crystallographic Data Centre and Fachinformationszentrum Karlsruhe Access Structures service www.ccdc.cam.ac.uk/structures

X-ray crystal structure determination of (^{Ph}bppe^{H,H})Ni(CH$_2$CHPhF) (2):

$C_{46}H_{37}FNiP_2$, Fw = 729.40, red block, 0.31 × 0.28 × 0.12 mm^3, monoclinic, $P2_1/c$ (no. 14), a = 13.9141(2), b = 12.66731(19), c = 20.9927(4) Å, β = 102.259(1) °, V = 3615.69(11) Å^3, Z = 4, D_x = 1.340 g/cm^3, μ = 0.66 mm^{-1}. The diffraction experiment was performed on a Bruker Kappa ApexII diffractometer with sealed tube and Triumph monochromator (λ = 0.71073 Å) at a temperature of 150(2) K up to a resolution of $(\sin \theta/\lambda)_{max}$ = 0.69 Å^{-1}. Intensity integration was performed with the Eval15 software.[7] A multi-scan absorption correction and scaling was performed with SADABS[8] (correction range 0.69-0.75). A total of 100578 reflections was measured, 10138 reflections were unique (R_{int} = 0.035), 8872 reflections were observed [I>2σ(I)]. The structure was solved with Patterson superposition methods using SHELXT.[9] Structure refinement was performed with SHELXL-2018[10] on F^2 of all reflections. Non-hydrogen atoms were refined freely with anisotropic displacement parameters. All hydrogen atoms were located in difference Fourier maps. Hydrogen atoms at C38, C39 and C40 were refined freely with isotropic displacement parameters. All other hydrogen atoms were refined with a riding model. 471 Parameters were refined with no restraints. R1/wR2 [I > 2σ(I)]: 0.0296 / 0.0782. R1/wR2 [all refl.]: 0.0357 / 0.0810. S = 1.043. Residual electron density between -0.33 and 0.75 e/Å^3. Geometry calculations and checking for higher symmetry was performed with the PLATON program.[11]

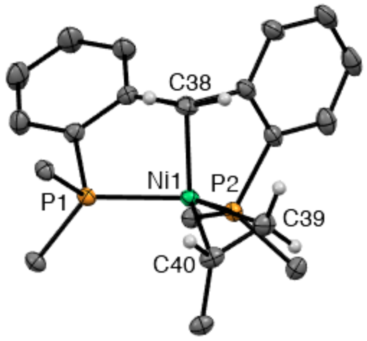

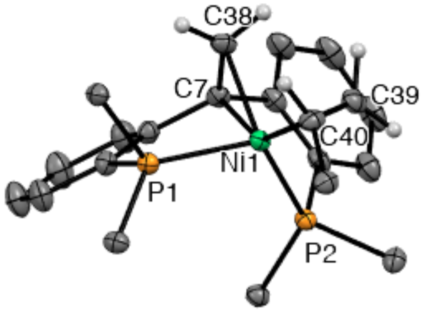

Figure S75. Molecular structure of **2**. Displacement ellipsoids are drawn at the 50% probability level. Hydrogen atoms and the phenyl rings of the phosphines and, p-Fluorostyrene are omitted for clarity. Selected bond lenghts (Å) and angles (°): Ni1-P1 2.2084(3), Ni1-P2 2.2419(3), Ni1-C7 2.0950(12), Ni1-C38 2.0847(12), Ni1-C39 2.0318(13), Ni1-C40 2.0783(12), C7-C38 1.3945(18), C39-C40 1.402(2), P2-Ni1-P2 113.851(13), P1-Ni1-C39 142.90(5), P2-Ni1-C39 97.51(4), P1-Ni1-C7 87.32(3), P2-Ni1-C7 81.65(4), C7-Ni1-C38 38.98(5), C39-Ni1-C40 39.87(5).

X-ray crystal structure determination of (^{Ph}bppe^{H,H})Ni(N$_2$Cptol$_2$) (3).

$C_{53}H_{44}N_2NiP_2$, Fw = 829.55, dark red plate, 0.22 × 0.12 × 0.03 mm^3, monoclinic, $P2_1/n$ (no. 14), a = 9.4409(3), b = 20.5696(5), c = 21.7964(5) Å, β = 93.807(1) °, V = 4223.40(19) Å^3, Z = 4, D_x = 1.305 g/cm^3, μ = 0.58 mm^{-1}. The diffraction experiment was performed on a Bruker Kappa ApexII diffractometer with sealed tube and Triumph monochromator (λ = 0.71073 Å) at a temperature of 150(2) K up to a resolution of $(\sin \theta/\lambda)_{max}$ = 0.65 Å^{-1}. Intensity integration was performed with the Eval15 software.[7] A multi-scan absorption correction and scaling was performed with SADABS[8] (correction range 0.66-0.75). A total of 47985 reflections was measured, 9666 reflections were unique (R_{int} = 0.049), 6951 reflections were observed [I>2σ(I)]. The structure was solved with Patterson superposition methods using SHELXT.[9] Structure refinement was performed with SHELXL-2018[10] on F^2 of all reflections. Non-hydrogen atoms were refined freely with anisotropic displacement parameters. The Ni atom was disordered over two positions (occupancy 97.4%:2.6%). The distance between the disorder positions is 0.839(12) Å. All hydrogen atoms were located in difference Fourier maps. Hydrogen atoms at C38 were refined freely with isotropic displacement parameters. All other hydrogen atoms were refined with a riding model. 537 Parameters were refined with no restraints. R1/wR2 [I > 2σ(I)]: 0.0432 / 0.1026. R1/wR2 [all refl.]: 0.0708 / 0.1137. S = 1.034. Residual electron density between -0.26 and 0.47 e/Å^3. Geometry calculations and checking for higher symmetry was performed with the PLATON program.[11]

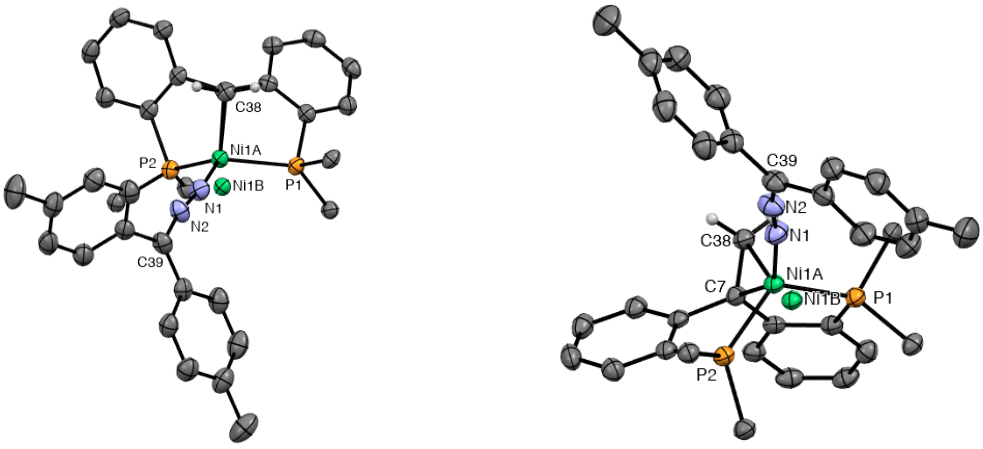

Figure S76. Molecular structure of **3**. Displacement ellipsoids are drawn at the 50% probability level. Hydrogen atoms and the phenyl rings of the phosphines are omitted for clarity. Selected bond lenghts (Å) and angles (°): Ni1A-P1 2.2203(6), Ni1A-P2 2.2198(6), Ni1B-P1 2.202(11), Ni1B-P2 2.123(11), Ni1A-C7 2.078(2), Ni1A-C38 2.040(2), C7-C38 1.395(3), Ni1A-N1 1.799(2), N1-N2 1.157(3), N2-C39 1.312(3), P2-Ni1A-N1 109.35(7), P1-Ni1A-N1 113.06(7), C7-Ni1A-C38 39.59(8), P1-Ni1A-C7 88.83(6), P2-Ni1A-C7 85.31(6).

X-ray crystal structure determination of Nickelacyclobutane (4):

$C_{53}H_{44}NiP_2 \cdot 0.5(C_4H_{10}O)$, Fw = 838.59, dark red needle, 0.34 × 0.10 × 0.06 mm^3, monoclinic, $P2_1/c$ (no. 14), a = 15.8842(4), b = 10.6927(3), c = 26.4835(7) Å, β = 106.116(2) °, V = 4321.3(2) Å^3, Z = 4, D_x = 1.289 g/cm^3, μ = 0.56 mm^{-1}. The diffraction experiment was performed on a Bruker Kappa ApexII diffractometer with sealed tube and Triumph monochromator (λ = 0.71073 Å) at a temperature of 150(2) K up to a resolution of $(\sin \theta/\lambda)_{max}$ = 0.65 Å^{-1}. The crystal was cracked into two fragments which are related by a rotation of 5.7° about an arbitrary axis. Consequently, two orientation matrices were used for the intensity integration with the Eval15 software.[7] Only the non-overlapping reflections of the major fragment were used for structure solution and refinement. A numerical absorption correction and scaling was performed with SADABS[8] (correction range 0.76-1.00). A total of 62246 reflections was measured, 9883 reflections were unique (R_{int} = 0.070), 7050 reflections were observed [$I>2\sigma(I)$]. The structure was solved with Patterson superposition methods using SHELXT.[9] Structure refinement was performed with SHELXL-2018[10] on F^2 of all reflections. Non-hydrogen atoms were refined freely with anisotropic displacement parameters. The diethyl ether molecule was disordered on an inversion center. All hydrogen atoms of the metal complex were located in difference Fourier maps. Hydrogen atoms of the diethyl ether were introduced in calculated positions. All hydrogen atoms were refined with a riding model. 552 Parameters were refined with 58 restraints (distances, angles and displacement parameters of the disordered diethyl ether). R1/wR2 [$I > 2\sigma(I)$]: 0.0402 / 0.0861. R1/wR2 [all refl.]: 0.0698 / 0.0959. S = 1.032. Residual electron density between -0.34 and 0.41 e/Å^3. Geometry calculations and checking for higher symmetry was performed with the PLATON program.[11]

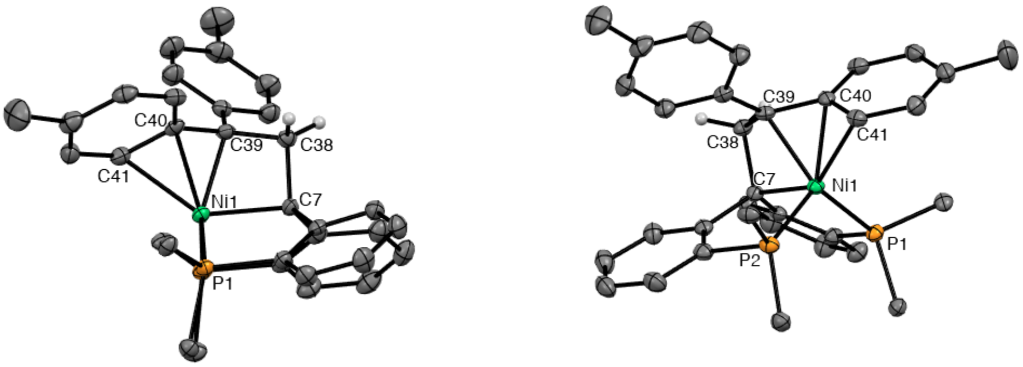

Figure S77. Molecular structure of **4**. Displacement ellipsoids are drawn at the 50% probability level. Solvent molecules, most H atoms and phenyl rings from the phosphines are omitted for clarity. Selected bond lenghts (Å) and angles (°): Ni1-C7 2.011(2), C7-C38 1.541(3), C38-C39 1.524(3), C39-C40 1.465(3), C40-C41 1.428(3), Ni1-C39 2.058(2), Ni1-C40 2.049(2), Ni1-C41 2.240(2), Ni-C38 2.577(2), C7-Ni1-C39 70.41(8), C7-C38-C39 99.88(16), Ni1-C7-C38 92.08(12), P2-Ni1-P1 112.34(2), P1-Ni1-C39 139.95(6), P2-Ni1-C39 102.89(6).

X-ray crystal structure determination of Cyclopropane Ni(CO)$_2$ (5):

$C_{55}H_{44}NiO_2P_2$ + disordered solvent, Fw = 857.55[*], yellow block, 0.34 × 0.25 × 0.08 mm^3, triclinic, $P\bar{1}$ (no. 2), a = 12.2453(3), b = 15.7459(4), c = 15.8736(4) Å, α = 87.726(1), β = 72.699(1), γ = 89.699(1) °, V = 2919.78(12) Å^3, Z = 2, D_x = 0.975 g/cm^3[*], μ = 0.42 mm^{-1}[*]. The diffraction experiment was performed on a Bruker Kappa ApexII diffractometer with sealed tube and Triumph monochromator (λ = 0.71073 Å) at a temperature of 150(2) K up to a resolution of $(\sin \theta/\lambda)_{max}$ = 0.65 Å^{-1}. Intensity integration was performed with the Eval15 software.[7] A multi-scan absorption correction and scaling was performed with SADABS[8] (correction range 0.70-0.75). A total of 64781 reflections was measured, 13415 reflections were unique (R_{int} = 0.030), 11285 reflections were observed [I>2σ(I)]. The structure was solved with Patterson superposition methods using SHELXT.[9] Structure refinement was performed with SHELXL-2018[10] on F^2 of all reflections. The crystal structure contains large voids (1029 Å^3 / unit cell), filled with disordered toluene and hexane solvent molecules. Their contribution to the structure factors was secured by back-Fourier transformation using the SQUEEZE routine[12] of the program PLATON[11], amounting to 251 electrons / unit cell. Non-hydrogen atoms were refined freely with anisotropic displacement parameters. All hydrogen atoms were introduced in calculated positions and refined with a riding model. 543 Parameters were refined with no restraints. R1/wR2 [I > 2σ(I)]: 0.0288 / 0.0750. R1/wR2 [all refl.]: 0.0362 / 0.0781. S = 1.048. Residual electron density between -0.23 and 0.36 e/Å^3. Geometry calculations and checking for higher symmetry was performed with the PLATON program.[11]

[*] Derived values do not contain the contribution of the disordered solvent molecules.

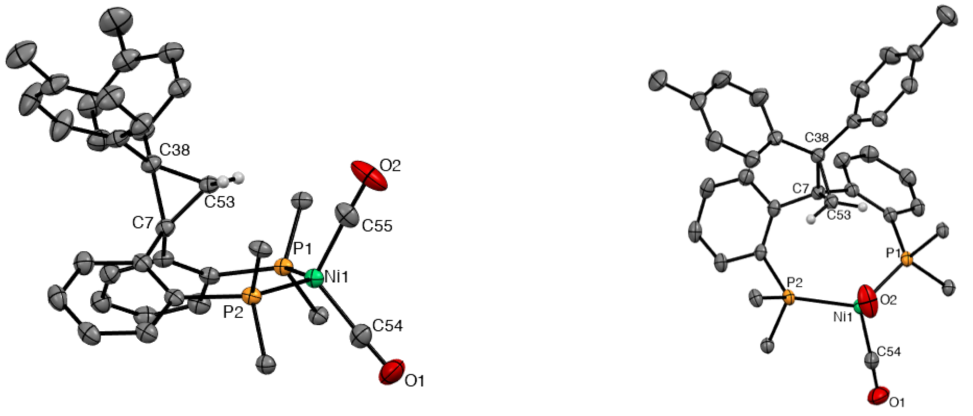

Figure S78. Molecular structure of **5**. Displacement ellipsoids are drawn at the 50% probability level. Solvent molecules, most H atoms and phenyl rings from the phosphines are omitted for clarity. Selected bond lenghts (Å) and angles (°): Ni1-C55 1.7728(14), Ni1-C54 1.7852(14), Ni1-P1 2.2203(3), Ni1-P2 2.2298(3), C7-C38 1.5723(16), C38-C53 1.5026(16), C53-C7 1.5076(16), C7-C38-C53 58.67(7), C7-C53-C38 62.97(8), C38-C7-C53 58.36(7).

X-ray crystal structure determination of (^{Ph}PCHP^{Ph})Ni(CH$_2$CN) (6):

$C_{39}H_{31}NNiP_2$, Fw = 634.30, yellow needle, 0.31 × 0.11 × 0.09 mm^3, monoclinic, $P2_1/c$ (no. 14), a = 9.9208(5), b = 31.9470(17), c = 10.6826(5) Å, β = 114.140(3) °, V = 3089.6(3) Å^3, Z = 4, D_x = 1.364 g/cm^3, μ = 0.76 mm^{-1}. The diffraction experiment was performed on a Bruker Kappa ApexII diffractometer with sealed tube and Triumph monochromator (λ = 0.71073 Å) at a temperature of 150(2) K up to a resolution of $(\sin \theta/\lambda)_{max}$ = 0.61 Å^{-1}. The weakly diffracting crystal was cracked into several fragments. Only one orientation matrix was used for the intensity integration of the major fragment with the Eval15 software.[7] A multi-scan absorption correction and scaling was performed with SADABS[8] (correction range 0.35-0.75). A total of 28135 reflections was measured, 5723 reflections were unique (R_{int} = 0.141), 3380 reflections were observed [I>2σ(I)]. The structure was solved with Patterson superposition methods using SHELXT.[9] Structure refinement was performed with SHELXL-2018[10] on F^2 of all reflections. Non-hydrogen atoms were refined freely with anisotropic displacement parameters. All hydrogen atoms were located in difference Fourier maps and refined with a riding model. 388 Parameters were refined with no restraints. R1/wR2 [I > 2σ(I)]: 0.0611 / 0.1292. R1/wR2 [all refl.]: 0.1258 / 0.1560. S = 0.993. Residual electron density between -0.42 and 0.74 e/Å^3. Geometry calculations and checking for higher symmetry was performed with the PLATON program.[11]

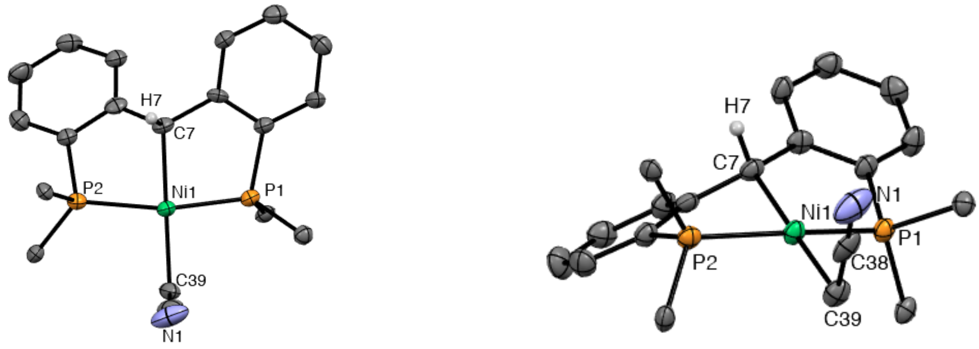

Figure S79. Molecular structure of **6**. Displacement ellipsoids are drawn at the 50% probability level. Solvent molecules, most H atoms and phenyl rings from the phosphines are omitted for clarity. Selected bond lenghts (Å) and angles (°): Ni1-C7 2.015(5), Ni1-C39 1.997(5), Ni1-P1 2.1356(14), Ni1-P2 2.1858(14), P1-Ni1-P2 164.99(6), C7-Ni1-C39 178.8(2).

X-ray crystal structure determination of [(^{Ph}bppe^{H,CHptol2})Ni]$_2$N$_2$ (7):

$C_{106}H_{88}N_2Ni_2P_4 \cdot C_6H_{14} \cdot C_4H_8O$, Fw = 1789.35, red plate, 0.13 × 0.11 × 0.04 mm^3, triclinic, P $\overline{1}$ (no. 2), a = 13.3390(4), b = 18.2056(7), c = 21.1768(7) Å, α = 104.795(2), β = 99.730(2), γ = 102.246(2) °, V = 4721.5(3) Å^3, Z = 2, D_x = 1.259 g/cm^3, μ = 0.52 mm^{-1}. The diffraction experiment was performed on a Bruker Kappa ApexII diffractometer with sealed tube and Triumph monochromator (λ = 0.71073 Å) at a temperature of 150(2) K up to a resolution of $(\sin \theta/\lambda)_{max}$ = 0.61 Å^{-1}. The crystal was cracked into two fragments which are related by a rotation of 2.4° about an arbitrary axis. Consequently, two orientation matrices were used for the intensity integration with the Eval15 software.[7] The integration results were written in HKLF5 format.[13] A multi-scan absorption correction and scaling was performed with TWINABS[8] (correction range 0.58-0.75). A total of 93256 reflections was measured, 17590 reflections were unique (R_{int} = 0.093), 11470 reflections were observed [$I>2\sigma(I)$]. The structure was solved with Patterson superposition methods using SHELXT.[9] Structure refinement was performed with SHELXL-2018[10] on F^2 of all reflections. Non-hydrogen atoms were refined freely with anisotropic displacement parameters. The hexane and THF molecules were refined with disorder models. All hydrogen atoms of the metal complex were located in difference Fourier maps. Hydrogen atoms of the solvent molecules were introduced in calculated positions. All hydrogen atoms were refined with a riding model. 1196 Parameters were refined with 276 restraints (distances, angles and displacement parameters of the disordered solvent molecules). R1/wR2 [$I > 2\sigma(I)$]: 0.0635 / 0.1502. R1/wR2 [all refl.]: 0.1105 / 0.1746. S = 1.024. Batch scale factor BASF=0.391(3). Residual electron density between -0.63 and 1.54 e/Å^3. Geometry calculations and checking for higher symmetry was performed with the PLATON program.[11]

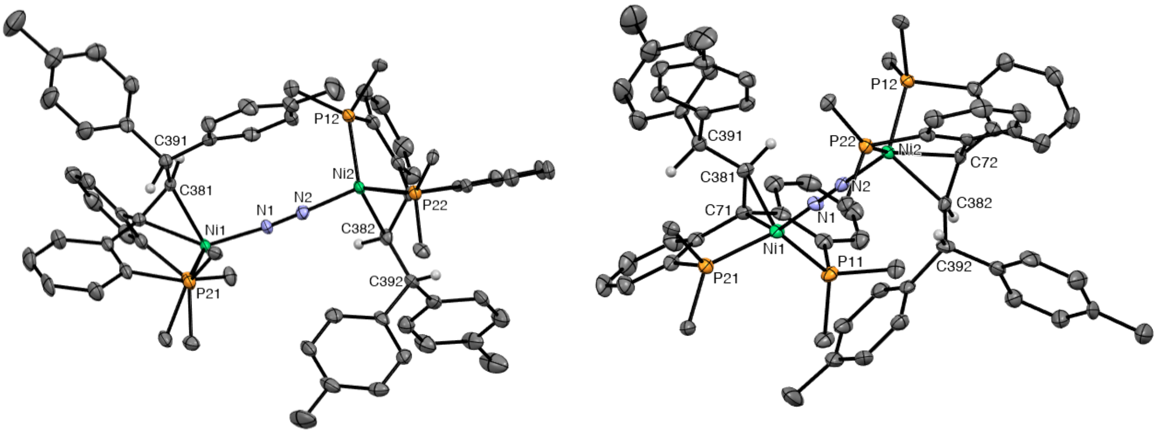

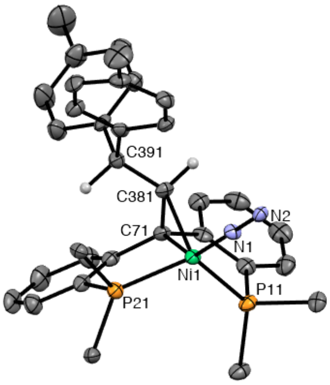

Figure S80. Molecular structure of **7.** Displacement ellipsoids are drawn at the 50% probability level. Solvent molecules, most H atoms and phenyl rings from the phosphines are omitted for clarity. Selected bond lenghts (Å) and angles (°): Ni1-C71 2.040(4), C71-C381 1.421(5), Ni1-C381 2.076(4), Ni1-N1 1.850(3), Ni1-P21 2.2505(12), Ni1-P11 2.2029(12), N1-N2 1.127(4),C72-C382 1.415(6), Ni2-C72 2.039(4), Ni2-C382 2.091(4), Ni2-P22 2.2447(12), Ni2-P12 2.2131(11), Ni2-N2 1.846(3), Ni1-N1-N2 172.1(3), Ni2-N2-N1 176.2(3), C71-Ni1-C381 40.39(15), P21-Ni1-C71 89.39(12), P11-Ni1-C71 86.88(12), P11-Ni1-N1 103.91(10), P21-Ni1-N2 115.69(11), Ni2-N2-N1 176.2(3), P12-Ni2-C382 117.58(11), P22-Ni2-C72 89.55(11), P12-Ni2-C72 86.17(11), P12-Ni2-N2 109.60(10), P22-Ni2-N2 113.40(10)

X-ray crystal structure determination of (^{Ph}bppe^{H,CHptol2})Ni(PhCN) (8):

$C_{60}H_{49}NNiP_2 \cdot 0.5(C_7H_8)$, Fw = 950.72, red needle, 0.30 × 0.13 × 0.05 mm^3, triclinic, P $\overline{1}$ (no. 2), a = 10.6215(4), b = 11.8844(4), c = 21.1448(7) Å, α = 104.793(1), β = 91.312(1), γ = 107.622(2) °, V = 2444.98(15) Å^3, Z = 2, D_x = 1.291 g/cm^3, μ = 0.51 mm^{-1}. The diffraction experiment was performed on a Bruker Kappa ApexII diffractometer with sealed tube and Triumph monochromator (λ = 0.71073 Å) at a temperature of 150(2) K up to a resolution of $(\sin \theta/\lambda)_{max}$ = 0.61 Å^{-1}. A split-mosaic model was used for the profile prediction with the Eval15 software.[7] A multi-scan absorption correction and scaling was performed with SADABS[8] (correction range 0.65-0.75). A total of 37499 reflections was measured, 9101 reflections were unique (R_{int} = 0.069), 6089 reflections were observed [I>2σ(I)]. The structure was solved with Patterson superposition methods using SHELXT.[10] Structure refinement was performed with SHELXL-2018[10] on F^2 of all reflections. Non-hydrogen atoms were refined freely with anisotropic displacement parameters. All hydrogen atoms of the metal complex were located in difference Fourier maps. Hydrogen atoms of the toluene molecule were introduced in calculated positions. Hydrogen atoms at C38 and C39 were refined freely with isotropic displacement parameters. All other hydrogen atoms were refined with a riding model. 650 Parameters were refined with 123 restraints (distances, angles, molecular flatness and displacement parameters of the toluene molecule). R1/wR2 [I > 2σ(I)]: 0.0502 / 0.1141. R1/wR2 [all refl.]: 0.0907 / 0.1311. S = 1.015. Residual electron density between -0.26 and 0.59 e/Å^3. Geometry calculations and checking for higher symmetry was performed with the PLATON program.[11]

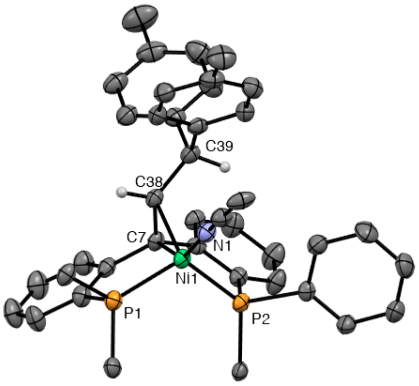

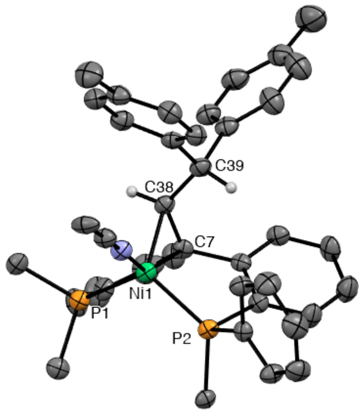

Figure S81. Molecular structure of **8**. Displacement ellipsoids are drawn at the 50% probability level. Solvent molecules, most H atoms and phenyl rings from the phosphines are omitted for clarity. Selected bond lenghts (Å) and angles (°): Ni1-P1 2.1845(9), Ni1-P2 2.1718(9), Ni1-N1 1.878(3), Ni1-C7 1.027(3), Ni1-C38 2.039(3), C38-C39 1.516(5), P1-Ni1-P2 114.58(4), P1-Ni1-C38 101.50(10), P2-Ni1-C38 117.47(10), C7-Ni1-C38 40.72(12).

5. DFT studies

5.1 General information

DFT calculations were performed using the Gaussian 16 software package version C.01.[14] Geometry optimizations were carried out in vacuum at the B3LYP-GDB3J/6-31g(d,p) level of theory on all atoms. Frequency analyses on all stationary points were used to ensure that they are minima (no imaginary frequency) or transition states (one imaginary frequency). Transition states were calculated using the QST3 (synchronous transit-guided quasi-Newton number 3) method or using the opt=TS (Berny algorithm) keyword. The guess structure proposed for each TS calculation was based on the results of relaxed potential energy surface scans (PES). ΔGº was calculated by single point calculation at B3LYP-GDB3J/def2TZVP level of theory adjusting the value with the thermal correction obtained at the B3LYP-GDB3J/6-31g(d,p) level of theory with temperature 298.15 K and pressure 1 atmosphere.

5.2 Additional comments per pathway

5.2.1) Nickelacyclobutane formation

Nickelacyclobutane formation is proposed to proceed via a nickel carbene intermediate (Figure S82). Nickel carbenes reported in literature are typically synthetized from the nickel diazo adduct in presence of Lewis acids or a light source to help the extrusion of nitrogen.[15,16] In our research, the alkene backbone is coordinated in the transition state what could suggest an important role of the alkene in the formation of the nickel carbene.

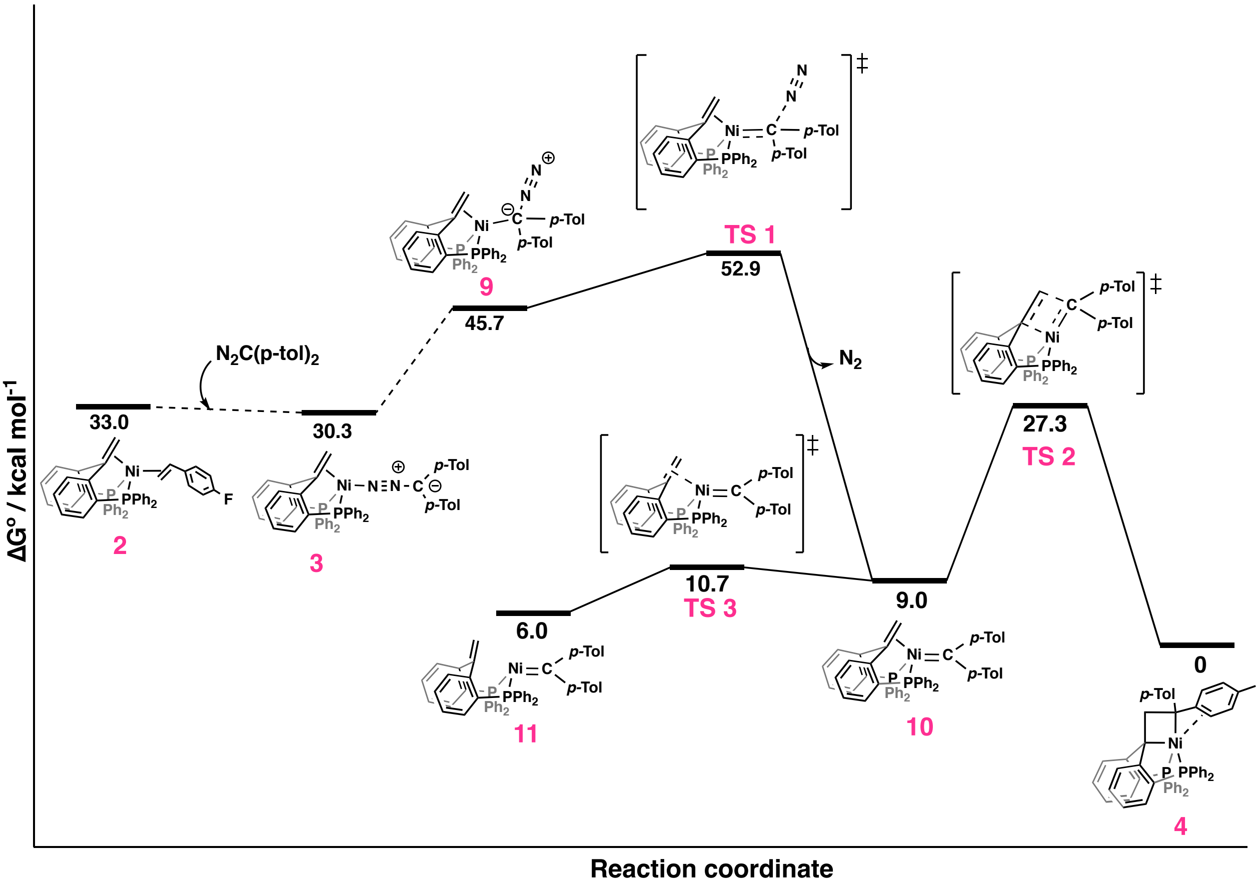

Figure S82. Calculated relative Gibbs free energies for the formation of nickelacyclobutane **4** via nickel carbene.

Calculations showed that intermediates **3** and **9** without the alkene backbone coordinated to the nickel center are slightly higher in energy in comparison with the tetra coordinated intermediates (Figure S83). Nickel carbene intermediate **4** is more stable without the alkene backbone coordination, this is in good agreement with the observations of Hillhouse where the three-coordination geometry is a stable configuration for a Nickel carbene.[15,16]

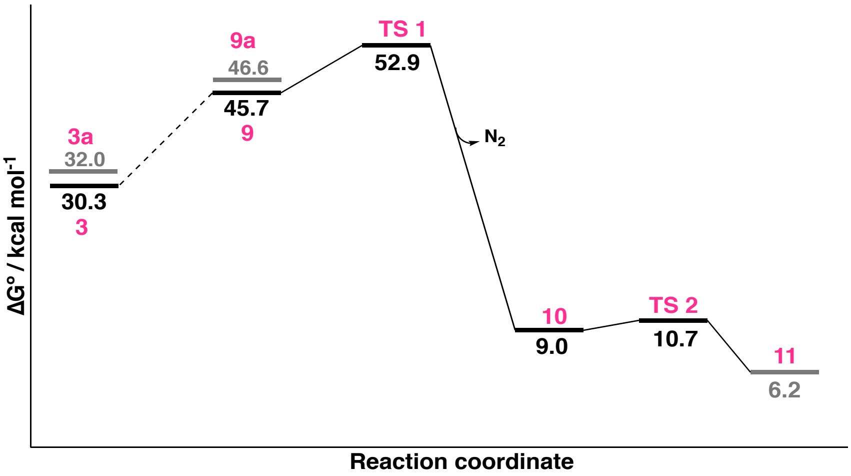

Figure S83. Comparison of the calculated relative Gibbs free energies for **2** to **4**. In black: pathway with C=C backbone coordinated, in gray: pathway without C=C coordination.

Additionally, the pathway of formation of the nickelacyclobutane via nucleophilic attack of the diazoalkane on the olefin was calculated (Figure S84). This route has been proposed for the formation of carbenes but not computed before to the best of our knowledge.[17] Attack of the diazoalkane on the alkene backbone proceeds through a TS of 24.8 Kcal/mol and leads to the formation of a six-membered ring intermediate **15**. This intermediate has no significant energy difference with respect with the diazoalkane adduct. To trigger the extrusion of nitrogen, one of the C=C bond from a tolyl group coordinates to nickel leading to an eighteen-electron complex with an energy change of 36.1 Kcal/mol, what it is too high for a process at room temperature. The following transition state leading to the nickelacyclobutane is located 17.9 Kcal/mol higher in energy. The resulting nickelacyclobutane is a pentacoordinated complex with a nitrogen molecule coordinated side-on in apical position that is 13.1 Kcal/mol lower in energy than the initial styrene complex. The released of nitrogen will lead to the nickelacyclobutane **4** that is a more stable structure. This pathway is not feasible at room temperature and the nickel carbene pathway is more thermodynamically available.

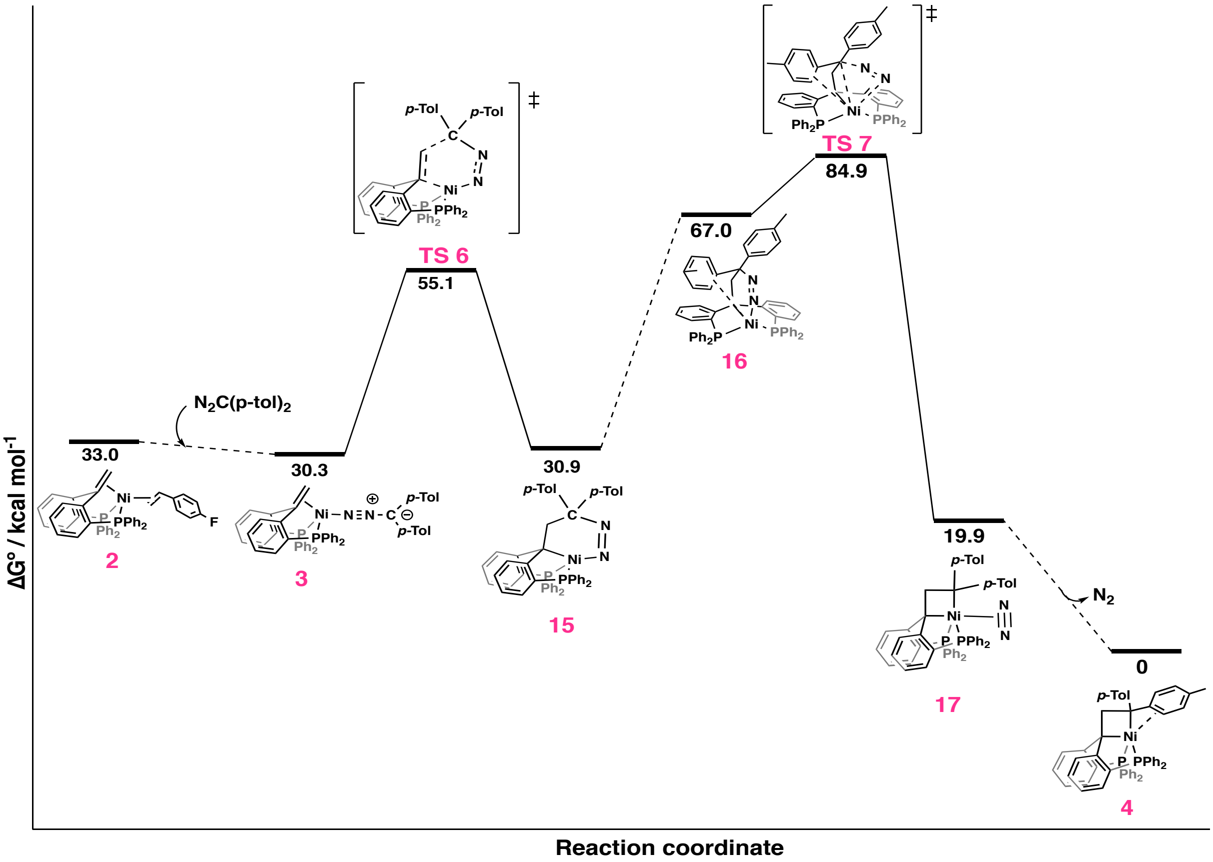

Figure S84. Calculated relative Gibbs free energies for the formation of nickelacyclobutane 4 via nucleophilic attack of the diazoalkane.

5.2.2) β-elimination

β-elimination is a challenging pathway for some metallacycles giving the restriction to achieve a syn coplanar angle with the H_β. A relaxed PES scan of the H_β-P bond of the nickelacyclobutane **4** showed the right angle could be achieved by the decoordination of one of the phosphines (Figure S85). This process requires a change of energy of 20.4 Kcal/mol higher being followed by a TS with a 35.3 Kcal/mol energy change. The activation energy for this process is not accessible at room temperature.

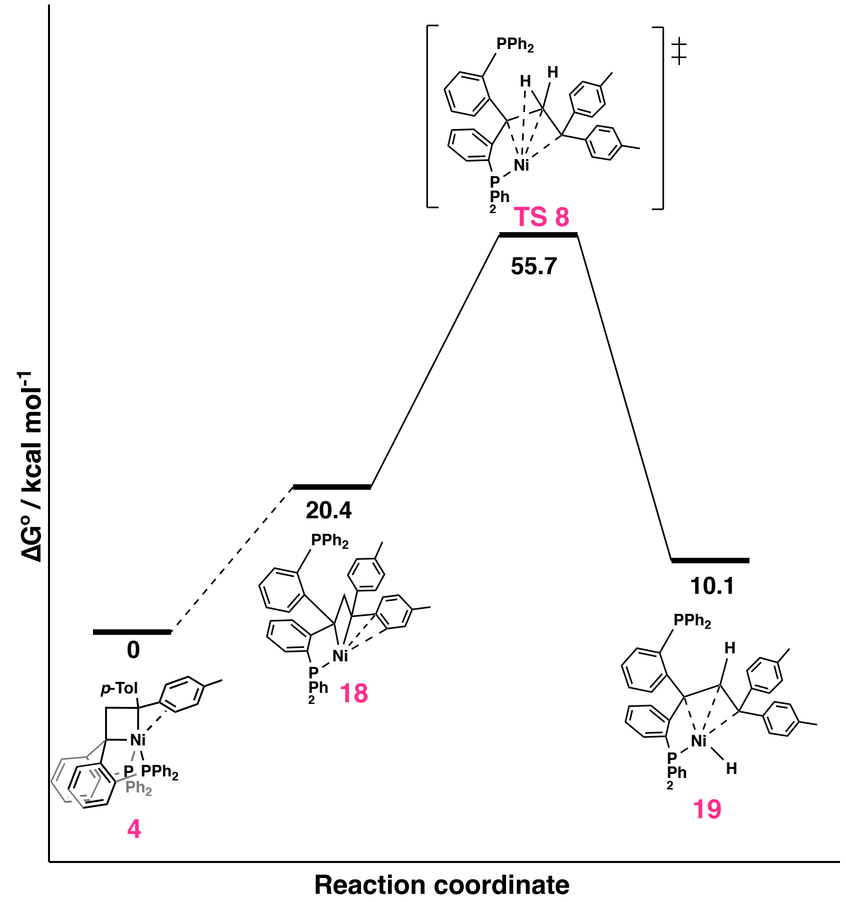

Figure S85. Calculated relative Gibbs free energies for β-elimination.

5.2.3) Cyclopropanation

For the cyclopropanation pathway in presence of CO as presented in the main text, it is proposed that the formation of the cyclopropane takes place first followed by the addition of another molecule of CO. After this, the rearrangement leading to a more stable structure occurs (Figure S86). However, a pathway where decoordination of phosphines takes place allowing the coordination of a second molecule of CO to the nickelacyclobutane can not be discarded.

In comparison, cyclopropanation in presence of MeCN was calculated (Figure S87). Results are consistent with the observation that this process does not to take place at room temperature: the TS is located 28.5 Kcal/mol higher in energy than **4-MeCN** and the cyclopropanation product is found to be 15.3 Kcal/mol higher in comparison with the initial nickelacyclobutane. Additionally, the pathway was calculated without the presence of an exogenous coligand (Figure S88). The transition state is higher in energy by 30.5 Kcal/mol leading to a cyclopropane product that is located at 30.8 Kcal/mol energy, making this route not feasible.

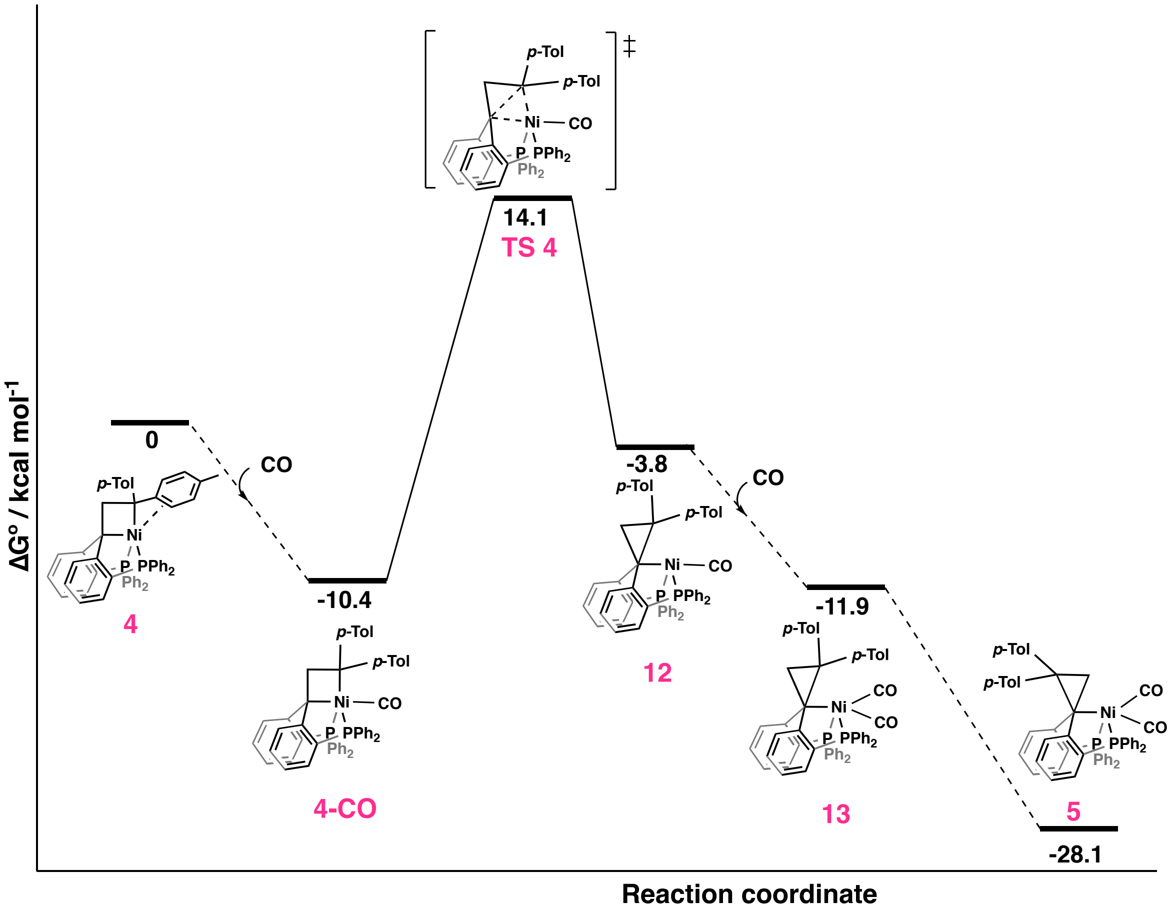

Figure S86. Calculated relative Gibbs free energies for the cyclopropanation with CO.

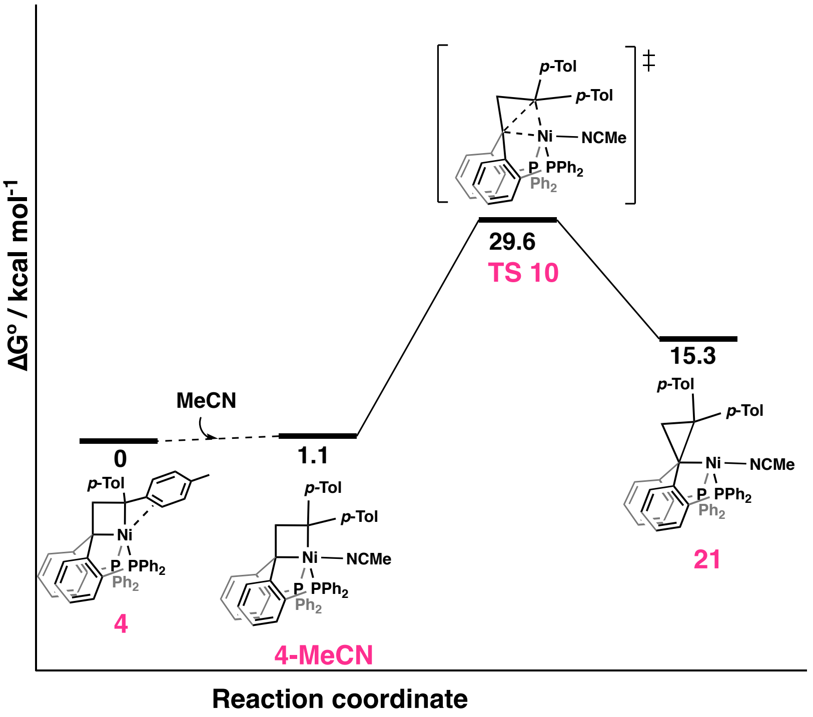

Figure S87. Calculated relative Gibbs free energies for the cyclopropanation with MeCN.

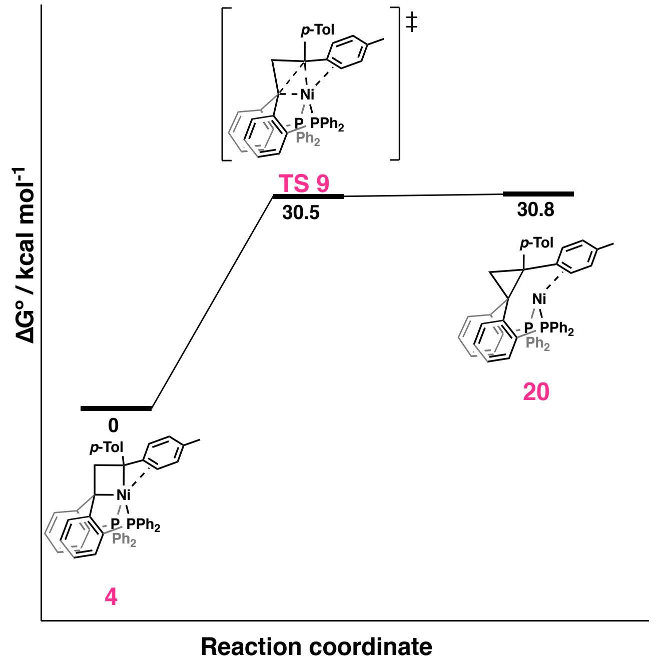

Figure S88. Calculated relative Gibbs free energies for the cyclopropanation from nickelacyclobutane **4**.

5.2.4) Cycloreversion

Cycloreversion in presence of MeCN gives place to ($PC_{carbene}P$)Ni(MeCN) that subsequently reacts with MeCN via C-H activation to lead to a more stable product at -13.0 Kcal/mol (Figure S89). According to the calculations, cycloreversion can also take place with a CO coligand in the apical position (Figure S90). The TS to overcome is 24.4 Kcal/more energy change what is comparable to the cyclopropanation TS (see section 5.2.3). The product being ($PC_{carbene}P$)Ni(CO) is less stable than the cyclopropanation product, but this route may take place in the absence of an excess concentration of CO (see SI section 2.2). Interestingly, as suggested by the reported literature,[18–20] metathesis is thermodynamically allowed for this pentacoordinated nickelacyclobutane with a TS energy to overcome of 24.4 Kcal/mol (Figure S91). However, the product is higher in energy by 13.6 Kcal/mol in comparison with the initial nickelacyclobutane.

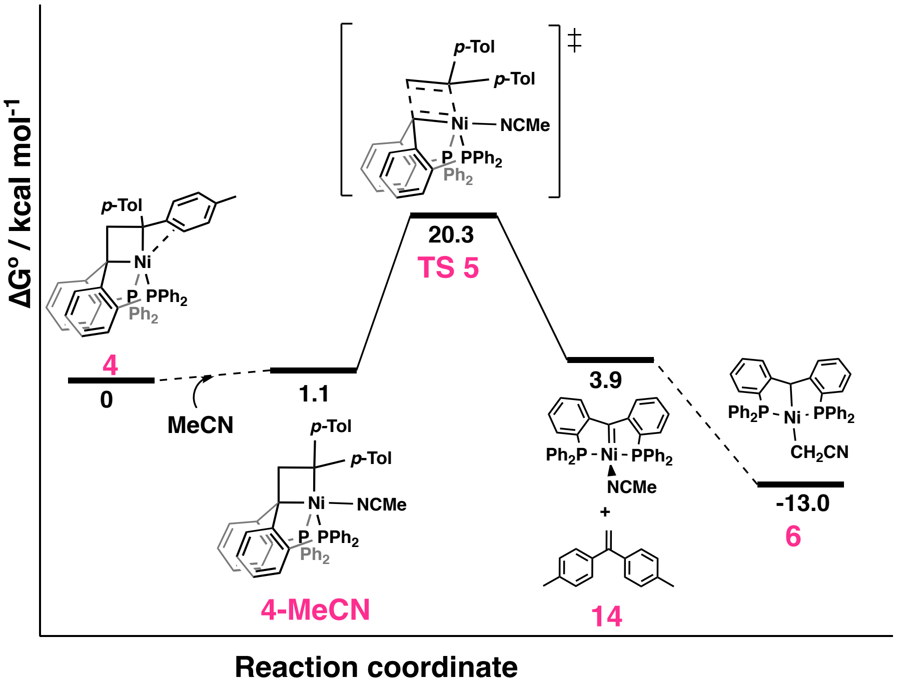

Figure S89. Calculated relative Gibbs free energies for the metathesis in MeCN.

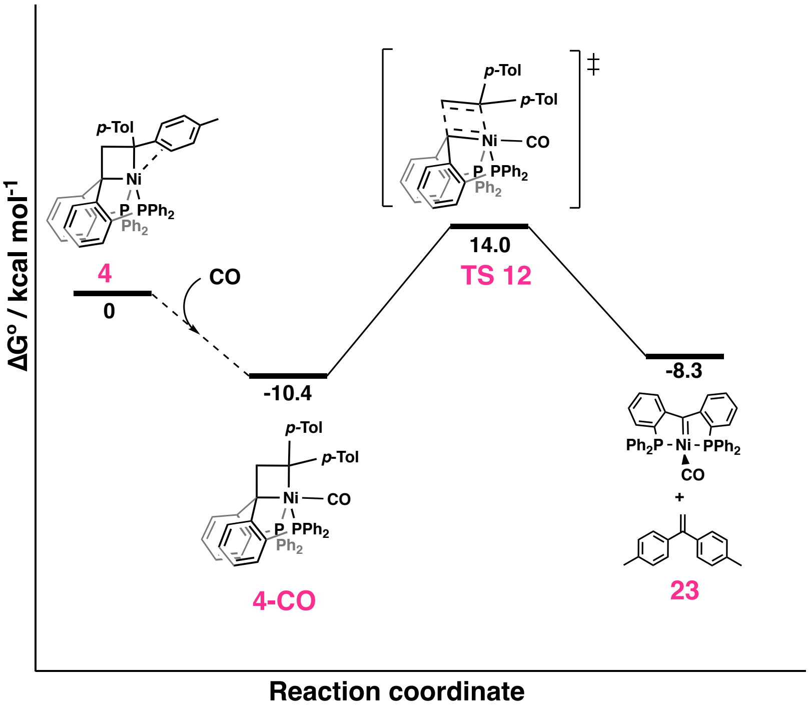

Figure S90. Calculated relative Gibbs free energies for the metathesis with CO.

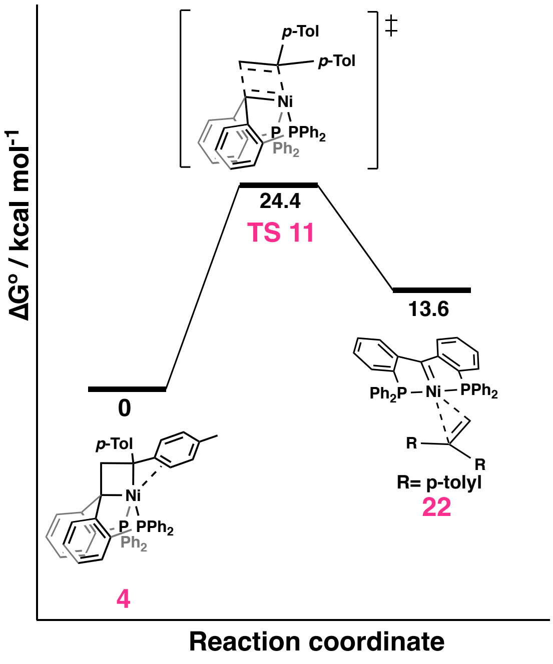

Figure S91. Calculated relative Gibbs free energies for the metathesis from nickelacyclobutane 4.

5.2.5) Additional comments

Important aspect to consider about the Gibbs free energies is the excess of some reactants in the reaction mixture (MeCN and CO). This would lead to an adjustment of the energy base on the concentration, for example for cycloreversion with MeCN:

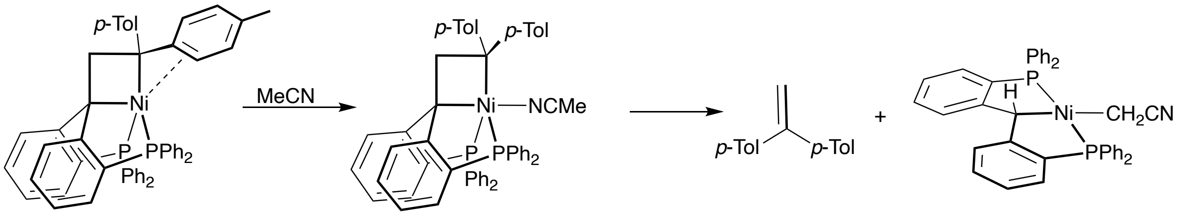

An adjustment of the energies could be made as follows:

$$\Delta G = \Delta G° + RTIn\frac{[P]}{[R]}$$

Where [R] is the concentration of the reactants and [P] of the products. Therefore, taking MeCN as solvent being 5000 times more abundant (according to experimental procedure):

$$\Delta G = \Delta G° + \left(1.99x10^{-3}\ \frac{Kcal}{K.mol}\right)(298.15\ K)In\frac{[1]}{[5000]}$$

$$\Delta G = \Delta G° - \left(5.05\ \frac{Kcal}{mol}\right)$$

The process is more thermodynamically favorable towards the metathesis products.

5.3 Cartesian coordinates

Complex 2

Energy: -4066.53233 Hartree

Ni	0.21979700	-0.19553500	-1.34351000
P	-1.44747900	-0.94195100	-0.09929500
P	1.86269900	0.35261700	0.05893100
F	-2.75286900	5.42715000	0.49492500
C	-0.59292300	-2.36105600	0.68922400
C	-0.99967900	-2.99454900	1.86609000
C	-0.25999500	-4.04723000	2.40117000
C	0.88989300	-4.48189600	1.74373600
C	1.30943100	-3.85010800	0.57561800
C	0.58795100	-2.78021100	0.02840300
C	1.10193400	-2.04728200	-1.18124600
C	2.59543000	-1.89825800	-1.24049600
C	3.44352400	-2.81499000	-1.86634100
C	4.82884700	-2.63659200	-1.81900100
C	5.37649100	-1.55093800	-1.13385100
C	4.53769800	-0.62263400	-0.51240700
C	3.15353000	-0.78800000	-0.58053700
C	-2.82338100	-1.74619700	-1.01408500
C	-3.84360600	-0.93069600	-1.52878000
C	-4.86839700	-1.47702400	-2.29566700
C	-4.88989400	-2.84852700	-2.56310400
C	-3.88449500	-3.66650200	-2.05083900
C	-2.85693200	-3.12094600	-1.27810500
C	-2.41445800	-0.03874800	1.17278800
C	-2.17886900	1.33028600	1.33929400
C	-2.92337000	2.07661400	2.25351200
C	-3.91308000	1.45637000	3.01454200
C	-4.17813200	0.09581000	2.83597600
C	-3.44742400	-0.64207000	1.90772500
C	2.70569900	1.98367900	0.07598000
C	2.54388600	2.90254200	1.12219800
C	3.05115300	4.19703400	1.01463000
C	3.73121800	4.59535800	-0.13677800

C	3.91367200	3.68353100	-1.17719200
C	3.40492100	2.39018800	-1.07377200
C	1.70076400	-0.11571800	1.82638600
C	0.71368000	0.51377200	2.59776200
C	0.45708300	0.10609700	3.90388200
C	1.17992400	-0.95165400	4.45630200
C	2.16458100	-1.58479200	3.69802800
C	2.42937200	-1.16788900	2.39412200
C	0.33234400	-1.94589900	-2.36108400
C	0.71193300	0.98766300	-2.86908000
C	-0.64696400	1.18976400	-2.54675600
C	-1.16201900	2.36867300	-1.82702500
C	-0.32967600	3.24214200	-1.10029600
C	-0.85466500	4.27592100	-0.33085000
C	-2.23288400	4.45629800	-0.29721300
C	-3.08839900	3.65500100	-1.03991700
C	-2.54425600	2.62291000	-1.80188800
H	-1.87876000	-2.64059000	2.39074500
H	-0.57491300	-4.51612700	3.32812000
H	1.47325300	-5.30311700	2.14981000
H	2.22353500	-4.17338800	0.08972100
H	3.01481000	-3.66339100	-2.39133200
H	5.48153800	-3.34742200	-2.31706600
H	6.45344500	-1.41867200	-1.09563300
H	4.95693000	0.23887600	-0.00280800
H	-3.83711600	0.13190700	-1.31013100
H	-5.65315900	-0.83434900	-2.68373900
H	-5.68864200	-3.27490900	-3.16216700
H	-3.89946900	-4.73474300	-2.24588900
H	-2.08603500	-3.76636600	-0.87144300
H	-1.42734700	1.81790400	0.73184700
H	-2.74415900	3.14320200	2.34549500
H	-4.49422700	2.03352800	3.72749100
H	-4.96719200	-0.38462700	3.40673300
H	-3.70011900	-1.68242300	1.73173600
H	2.02421900	2.60786100	2.02606500
H	2.91761900	4.89431100	1.83644000
H	4.12087900	5.60503300	-0.22020100
H	4.45102900	3.97881100	-2.07328300
H	3.55602300	1.68751700	-1.88578800
H	0.12235500	1.30964200	2.16625500
H	-0.32296900	0.60027500	4.47483100
H	0.97112600	-1.28668200	5.46774800
H	2.72366400	-2.41567400	4.11731300
H	3.18474200	-1.67869200	1.80918000
H	0.84426500	-1.80217500	-3.30763100
H	-0.61727800	-2.46611400	-2.43457400
H	1.43305400	1.76184600	-2.62651300
H	0.96735800	0.41260400	-3.75360000
H	-1.39500600	0.67900600	-3.14998200
H	0.74187500	3.09941800	-1.10947700
H	-0.20759200	4.92775900	0.24578400
H	-4.15763700	3.83081800	-1.00158100
H	-3.20566700	1.98433600	-2.37941100

Complex 3

Energy: -4347.15918 Hartree

Ni	0.54224900	-0.62576500	-1.14888900
P	2.17235600	0.82586000	-0.85470000
P	-0.15808800	-1.70998400	0.63786100

N	-0.87136900	0.31278300	-1.76602500
N	-1.95544700	0.67210800	-1.46590400
C	3.53728600	-0.36361000	-0.58192600
C	4.79012800	0.00423200	-0.07826500
C	5.73931000	-0.96397800	0.24124200
C	5.42696800	-2.31169800	0.06105400
C	4.18483900	-2.68615300	-0.44834600
C	3.22406500	-1.72573600	-0.79573200
C	1.88267100	-2.11005100	-1.35528000
C	1.32003100	-3.42230500	-0.87736800
C	1.71036400	-4.65481500	-1.41226200
C	1.18238600	-5.84331900	-0.90444600
C	0.26095200	-5.81440900	0.14467100
C	-0.14844000	-4.59165900	0.67600500
C	0.37612900	-3.40267300	0.16378400
C	2.43601600	2.07191700	0.46738900
C	2.34796400	3.44883700	0.22312500
C	2.49869900	4.36265600	1.26601100
C	2.74545200	3.91472100	2.56411100
C	2.82475100	2.54306600	2.81622900
C	2.66277500	1.62728300	1.77945100
C	2.60101000	1.74812700	-2.38064100
C	1.56598000	2.43572200	-3.03345200
C	1.82491700	3.16351700	-4.19356500
C	3.11468800	3.19381900	-4.72703200
C	4.14377700	2.49539600	-4.09480600
C	3.89088200	1.77907500	-2.92471300
C	0.66478700	-1.38053200	2.23809000
C	0.18050600	-0.37124900	3.08654900
C	0.88051100	-0.02309400	4.23936600
C	2.08511500	-0.65704000	4.55048700
C	2.58531300	-1.64376200	3.69884900
C	1.87830200	-2.00900400	2.55352400
C	-1.92941000	-1.95502000	1.04555000
C	-2.42113700	-2.14698600	2.34355700
C	-3.78772300	-2.33202700	2.55787900
C	-4.67431700	-2.33261800	1.48133800
C	-4.19139500	-2.16054200	0.18350600
C	-2.82843000	-1.97602200	-0.03172000
C	1.49411400	-1.59400900	-2.61825100
C	-2.88913700	1.30538800	-0.80284300
C	-2.45337300	2.25208600	0.23966900
C	-3.23157400	3.36285000	0.60687000
C	-2.78720200	4.24848000	1.58599100
C	-1.55631600	4.07011400	2.22782500
C	-0.77580600	2.97061000	1.84880300
C	-1.21028600	2.08015500	0.87596200
C	-1.07896200	5.01654300	3.30007600
C	-4.28057000	0.94712200	-1.10538800
C	-5.29275800	1.05339400	-0.13620900
C	-6.58311000	0.60516900	-0.39998100
C	-6.92186400	0.02858600	-1.62976000
C	-5.91062500	-0.08046500	-2.59409300
C	-4.62136600	0.37565100	-2.34449700
C	-8.32890800	-0.43093100	-1.91810800
H	5.00348000	1.05248500	0.10490100
H	6.70370400	-0.67108900	0.64437900
H	6.15084500	-3.07789400	0.32287500
H	3.95398500	-3.73788400	-0.57184800
H	2.41678500	-4.67686200	-2.23664800
H	1.48347400	-6.79375100	-1.33499100

H	-0.15114600	-6.74088000	0.53278500
H	-0.89432500	-4.55631000	1.46396800
H	2.16779500	3.80956700	-0.78328700
H	2.42909500	5.42655700	1.06052300
H	2.87229900	4.62755700	3.37333400
H	3.00563500	2.17819900	3.82201000
H	2.72553500	0.56606100	1.98727500
H	0.55705000	2.38115100	-2.63658100
H	1.01742800	3.69516400	-4.68771300
H	3.31458500	3.75205800	-5.63662500
H	5.14632400	2.50793400	-4.51209800
H	4.69437000	1.23591300	-2.43969700
H	-0.73866000	0.14897200	2.84183600
H	0.49030900	0.75645500	4.88652100
H	2.63260200	-0.38060000	5.44645800
H	3.52661600	-2.13482800	3.92622300
H	2.27567700	-2.77480500	1.89715500
H	-1.74063500	-2.14492600	3.18744500
H	-4.15787200	-2.47195300	3.56931700
H	-5.73932400	-2.45721200	1.65097400
H	-4.87668700	-2.13611500	-0.65682000
H	-2.45811400	-1.83081500	-1.04094300
H	2.18686900	-0.97410000	-3.18067700
H	0.77569300	-2.14815400	-3.21579900
H	-4.17909200	3.54110900	0.11040400
H	-3.40539500	5.10422800	1.84628500
H	0.18778900	2.80725800	2.31777000
H	-0.57573600	1.24765000	0.59693700
H	-1.56366000	5.99380700	3.21763100
H	0.00372100	5.16294500	3.23841600
H	-1.29621600	4.62785500	4.30322300
H	-5.05403200	1.44884300	0.84396100
H	-7.34078600	0.68816200	0.37535200
H	-6.14067100	-0.52313800	-3.56013600
H	-3.85705200	0.28064900	-3.11000300
H	-8.84455200	-0.73639300	-1.00262900
H	-8.33923700	-1.27751800	-2.61136700
H	-8.92562900	0.36885600	-2.37479900

Complex 3a
Energy: -4347.156434 Hartree

Ni	-0.21668100	0.03478700	-0.30358100
P	-1.20746500	-1.85208500	-0.17956100
P	-1.51483700	1.63372700	0.34190600
C	-1.82569300	-2.30560600	-1.86016700
C	-1.96785700	-3.63495000	-2.27465300
C	-2.36372500	-3.93656300	-3.57646900
C	-2.60347300	-2.90684900	-4.48551700
C	-2.46275000	-1.57910200	-4.08354200
C	-2.08486100	-1.26341200	-2.77283000
C	-1.98898300	0.16865800	-2.34948800
C	-3.20088000	0.76146200	-1.71621400
C	-4.45785500	0.53739700	-2.30022500
C	-5.61632000	1.07680900	-1.75016500
C	-5.54136500	1.85270200	-0.59291000
C	-4.30523300	2.06745700	0.01024500
C	-3.13004900	1.52198600	-0.52516900
C	-0.21881800	-3.33866000	0.26400000
C	-0.75623900	-4.46529500	0.90499600
C	0.04001800	-5.58274600	1.15450600

C	1.37901800	-5.59334400	0.76098200
C	1.91881800	-4.48217400	0.11311300
C	1.12555900	-3.36186300	-0.13347100
C	-2.68473300	-2.01342300	0.89158300
C	-3.98234200	-1.86661800	0.38649900
C	-5.08033500	-1.88542000	1.24686400
C	-4.89740200	-2.08180600	2.61655700
C	-3.60632200	-2.23704100	3.12593800
C	-2.50674400	-2.18172900	2.27341000
C	-0.92775400	3.32948500	-0.07082400
C	-1.78848100	4.34939200	-0.49969800
C	-1.29856000	5.63348300	-0.73515700
C	0.05171400	5.91864800	-0.52740300
C	0.91631500	4.90842300	-0.10582400
C	0.43311800	3.61751300	0.10573700
C	-1.94964400	1.80100700	2.11688300
C	-2.97226600	1.02069300	2.67352500
C	-3.24388600	1.08298200	4.03866900
C	-2.49174700	1.91328000	4.86953700
C	-1.46439500	2.68433900	4.32443700
C	-1.19531600	2.63167100	2.95791900
C	-0.88424000	0.90047500	-2.61480500
H	-1.74674400	-4.43727400	-1.57952400
H	-2.46811600	-4.97259600	-3.88343400
H	-2.89059900	-3.13468400	-5.50745000
H	-0.85513100	1.96664600	-2.42069200
H	-2.63678400	-0.77224900	-4.78878200
H	-4.52082900	-0.07043100	-3.19624000
H	-6.57526600	0.89113300	-2.22425600
H	-6.43964300	2.27686500	-0.15532500
H	-4.25194500	2.63936700	0.93010600
H	-1.79552000	-4.46972600	1.21297600
H	-0.38820500	-6.44608300	1.65527100
H	2.96128700	-4.47244900	-0.18767000
H	1.55519100	-2.50074200	-0.62775100
H	-4.13692600	-1.72887300	-0.67652000
H	-6.07913300	-1.75436700	0.84201000
H	-3.45346300	-2.37881400	4.19125200
H	-1.50397300	-2.27109000	2.67861900
H	-2.84038800	4.13957900	-0.65655300
H	-1.97407200	6.41247600	-1.07622500
H	1.97111800	5.11090400	0.04189700
H	1.11889000	2.82573200	0.38923200
H	-3.56385000	0.37152700	2.04182500
H	-4.04291500	0.47333800	4.44834700
H	-0.87276300	3.33405500	4.96231100
H	-0.40175400	3.24396200	2.54515300
N	1.46597000	-0.02425600	-0.65230700
H	-0.02987200	0.46163000	-3.11639200
H	1.99798300	-6.46317300	0.95955900
H	0.42971700	6.92147800	-0.70238600
H	-5.75398800	-2.11001200	3.28306800
H	-2.70350400	1.95939800	5.93349200
C	3.82359300	0.08100000	-0.17860400
N	2.59058700	-0.34254100	-0.36094000
C	4.81571500	-0.94424000	0.18182000
C	4.42417300	-2.08950800	0.90074400
C	6.16495900	-0.85126000	-0.20117500
C	5.33272000	-3.09772600	1.20053500
H	3.39438900	-2.17554900	1.22947000
C	7.07207900	-1.85860500	0.11796400

H	6.50121400	0.01003000	-0.76805200
C	6.67763100	-3.00438400	0.81781400
H	4.99498300	-3.96663100	1.76103000
H	8.10827000	-1.75747900	-0.19586500
C	4.13896500	1.50513100	-0.37374900
C	3.37341000	2.29753400	-1.24900200
C	5.21330500	2.12559400	0.29206200
C	3.68862500	3.63406800	-1.46814800
H	2.53327000	1.85400100	-1.77260300
C	5.51061400	3.46735900	0.07722900
H	5.81251500	1.54808900	0.98706600
C	4.75829200	4.25047300	-0.80738900
H	3.08585200	4.21251800	-2.16328000
H	6.34314300	3.91844200	0.61183000
C	7.65581200	-4.10943600	1.12941800
H	7.61838500	-4.90364100	0.37265800
H	8.68404700	-3.73698700	1.15788300
H	7.43862200	-4.57530400	2.09591700
C	5.06153800	5.71358300	-1.01358800
H	4.81062300	6.03403800	-2.02947500
H	4.48557400	6.34520300	-0.32414800
H	6.11967900	5.93158600	-0.84132600

Complex 4

Energy: -4237.621248 Hartree

Ni	0.10664100	-0.64889200	0.06792000
P	2.16894200	0.03037300	0.15688900
P	-1.21970200	1.08244600	-0.32319500
C	2.45094200	0.10209400	1.96151100
C	3.70677700	0.28877900	2.55620700
C	3.86037600	0.14138900	3.93100900
C	2.76062300	-0.23851800	4.70366800
C	1.51685900	-0.43815900	4.10884000
C	1.32289900	-0.24220200	2.73048000
C	-0.01316300	-0.46004700	2.04161100
C	-1.05986900	0.58697100	2.40545500
C	-1.45490500	0.79427300	3.73972100
C	-2.41231400	1.74252300	4.08755200
C	-3.00471900	2.53655000	3.10358300
C	-2.63957900	2.34921800	1.77606200
C	-1.70206300	1.36805900	1.42035800
C	2.61686900	1.65754500	-0.55914000
C	3.39021200	2.61917700	0.10025600
C	3.72252700	3.81309100	-0.53917100
C	3.28979300	4.05601400	-1.84249700
C	2.50877700	3.10751400	-2.50324400
C	2.16700900	1.92026200	-1.86159300
C	3.58997500	-1.00617500	-0.39436600
C	4.28818500	-0.74179900	-1.57898600
C	5.34756200	-1.55849100	-1.97434900
C	5.72048200	-2.65389300	-1.19511300
C	5.01940900	-2.93309400	-0.02056000
C	3.96001300	-2.11949500	0.37358200
C	-0.79472000	2.80678200	-0.82142000
C	0.01006400	3.56024000	0.04828900
C	0.39705100	4.85396700	-0.28392700
C	-0.00789400	5.42045100	-1.49412700
C	-0.80108100	4.67852700	-2.36720100
C	-1.19340200	3.38116100	-2.03413000
C	-2.74921600	0.76968300	-1.28633400

C	-4.05036700	0.83059000	-0.77803900
C	-5.14212800	0.54384900	-1.59620800
C	-4.95385200	0.19527700	-2.93287300
C	-3.66009400	0.12464300	-3.45091900
C	-2.56793300	0.40118100	-2.63136900
C	-0.56536100	-1.89801200	2.18867100
C	-1.10047300	-2.09118800	0.77284300
C	-0.18032100	-2.62138700	-0.22929100
C	-0.23527100	-2.02887500	-1.54229300
C	0.70989300	-2.42317600	-2.52733700
C	1.70060600	-3.34231900	-2.26401600
C	1.73982500	-3.93001600	-0.96712600
C	0.85471100	-3.57383400	0.01756400
C	2.69917800	-3.76757900	-3.30775500
C	-2.56275900	-2.10535800	0.56548800
C	-3.44896100	-1.55172800	1.50860700
C	-4.83013000	-1.62546500	1.34187800
C	-5.39890800	-2.24274400	0.22695000
C	-4.52398300	-2.80504500	-0.71234300
C	-3.14818700	-2.74909200	-0.54555600
C	-6.89103900	-2.29926800	0.02458100
H	4.57088700	0.50003000	1.93444300
H	4.83294000	0.28404400	4.39176200
H	2.87758600	-0.39989700	5.77146600
H	0.69393400	-0.78428700	4.72360600
H	-1.01127900	0.19683000	4.52581000
H	-2.68977500	1.86837000	5.13021100
H	-3.73685300	3.29233600	3.37072600
H	-3.07287800	2.97452900	1.00201300
H	3.72071700	2.44476500	1.11739300
H	4.31718200	4.55619900	-0.01604600
H	3.54328000	4.98943900	-2.33534400
H	2.14219000	3.30499800	-3.50513300
H	1.52942900	1.19465100	-2.35812500
H	4.02010600	0.11579700	-2.18540700
H	5.88563500	-1.33280700	-2.89024900
H	6.55017300	-3.28463700	-1.49951800
H	5.29888900	-3.78550200	0.59156300
H	3.42030400	-2.34310200	1.28669200
H	0.33162700	3.12934600	0.99038400
H	1.02373800	5.41777600	0.39962400
H	0.29503800	6.43042500	-1.75324600
H	-1.12808600	5.11159900	-3.30827400
H	-1.82694200	2.82549200	-2.71581900
H	-4.21387800	1.07545300	0.26254800
H	-6.14441000	0.58712400	-1.18173200
H	-5.80787500	-0.02941000	-3.56454400
H	-3.50031600	-0.15463600	-4.48805500
H	-1.56076300	0.33404800	-3.03419300
H	0.25293300	-2.59061000	2.40230400
H	-1.32038500	-2.03373600	2.97296500
H	-1.15377200	-1.57087100	-1.88491700
H	0.62605700	-2.00027700	-3.52597500
H	2.50051100	-4.67646300	-0.75698100
H	0.91276300	-4.04415200	0.99397300
H	2.54645400	-3.22784000	-4.24645900
H	2.62009700	-4.84084000	-3.52025700
H	3.72278000	-3.57689700	-2.97137400
H	-3.06411300	-1.03711300	2.37848300
H	-5.47655700	-1.17940500	2.09394700
H	-4.93079900	-3.30479600	-1.58808400

H	-2.51132900	-3.23270200	-1.27559200
H	-7.42458000	-1.82942700	0.85560000
H	-7.24847000	-3.33194000	-0.06119400
H	-7.18427400	-1.78233500	-0.89707200

4-CO
Energy: -4351.01481 Hartree

Ni	0.07751200	0.37404900	-0.11091000
P	-1.85938000	-0.73419900	-0.21300000
P	1.71004300	-1.17770300	-0.08514100
C	-2.48261800	-0.41402700	1.46932400
C	-3.82084900	-0.54963300	1.85407100
C	-4.21598500	-0.17388300	3.13429200
C	-3.26876700	0.35097300	4.01721700
C	-1.93792800	0.48288700	3.62665400
C	-1.51142100	0.08465300	2.34869300
C	-0.07372300	0.23241400	1.87374700
C	0.83899800	-0.82571000	2.48816600
C	0.81397200	-1.11900400	3.86385700
C	1.67173500	-2.05636100	4.43004500
C	2.58368100	-2.75281200	3.63408400
C	2.62268500	-2.49170300	2.27035300
C	1.77057900	-1.53149800	1.69927100
C	-1.93144200	-2.55529100	-0.38758300
C	-2.18275200	-3.39830400	0.70044000
C	-2.29146400	-4.77745900	0.51040800
C	-2.15486600	-5.32217700	-0.76447700
C	-1.87848200	-4.48776100	-1.85074600
C	-1.75674800	-3.11601700	-1.66267100
C	-3.24683900	-0.19858800	-1.28915800
C	-4.30585400	-1.04631300	-1.64809900
C	-5.36477800	-0.55986100	-2.41379900
C	-5.37902300	0.77380000	-2.82687600
C	-4.32958600	1.62048400	-2.46962100
C	-3.26924300	1.13678600	-1.70582900
C	1.57923400	-2.84112500	-0.87149700
C	1.19121500	-3.97648500	-0.14836300
C	1.12242500	-5.22167300	-0.77039700
C	1.41597900	-5.35175600	-2.12646800
C	1.76613200	-4.22017300	-2.86385300
C	1.84773500	-2.97662000	-2.24225100
C	3.40630300	-0.68794900	-0.62122800
C	4.55494800	-0.85628500	0.16190500
C	5.80296400	-0.45410500	-0.31518500
C	5.92341000	0.11854000	-1.58121100
C	4.78305300	0.30539600	-2.36202300
C	3.53545300	-0.08425400	-1.88135400
C	0.46144900	1.66432000	2.08662500
C	0.40007400	2.27944300	0.67908500
C	-0.85531100	3.02156300	0.33580600
C	-1.01072700	3.66910000	-0.91118000
C	-2.17285100	4.35194200	-1.25184700
C	-3.27247200	4.40049300	-0.38533400
C	-3.13789500	3.75187500	0.84389900
C	-1.96262400	3.09372200	1.20085300
C	-4.56171600	5.07375800	-0.78073400
C	1.69978200	2.94818600	0.31374400
C	2.91446600	2.26436200	0.50790900
C	4.14601700	2.83821400	0.23084800
C	4.24026600	4.14865700	-0.25489700

C	3.04791300	4.85776800	-0.40603400
C	1.80945600	4.27888900	-0.12339900
C	5.57645600	4.75417200	-0.60281300
H	-4.55193200	-0.91572700	1.14033500
H	-5.25524300	-0.26360400	3.43466600
H	-3.57226600	0.67638200	5.00796000
H	-1.23267700	0.94488600	4.30981400
H	0.10513100	-0.61294300	4.50529300
H	1.62152000	-2.25133400	5.49737900
H	3.24301700	-3.49704200	4.06944300
H	3.29286000	-3.05857600	1.63287900
H	-2.31252800	-2.97566900	1.69052200
H	-2.49509500	-5.42218400	1.36023300
H	-2.24860600	-6.39360000	-0.91280600
H	-1.74315000	-4.90946500	-2.84114000
H	-1.54056800	-2.47162000	-2.50923100
H	-4.29914500	-2.08527200	-1.33791900
H	-6.17841300	-1.22433900	-2.68928200
H	-6.20295100	1.14822900	-3.42703700
H	-4.32514200	2.65869500	-2.78509700
H	-2.46204500	1.80209600	-1.43080800
H	0.93974500	-3.89208000	0.90083200
H	0.82378800	-6.08943300	-0.19130600
H	1.36240300	-6.32353000	-2.60768500
H	1.98840800	-4.30562500	-3.92337700
H	2.14478300	-2.11268200	-2.82605600
H	4.47895500	-1.27589800	1.15682500
H	6.68205800	-0.58723400	0.30839300
H	6.89542400	0.43232200	-1.94885500
H	4.85905000	0.77510200	-3.33763500
H	2.65395200	0.11397900	-2.47954700
H	-0.08264300	2.24538200	2.84527700
H	1.49658600	1.62760900	2.42476700
H	-0.20741300	3.62507000	-1.63576900
H	-2.23410800	4.83843200	-2.22257400
H	-3.96834800	3.75815200	1.54585300
H	-1.92929100	2.61594000	2.16837500
H	-4.40320300	5.80415700	-1.57974900
H	-5.02019400	5.59359800	0.06654100
H	-5.29443400	4.34181200	-1.14425300
H	2.89506200	1.25271900	0.88739300
H	5.04678300	2.25079600	0.38238900
H	3.08048000	5.89109900	-0.74371600
H	0.92224300	4.89023600	-0.22259900
H	6.31211100	4.58756400	0.19176600
H	5.49621500	5.83253600	-0.76696500
H	5.98849400	4.31041800	-1.51785800
C	0.24402200	0.94128400	-1.76702000
O	0.36667800	1.30933400	-2.85502300

4-MeCN
Energy: -4370.411706 Hartree

Ni	0.01732600	0.37430800	0.04056100
P	-1.83380700	-0.88187200	-0.08612300
P	1.75296400	-1.09118300	-0.10004800
C	-2.43040000	-0.67520200	1.62018400
C	-3.74428400	-0.92009000	2.03369600
C	-4.13197800	-0.61231700	3.33456400
C	-3.20090800	-0.04693000	4.20927400
C	-1.89320700	0.19279300	3.79087400

C	-1.47099100	-0.13386000	2.49145200
C	-0.05809500	0.13399300	1.98698400
C	0.94056900	-0.89241600	2.51833600
C	0.97742800	-1.24207900	3.88044700
C	1.91556100	-2.13618900	4.38522700
C	2.84919500	-2.73353900	3.53572500
C	2.82714700	-2.41734900	2.18343300
C	1.89763100	-1.49551700	1.67291000
C	-1.84057700	-2.68988500	-0.37668100
C	-2.09194100	-3.61801100	0.63973900
C	-2.11915700	-4.98480700	0.35406100
C	-1.90391800	-5.43439100	-0.94693500
C	-1.62918400	-4.51477100	-1.96257300
C	-1.58421200	-3.15551200	-1.67685600
C	-3.25268700	-0.33095700	-1.12154900
C	-4.18638000	-1.20448800	-1.69733100
C	-5.23108800	-0.70764900	-2.47790200
C	-5.35809900	0.66527000	-2.69218400
C	-4.43900300	1.54234300	-2.11239800
C	-3.39359900	1.04829600	-1.33381800
C	1.75069100	-2.75053000	-0.90760200
C	1.37419100	-3.91177200	-0.21825000
C	1.37423900	-5.14851000	-0.85975800
C	1.72383400	-5.24740500	-2.20574200
C	2.06674700	-4.09275500	-2.91063300
C	2.08294900	-2.85725800	-2.26747600
C	3.37791200	-0.46076200	-0.70574400
C	4.61715800	-0.71764400	-0.10733500
C	5.78907900	-0.19666700	-0.65544000
C	5.74157100	0.58102000	-1.81327300
C	4.51159100	0.84648800	-2.41412300
C	3.33975500	0.33715200	-1.85881400
C	0.39595200	1.58090800	2.27921100
C	0.35337900	2.23414500	0.88986700
C	-0.90202600	2.96402500	0.55486200
C	-1.06943200	3.66568300	-0.66374000
C	-2.24931700	4.32966500	-0.98940300
C	-3.35399200	4.32650500	-0.13166200
C	-3.21413000	3.62115400	1.06890700
C	-2.03337700	2.96743000	1.40354100
C	-4.64419400	5.02051100	-0.48792500
C	1.66227400	2.88208000	0.53527100
C	2.87017500	2.20660700	0.80791700
C	4.11377400	2.75957000	0.54617900
C	4.23754500	4.03468100	-0.01846400
C	3.05621300	4.73964700	-0.25011200
C	1.80475400	4.18743200	0.03011000
C	5.59082800	4.60236700	-0.36258500
H	-4.46433100	-1.31927200	1.32567000
H	-5.15355900	-0.78642300	3.65798900
H	-3.49980300	0.22472600	5.21757500
H	-1.20351300	0.67884900	4.47330100
H	0.25264000	-0.81308200	4.55989200
H	1.91093900	-2.37656800	5.44456200
H	3.57158600	-3.44638300	3.92106800
H	3.50779100	-2.92020100	1.50494700
H	-2.28079600	-3.27136600	1.64973500
H	-2.32060700	-5.69573500	1.15000800
H	-1.93287400	-6.49673900	-1.16906600
H	-1.42806800	-4.86070600	-2.97104900
H	-1.35931000	-2.44467100	-2.46623100

H	-4.09508200	-2.27348500	-1.54333300
H	-5.94670000	-1.39624600	-2.91766300
H	-6.17010400	1.04891400	-3.30290000
H	-4.52860400	2.61393400	-2.25818700
H	-2.68670400	1.73518200	-0.89009900
H	1.08116600	-3.85258000	0.82238000
H	1.08508200	-6.03539900	-0.30516900
H	1.72303000	-6.21279800	-2.70278000
H	2.33906400	-4.15528400	-3.96042100
H	2.38406500	-1.97276300	-2.81940300
H	4.67157400	-1.30073000	0.80332800
H	6.74182500	-0.39723600	-0.17402800
H	6.65563200	0.98719100	-2.23561700
H	4.46155200	1.47026300	-3.30142700
H	2.38036000	0.58647500	-2.29873600
H	-0.21744000	2.09801100	3.02845000
H	1.41229200	1.59186800	2.67140500
H	-0.25439500	3.68664000	-1.37407600
H	-2.31237700	4.86078500	-1.93735400
H	-4.05066000	3.57647700	1.76255400
H	-2.00936700	2.43014600	2.34014400
H	-4.54837200	5.58467500	-1.42061100
H	-4.95509200	5.72244100	0.29420200
H	-5.46422200	4.30286100	-0.61602100
H	2.83600400	1.21181900	1.23166600
H	5.00445700	2.17645600	0.75988400
H	3.10728500	5.75186700	-0.64585600
H	0.93184400	4.80893600	-0.11635400
H	6.27434400	4.56395100	0.49337900
H	5.51759000	5.64465800	-0.68641900
H	6.06387500	4.03340900	-1.17298600
N	-0.11540800	0.97982700	-1.72250600
C	-0.47166800	1.38595400	-2.74755200
C	-1.01354400	1.92362300	-3.98366800
H	-0.73568100	2.97639600	-4.08598500
H	-0.64059300	1.37032200	-4.84999200
H	-2.10491400	1.84635400	-3.94742100

Complex 5

Energy: -4464.420009 Hartree

Ni	1.75422700	-0.46328600	-1.38150300
P	0.57907300	-2.00410200	-0.32024900
P	2.04263100	1.32996200	-0.11294200
O	4.47268500	-1.45996400	-1.76407500
O	0.52274900	0.37031600	-3.89469200
C	-0.15535100	-1.60620600	1.33503000
C	0.01949500	-2.55123400	2.36600500
C	-0.38874900	-2.31179700	3.67182100
C	-0.98906900	-1.09343000	3.98037100
C	-1.19124600	-0.16244100	2.97153000
C	-0.81291700	-0.39000600	1.63547600
C	-1.11692300	0.74397700	0.67310000
C	-0.48020100	2.03202900	1.15222200
C	-1.22170200	2.89078900	1.98388100
C	-0.66940200	4.03523300	2.54185000
C	0.66555500	4.35018800	2.28786400
C	1.41507700	3.51187200	1.47495100
C	0.86269800	2.35697700	0.88756800
C	1.65494000	-3.44989600	0.07431500
C	1.36683000	-4.75657800	-0.33477800

C	2.25359200	-5.79737600	-0.04735700
C	3.42710200	-5.54752000	0.66053900
C	3.71909100	-4.24564700	1.07626500
C	2.84623200	-3.20516300	0.77752200
C	-0.83370400	-2.82849300	-1.16958300
C	-0.86451500	-2.85692400	-2.56963900
C	-1.86553900	-3.55989600	-3.24035800
C	-2.83863200	-4.25055200	-2.51839400
C	-2.81846000	-4.22073300	-1.12349300
C	-1.82804000	-3.50861400	-0.45222800
C	3.28962300	1.03664500	1.21296400
C	4.55560700	1.62608900	1.27682800
C	5.42913500	1.30695900	2.31944700
C	5.04456900	0.40898100	3.31336000
C	3.77283200	-0.16759900	3.26702700
C	2.90376900	0.14256600	2.22536500
C	2.78504600	2.59664500	-1.22245600
C	4.00435900	2.31255900	-1.86091700
C	4.53035500	3.18757400	-2.80769100
C	3.83389200	4.34590900	-3.15831000
C	2.60878600	4.62051200	-2.55345000
C	2.08778500	3.75482700	-1.59110800
C	-2.54065100	0.84925700	0.00932300
C	-3.49913200	-0.31165500	0.10408300
C	-4.04491700	-0.80358200	-1.09013300
C	-4.99878500	-1.81185500	-1.08193900
C	-5.43666100	-2.38014100	0.12224100
C	-4.89138200	-1.89200800	1.31134400
C	-3.94381300	-0.86566400	1.30601900
C	-3.22458500	2.18779900	-0.09896700
C	-2.67491300	3.25714100	-0.81915500
C	-3.30872300	4.49485100	-0.86934600
C	-4.51931600	4.71977600	-0.20495500
C	-5.07907000	3.64859200	0.49902200
C	-4.44878300	2.40767200	0.54851300
C	-6.45502300	-3.49238700	0.12030700
C	-5.18400000	6.07320900	-0.23232100
C	-1.31238700	0.56971700	-0.80479800
C	3.39158200	-1.08598100	-1.58258900
C	0.99189000	0.03946000	-2.88749100
H	0.50561300	-3.49093700	2.13826800
H	-0.22837200	-3.06468800	4.43719100
H	-1.30173700	-0.86819100	4.99530000
H	-1.66091800	0.78072800	3.22083700
H	-2.26194800	2.66127400	2.17456800
H	-1.27834700	4.67549300	3.17249800
H	1.11939000	5.23531800	2.72262700
H	2.45680000	3.74991700	1.28950000
H	0.45349700	-4.96662800	-0.87822700
H	2.02122700	-6.80505700	-0.37889200
H	4.11385500	-6.35846300	0.88321600
H	4.63325400	-4.03781700	1.62393900
H	3.08825400	-2.19590800	1.09238500
H	-0.10967200	-2.32021300	-3.13365800
H	-1.88033500	-3.56977600	-4.32587800
H	-3.61554600	-4.80140600	-3.04010400
H	-3.58179400	-4.74155000	-0.55503800
H	-1.83310500	-3.47507200	0.63084300
H	4.86540300	2.33563500	0.51870500
H	6.41219700	1.76726200	2.35295000
H	5.72732700	0.16285700	4.12077800

H	3.45562000	-0.86110900	4.04005400
H	1.91568800	-0.30378200	2.19978400
H	4.53630300	1.39746800	-1.62600800
H	5.47948000	2.95648200	-3.28148600
H	4.23964900	5.02243900	-3.90404300
H	2.05274600	5.51171300	-2.82873100
H	1.13715800	3.98634800	-1.12462100
H	-3.70921200	-0.38511700	-2.03394700
H	-5.39772800	-2.17914400	-2.02345100
H	-5.21340900	-2.31423500	2.25970000
H	-3.54989100	-0.50338300	2.24656500
H	-1.73401500	3.13608500	-1.34266500
H	-2.85347400	5.30148300	-1.43821400
H	-6.02754200	3.78430100	1.01250800
H	-4.91690900	1.59447500	1.09170300
H	-7.38815000	-3.17874000	-0.36084200
H	-6.69439800	-3.81786300	1.13607100
H	-6.08883500	-4.36488700	-0.43365400
H	-4.78085900	6.72903900	0.54945500
H	-6.26193900	5.99241600	-0.06588000
H	-5.02511200	6.57724000	-1.19038200
H	-0.90589200	1.33867200	-1.44774200
H	-1.26122200	-0.41795300	-1.23408200

Complex 6

Energy: -3751.030571 Hartree

Ni	-0.00223200	-0.55500700	-0.26028600
P	2.07930600	-0.16299300	-0.12153100
P	-2.12178100	-0.10964900	-0.11823600
N	-0.41526800	-3.56925300	-1.41839100
C	2.44301500	-0.22640500	1.65879400
C	3.70424300	-0.27077200	2.25968600
C	3.79855600	-0.32640400	3.64821500
C	2.63325600	-0.33007100	4.42186500
C	1.37943700	-0.27028500	3.81436300
C	1.26265900	-0.21106600	2.41871600
C	-0.06029400	-0.13208400	1.69675000
C	-0.74286300	1.21062400	1.76517000
C	-0.32936300	2.31628000	2.51316700
C	-1.03642200	3.52147700	2.44979100
C	-2.17153800	3.64593000	1.64910500
C	-2.59640000	2.55475800	0.88425600
C	-1.87709800	1.36333800	0.92970400
C	3.26186500	-1.27678000	-0.94087300
C	3.70087100	-1.03765700	-2.25132100
C	4.48968500	-1.97906400	-2.91106000
C	4.83984300	-3.16970000	-2.27420800
C	4.39404800	-3.41990800	-0.97540600
C	3.60494100	-2.48295700	-0.31253900
C	2.54034200	1.53646600	-0.62643200
C	1.52846100	2.50619100	-0.66937600
C	1.83887600	3.83544400	-0.95135400
C	3.16061400	4.20764900	-1.19753400
C	4.17405000	3.24815000	-1.15217000
C	3.86789700	1.91966900	-0.86220400
C	-3.24769900	-1.22445600	0.78679300
C	-3.14234000	-2.60582300	0.56998000
C	-4.00109500	-3.47838500	1.23903500
C	-4.95567600	-2.98181700	2.12745400
C	-5.05434400	-1.60687000	2.35239400

C	-4.20324600	-0.72884100	1.68431400
C	-3.07819000	0.48241800	-1.55442000
C	-2.49308500	1.46598500	-2.36824200
C	-3.16263400	1.93631800	-3.49395000
C	-4.41798200	1.42087300	-3.82752200
C	-4.99886400	0.43652100	-3.02939100
C	-4.33349400	-0.03238900	-1.89536100
C	-0.20106000	-2.49179900	-1.82119600
C	0.03567200	-1.11388600	-2.14794700
H	4.60039300	-0.28815400	1.64665400
H	4.77141000	-0.37755800	4.12726000
H	2.70544000	-0.38358200	5.50428500
H	0.47872400	-0.27232100	4.42196000
H	-0.73242900	-0.86974500	2.16011100
H	0.55388300	2.23920500	3.13832600
H	-0.69164900	4.37199200	3.03113500
H	-2.71114800	4.58672000	1.60388800
H	-3.45216800	2.64628100	0.22216200
H	3.43305100	-0.11274500	-2.75120800
H	4.82803500	-1.78240600	-3.92372100
H	5.45217700	-3.90282800	-2.78986000
H	4.65550700	-4.34934600	-0.47945800
H	3.25175700	-2.68496600	0.69298600
H	0.50248400	2.22191800	-0.46332700
H	1.04678300	4.57733300	-0.97501300
H	3.40243400	5.24219500	-1.42135700
H	5.20442500	3.53514400	-1.33904200
H	4.65965600	1.17871000	-0.82664600
H	-2.38732500	-2.99750900	-0.10590500
H	-3.91563900	-4.54724100	1.07011900
H	-5.61804200	-3.66499200	2.65068300
H	-5.79110500	-1.22001200	3.04997700
H	-4.26902100	0.33949600	1.86514400
H	-1.51384200	1.85979100	-2.11206300
H	-2.70453400	2.70015900	-4.11482000
H	-4.93756600	1.78386200	-4.70893700
H	-5.97251000	0.03104200	-3.28699000
H	-4.78918500	-0.79645800	-1.27518300
H	-0.76789700	-0.71131400	-2.76761600
H	0.98655400	-1.00209900	-2.67257900

Complex 9

Energy: -4347.134662 Hartree

Ni	0.10924700	-0.23614100	-0.80335700
P	1.59137400	-0.93470800	0.61986900
P	-2.07484500	-0.47843700	-0.41404500
C	2.06962800	-2.53162800	-0.15813200
C	3.17747900	-3.29161400	0.23846000
C	3.62796900	-4.33940000	-0.55880500
C	2.99935900	-4.59386400	-1.78081700
C	1.89502400	-3.83991000	-2.17321500
C	1.38621200	-2.82590600	-1.34723100
C	0.15202900	-2.03422900	-1.69496600
C	-1.14772500	-2.79922900	-1.57992400
C	-1.26941900	-4.13984000	-1.98296800
C	-2.48258400	-4.81765100	-1.89261400
C	-3.61728000	-4.17772600	-1.39089100
C	-3.51562600	-2.85725100	-0.96768200
C	-2.29451800	-2.17702400	-1.04767200
C	3.30209400	-0.24645000	0.78662100

C	3.92491900	0.09983000	1.98985900
C	5.24593100	0.55376000	1.99951000
C	5.96466800	0.65157800	0.81023500
C	5.34966800	0.30566000	-0.39540100
C	4.03117200	-0.13234000	-0.40712100
C	1.15605700	-1.35511400	2.35262500
C	0.86699900	-2.67022900	2.74167500
C	0.40035400	-2.94350200	4.02792000
C	0.22438600	-1.91009200	4.94884500
C	0.50700300	-0.59524200	4.57165100
C	0.95994300	-0.32003400	3.28379500
C	-3.11913700	0.52194100	-1.54227200
C	-3.37303200	0.08852400	-2.85191600
C	-3.99927400	0.93059700	-3.77064100
C	-4.38279700	2.21864600	-3.39743800
C	-4.14156900	2.65739300	-2.09401300
C	-3.51453800	1.81973400	-1.17521700
C	-3.05828400	-0.49238400	1.14679400
C	-2.40833000	-0.95533000	2.29921700
C	-3.06272100	-0.97213800	3.52915800
C	-4.37940900	-0.51869400	3.62745100
C	-5.04034000	-0.06758300	2.48456200
C	-4.38736700	-0.06018200	1.25156200
C	0.27743700	-0.97489200	-2.63798200
H	3.70519800	-3.03321000	1.15133900
H	4.48601300	-4.92954900	-0.25163800
H	3.37805500	-5.37441300	-2.43422500
H	-0.57093300	-0.71923700	-3.27206200
H	1.43472800	-4.02091200	-3.13898900
H	-0.40606900	-4.66772800	-2.36483600
H	-2.53942000	-5.85364900	-2.21415400
H	-4.56210100	-4.70741300	-1.31681500
H	-4.37875200	-2.35046100	-0.54745200
H	3.39393800	-0.00086900	2.92865100
H	5.71563000	0.81444700	2.94366700
H	5.89683600	0.38702900	-1.32933600
H	3.55852800	-0.39700000	-1.34695800
H	0.99814400	-3.47891800	2.03107000
H	0.17595200	-3.96757700	4.31092300
H	0.36448300	0.21739700	5.27769200
H	1.15258400	0.70825100	2.99467800
H	-3.07916400	-0.91130400	-3.15125400
H	-4.18497600	0.57848600	-4.78099400
H	-4.43551900	3.65767000	-1.78975300
H	-3.33152400	2.17667000	-0.16917200
H	-1.38276400	-1.29556300	2.23060300
H	-2.53558300	-1.33203200	4.40636000
H	-6.06864700	0.27654800	2.54993800
H	-4.91262900	0.29348600	0.37193800
N	-0.74726200	1.98199900	-3.21004700
H	1.25336800	-0.79541400	-3.08724700
H	6.99646900	0.99020100	0.82039800
H	-4.86571000	2.87481200	-4.11506200
H	-0.13447400	-2.12568400	5.95060800
H	-4.88789900	-0.52009800	4.58712400
C	0.28503600	1.82948000	-0.93588700
N	-0.29698200	1.87825600	-2.17530100
C	1.71646700	2.28910600	-1.02231800
C	2.44578800	2.27740800	-2.21646600
C	2.34101200	2.78931400	0.12867700
C	3.74446500	2.77992400	-2.26095200

H	2.00182100	1.88196400	-3.12418800
C	3.63820900	3.28221900	0.07540000
H	1.79845500	2.80440200	1.06648800
C	4.36451000	3.29743200	-1.12094300
H	4.28478300	2.76690300	-3.20389700
H	4.09969100	3.65478700	0.98544700
C	-0.58030300	2.58213000	0.06011900
C	-1.07669700	3.85183200	-0.25097300
C	-0.89164100	2.03190200	1.30503300
C	-1.90416300	4.52911300	0.64262800
H	-0.83865000	4.30313700	-1.20976300
C	-1.71263700	2.71113800	2.19957300
H	-0.52737100	1.04458700	1.54501000
C	-2.24544900	3.96597900	1.87874700
H	-2.30138800	5.50352900	0.37150300
H	-1.97062300	2.23689400	3.14238400
C	5.77593600	3.82276500	-1.15949900
H	5.83985200	4.83527600	-0.74619500
H	6.44270200	3.18807800	-0.56464100
H	6.16396900	3.85205100	-2.18137200
C	-3.15208800	4.68575100	2.84500900
H	-2.58147000	5.12052700	3.67442400
H	-3.69443700	5.49905800	2.35560800
H	-3.88520600	4.00006800	3.28119400

Complex 9a

Energy: -4347.13318 Hartree

Ni	-0.12479000	-0.33873400	-0.67326500
P	-0.79306600	1.23133900	0.73370400
P	2.05474800	-0.33091200	-0.81968500
C	-0.97964200	2.92824300	0.02226900
C	-1.87687200	3.84298200	0.59245300
C	-2.11452900	5.08055600	-0.00028500
C	-1.47281500	5.41350700	-1.19298400
C	-0.56870000	4.51986000	-1.76339100
C	-0.29782500	3.28746900	-1.15559700
C	0.75842400	2.39823500	-1.72184200
C	2.07282300	2.47733500	-1.02332000
C	2.59525400	3.74906200	-0.73155100
C	3.80397400	3.90055900	-0.06155300
C	4.51734100	2.77205300	0.34393000
C	4.00736300	1.50540600	0.07856000
C	2.78538000	1.33638100	-0.58870900
C	-2.49539600	1.02472700	1.40134000
C	-2.83095900	0.93931600	2.75746600
C	-4.15775500	0.73714500	3.14194100
C	-5.16276200	0.63081600	2.18040200
C	-4.84034700	0.75231700	0.82790100
C	-3.51809000	0.95448600	0.44515700
C	0.24025200	1.46950800	2.22560100
C	1.09135400	2.57143800	2.37179000
C	1.95502200	2.65305300	3.46419700
C	1.95503300	1.65420700	4.43882900
C	1.10449100	0.55488500	4.30163900
C	0.27267400	0.44727800	3.18922100
C	2.79289000	-0.87330400	-2.40716300
C	3.67878700	-0.06483900	-3.13447800
C	4.20087800	-0.50116800	-4.35116100
C	3.85168000	-1.75430900	-4.85591000
C	2.97421600	-2.56731200	-4.13987200

C	2.44104400	-2.12749200	-2.92955400
C	2.93234100	-1.36214800	0.42149800
C	2.83111000	-0.98172300	1.76583900
C	3.39070800	-1.76301800	2.77144500
C	4.06209500	-2.94397200	2.44914900
C	4.17798800	-3.32454800	1.11237700
C	3.62153600	-2.53782400	0.10217300
C	0.50046400	1.61240900	-2.77859400
H	-2.41174900	3.57095300	1.49530800
H	-2.81220400	5.77394000	0.45885500
H	-1.67309900	6.36462900	-1.67669000
H	1.25219100	0.98627300	-3.24082900
H	-0.05494600	4.77082600	-2.68623000
H	2.03645100	4.62715400	-1.03624100
H	4.18608100	4.89563400	0.14470000
H	5.46147800	2.87658400	0.86927900
H	4.55294900	0.63009000	0.41101100
H	-2.06519700	1.02628500	3.51768700
H	-4.40310600	0.66370500	4.19735300
H	-5.60664900	0.66688300	0.06536600
H	-3.27887900	1.04191800	-0.60668800
H	1.09149200	3.35799700	1.62673800
H	2.62458300	3.50292400	3.55364500
H	1.10759500	-0.23663400	5.04505300
H	-0.32872300	-0.44420600	3.05028700
H	3.96148300	0.90802000	-2.74872000
H	4.88568800	0.13724600	-4.90121800
H	2.68782100	-3.53931600	-4.52848900
H	1.75380700	-2.76518500	-2.39053900
H	2.32049300	-0.06557600	2.02213700
H	3.29748500	-1.44486300	3.80499100
H	4.70871900	-4.23468700	0.84922700
H	3.73077600	-2.84287200	-0.93115200
N	-0.49644600	-1.88429100	-3.04728600
H	-0.48849800	1.60024800	-3.22418100
H	-6.19125800	0.45984500	2.48347400
H	4.25866200	-2.09329900	-5.80363900
H	2.61906400	1.72733000	5.29464400
H	4.49566700	-3.55892300	3.23191200
C	-1.59344600	-1.68481400	-0.83994600
N	-0.61512900	-1.62213000	-1.89618300
C	-2.98460800	-1.58738900	-1.28863500
C	-3.33593000	-0.77566600	-2.38503700
C	-4.01781700	-2.32048600	-0.68038400
C	-4.65184500	-0.67671000	-2.81995200
H	-2.56583600	-0.19756400	-2.88571100
C	-5.33338100	-2.21635300	-1.12180400
H	-3.78814800	-2.97934300	0.14799900
C	-5.68391100	-1.38910400	-2.19426700
H	-4.88490500	-0.02934300	-3.66208000
H	-6.10596500	-2.79347800	-0.61921200
C	-1.14485600	-2.39180800	0.37364400
C	0.01106400	-3.20402300	0.37531800
C	-1.80973200	-2.22935500	1.60720400
C	0.51524100	-3.74589800	1.54907000
H	0.53030800	-3.38902900	-0.55785100
C	-1.31038800	-2.80167500	2.77490800
H	-2.70594000	-1.62519300	1.65318400
C	-0.12398400	-3.54370700	2.77942700
H	1.43218800	-4.32619700	1.51090900
H	-1.84520300	-2.64272800	3.70865800

C	-7.10728700	-1.29729300	-2.68481800
H	-7.30259200	-2.01874400	-3.48854500
H	-7.82117000	-1.50663100	-1.88224800
H	-7.33021600	-0.30283700	-3.08445700
C	0.46279600	-4.09715400	4.05246300
H	0.59084400	-5.18459100	4.00059500
H	1.45354300	-3.66795100	4.24405700
H	-0.17324800	-3.87706800	4.91465100

Complex 10
Energy: -4237.606757 Hartree

Ni	0.35321000	-0.02918900	-0.76162300
P	1.53939200	-1.12733900	0.62812500
P	-2.04504900	-0.13481000	-0.59821200
C	2.18855900	-2.48283100	-0.41423200
C	3.34464200	-3.23050200	-0.17510000
C	3.79087800	-4.13085800	-1.14152100
C	3.09354100	-4.26191200	-2.34535700
C	1.93712200	-3.51493800	-2.57789400
C	1.46164000	-2.62853800	-1.60480000
C	0.20637500	-1.81030700	-1.76194000
C	-1.08102100	-2.55298100	-1.55831200
C	-1.17545100	-3.93735800	-1.77941900
C	-2.37674400	-4.62149200	-1.60466400
C	-3.52593600	-3.93982900	-1.20575300
C	-3.44937800	-2.57117400	-0.96066100
C	-2.24097800	-1.88065400	-1.11109000
C	3.08185400	-0.51270200	1.41192600
C	3.40171800	-0.64215600	2.76945600
C	4.62024100	-0.16615800	3.25663300
C	5.53742600	0.43329700	2.39440500
C	5.23564500	0.54584700	1.03634200
C	4.01961600	0.07680700	0.54927800
C	0.58414700	-1.91210200	1.97724700
C	0.05293000	-3.19895600	1.81064900
C	-0.78295900	-3.74712700	2.78258600
C	-1.09093600	-3.02500800	3.93721500
C	-0.57759600	-1.73746100	4.10413600
C	0.24075800	-1.17788100	3.12486300
C	-2.81229200	0.82755200	-1.95818900
C	-3.36420200	0.22576300	-3.09639700
C	-3.87235700	1.00812700	-4.13482900
C	-3.84468900	2.39935500	-4.04529000
C	-3.29946400	3.00740100	-2.91244300
C	-2.77878700	2.22984500	-1.88078500
C	-3.27866100	0.00675000	0.75876300
C	-3.05431400	-0.77845600	1.90065300
C	-3.85303800	-0.63311300	3.03058400
C	-4.89075100	0.30329100	3.04224400
C	-5.12726100	1.07954200	1.90780100
C	-4.32909000	0.93216100	0.77199000
C	0.26680400	-0.68343300	-2.63513800
H	3.90290600	-3.08579900	0.74430600
H	4.69074900	-4.71300100	-0.96785400
H	3.45733700	-4.94259200	-3.10944800
H	-0.62807600	-0.36168200	-3.15939200
H	1.40476200	-3.60911900	-3.51954200
H	-0.29598500	-4.48941700	-2.08711900
H	-2.41124000	-5.69275000	-1.78155800
H	-4.46388900	-4.46908200	-1.06911600

H	-4.32444600	-2.03321500	-0.61083700
H	2.71105900	-1.12549400	3.44942200
H	4.85294900	-0.27272400	4.31207100
H	5.94005100	1.00878400	0.35303500
H	3.79482600	0.16959600	-0.50602400
H	0.28299800	-3.76457500	0.91504300
H	-1.19350800	-4.74137500	2.63619900
H	-0.82764800	-1.16033600	4.98892600
H	0.61101500	-0.16589400	3.25051300
H	-3.39534700	-0.85580100	-3.17041000
H	-4.29372100	0.52711800	-5.01278000
H	-3.26922000	4.09018100	-2.83389900
H	-2.34436400	2.71264200	-1.01428800
H	-2.24900900	-1.50318800	1.90078900
H	-3.65896200	-1.24985800	3.90276300
H	-5.93826700	1.80239900	1.90220000
H	-4.52048000	1.54685100	-0.09963700
H	1.19383300	-0.50751400	-3.17451100
H	6.48322500	0.80513100	2.77694200
H	-4.24203600	3.00637000	-4.85326100
H	-1.73571000	-3.45793600	4.69570600
H	-5.51058500	0.42249000	3.92586400
C	0.74115400	1.71674700	-0.41787900
C	-0.12931100	2.55049600	0.40783600
C	-0.34448300	3.91292700	0.10262800
C	-0.87379100	2.00953400	1.47718500
C	-1.31640900	4.65374500	0.76911500
H	0.21598400	4.36403000	-0.70948900
C	-1.81791600	2.76325700	2.16018400
H	-0.70886900	0.97927900	1.75956400
C	-2.07867500	4.09226600	1.80302600
H	-1.49342300	5.68626700	0.47815600
H	-2.38875300	2.29784700	2.95877600
C	2.03920700	2.31317900	-0.74508700
C	2.73029900	3.15356400	0.15247900
C	2.67927800	2.00495500	-1.96264400
C	4.00327900	3.62950500	-0.13937400
H	2.26907900	3.39738900	1.10317200
C	3.94071300	2.50276600	-2.26243600
H	2.15428400	1.36837300	-2.66394800
C	4.63074300	3.31750500	-1.35183000
H	4.52601900	4.24595300	0.58732000
H	4.40634600	2.25322400	-3.21254900
C	-3.14309900	4.88253700	2.51984000
H	-4.06501200	4.29850600	2.61328600
H	-2.82793500	5.15075600	3.53572400
H	-3.37870100	5.81024900	1.99119600
C	5.99729300	3.86161700	-1.68140400
H	5.92289300	4.80974300	-2.22856600
H	6.57958300	4.05450300	-0.77576900
H	6.56261600	3.16873000	-2.31178700

Complex 11

Energy: -4237.611362 Hartree

Ni	-0.32325600	0.43815600	-0.09775300
P	1.56699200	1.21473900	0.49702200
P	-0.05201800	-1.46160400	-0.96901700
C	2.54565000	2.12597400	-0.78309300
C	3.37401100	3.20300000	-0.44009100
C	4.06010300	3.92649900	-1.41338400

C	3.91278900	3.59189200	-2.75778000
C	3.08947200	2.52605700	-3.11521600
C	2.40915600	1.77901500	-2.14436700
C	1.57178100	0.62455400	-2.59462400
C	2.18266300	-0.73327800	-2.47878100
C	3.46188100	-0.94018400	-3.01559600
C	4.08097100	-2.18522900	-2.94429200
C	3.42479700	-3.25142700	-2.32901800
C	2.15803500	-3.05816200	-1.78329800
C	1.52867700	-1.80669600	-1.83403200
C	1.42252600	2.46941800	1.84593200
C	2.44616600	2.72378800	2.77142500
C	2.28833500	3.70579700	3.74871500
C	1.10925800	4.45074700	3.80879500
C	0.08755400	4.20548800	2.89107800
C	0.24067800	3.21767400	1.91910700
C	2.76543600	0.03098400	1.22634200
C	3.83153700	-0.49282900	0.48577800
C	4.62949200	-1.50716200	1.01460600
C	4.39012900	-1.99199600	2.30126100
C	3.33966700	-1.46193500	3.05350400
C	2.52353500	-0.47052100	2.51490100
C	-1.30983700	-1.98228000	-2.20697300
C	-0.98946300	-2.74850400	-3.33632700
C	-1.97672100	-3.09305500	-4.25975200
C	-3.29666500	-2.68502900	-4.06350500
C	-3.62528600	-1.92541300	-2.94002900
C	-2.63776900	-1.56994300	-2.02341700
C	-0.18053600	-2.71462600	0.35765700
C	0.93186100	-3.00217300	1.16216200
C	0.79622500	-3.80965500	2.28850400
C	-0.45195500	-4.32766500	2.63686500
C	-1.56418100	-4.03415000	1.84820900
C	-1.43190700	-3.23292900	0.71655600
C	0.38125000	0.83481400	-3.17541400
H	3.46684700	3.49412900	0.59927100
H	4.69559600	4.75661900	-1.12066200
H	4.42829200	4.16010300	-3.52600900
H	-0.21676800	0.01921900	-3.56485700
H	2.95926500	2.26123400	-4.15989200
H	3.97397000	-0.10874900	-3.48892300
H	5.07149300	-2.32124200	-3.36760800
H	3.89860400	-4.22624100	-2.26697800
H	1.65818300	-3.88184400	-1.28562200
H	3.36248300	2.14416000	2.73585100
H	3.08518600	3.88731000	4.46400000
H	-0.83602200	4.77454800	2.93741300
H	-0.56377300	2.99106000	1.22561300
H	4.03464900	-0.11788500	-0.50914600
H	5.44036000	-1.91403100	0.41828700
H	3.14321400	-1.83388300	4.05446200
H	1.69064700	-0.08747600	3.09423300
H	0.03351600	-3.06886700	-3.49957300
H	-1.71297400	-3.68122100	-5.13384200
H	-4.64669400	-1.59247700	-2.78292600
H	-2.88608600	-0.95401700	-1.16794300
H	1.90196900	-2.59411900	0.91085300
H	1.66934100	-4.02171500	2.89762100
H	-2.54225300	-4.41845300	2.11910600
H	-2.30644900	-3.00119500	0.12151600
H	-0.01872400	1.83892100	-3.26747700

H	0.98580200	5.21273500	4.57260900
H	-4.06247100	-2.95157300	-4.78568900
H	5.01670900	-2.77614300	2.71513200
H	-0.55771700	-4.95224200	3.51902100
C	-1.98535500	0.85820300	0.41070900
C	-2.39552600	0.12478500	1.62268600
C	-3.61507900	-0.57960600	1.70011700
C	-1.49904700	-0.03689300	2.70185700
C	-3.89220900	-1.43605100	2.76318300
H	-4.33604600	-0.47313500	0.89555300
C	-1.78800400	-0.87659900	3.76744500
H	-0.57365900	0.52737500	2.68993400
C	-2.98238600	-1.60986600	3.81090400
H	-4.83067800	-1.98562200	2.77698200
H	-1.06992000	-0.98015500	4.57737700
C	-3.01661000	1.63075400	-0.28116500
C	-4.08037000	2.26174000	0.39873900
C	-2.95460900	1.82016600	-1.67940000
C	-5.01127300	3.04462000	-0.27841500
H	-4.15972900	2.14154300	1.47419200
C	-3.90303200	2.57411600	-2.35428000
H	-2.15114100	1.33805200	-2.22506600
C	-4.94731900	3.21191200	-1.66623500
H	-5.81020400	3.52834200	0.27879900
H	-3.83490700	2.67937800	-3.43465200
C	-3.25204500	-2.57755000	4.93398500
H	-2.64668100	-3.48539100	4.81868200
H	-3.00010400	-2.14537500	5.90818300
H	-4.30253300	-2.88086300	4.95978500
C	-5.94672100	4.07069200	-2.39880100
H	-6.21975900	3.63222600	-3.36417400
H	-6.86240900	4.20441100	-1.81570100
H	-5.54025500	5.06944600	-2.60315100

Complex 12

Energy: -4351.004291 Hartree

Ni	-0.23656300	0.03588000	-1.17240400
P	1.44894700	0.89474400	-0.17468100
P	-1.98831800	0.41673000	-0.04079200
O	-0.56175800	0.93310600	-3.89024800
C	1.67665600	0.94156100	1.66252400
C	2.46786400	1.97372500	2.20467800
C	2.72057300	2.08477400	3.56604800
C	2.16989500	1.14632200	4.43620900
C	1.37921500	0.12702800	3.92212200
C	1.11273600	-0.00298200	2.54788700
C	0.20582000	-1.16448700	2.17220100
C	-1.26652600	-0.84098200	2.48378000
C	-1.66263100	-1.20253300	3.79077500
C	-2.91870200	-0.93111100	4.31227100
C	-3.86295400	-0.28460900	3.51826800
C	-3.50570400	0.07652400	2.22901400
C	-2.22181900	-0.16550900	1.69411100
C	1.30778000	2.69932700	-0.55089300
C	1.74629900	3.17056600	-1.79648900
C	1.57979700	4.50869000	-2.14693500
C	0.97675500	5.39879600	-1.25727500
C	0.52589400	4.93512800	-0.02226400
C	0.67672400	3.59389900	0.32298800
C	3.13533900	0.56557000	-0.86737000

C	3.17281100	-0.07477000	-2.11072500
C	4.37365500	-0.22204500	-2.80606500
C	5.55960500	0.26158900	-2.25846800
C	5.54017000	0.86899500	-1.00053900
C	4.33950200	1.01876900	-0.31163900
C	-2.33000600	2.22731700	0.05511200
C	-2.26180500	2.95693500	-1.14478500
C	-2.53534700	4.32111700	-1.16465700
C	-2.84368800	4.99267200	0.01893800
C	-2.85886800	4.29019800	1.22246500
C	-2.60614600	2.91746800	1.24206700
C	-3.54594000	-0.25831800	-0.77668900
C	-4.27636300	0.43257600	-1.75180100
C	-5.39763100	-0.14861500	-2.34366000
C	-5.80821100	-1.42881800	-1.97133700
C	-5.08191400	-2.12634600	-1.00545000
C	-3.95684300	-1.55054900	-0.41966900
C	0.65732000	-2.29361900	1.18765800
C	-0.39337900	-2.88618900	0.29097200
C	-1.18810900	-3.97016700	0.66268200
C	-2.09296500	-4.54168000	-0.23750900
C	-2.23243300	-4.04764700	-1.53663800
C	-1.44431400	-2.94977900	-1.90397400
C	-0.55509200	-2.37034700	-1.00581800
C	2.07538600	-2.35270000	0.63574800
C	3.18287300	-1.88834500	1.36709600
C	4.48506400	-2.07867800	0.92036800
C	4.75822000	-2.75429300	-0.27221500
C	3.66472700	-3.23989700	-0.99068000
C	2.35802700	-3.05068000	-0.54790900
C	-3.19973400	-4.65588400	-2.51822000
C	6.17030400	-2.93844500	-0.76024600
C	0.59018900	-2.54424000	2.66905800
C	-0.39231200	0.44811100	-2.84365000
H	2.87028300	2.72767500	1.53981000
H	3.33595000	2.89691800	3.94044700
H	2.35036500	1.20733300	5.50510600
H	0.95584000	-0.60372900	4.60140100
H	-0.95245900	-1.72081400	4.42251200
H	-3.15780300	-1.23299800	5.32748100
H	-4.85966100	-0.07175900	3.89157900
H	-4.24284700	0.56106300	1.59887300
H	2.22449200	2.48929700	-2.49141000
H	1.92932000	4.85635000	-3.11454600
H	0.85415100	6.44335800	-1.52749100
H	0.03573100	5.61093000	0.67024900
H	0.30153200	3.24659500	1.27731700
H	2.24367200	-0.44354200	-2.53137700
H	4.37776800	-0.71490000	-3.77353500
H	6.49476300	0.16041400	-2.80140800
H	6.46300300	1.23140900	-0.55698200
H	4.34868100	1.48389100	0.66575900
H	-1.97232500	2.45864200	-2.06268600
H	-2.47985100	4.86357700	-2.10293300
H	-3.04765500	6.05904700	0.00526100
H	-3.07192100	4.80779200	2.15331000
H	-2.62427700	2.38928200	2.18723200
H	-3.98498100	1.43381900	-2.04356700
H	-5.95555000	0.40674900	-3.09174700
H	-6.68602800	-1.87681100	-2.42719300
H	-5.38678800	-3.12467700	-0.70609700

H	-3.40165300	-2.10728000	0.32527000
H	-1.08662500	-4.40249300	1.65204200
H	-2.68927100	-5.39412600	0.07666100
H	-1.52947800	-2.53266600	-2.90295300
H	0.19942500	-1.66208500	-1.40051000
H	3.04577900	-1.36977200	2.30511500
H	5.30700300	-1.69183800	1.51655800
H	3.83273100	-3.78795000	-1.91399600
H	1.55561100	-3.48524500	-1.12710500
H	-3.69158800	-5.53854400	-2.10039000
H	-2.69205200	-4.95700800	-3.44089400
H	-3.97532400	-3.93316600	-2.79286200
H	6.53197900	-2.02402800	-1.24299400
H	6.23750300	-3.74896800	-1.49143500
H	6.85251700	-3.16602800	0.06505300
H	1.53742000	-2.67761400	3.17815100
H	-0.20757100	-3.16805900	3.05150700

Complex 13
Energy: -4464.394208 Hartree

Ni	0.04392700	0.47019600	-1.38289900
P	1.80913300	0.52265300	-0.08143100
P	-1.65124800	1.22284200	-0.21161000
O	0.61176700	2.49121500	-3.40818500
C	1.71438000	0.45699700	1.76684500
C	2.72059000	1.17866100	2.44787600
C	2.75431900	1.31196400	3.82752600
C	1.74671100	0.71710700	4.58228100
C	0.76401400	-0.01534200	3.93440500
C	0.71408900	-0.18559000	2.53458800
C	-0.46949800	-1.07294100	2.11257800
C	-1.80333000	-0.33711800	2.27036500
C	-2.50140300	-0.58415300	3.47055100
C	-3.65654700	0.09290200	3.83574500
C	-4.16353800	1.08058600	2.99694800
C	-3.49716100	1.34617900	1.81042500
C	-2.33595500	0.65067300	1.41210400
C	2.53149400	2.21466600	-0.33025500
C	3.52359700	2.43828600	-1.29365100
C	3.94497400	3.73558900	-1.58791500
C	3.38394800	4.82718600	-0.92747700
C	2.39443300	4.61257400	0.03326000
C	1.97102200	3.31946700	0.32551100
C	3.27467000	-0.47698000	-0.57835100
C	3.27159900	-0.99713000	-1.87629700
C	4.42560700	-1.56752600	-2.41625200
C	5.59224400	-1.63356500	-1.65832000
C	5.59564500	-1.14303200	-0.35029900
C	4.44676500	-0.56715200	0.18425900
C	-1.47346400	3.02208200	0.19395200
C	-1.44924800	3.98742100	-0.82099200
C	-1.21961500	5.32886700	-0.52451300
C	-0.99358600	5.73139700	0.79206700
C	-0.99276900	4.77696700	1.80843800
C	-1.22983300	3.43445700	1.51322600
C	-3.15309800	1.12185600	-1.30854800
C	-3.04959200	1.61465800	-2.62105200
C	-4.10673500	1.49822800	-3.51956900
C	-5.28275500	0.84893500	-3.14280900
C	-5.38399400	0.31656300	-1.85989800

C	-4.33245200	0.45219700	-0.95182700
C	-0.32883700	-2.38483100	1.28265200
C	-1.55782300	-2.97735600	0.62588500
C	-1.82388200	-4.33519700	0.86655300
C	-2.90690800	-4.97300400	0.27517400
C	-3.77521600	-4.27711800	-0.57925000
C	-3.51057100	-2.92796500	-0.81621800
C	-2.41924600	-2.29123300	-0.22383700
C	0.97432500	-2.84372400	0.67011600
C	2.17441900	-2.87193000	1.39406600
C	3.31363200	-3.48299700	0.87771900
C	3.31012100	-4.08087900	-0.38440500
C	2.12507500	-4.01734900	-1.12427900
C	0.98062500	-3.41968200	-0.60795200
C	-4.96178600	-4.96605200	-1.20536600
C	4.52688700	-4.79154300	-0.91751900
C	-0.41604100	-2.43507800	2.78275200
C	0.38990000	1.71675300	-2.57529100
H	3.48070100	1.68275400	1.86515300
H	3.54774400	1.88203400	4.30047800
H	1.72772000	0.81275700	5.66359900
H	-0.00458500	-0.49097600	4.53174300
H	-2.11408100	-1.32748400	4.15396000
H	-4.14522500	-0.14397400	4.77588300
H	-5.05618000	1.63883300	3.26026700
H	-3.88298500	2.12497200	1.16288300
H	3.96890500	1.60332400	-1.82096400
H	4.71544300	3.88887900	-2.33776700
H	3.71227800	5.83568000	-1.16021200
H	1.93934600	5.44930400	0.55271700
H	1.20161000	3.17098600	1.07047200
H	2.36257600	-0.93904500	-2.46441600
H	4.40727100	-1.96063400	-3.42812900
H	6.49194800	-2.07325900	-2.07787600
H	6.49726800	-1.20463900	0.25205800
H	4.46348900	-0.19231000	1.20062100
H	-1.60265400	3.69892400	-1.85180200
H	-1.21077400	6.05912500	-1.32766000
H	-0.81406400	6.77726500	1.02167900
H	-0.80581100	5.07285600	2.83643500
H	-1.21794600	2.70730200	2.31621200
H	-2.12640500	2.06387500	-2.96297100
H	-4.00009800	1.89863100	-4.52299400
H	-6.10221900	0.74545000	-3.84722700
H	-6.28238500	-0.21286400	-1.55644500
H	-4.43465100	0.01162700	0.03058200
H	-1.15855600	-4.89473600	1.51782600
H	-3.08350100	-6.02688500	0.47561400
H	-4.15435900	-2.35757500	-1.47891600
H	-2.23626100	-1.25430100	-0.44296200
H	2.23659600	-2.41811900	2.37586000
H	4.22481200	-3.48935600	1.46870500
H	2.09012300	-4.45360500	-2.11907000
H	0.07318500	-3.42732100	-1.19470200
H	-4.68243300	-5.93647600	-1.62873400
H	-5.39524400	-4.36044700	-2.00566500
H	-5.75005000	-5.15248700	-0.46574700
H	4.60157900	-4.68921200	-2.00368900
H	4.48985200	-5.86428400	-0.68989200
H	5.44398000	-4.38998100	-0.47917400
H	0.47536100	-2.68388200	3.34475300

H	-1.32690700	-2.85770800	3.18697200
C	-0.37806300	-1.00426500	-2.25800800
O	-0.67620100	-1.82944400	-3.01519000

Complex 14
Energy: -3751.003581 Hartree

Ni	0.00482000	0.33525200	-0.53635800
P	2.05344600	-0.10078000	-0.05574200
P	-2.11039700	0.08445100	-0.13862000
C	2.45081600	-1.41832500	-1.19433800
C	3.71932700	-1.82726600	-1.59861200
C	3.85170200	-2.83584300	-2.55190700
C	2.68254800	-3.39281700	-3.09185500
C	1.41609300	-2.99447100	-2.68461400
C	1.23489700	-1.98584400	-1.69406100
C	-0.02369700	-1.42085900	-1.23997100
C	-1.24115900	-2.20826000	-1.17535400
C	-1.37453000	-3.60091900	-1.44671600
C	-2.60621100	-4.23845300	-1.40329000
C	-3.78173900	-3.55730700	-1.05453600
C	-3.68129500	-2.21394900	-0.69898000
C	-2.44968600	-1.56542200	-0.75665600
C	3.26378900	1.24399700	-0.30647800
C	3.82688300	1.97899600	0.74263200
C	4.60860300	3.10495200	0.47419600
C	4.83234700	3.50748200	-0.84296500
C	4.27428700	2.77480900	-1.89586000
C	3.49218100	1.65421100	-1.63049100
C	2.27743800	-0.66919700	1.66261700
C	2.91510400	-1.88190500	1.95247600
C	3.01772100	-2.31900800	3.27305700
C	2.49556700	-1.54795400	4.31252300
C	1.85800900	-0.33787500	4.02954800
C	1.74007500	0.09478900	2.71061400
C	-3.29573800	1.26829900	-0.85143500
C	-4.06166500	0.93351500	-1.97528100
C	-4.88060200	1.88967800	-2.57655400
C	-4.93966400	3.18672700	-2.06730100
C	-4.16751600	3.53139800	-0.95525300
C	-3.34434300	2.58171800	-0.35573800
C	-2.47123900	-0.00203800	1.65369100
C	-1.56006200	-0.71597300	2.45023000
C	-1.77491700	-0.85144800	3.81755600
C	-2.89871200	-0.27107000	4.41302600
C	-3.81024100	0.43285900	3.62706100
C	-3.60354900	0.56428000	2.25244300
H	4.59837200	-1.34254700	-1.18078800
H	4.83066800	-3.16389700	-2.88431700
H	2.76450200	-4.15160600	-3.86645000
H	0.55238400	-3.41740800	-3.18105800
H	-0.49324800	-4.19054500	-1.66152300
H	-2.65213100	-5.30224000	-1.62413600
H	-4.73633100	-4.07173600	-1.02347500
H	-4.55795200	-1.67011900	-0.35553300
H	3.65999500	1.67048700	1.76900900
H	5.04809300	3.66282600	1.29587900
H	4.45085200	3.07809500	-2.92343800
H	3.05310000	1.08594400	-2.44480600
H	3.31238600	-2.48189800	1.14088200
H	3.50527900	-3.26459800	3.48967800

H	1.43362500	0.25757300	4.83190300
H	1.20601500	1.01357700	2.48740900
H	-4.01173300	-0.07414500	-2.37274500
H	-5.47382500	1.61877000	-3.44470900
H	-4.20659400	4.54126300	-0.55739300
H	-2.73964100	2.85297700	0.50424300
H	-0.68436000	-1.16244700	1.99031500
H	-1.05716800	-1.40237400	4.41733500
H	-4.69033000	0.87892100	4.08120300
H	-4.32390400	1.10524100	1.64889900
H	5.44444900	4.38003800	-1.05046000
H	-5.58015900	3.92778100	-2.53574700
H	2.57724600	-1.89196700	5.33906400
H	-3.06232100	-0.36926700	5.48196200
N	0.17936300	2.16587500	-0.41449000
C	0.53993000	3.26985000	-0.40751500
C	1.07555300	4.61954400	-0.44415800
H	0.70005700	5.15432600	-1.32185500
H	0.79068800	5.17553000	0.45410600
H	2.16791700	4.56480200	-0.49910100

Complex 15

Energy: -4347.158229 Hartree

Ni	-0.49585600	0.07989800	-0.86996000
P	-0.88918100	2.19418500	-0.36073300
P	-2.04114000	-1.38045700	-0.42968400
N	0.95702700	-0.09894900	-1.73577600
N	2.14750400	-0.23611200	-1.62715800
C	-1.03560100	1.85842000	1.43174700
C	-1.73053500	2.69293000	2.31450700
C	-1.85259900	2.35678700	3.66053300
C	-1.26374900	1.18129800	4.12646300
C	-0.57294700	0.34679500	3.24886400
C	-0.44382500	0.65889800	1.88693900
C	0.29466400	-0.26969000	0.91186300
C	0.03057200	-1.70963600	1.32508600
C	0.82748200	-2.41888600	2.23315200
C	0.50320800	-3.71796700	2.61677200
C	-0.64611100	-4.33557400	2.12095100
C	-1.46598500	-3.64097500	1.23392100
C	-1.12938000	-2.34476200	0.83518200
C	-2.29045500	3.32544800	-0.68016200
C	-2.13875600	4.64711400	-1.11308400
C	-3.26397600	5.43321500	-1.37161800
C	-4.54397800	4.91055600	-1.18966200
C	-4.69980100	3.59160800	-0.75296900
C	-3.58198800	2.80124600	-0.50862300
C	0.61559200	3.24377900	-0.47608200
C	1.41717300	3.13160600	-1.62120700
C	2.63312700	3.80776100	-1.70293900
C	3.06199000	4.60396100	-0.64131700
C	2.26589400	4.73163500	0.49835100
C	1.04943400	4.05536100	0.58230900
C	-3.63318400	-0.90693400	0.33457600
C	-4.74440500	-0.62096600	-0.47446700
C	-5.89284600	-0.05605800	0.07760900
C	-5.93908500	0.25121600	1.43935600
C	-4.83649400	-0.02773500	2.24809700
C	-3.69405000	-0.61170000	1.70371000
C	-2.47441000	-2.61635300	-1.70797700

C	-3.55736900	-3.49951500	-1.59383100
C	-3.78167100	-4.46500700	-2.57419900
C	-2.92679600	-4.55895000	-3.67426200
C	-1.85081700	-3.67897400	-3.79731100
C	-1.62888500	-2.70782200	-2.82196100
C	1.76489500	0.16570600	0.86831900
C	2.72797900	-0.38601700	-0.21082400
C	4.04023200	0.39361100	-0.25933700
C	4.35544500	1.44491000	0.60180900
C	5.55725500	2.14451500	0.46580800
C	6.47707500	1.82058800	-0.53125700
C	6.15815000	0.76124100	-1.39451200
C	4.96771700	0.06031300	-1.25908400
C	7.77590800	2.57295000	-0.67990100
C	2.97267300	-1.88493200	-0.01775900
C	2.20264000	-2.85144800	-0.67442200
C	2.34653800	-4.20104700	-0.37008300
C	3.26019500	-4.63375100	0.59696800
C	4.05341100	-3.66793500	1.22509300
C	3.91848000	-2.31535000	0.91744500
C	3.35847300	-6.09249700	0.96529000
H	-2.20106300	3.59445000	1.93606700
H	-2.40359400	3.00344400	4.33663000
H	-1.34832100	0.90585800	5.17379900
H	-0.14438200	-0.57519400	3.62434300
H	1.73284700	-1.96879500	2.62071000
H	1.15868800	-4.25260100	3.29757700
H	-0.89482000	-5.35088400	2.41427500
H	-2.35050600	-4.11876900	0.82482000
H	-1.14455100	5.06138800	-1.24452900
H	-3.13768000	6.45757600	-1.70972100
H	-5.41634400	5.52606200	-1.38778400
H	-5.69112700	3.17355700	-0.60576500
H	-3.70563100	1.77741700	-0.17472900
H	1.10369700	2.48007400	-2.42980100
H	3.25737100	3.68915800	-2.58261200
H	4.02086800	5.10978100	-0.69496400
H	2.59769000	5.34896600	1.32805400
H	0.44711600	4.13463600	1.48111800
H	-4.70482800	-0.83080300	-1.53869500
H	-6.74762100	0.15434800	-0.55836700
H	-6.82822600	0.70541100	1.86565300
H	-4.85954800	0.21287000	3.30640000
H	-2.83974100	-0.81493300	2.33810400
H	-4.22971500	-3.42308300	-0.74534800
H	-4.62335100	-5.14461300	-2.47952200
H	-3.10392400	-5.31172200	-4.43642700
H	-1.18922200	-3.74219100	-4.65578500
H	-0.80275200	-2.00800500	-2.91502700
H	1.76515500	1.25294100	0.78281700
H	2.25182900	-0.06131900	1.82558100
H	3.66911800	1.74854900	1.38175800
H	5.77330200	2.96225600	1.14825100
H	6.85424500	0.48639800	-2.18317500
H	4.73330600	-0.75056000	-1.94000800
H	7.82559600	3.42220100	0.00695100
H	7.90075600	2.95587600	-1.69882700
H	8.63706100	1.92671100	-0.47321200
H	1.46698200	-2.55155200	-1.40928300
H	1.71952300	-4.92860500	-0.87840800
H	4.78592400	-3.97737400	1.96622500

H	4.54506500	-1.58298900	1.41695700
H	2.47617100	-6.40902700	1.53491700
H	4.24058600	-6.29337800	1.57934300
H	3.41208600	-6.72908200	0.07608400

Complex 16
Energy: -4347.100737 Hartree

Ni	0.32558000	0.25153200	-0.48673300
P	-1.45882200	1.58636100	-0.56133600
P	-0.81404600	-1.51294100	0.34669600
N	1.16864300	1.86987200	-0.97161600
N	2.36931800	1.95354400	-0.79447100
C	-1.21229200	2.14996300	1.18619500
C	-2.04003900	3.16028300	1.70263700
C	-1.85262000	3.67154200	2.98148000
C	-0.79396800	3.19254700	3.75221400
C	0.05372400	2.21769600	3.23513800
C	-0.11775300	1.66210300	1.95453300
C	0.86090900	0.58458600	1.46226900
C	0.76998100	-0.65854100	2.34938000
C	1.40933900	-0.74834400	3.60131500
C	1.25383200	-1.85797000	4.42628600
C	0.42926900	-2.91615800	4.03582400
C	-0.20416700	-2.86009300	2.80027000
C	0.01127100	-1.76980000	1.94549100
C	-3.20096000	1.05976200	-0.81846500
C	-4.29605400	1.36270500	-0.00431500
C	-5.58083100	0.97301400	-0.37955700
C	-5.79266400	0.28567600	-1.57463000
C	-4.70618800	-0.03205800	-2.38947200
C	-3.42060700	0.34519700	-2.00727600
C	-1.44769400	3.19300900	-1.46081000
C	-2.23663900	3.39344600	-2.60219100
C	-2.16731000	4.59055100	-3.31319600
C	-1.30617000	5.60614500	-2.89843800
C	-0.51730500	5.41648200	-1.76389400
C	-0.58504900	4.22196300	-1.05032900
C	-2.55080700	-1.41186800	0.98930300
C	-3.63240400	-2.05427500	0.37648200
C	-4.90339500	-2.00087700	0.94613800
C	-5.11571300	-1.30547100	2.13530000
C	-4.04628700	-0.65308300	2.74796100
C	-2.77480900	-0.70727700	2.18349000
C	-0.88525300	-3.06717000	-0.61977000
C	-0.49793000	-4.32980100	-0.15894100
C	-0.69241400	-5.46035900	-0.95284800
C	-1.26455500	-5.34318500	-2.21966300
C	-1.61627100	-4.08248600	-2.70334600
C	-1.41996700	-2.95292400	-1.91158800
C	2.34015100	1.02610300	1.39756500
C	2.92280000	0.76932300	-0.03009400
C	4.43693500	0.78870500	-0.09111600
C	5.16109500	1.72983100	0.65374300
C	6.54973400	1.78613700	0.58589000
C	7.27062600	0.90541400	-0.22972000
C	6.54675100	-0.02232200	-0.98411100
C	5.15541600	-0.07999700	-0.91994600
C	8.77782400	0.94506300	-0.27440400
C	2.30024100	-0.49712200	-0.64474600
C	1.83974800	-0.53771800	-1.99779300

C	1.73316300	-1.77684500	-2.67348900
C	2.06911400	-2.97386700	-2.07486000
C	2.48943200	-2.93751200	-0.71878200
C	2.61206100	-1.75421000	-0.03355400
C	2.06832600	-4.27212700	-2.83693500
H	-2.81971700	3.57706300	1.07428200
H	-2.50875300	4.44813000	3.36216500
H	-0.61776900	3.58822900	4.74837600
H	0.88145900	1.87317800	3.84441700
H	2.03679300	0.06697700	3.94447200
H	1.75889400	-1.88776300	5.38738300
H	0.27871100	-3.76858000	4.69084300
H	-0.88421200	-3.65215200	2.50827900
H	-4.14823800	1.87576500	0.93674600
H	-6.41939000	1.20211300	0.27069200
H	-6.79651000	-0.01196300	-1.86181400
H	-4.85735300	-0.57794300	-3.31598700
H	-2.56899500	0.09661000	-2.63518900
H	-2.91906100	2.62073500	-2.93555400
H	-2.79289200	4.72894600	-4.19015800
H	-1.25282900	6.53851600	-3.45252300
H	0.15752100	6.19940900	-1.43118300
H	0.02838900	4.09086100	-0.16898800
H	-3.48844800	-2.60612500	-0.54365600
H	-5.72922300	-2.50509700	0.45407900
H	-6.10635900	-1.26953200	2.57835000
H	-4.19589800	-0.10430600	3.67289400
H	-1.95528200	-0.20896200	2.68477600
H	-0.03018800	-4.43405000	0.81079000
H	-0.39138200	-6.43487000	-0.57997800
H	-1.41701700	-6.22555900	-2.83330200
H	-2.03930300	-3.97743900	-3.69775800
H	-1.68304300	-1.97157400	-2.29254100
H	2.47144100	2.08780200	1.62623300
H	2.95763000	0.47063600	2.10676200
H	4.63421200	2.43692400	1.28493300
H	7.08409100	2.52874200	1.17324400
H	7.07830500	-0.71046300	-1.63652100
H	4.62758900	-0.80800800	-1.52421300
H	9.16035500	0.52549500	-1.20923000
H	9.21520600	0.36469600	0.54761500
H	9.15352400	1.96878300	-0.18277600
H	1.83575100	0.35687600	-2.60506600
H	1.42412700	-1.76956000	-3.71556600
H	2.74608400	-3.86957400	-0.22205400
H	2.98248500	-1.76151600	0.98510200
H	1.47098100	-4.19793900	-3.74898200
H	1.65953900	-5.08764800	-2.23600900
H	3.08809300	-4.55668700	-3.12640000

Complex 17
Energy: -4347.172437 Hartree

Ni	-0.14183700	0.48874500	-0.05687000
P	-1.82099800	-1.05543500	-0.08933400
P	1.66208300	-0.90897900	-0.27181400
N	0.27421500	1.28710600	-1.99712800
N	-0.83096600	1.07271800	-2.05676200
C	-1.68645800	-1.57560700	1.64769300
C	-2.48099400	-2.59117000	2.20308000
C	-2.32344100	-2.97154900	3.53024900

C	-1.36892500	-2.31815600	4.31254300
C	-0.59725100	-1.29325800	3.77296400
C	-0.73960200	-0.88454400	2.43478000
C	0.09448500	0.26678000	1.87170300
C	1.55441700	0.08850300	2.25109300
C	2.06884200	0.44635100	3.50729100
C	3.40344500	0.21619200	3.82970900
C	4.25872000	-0.39154100	2.90574900
C	3.76764600	-0.75621900	1.65622900
C	2.43305700	-0.49323900	1.32673000
C	-1.45028900	-2.51360200	-1.14876000
C	-1.39125300	-3.83441200	-0.69175800
C	-1.04360400	-4.86909900	-1.55955500
C	-0.75628800	-4.60293300	-2.89699900
C	-0.80675000	-3.28931000	-3.36424900
C	-1.13935300	-2.25275400	-2.49428800
C	-3.62495400	-0.81419700	-0.34712700
C	-4.15761300	-0.83424600	-1.64476200
C	-5.49465400	-0.51498500	-1.86840100
C	-6.32424500	-0.16491300	-0.80183500
C	-5.80450900	-0.14064300	0.49242300
C	-4.46679100	-0.46195100	0.71934900
C	1.82249200	-2.74618800	-0.21919300
C	2.34825300	-3.53173700	-1.25014300
C	2.42380000	-4.91887600	-1.11559000
C	1.98203500	-5.54116800	0.05008100
C	1.46046400	-4.76480500	1.08648800
C	1.37821400	-3.38264100	0.95337400
C	2.78703000	-0.31478000	-1.57659300
C	3.73859700	0.67951900	-1.31377600
C	4.46998100	1.24283700	-2.35830900
C	4.25694600	0.83156100	-3.67361700
C	3.28383300	-0.13150500	-3.94712600
C	2.54156600	-0.68924000	-2.90891400
C	-0.46922000	1.66764200	2.16214300
C	-0.28532800	2.33620700	0.79097900
C	-1.49369700	3.09337700	0.31907300
C	-2.75660500	2.47417700	0.29395900
C	-3.90287300	3.13568800	-0.12312800
C	-3.85354500	4.47318200	-0.53636000
C	-2.61297900	5.11371900	-0.48079100
C	-1.46185600	4.44577200	-0.06337800
C	-5.08697100	5.18229200	-1.03597800
C	1.03959100	2.99106000	0.54846600
C	1.36350300	3.55799600	-0.70645500
C	2.62055500	4.07397600	-0.98521800
C	3.64059600	4.07465500	-0.02417100
C	3.32378400	3.56309400	1.23539600
C	2.05715600	3.05253800	1.51666800
C	5.00516900	4.63751300	-0.33159400
H	-3.23387200	-3.07324700	1.58772000
H	-2.93564700	-3.76285000	3.95126100
H	-1.23085500	-2.60093700	5.35203700
H	0.13445600	-0.80512600	4.40357300
H	1.42488400	0.94017100	4.22886200
H	3.78082700	0.51439300	4.80344400
H	5.29855500	-0.57288700	3.15905300
H	4.41940900	-1.22615300	0.92634600
H	-1.58420200	-4.05775200	0.34938300
H	-0.98032700	-5.88400600	-1.18122300
H	-0.48091700	-5.41116100	-3.56732600

H	-0.57745000	-3.06813300	-4.40250700
H	-1.14783200	-1.23217900	-2.86096700
H	-3.52779900	-1.10559300	-2.48451900
H	-5.88916700	-0.53860100	-2.87975300
H	-7.36551800	0.08610800	-0.97826400
H	-6.43977700	0.12853900	1.33096700
H	-4.07476000	-0.43247900	1.72977500
H	2.72151700	-3.06849400	-2.15450000
H	2.83846200	-5.51103800	-1.92601300
H	2.04755600	-6.62008900	0.15306500
H	1.11604600	-5.23438400	2.00309300
H	0.97416100	-2.79563000	1.76971800
H	3.88577700	1.03953700	-0.30293300
H	5.20123900	2.01414800	-2.13872500
H	4.82979900	1.27240300	-4.48365000
H	3.09099400	-0.43690400	-4.97120600
H	1.75163700	-1.39917500	-3.13520400
H	-1.53346600	1.59518400	2.39093800
H	-0.00696000	2.19744900	3.01024600
H	-2.84387900	1.44477200	0.60612500
H	-4.84675600	2.59653800	-0.13519700
H	-2.54127900	6.16176100	-0.76286700
H	-0.52944100	4.99461100	-0.01552000
H	-5.28682300	4.94367800	-2.08844400
H	-4.98081900	6.26875000	-0.96291700
H	-5.97530200	4.88959200	-0.46674500
H	0.61338600	3.58681700	-1.48535000
H	2.81940000	4.47374800	-1.97661100
H	4.07727100	3.56088400	2.01971800
H	1.87579600	2.68414000	2.51357700
H	5.76806500	4.22139600	0.33331700
H	5.02891800	5.72825900	-0.21181900
H	5.30232500	4.42436300	-1.36398500

Complex 18
Energy: -4237.588695 Hartree

Ni	1.07308000	-0.42472400	0.80897900
P	-3.55042500	-0.01084700	-1.10094200
P	2.32751600	1.22106300	0.21473400
C	-2.77706500	0.77038900	0.39422400
C	-3.60615600	1.40303200	1.33620500
C	-3.08881900	2.03892600	2.46101300
C	-1.70970400	2.05579900	2.65350200
C	-0.87924300	1.43749100	1.72476900
C	-1.37243700	0.77681800	0.58260000
C	-0.36543000	0.15286100	-0.37295500
C	0.10584200	1.17439800	-1.38678500
C	-0.65339300	1.52020100	-2.51546800
C	-0.27030900	2.57481400	-3.34222900
C	0.87181200	3.32527000	-3.04932200
C	1.65702200	2.97770200	-1.95317300
C	1.30328300	1.88321000	-1.15281100
C	-5.05325100	1.04363400	-1.29124000
C	-6.35594100	0.62213400	-0.99782300
C	-7.44059900	1.47539000	-1.21234800
C	-7.23735600	2.75971100	-1.71460000
C	-5.94098400	3.18901800	-2.01028800
C	-4.86012600	2.33542400	-1.80876400
C	-4.25614500	-1.56804700	-0.40141400
C	-4.30190000	-1.86709700	0.96768400

C	-4.81369000	-3.08817900	1.40954000
C	-5.29799300	-4.02141100	0.49308800
C	-5.26190400	-3.73240900	-0.87231900
C	-4.73478400	-2.52080100	-1.31494200
C	3.87211700	0.59430800	-0.56088000
C	4.36479700	1.04357700	-1.79201500
C	5.53135800	0.49802900	-2.32496600
C	6.22166000	-0.49938800	-1.63522400
C	5.73466400	-0.95814000	-0.41143200
C	4.56655100	-0.41564100	0.12015900
C	2.85497100	2.71902300	1.12900900
C	4.14766000	2.84251900	1.65574900
C	4.48681800	3.94438100	2.43965200
C	3.54086700	4.93432400	2.70920500
C	2.25120700	4.81808000	2.18751500
C	1.90794600	3.71946400	1.40242200
C	-0.68285200	-1.27173400	-0.87514200
H	-4.67873200	1.40463400	1.17441700
H	-3.75581800	2.51927100	3.17043500
H	-1.27580400	2.54603300	3.51983900
H	-0.65239900	-1.37713000	-1.96119800
H	0.19071200	1.45851900	1.90234500
H	-1.56277500	0.96576000	-2.72268800
H	-0.87713900	2.82695300	-4.20730200
H	1.15003000	4.16800900	-3.67474800
H	2.54341000	3.55705100	-1.71273700
H	-6.52337300	-0.37163900	-0.59708500
H	-8.44544200	1.13501900	-0.97947400
H	-5.77439300	4.18775700	-2.40302300
H	-3.85394800	2.67310900	-2.04435400
H	-3.92913200	-1.14813000	1.68810200
H	-4.83452600	-3.30822600	2.47291100
H	-5.63144000	-4.45613500	-1.59256100
H	-4.68767400	-2.30966900	-2.37989900
H	3.82663300	1.80053000	-2.34865500
H	5.89958200	0.85048200	-3.28382600
H	6.25483300	-1.74657400	0.12321200
H	4.17401100	-0.78697700	1.06064600
H	4.89018000	2.07935500	1.44623800
H	5.49402200	4.03186900	2.83626500
H	1.51105400	5.58713100	2.38777300
H	0.90626800	3.64057800	0.99287600
H	-1.64395500	-1.66040800	-0.53819200
H	-8.08175200	3.42254600	-1.87740200
H	7.13024900	-0.92192600	-2.05335500
H	-5.69552800	-4.97059800	0.83912600
H	3.80753000	5.79219400	3.31878400
C	0.48788400	-1.97366000	-0.19581400
C	1.62841100	-2.42407400	-1.02431400
C	2.02423300	-1.71769300	-2.17584600
C	2.36108400	-3.57635400	-0.69342700
C	3.11471800	-2.12602000	-2.93211000
H	2.06000700	-4.16485000	0.16655600
C	3.45539900	-3.97830200	-1.45369500
H	1.49571200	-0.81532700	-2.46127800
C	3.86356200	-3.25535500	-2.57978100
H	4.00142000	-4.87434700	-1.16892200
H	3.40686500	-1.54123700	-3.79985600
C	0.41935900	-2.26100300	1.22535400
C	-0.80698000	-2.39644300	1.95663600
C	1.63373200	-2.09217700	2.00314500

C	-0.81918000	-2.39783000	3.32667900
H	-1.73418300	-2.51716200	1.40909900
C	1.56482200	-2.14219300	3.42163000
H	2.59649100	-2.28771100	1.54569400
C	0.37129100	-2.25773300	4.09916500
H	-1.76723900	-2.52290100	3.84530700
H	2.49326500	-2.08306700	3.98502200
C	5.08261800	-3.65209200	-3.37078800
H	5.92579100	-2.98833800	-3.14287300
H	5.39412700	-4.67521700	-3.14237600
H	4.90272200	-3.58462100	-4.44873900
C	0.29795000	-2.26764400	5.60401200
H	-0.29277200	-1.42368900	5.98113400
H	-0.17775700	-3.18179500	5.97965000
H	1.29386900	-2.20114300	6.05058400

Complex 19
Energy: -4237.6051 Hartree

Ni	-1.93460000	0.59247200	0.27200700
P	2.97632100	-0.03091000	-1.26229400
P	-2.29737300	-1.51244200	0.41756500
C	2.22404400	-0.11298800	0.42173700
C	3.05778900	-0.18922800	1.54963400
C	2.54508900	-0.14493900	2.84162700
C	1.17101300	-0.01181900	3.02167900
C	0.34019300	0.04881900	1.91196400
C	0.82547500	-0.00857800	0.59711600
C	-0.17681600	0.05114200	-0.54456700
C	-0.33447900	-1.18218200	-1.41439400
C	0.35969800	-1.43032200	-2.59969300
C	0.13975100	-2.60575100	-3.31985800
C	-0.79666100	-3.54120600	-2.88098800
C	-1.54917000	-3.28026000	-1.73557900
C	-1.32463700	-2.10539500	-1.01597900
C	3.28181700	-1.81654200	-1.61526200
C	3.83381900	-2.15648000	-2.86021600
C	3.96244800	-3.48990100	-3.24261900
C	3.52294800	-4.50696100	-2.39222700
C	2.97429000	-4.17992900	-1.15265200
C	2.86092300	-2.84547400	-0.76287000
C	4.66536600	0.59719500	-0.87267700
C	5.79536400	-0.21602700	-0.71543200
C	7.03696300	0.34831000	-0.41777700
C	7.16392300	1.72889000	-0.26517900
C	6.04440700	2.54866000	-0.42361000
C	4.80836900	1.98735000	-0.73569900
C	-3.97594900	-2.18488400	0.19972800
C	-4.96644700	-1.32519800	-0.29090600
C	-6.25383800	-1.80523100	-0.52759300
C	-6.56032900	-3.14130500	-0.26588800
C	-5.58046100	-3.99832000	0.24025800
C	-4.29164800	-3.52283000	0.47537700
C	-1.61578300	-2.41865700	1.84918200
C	-0.44829500	-3.18366400	1.74733600
C	0.12508900	-3.74051000	2.88988500
C	-0.46224600	-3.53963500	4.13866100
C	-1.63343700	-2.78499300	4.24418500
C	-2.20714500	-2.22481200	3.10573000
C	-0.71958100	1.24829000	-1.10005800
H	4.12953200	-0.26657200	1.40820200

H	3.21451700	-0.20300300	3.69469700
H	0.74119000	0.04432900	4.01615700
H	-3.16584300	1.14657300	0.91578800
H	-0.72450700	0.16440400	2.07516100
H	1.07391700	-0.69977600	-2.95760400
H	0.70827300	-2.78870500	-4.22589100
H	-0.96259400	-4.45415900	-3.44447500
H	-2.32738400	-3.96838200	-1.42125000
H	4.15398300	-1.36920000	-3.53800600
H	4.39444900	-3.73578100	-4.20825300
H	2.63169700	-4.96562300	-0.48541600
H	2.42852800	-2.59921900	0.20036700
H	5.70136500	-1.29140500	-0.82188100
H	7.90481300	-0.29385400	-0.29880900
H	6.13697100	3.62517100	-0.31498100
H	3.94454100	2.63122300	-0.87696900
H	-4.71876100	-0.28085500	-0.45588200
H	-7.01913300	-1.13436700	-0.90545600
H	-5.82237600	-5.03455000	0.45627400
H	-3.53335800	-4.18276800	0.88550100
H	0.01956100	-3.32664500	0.77959300
H	1.03620200	-4.32485900	2.80433000
H	-2.09502700	-2.62856400	5.21437900
H	-3.10892600	-1.62472100	3.18770000
H	-1.13065900	1.16082500	-2.10388800
H	3.60740200	-5.54597200	-2.69575900
H	-7.56448000	-3.51323800	-0.44564600
H	8.12997600	2.16513200	-0.03013300
H	-0.00997900	-3.96754300	5.02804600
C	-1.14724100	2.36749400	-0.31572500
C	-2.01605000	3.37609800	-0.99207600
C	-3.09079000	2.98295000	-1.80748200
C	-1.77479900	4.74898600	-0.85988900
C	-3.86960900	3.91863600	-2.47525200
H	-0.95269400	5.08562700	-0.23819400
C	-2.56576100	5.68695300	-1.52329600
H	-3.32957100	1.92752200	-1.89687900
C	-3.62529600	5.29339500	-2.34371400
H	-2.34980200	6.74559800	-1.40228300
H	-4.69486400	3.58046100	-3.09744400
C	-0.27916900	2.87108200	0.79199200
C	1.10672800	2.97534900	0.61265200
C	-0.81283800	3.23575400	2.03360200
C	1.93948000	3.35064600	1.66082700
H	1.52686400	2.72104300	-0.35493700
C	0.02202600	3.62105700	3.07946200
H	-1.88652600	3.17547200	2.17987300
C	1.41275800	3.65990900	2.92002900
H	3.01501200	3.37896100	1.50606600
H	-0.41049500	3.87528100	4.04382000
C	-4.50013400	6.30587300	-3.03980800
H	-4.02567400	7.29084400	-3.06659900
H	-4.71685500	6.00661300	-4.07063700
H	-5.46405500	6.41916100	-2.52828800
C	2.32454800	3.98169200	4.07648400
H	2.75380600	3.06364900	4.49731300
H	3.16161900	4.61481600	3.76549300
H	1.78967000	4.49504300	4.88046100

Complex 20
Energy: -4237.572122 Hartree

Ni	0.36669500	-0.39961200	-0.99433400
P	2.18217400	-0.05025500	-0.04257500
P	-1.05001800	1.02252600	-0.30216200
C	2.06152700	-0.71714700	1.67638700
C	3.28957400	-0.71932800	2.37370500
C	3.43502400	-1.22069200	3.65725500
C	2.31822700	-1.77007300	4.27867700
C	1.10891500	-1.79767800	3.60033900
C	0.91559900	-1.27886100	2.29754700
C	-0.59418100	-1.29811000	1.91884200
C	-1.23437900	-0.00611000	2.37347000
C	-1.57534400	0.12664200	3.72802500
C	-2.20800900	1.26321400	4.21771400
C	-2.50675400	2.31061700	3.34662200
C	-2.14853300	2.20646400	2.00707800
C	-1.51018300	1.06292900	1.49369100
C	2.81556600	1.67203900	0.12734200
C	3.24580700	2.28055600	1.31182400
C	3.75299700	3.58119700	1.29583100
C	3.84262700	4.28862400	0.09823300
C	3.38343100	3.70457200	-1.08333600
C	2.85816800	2.41659200	-1.06386900
C	3.70685600	-0.92504900	-0.62752200
C	4.59303000	-0.33026700	-1.53723600
C	5.67267700	-1.04525600	-2.05462700
C	5.88431700	-2.37228600	-1.67914600
C	5.00685300	-2.97514900	-0.77846500
C	3.92907000	-2.26068200	-0.25883400
C	-0.52705200	2.77691700	-0.56604600
C	0.14286400	3.49501000	0.43526000
C	0.56269100	4.80333400	0.21294700
C	0.35160700	5.40981100	-1.02523400
C	-0.28085500	4.69279700	-2.04004900
C	-0.72032600	3.38906700	-1.81159500
C	-2.66818100	1.03874300	-1.19988700
C	-3.90312700	1.38654300	-0.64365500
C	-5.05523000	1.40912100	-1.42867800
C	-4.99385600	1.08630000	-2.78377200
C	-3.76932500	0.73273300	-3.35099000
C	-2.62052100	0.70348300	-2.56281800
C	-1.20002000	-2.65218400	2.24051300
C	-1.35841800	-2.16761400	0.82822600
C	-0.51578700	-2.61860600	-0.32639200
C	-0.66946400	-1.88826700	-1.57868000
C	0.46783800	-1.90654200	-2.44463700
C	1.45032300	-2.94370600	-2.34641900
C	1.40895700	-3.79319700	-1.26312000
C	0.47992800	-3.56632400	-0.21547900
C	2.52468800	-3.04634200	-3.39244200
C	-2.82527800	-1.91836000	0.50087000
C	-3.72853200	-1.24837500	1.34127500
C	-5.08389100	-1.16013500	1.03491500
C	-5.61415300	-1.74368400	-0.11673100
C	-4.73715000	-2.48081700	-0.91612100
C	-3.38524700	-2.58121300	-0.60513200
C	-7.06024900	-1.56962500	-0.49771100
H	4.16568300	-0.33228200	1.86661800
H	4.40129900	-1.19586900	4.15095600
H	2.38274200	-2.18428000	5.28028200
H	0.26147700	-2.24000600	4.10805300

H	-1.35693200	-0.69431500	4.40183300
H	-2.46841600	1.32936400	5.26974000
H	-3.00637900	3.20438800	3.70716100
H	-2.35528100	3.03687400	1.34211500
H	3.18335900	1.74616400	2.25205900
H	4.08288800	4.03883100	2.22410300
H	4.24305200	5.29780600	0.08849400
H	3.40631300	4.26303000	-2.01352600
H	2.45214500	1.98419300	-1.97376800
H	4.45596000	0.70305300	-1.83251400
H	6.35276500	-0.55995300	-2.74865300
H	6.72514900	-2.92870500	-2.08191700
H	5.15956700	-4.00736600	-0.47667600
H	3.25902200	-2.73980400	0.44237200
H	0.33378900	3.03376500	1.39616800
H	1.07456100	5.34062700	1.00419100
H	0.68343600	6.42893000	-1.19923400
H	-0.44802000	5.15208200	-3.01003900
H	-1.23600800	2.85560600	-2.60171400
H	-3.97629500	1.62161800	0.41002500
H	-6.00575200	1.67428100	-0.97529800
H	-5.89359600	1.10387800	-3.39145200
H	-3.70862200	0.47185500	-4.40333400
H	-1.67031400	0.41531500	-3.00317400
H	-0.48810700	-3.44356200	2.44453900
H	-2.11493800	-2.72011400	2.82032400
H	-1.64809900	-1.59279400	-1.93197400
H	0.37912800	-1.41303800	-3.41218600
H	2.14260900	-4.58718800	-1.16668400
H	0.58030200	-4.13330400	0.70560100
H	2.10368900	-3.06126000	-4.40463300
H	3.12848500	-3.94681500	-3.25748700
H	3.19962000	-2.18521200	-3.33035500
H	-3.39411000	-0.77857400	2.25274700
H	-5.73841700	-0.61109900	1.70725500
H	-5.11556500	-2.99479900	-1.79588000
H	-2.75793800	-3.20841800	-1.22424900
H	-7.67503300	-1.30321400	0.36694100
H	-7.47291800	-2.48029700	-0.94298000
H	-7.16679900	-0.76810800	-1.23924700

Complex 21
Energy: -4370.411706 Hartree

Ni	-0.14332000	0.19676700	-1.04643900
P	1.68351800	0.71256800	-0.17656500
P	-1.77093800	0.84873300	0.07518300
C	1.97886100	0.72214300	1.64913400
C	2.97477800	1.59251500	2.13788100
C	3.24508700	1.73714000	3.49152900
C	2.49648900	1.00318200	4.40952900
C	1.51052900	0.14312400	3.94645500
C	1.22417000	-0.02556500	2.57784400
C	0.10205200	-1.02701700	2.30173500
C	-1.28801900	-0.45870500	2.62568500
C	-1.73635300	-0.71833900	3.93827100
C	-2.93692000	-0.24105100	4.44562200
C	-3.76803000	0.52199600	3.63043100
C	-3.35358300	0.79440300	2.33505800
C	-2.12419400	0.34176000	1.81214500
C	1.77956100	2.53375900	-0.51086200

C	2.27039500	3.00202900	-1.73734000
C	2.27540000	4.36605800	-2.02746100
C	1.79562200	5.28577500	-1.09470700
C	1.29507700	4.82825400	0.12422400
C	1.27439400	3.46558700	0.40759300
C	3.28897400	0.16901300	-0.93175900
C	3.19127100	-0.26256500	-2.26140000
C	4.33194200	-0.57053200	-3.00266300
C	5.59213500	-0.47428200	-2.41426400
C	5.69918700	-0.08573400	-1.07816900
C	4.55774000	0.23139200	-0.34331300
C	-1.88908700	2.69053700	0.09133100
C	-1.62311800	3.33648400	-1.12941200
C	-1.71274600	4.71983000	-1.24192800
C	-2.02858500	5.49375700	-0.12446200
C	-2.23247000	4.87042500	1.10546500
C	-2.16586000	3.48007600	1.21405300
C	-3.37889100	0.36864300	-0.72098700
C	-3.76318600	0.96573700	-1.93362200
C	-4.85900000	0.49364000	-2.65407900
C	-5.59730900	-0.59514200	-2.18445100
C	-5.22818000	-1.19638300	-0.98140500
C	-4.12985800	-0.72524400	-0.26290100
C	0.32771800	-2.30130800	1.41787400
C	-0.85749300	-2.95868200	0.75262300
C	-1.40852000	-4.13976600	1.26014800
C	-2.44974100	-4.79109100	0.59685800
C	-2.95966600	-4.30062500	-0.60918500
C	-2.39229200	-3.13028300	-1.12558800
C	-1.37096500	-2.47540900	-0.45191300
C	1.65341700	-2.53829400	0.71523200
C	2.87992700	-2.39477100	1.38390300
C	4.08312000	-2.74616000	0.78033100
C	4.12583100	-3.25614000	-0.51931700
C	2.91123300	-3.37459300	-1.19824700
C	1.70431400	-3.02820800	-0.59793500
C	-4.08180500	-4.99614900	-1.33757800
C	5.42419500	-3.68089400	-1.15397700
C	0.34931700	-2.39218100	2.91595600
H	3.52534100	2.20077600	1.43054700
H	4.02003100	2.42122900	3.82348800
H	2.67587800	1.09871600	5.47620000
H	0.93705700	-0.42870500	4.66672000
H	-1.11550500	-1.32070700	4.58898800
H	-3.22099200	-0.47449100	5.46736100
H	-4.72130500	0.89476500	3.99203500
H	-4.00613800	1.37013800	1.68821500
H	2.66473200	2.29833400	-2.46204200
H	2.66733800	4.70987600	-2.98057000
H	1.80697500	6.34859300	-1.31751300
H	0.89745000	5.52968800	0.84995000
H	0.86379600	3.12495300	1.35011600
H	2.20150500	-0.35320100	-2.70059800
H	4.23407200	-0.89425600	-4.03498300
H	6.48360300	-0.71560800	-2.98563400
H	6.67542900	-0.02993200	-0.60476200
H	4.66252100	0.51332400	0.69662600
H	-1.30088400	2.74636700	-1.98094200
H	-1.50088300	5.19573300	-2.19410500
H	-2.08672600	6.57493500	-0.20632300
H	-2.44918000	5.46556700	1.98803300

H	-2.32651200	3.01606000	2.17935300
H	-3.21174400	1.81634400	-2.31400000
H	-5.14437300	0.98663100	-3.57973800
H	-6.45308800	-0.96165800	-2.74344300
H	-5.79204500	-2.04185400	-0.59781800
H	-3.85112800	-1.22352800	0.65682200
H	-1.01761600	-4.56774600	2.17738200
H	-2.86703700	-5.70007200	1.02253500
H	-2.77314800	-2.70635500	-2.04988000
H	-0.97644600	-1.54489900	-0.85510700
H	2.91575200	-1.99793800	2.39007500
H	5.00899400	-2.61618600	1.33409300
H	2.90397300	-3.74909600	-2.21839600
H	0.79013600	-3.16392300	-1.15873300
H	-4.56289900	-5.74974500	-0.70808400
H	-3.71883600	-5.50163400	-2.24066000
H	-4.84530300	-4.27832800	-1.65594900
H	6.26694500	-3.11671200	-0.74626300
H	5.40642200	-3.51846000	-2.23479800
H	5.62105000	-4.74646700	-0.98036500
H	1.29580600	-2.57643300	3.40833600
H	-0.49149000	-2.88742700	3.38377500
N	-0.63654200	-0.10532600	-2.74919900
C	-1.22793700	-0.31513700	-3.73622600
C	-2.03814600	-0.57640600	-4.91504900
H	-1.69772100	-1.48018700	-5.43146000
H	-3.08821600	-0.70439900	-4.62818200
H	-1.97023800	0.26257600	-5.61516900

Complex 22

Energy: -4237.599582 Hartree

Ni	0.06242700	-0.40876900	-0.77751300
P	2.17066600	-0.65679800	-0.09840700
P	-2.00203900	-0.74364000	-0.00603700
C	2.59541500	-2.19141200	-0.96484300
C	3.87614000	-2.64385900	-1.27346800
C	4.03410300	-3.78047400	-2.06559500
C	2.89563900	-4.43253200	-2.55326500
C	1.61594400	-3.99733300	-2.22355000
C	1.42838100	-2.86432200	-1.39549500
C	0.14771500	-2.26247400	-1.01678900
C	-0.94435800	-3.14977400	-0.60520200
C	-0.90414200	-4.56501200	-0.56124800
C	-2.04165600	-5.30964300	-0.26472100
C	-3.25771800	-4.68389300	0.02265000
C	-3.30510000	-3.28907400	0.06767100
C	-2.16281800	-2.54582800	-0.20325100
C	3.62860600	0.37239700	-0.51956800
C	4.60566300	0.81376900	0.37699400
C	5.72102900	1.51750200	-0.08289300
C	5.87351000	1.78761500	-1.44097300
C	4.91118400	1.33517200	-2.34616600
C	3.80078800	0.63447300	-1.88889400
C	2.23446900	-1.04208300	1.69377700
C	1.90325900	-2.34428400	2.10264300
C	1.79491400	-2.65620500	3.45423300
C	2.02041100	-1.67826300	4.42586800
C	2.34661600	-0.38239700	4.03032500
C	2.44236100	-0.06111000	2.67503500
C	-3.48842700	-0.15525800	-0.89376100

C	-3.55552300	-0.52796800	-2.24753600
C	-4.63021900	-0.13128100	-3.03643100
C	-5.65981700	0.63366200	-2.48188600
C	-5.59908500	1.00309900	-1.14028100
C	-4.51449900	0.61994600	-0.34973800
C	-2.28005200	-0.50820600	1.79185300
C	-1.17487400	-0.15158200	2.57515600
C	-1.29636500	-0.00930400	3.95653000
C	-2.53059900	-0.21297700	4.57222300
C	-3.63727300	-0.57744800	3.80257900
C	-3.51241000	-0.73325500	2.42348300
C	0.05811600	0.94888600	-2.22995700
H	4.74272700	-2.09629800	-0.91645900
H	5.02597900	-4.13907900	-2.32081200
H	3.00849900	-5.29291300	-3.20736500
H	-0.79372500	0.75942400	-2.87524500
H	0.75598100	-4.50172000	-2.64781500
H	0.02491200	-5.08416300	-0.75746000
H	-1.97765700	-6.39431600	-0.25368700
H	-4.14509400	-5.27130900	0.23573000
H	-4.23177600	-2.78434400	0.32262900
H	4.51999400	0.59090100	1.43237900
H	6.47642500	1.84307100	0.62607100
H	5.02715700	1.52733700	-3.40836600
H	3.07028500	0.26078100	-2.59874000
H	1.71560400	-3.10872100	1.35725300
H	1.53269900	-3.66728600	3.74984100
H	2.52297500	0.38845900	4.77512000
H	2.66502700	0.95614700	2.38503000
H	-2.76191900	-1.13689700	-2.67165400
H	-4.66952800	-0.42381600	-4.08134900
H	-6.39365900	1.59988500	-0.70294500
H	-4.46132800	0.94763900	0.67959800
H	-0.21384300	-0.00149400	2.09801800
H	-0.42268400	0.25411200	4.54256500
H	-4.59859700	-0.74871600	4.27773500
H	-4.37693900	-1.03104500	1.84220600
H	1.02095700	0.93129000	-2.72842400
H	6.74164800	2.33517800	-1.79487900
H	-6.50253800	0.93918500	-3.09456900
H	1.93732700	-1.92499500	5.47968000
H	-2.63103700	-0.09528900	5.64698700
C	-0.11418800	1.68137800	-1.03769900
C	-1.43321200	2.35360800	-0.77122100
C	-1.88298600	2.66983000	0.51868500
C	-2.21498300	2.80766600	-1.84634100
C	-3.04821900	3.40506100	0.72177900
H	-1.32945500	2.33171500	1.38295000
C	-3.38130600	3.53618000	-1.64168500
H	-1.88808900	2.60730200	-2.86011800
C	-3.82000700	3.85599400	-0.35239600
H	-3.36479700	3.62542600	1.73819100
H	-3.96027700	3.86487500	-2.50087700
C	1.04386800	2.45573300	-0.47696400
C	1.28808200	2.56978700	0.89768500
C	1.89248200	3.16382700	-1.33909400
C	2.34140800	3.33939200	1.38399600
H	0.66669700	2.02531300	1.59723500
C	2.95314500	3.92250300	-0.85309800
H	1.71190600	3.11807000	-2.40771500
C	3.20394100	4.02042100	0.51840100

H	2.50514700	3.40263300	2.45741300
H	3.60296000	4.44074100	-1.55298400
C	-5.09416700	4.63206600	-0.13868400
H	-5.96719700	4.05455900	-0.46605400
H	-5.23727600	4.88371300	0.91569800
H	-5.09665700	5.56608600	-0.71112700
C	4.38691100	4.79456700	1.03795500
H	4.54216500	5.71888200	0.47287700
H	5.30469700	4.20072000	0.94694800
H	4.26435800	5.05736200	2.09254300

Complex 23

Energy: -3731.60789 Hartree

Ni	0.03870600	0.50769500	-0.78466800
P	2.04952000	0.18688500	-0.07896600
P	-2.03177900	0.19108400	-0.18912800
C	2.54165700	-1.25585000	-1.04015200
C	3.84106700	-1.66479900	-1.31961700
C	4.05166100	-2.75249900	-2.16843100
C	2.94218200	-3.38989300	-2.73406200
C	1.64065300	-2.98927400	-2.44253600
C	1.39211700	-1.90349700	-1.56820000
C	0.08991600	-1.31328300	-1.24287600
C	-1.07261500	-2.17550200	-1.01037500
C	-1.11389800	-3.58770100	-1.11497800
C	-2.29659800	-4.29486700	-0.91996100
C	-3.48506200	-3.64575600	-0.57347400
C	-3.46176500	-2.26234500	-0.38557800
C	-2.28440600	-1.55342800	-0.59687800
C	3.39484700	1.39930100	-0.29424100
C	4.41985900	1.58041900	0.64293400
C	5.45377800	2.48094300	0.38506800
C	5.47452400	3.20113400	-0.80926100
C	4.45871800	3.01953300	-1.75015000
C	3.42290900	2.12495100	-1.49419100
C	2.07082300	-0.25966100	1.68808500
C	2.15886500	-1.59812500	2.09134600
C	2.06045200	-1.92800800	3.44302700
C	1.88688100	-0.92776800	4.40104800
C	1.79677600	0.40804200	4.00242300
C	1.87689700	0.74082600	2.65303700
C	-3.31514200	1.17170700	-1.03158000
C	-4.11993400	0.61971700	-2.03556600
C	-5.03003000	1.42247500	-2.72423900
C	-5.14269200	2.77849300	-2.42001600
C	-4.33270400	3.33796800	-1.42901900
C	-3.41805400	2.54262100	-0.74444900
C	-2.41830500	0.25953100	1.59924000
C	-1.50678500	-0.34466900	2.47929000
C	-1.76060700	-0.36252100	3.84696500
C	-2.91834500	0.23214000	4.35541000
C	-3.82610300	0.83397900	3.48533100
C	-3.58324600	0.84429800	2.11069400
H	4.68089800	-1.12436000	-0.89205900
H	5.05805600	-3.08154000	-2.40488000
H	3.09447000	-4.21173300	-3.42859600
H	0.81505400	-3.47621900	-2.94633700
H	-0.20764400	-4.13898400	-1.32668600
H	-2.28566100	-5.37708900	-1.01854700
H	-4.39909600	-4.20869400	-0.41584300

H	-4.35240700	-1.73988500	-0.04741800
H	4.40759500	1.02170600	1.57226900
H	6.24333900	2.61778300	1.11770900
H	4.47088300	3.57864000	-2.68046000
H	2.63013700	1.98705100	-2.22331300
H	2.29805400	-2.37347500	1.34577900
H	2.12703900	-2.96787700	3.74782700
H	1.64854600	1.18886700	4.74172700
H	1.78397600	1.77747800	2.34385900
H	-4.03017200	-0.43287700	-2.27835300
H	-5.65023100	0.98542800	-3.50086400
H	-4.40716500	4.39560700	-1.19598000
H	-2.77854600	2.98542400	0.01277100
H	-0.60371600	-0.80281600	2.09055200
H	-1.04528800	-0.83241400	4.51332300
H	-4.73045400	1.29383200	3.87274100
H	-4.30004200	1.30507600	1.44056300
H	6.27851800	3.90354800	-1.00649300
H	-5.85195500	3.40034300	-2.95744300
H	1.81849100	-1.18724900	5.45305700
H	-3.11021300	0.22585300	5.42409300
O	-0.03191200	3.39809600	-1.15353900
C	-0.00446600	2.24783300	-1.02282700

TS1

Energy: -4237.577702 Hartree

Ni	0.13186200	-0.20018400	-0.77705900
P	1.52885700	-1.02739500	0.64751600
P	-2.08529000	-0.39928200	-0.45359900
C	2.00639500	-2.58568900	-0.20206400
C	3.09456400	-3.38233700	0.17647700
C	3.52691000	-4.41341600	-0.65223600
C	2.89865800	-4.61516000	-1.88410400
C	1.81423900	-3.82373700	-2.25851400
C	1.32258500	-2.82435400	-1.40451500
C	0.11944300	-1.97964900	-1.73774200
C	-1.21009400	-2.69522300	-1.70446500
C	-1.36990200	-4.01342600	-2.16488700
C	-2.61303000	-4.64141600	-2.15363100
C	-3.74236700	-3.97237200	-1.67883500
C	-3.60458800	-2.67500700	-1.19760700
C	-2.35356100	-2.04664000	-1.19486300
C	3.22796000	-0.35777500	0.94024500
C	3.74784500	0.02182100	2.18233900
C	5.05829500	0.49275900	2.28994300
C	5.87063500	0.57693600	1.16144200
C	5.36026000	0.19697200	-0.08170100
C	4.05160000	-0.25709300	-0.19190300
C	0.98906300	-1.53922700	2.32566100
C	0.67496400	-2.87399100	2.61760700
C	0.13723600	-3.22204900	3.85705500
C	-0.08689600	-2.24539000	4.82816400
C	0.21774500	-0.91194700	4.54697600
C	0.74154200	-0.56109200	3.30509200
C	-3.01744100	0.72399900	-1.56265200
C	-3.26260400	0.37061500	-2.89771000
C	-3.78462900	1.30204800	-3.79522700
C	-4.07491600	2.60013800	-3.37413700
C	-3.84761500	2.95835000	-2.04404200
C	-3.32083200	2.03270900	-1.14689000

C	-3.14064400	-0.45756700	1.05750500
C	-2.58274100	-1.06175400	2.19328700
C	-3.29365900	-1.12157500	3.38982200
C	-4.57389300	-0.57065200	3.47207600
C	-5.14249800	0.02348300	2.34515200
C	-4.43399300	0.07488700	1.14419900
C	0.31667400	-0.87278200	-2.61694900
H	3.61915800	-3.16774600	1.10234900
H	4.36920200	-5.03286700	-0.35951500
H	3.26252100	-5.38385000	-2.55966300
H	-0.50376900	-0.54334800	-3.25592700
H	1.35801000	-3.96342000	-3.23293000
H	-0.51334100	-4.56386200	-2.52977900
H	-2.69766300	-5.66166400	-2.51687400
H	-4.71073300	-4.46313900	-1.66736400
H	-4.46316400	-2.14849900	-0.79232700
H	3.14566200	-0.06857300	3.07786800
H	5.44572300	0.77860700	3.26363400
H	5.98079900	0.26434500	-0.96976800
H	3.66204400	-0.54643700	-1.16156300
H	0.84365900	-3.63939000	1.86820400
H	-0.10463000	-4.26019200	4.06425500
H	0.03553400	-0.14219800	5.29085100
H	0.94515100	0.48245600	3.08984900
H	-3.04040600	-0.63582500	-3.23438600
H	-3.96228700	1.01194800	-4.82652700
H	-4.07034100	3.96474500	-1.70207500
H	-3.14388900	2.32921700	-0.12039500
H	-1.58515200	-1.48000600	2.13812700
H	-2.83831600	-1.59112300	4.25537200
H	-6.14210900	0.44555900	2.39765300
H	-4.88790600	0.53854400	0.27597700
N	-0.61000400	2.11603500	-3.28633000
H	1.30921700	-0.72541700	-3.03992900
H	6.89344300	0.93160700	1.24798800
H	-4.47638000	3.32524900	-4.07539900
H	-0.50078000	-2.51926400	5.79382000
H	-5.12533400	-0.60650300	4.40703000
C	0.39550800	1.72506500	-0.77708700
N	-0.26482400	2.17683200	-2.22154400
C	1.81083600	2.21325600	-0.92971000
C	2.54630200	2.04017300	-2.11090900
C	2.45342400	2.83106200	0.15403100
C	3.85262000	2.50562700	-2.21597800
H	2.08724000	1.54885700	-2.96146400
C	3.77226600	3.25986600	0.05404700
H	1.91459000	2.97338900	1.08313000
C	4.49339100	3.12254200	-1.13593900
H	4.38936100	2.37596900	-3.15221200
H	4.24849300	3.71474800	0.91809300
C	-0.41243400	2.54233300	0.20521200
C	-0.73855600	3.88491600	-0.03115000
C	-0.85891800	1.96393500	1.39714200
C	-1.53990900	4.59574300	0.85773800
H	-0.38370900	4.36813900	-0.93661500
C	-1.64710700	2.67895000	2.29525300
H	-0.62178800	0.93098500	1.59737200
C	-2.01612300	4.00310400	2.03532400
H	-1.80622100	5.62512700	0.63252700
H	-2.00753800	2.18032800	3.19083200
C	5.92355500	3.58640900	-1.22948000

H	6.58009000	2.93956600	-0.63550000
H	6.28383900	3.57039600	-2.26172700
H	6.04201600	4.60508000	-0.84543000
C	-2.87964300	4.77193100	3.00323300
H	-3.60796400	4.11654300	3.48971800
H	-2.27461200	5.23118300	3.79468400
H	-3.42511600	5.57613400	2.50151400

TS2

Energy: -4237.577702 Hartree

Ni	-0.05013300	0.15137300	0.11838400
P	-1.83480000	-0.96902000	-0.21612900
P	1.94403000	-0.72022600	-0.07656200
C	-2.46009100	-1.05299200	1.51947400
C	-3.77224800	-1.31206900	1.92226500
C	-4.13359200	-1.17950700	3.26236500
C	-3.18484000	-0.76902000	4.19914300
C	-1.87189300	-0.51509300	3.80145500
C	-1.48572700	-0.67610300	2.46431600
C	-0.10353800	-0.35110600	1.94770600
C	1.05140600	-1.16175300	2.47519800
C	1.08514400	-1.77381800	3.73687700
C	2.24113400	-2.40464200	4.19739200
C	3.39697700	-2.42537800	3.41692000
C	3.36674400	-1.86541100	2.14045000
C	2.19211200	-1.28923000	1.64984700
C	-1.64275000	-2.72708100	-0.71057300
C	-0.87865600	-3.55952800	0.12157700
C	-0.59799700	-4.86847900	-0.25552300
C	-1.07490400	-5.36694800	-1.47003800
C	-1.82838000	-4.54480300	-2.30505500
C	-2.10587000	-3.22863900	-1.93242500
C	-3.24533500	-0.36959800	-1.22344900
C	-4.45973500	-1.05969100	-1.37001800
C	-5.50309600	-0.50155400	-2.10590700
C	-5.34360900	0.74880500	-2.70942200
C	-4.13260100	1.42914800	-2.58979900
C	-3.08695400	0.86865200	-1.85666200
C	2.04106400	-2.22807700	-1.12232100
C	1.27630400	-2.23306200	-2.29678500
C	1.31611700	-3.32257100	-3.16429200
C	2.11052800	-4.42728900	-2.86033600
C	2.86546700	-4.43683900	-1.68690300
C	2.83538900	-3.34238500	-0.82481700
C	3.53046400	0.12746600	-0.44004800
C	4.06892400	1.00530800	0.51214700
C	5.21729200	1.73796800	0.22915500
C	5.84319900	1.61019600	-1.01226100
C	5.30761400	0.74845200	-1.96951000
C	4.15891300	0.00984600	-1.68597100
C	0.17678600	1.16521300	1.83643100
C	-0.09892600	2.03848100	0.24563300
C	-1.37320600	2.78555700	0.23886000
C	-1.54136500	3.87897600	-0.63669500
C	-2.78899500	4.46475300	-0.83697700
C	-3.92390200	4.02457500	-0.14798600
C	-3.74994200	2.98540900	0.77552200
C	-2.51586300	2.38065500	0.96183400
C	-5.28679900	4.61093100	-0.41079400
C	1.10460400	2.87818100	-0.04044800

C	1.77391700	2.75052100	-1.26661400
C	2.87914300	3.53623300	-1.57249300
C	3.38459100	4.46023900	-0.65043700
C	2.72082700	4.59388200	0.57326400
C	1.58834400	3.83428900	0.86412300
C	4.62539800	5.25688300	-0.96321200
H	-4.52349500	-1.58120700	1.18978700
H	-5.15701500	-1.37128100	3.56982800
H	-3.46981000	-0.62902900	5.23753500
H	-1.15333200	-0.15077200	4.52831800
H	0.20874000	-1.75409200	4.37134200
H	2.24144900	-2.86510500	5.18102400
H	4.30486900	-2.88901700	3.78996300
H	4.25545000	-1.88101100	1.51742100
H	-0.48823900	-3.17270400	1.05756900
H	0.00368400	-5.49732400	0.39313900
H	-0.84959700	-6.38684100	-1.76607500
H	-2.19559400	-4.92244800	-3.25494800
H	-2.68067900	-2.59261400	-2.59598700
H	-4.58251200	-2.04374300	-0.92933800
H	-6.43889100	-1.04237800	-2.21179800
H	-6.16018600	1.18422800	-3.27788700
H	-3.99827700	2.40121200	-3.05262000
H	-2.14804400	1.39785000	-1.74777600
H	0.63169000	-1.38621000	-2.51105800
H	0.70887700	-3.31713500	-4.06349300
H	2.13203900	-5.28275700	-3.52866400
H	3.47803100	-5.29917400	-1.44063600
H	3.41727400	-3.36643000	0.08827400
H	3.58430500	1.11895900	1.47574900
H	5.61792000	2.41762300	0.97470400
H	6.74099800	2.18005000	-1.23204000
H	5.78879200	0.64347500	-2.93755600
H	3.75235100	-0.66437100	-2.43279600
H	-0.51620000	1.73197300	2.45556300
H	1.20243900	1.41458300	2.09695000
H	-0.68701600	4.24309700	-1.19617700
H	-2.88092300	5.28552500	-1.54479100
H	-4.60412500	2.62508700	1.34369000
H	-2.44825900	1.56102600	1.66465600
H	-5.21716700	5.57446300	-0.92402900
H	-5.84766600	4.76169100	0.51763400
H	-5.88530000	3.94208700	-1.04283400
H	1.41497100	2.00769700	-1.97108700
H	3.38204800	3.40312500	-2.52624400
H	3.08844700	5.31077300	1.30356100
H	1.07073900	3.98187400	1.80786800
H	4.55964000	5.73277100	-1.94748900
H	5.50966000	4.60848600	-0.97854500
H	4.79767200	6.04053200	-0.22021000

TS3

Energy: -4237.604216 Hartree

Ni	0.32863300	0.06188000	-0.67190300
P	1.46502700	-1.16178100	0.61674400
P	-1.99626000	-0.13027900	-0.65187100
C	2.08848700	-2.55503200	-0.40410000
C	3.22596100	-3.31228700	-0.10563700
C	3.68050600	-4.27314900	-1.00701700
C	3.01182700	-4.46156200	-2.21810100

C	1.87688500	-3.70712400	-2.51738800
C	1.39311300	-2.75946100	-1.60694600
C	0.15030700	-1.96209500	-1.88902100
C	-1.15717000	-2.62188600	-1.57286600
C	-1.30194600	-4.00938000	-1.73615900
C	-2.50803000	-4.65074200	-1.46106500
C	-3.60736500	-3.91787500	-1.01700800
C	-3.47918500	-2.54263900	-0.83473700
C	-2.26642300	-1.89095800	-1.08739600
C	3.02562900	-0.60724300	1.41673800
C	3.39298300	-0.89866700	2.73736000
C	4.62683300	-0.47867700	3.23674600
C	5.51245000	0.22625900	2.42182400
C	5.16337200	0.50132800	1.09893400
C	3.93144900	0.08675200	0.60028200
C	0.50510200	-1.93373200	1.97354300
C	-0.09575200	-3.18836400	1.79661500
C	-0.94893000	-3.70592500	2.77010800
C	-1.20530700	-2.98579200	3.93880400
C	-0.61680100	-1.73265600	4.12000100
C	0.22066500	-1.20362500	3.13979400
C	-2.74910200	0.79017500	-2.04974500
C	-3.45345500	0.16741500	-3.08766900
C	-3.96033100	0.91975100	-4.14893200
C	-3.78033900	2.30215600	-4.17867900
C	-3.08250400	2.93129100	-3.14512900
C	-2.56050700	2.18170700	-2.09353800
C	-3.19198200	0.13962000	0.71236000
C	-3.01263000	-0.61731100	1.88092900
C	-3.79275000	-0.37675600	3.00730900
C	-4.76388100	0.62856100	2.98824100
C	-4.95278900	1.37948600	1.82819000
C	-4.17457900	1.13647300	0.69538800
C	0.24549900	-0.88346400	-2.76388000
H	3.76451900	-3.12950200	0.81838000
H	4.56464700	-4.85923400	-0.77556900
H	3.37884500	-5.19051500	-2.93437700
H	-0.63735500	-0.46543200	-3.23381800
H	1.36433500	-3.84294000	-3.46491400
H	-0.45707900	-4.59566200	-2.07692600
H	-2.58440800	-5.72594400	-1.59367800
H	-4.54884400	-4.41206100	-0.79813300
H	-4.31610300	-1.96749500	-0.45288600
H	2.72314600	-1.46031700	3.37737700
H	4.89600400	-0.70883800	4.26347900
H	5.84164600	1.04910200	0.45287300
H	3.66911300	0.30436900	-0.42744700
H	0.09307600	-3.75387400	0.89151000
H	-1.41374700	-4.67453600	2.61371800
H	-0.82064800	-1.15877100	5.01880000
H	0.65589700	-0.21963800	3.27970400
H	-3.60238500	-0.90673700	-3.06839900
H	-4.49966500	0.42327000	-4.95037700
H	-2.93409900	4.00693900	-3.16190200
H	-2.00352500	2.67418500	-1.30491000
H	-2.25817400	-1.39385700	1.90429000
H	-3.63519200	-0.97305500	3.90073200
H	-5.70999200	2.15787500	1.80070500
H	-4.32535600	1.73370000	-0.19619200
H	1.20191200	-0.64737600	-3.21587700
H	6.46999400	0.55538600	2.81430500

H	-4.17739300	2.88615500	-5.00358100
H	-1.86477300	-3.39430600	4.69823400
H	-5.36862400	0.82177500	3.86922700
C	0.77227500	1.79982100	-0.41475000
C	-0.05983600	2.63591100	0.45506700
C	-0.29441400	3.99752900	0.16322500
C	-0.75896500	2.08927100	1.55275700
C	-1.23428400	4.73553000	0.87889700
H	0.23163600	4.45497800	-0.66867800
C	-1.66728400	2.83997900	2.28328500
H	-0.59166100	1.05261500	1.81270300
C	-1.94373800	4.17196200	1.94673600
H	-1.42376600	5.76973400	0.60143500
H	-2.20221800	2.37046500	3.10411000
C	2.06094200	2.38737700	-0.79952000
C	2.79595500	3.22735400	0.06109000
C	2.65632400	2.04998000	-2.03256500
C	4.07076400	3.67064000	-0.27733000
H	2.36932100	3.49671900	1.02104700
C	3.91853300	2.51254200	-2.37845400
H	2.09667800	1.40967700	-2.70486000
C	4.65516700	3.32496500	-1.50122500
H	4.62769300	4.28864600	0.42236200
H	4.34992200	2.23717700	-3.33769800
C	-2.97660800	4.95641200	2.71440900
H	-3.92281600	4.40617200	2.76848100
H	-2.65543100	5.14326900	3.74633900
H	-3.17343800	5.92542600	2.24750800
C	6.02379300	3.82940700	-1.88214800
H	5.95344000	4.71647700	-2.52401500
H	6.60613500	4.11044700	-1.00017600
H	6.58791800	3.07430600	-2.43848200

TS4

Energy: -4350.975764 Hartree

Ni	-0.06891200	-0.30673300	-0.53017500
P	1.75048400	0.73702200	-0.19189900
P	-1.69567100	1.00794400	-0.15092300
C	2.23581800	0.39249600	1.53787800
C	3.49807000	0.76227100	2.02945900
C	3.85274500	0.54924000	3.35413600
C	2.92670800	-0.04478500	4.21248200
C	1.68948400	-0.44300900	3.72323000
C	1.31249300	-0.25013800	2.37948700
C	-0.06745000	-0.79458800	1.97175100
C	-1.19318100	0.14434900	2.43832100
C	-1.50357800	0.07972900	3.80977900
C	-2.36738000	0.98265100	4.41810400
C	-2.96887000	1.98682500	3.66007500
C	-2.72856800	2.03033500	2.29229600
C	-1.87259700	1.10850400	1.66572600
C	1.79647600	2.57399200	-0.26297500
C	2.01415900	3.40365800	0.84174000
C	2.11635100	4.78737900	0.67730500
C	1.99662100	5.35486500	-0.58889800
C	1.74806700	4.53534400	-1.69312400
C	1.64518500	3.15902900	-1.53073400
C	3.28090800	0.38959400	-1.16482900
C	4.37896400	1.26356700	-1.21711800
C	5.51680400	0.92659700	-1.94749700

C	5.57015600	-0.28205700	-2.64611500
C	4.47540100	-1.14483900	-2.61678800
C	3.33856600	-0.80703700	-1.88282100
C	-1.66914500	2.75735300	-0.71234300
C	-1.25780600	3.80955800	0.11509900
C	-1.24922800	5.12001000	-0.35792200
C	-1.62634000	5.39858000	-1.67064300
C	-2.00531700	4.35282000	-2.51283200
C	-2.03026400	3.04394600	-2.03730200
C	-3.38507400	0.52202200	-0.74930600
C	-4.57419100	0.72929400	-0.03941600
C	-5.80021400	0.32652600	-0.57039900
C	-5.85917000	-0.28114100	-1.82485400
C	-4.68088600	-0.48893100	-2.54180500
C	-3.45568800	-0.09826500	-2.00471700
C	-0.23976300	-2.20832900	2.46246500
C	-0.13520100	-2.31234100	0.97030000
C	1.17380600	-2.84888800	0.43497700
C	1.28375900	-3.39041400	-0.86081700
C	2.45948500	-3.97113100	-1.31736800
C	3.61778400	-3.99492200	-0.53063100
C	3.52557200	-3.44634000	0.74777100
C	2.33175900	-2.90929500	1.22715400
C	4.91968300	-4.53458600	-1.06141300
C	-1.43358300	-2.83589900	0.39129500
C	-2.65404300	-2.18332000	0.61638700
C	-3.86071900	-2.71206000	0.18095500
C	-3.91766800	-3.93815800	-0.48871500
C	-2.71885800	-4.63593600	-0.63956100
C	-1.50579700	-4.11055700	-0.19275600
C	-5.22412900	-4.46619600	-1.02269000
H	4.21195200	1.21554800	1.35213600
H	4.83560700	0.83859300	3.71289400
H	3.17313900	-0.21696500	5.25582600
H	1.00749100	-0.94014000	4.40137600
H	-1.05041300	-0.69205600	4.41960800
H	-2.56432100	0.90393400	5.48291200
H	-3.63409300	2.70877100	4.12329100
H	-3.21061200	2.79081400	1.68813100
H	2.12119200	2.97311100	1.83056100
H	2.29641800	5.41846400	1.54294900
H	2.08054800	6.42986400	-0.71625100
H	1.62569300	4.97216000	-2.67902400
H	1.45540200	2.52492500	-2.39154400
H	4.33478400	2.21987100	-0.70643100
H	6.35877800	1.61195000	-1.98007900
H	6.45482500	-0.53966000	-3.22101900
H	4.49633700	-2.07801300	-3.17132400
H	2.48341900	-1.46717400	-1.87307800
H	-0.93701200	3.60814800	1.12925700
H	-0.92856900	5.92161300	0.29919500
H	-1.61632400	6.42049300	-2.03738000
H	-2.29344600	4.55617900	-3.53998700
H	-2.34960300	2.24294500	-2.69513000
H	-4.55186600	1.18143700	0.94398400
H	-6.71018300	0.48663100	0.00076600
H	-6.81375000	-0.59561800	-2.23583500
H	-4.70989300	-0.97121600	-3.51414200
H	-2.54434500	-0.30537900	-2.55186100
H	0.55698600	-2.65221100	3.05045200
H	-1.22196300	-2.45955200	2.85019300

H	0.44272400	-3.34572200	-1.53586000
H	2.48480900	-4.38281100	-2.32302400
H	4.39903800	-3.44059700	1.39432300
H	2.33638700	-2.51734200	2.23326900
H	4.75560500	-5.27061700	-1.85387500
H	5.51058300	-5.00843600	-0.27177600
H	5.52778600	-3.72478100	-1.48313200
H	-2.67319100	-1.24006000	1.13765100
H	-4.76973700	-2.14184700	0.34286300
H	-2.72400700	-5.61624900	-1.10938600
H	-0.61660500	-4.71679000	-0.29364100
H	-5.99961400	-4.47613400	-0.24878200
H	-5.11852500	-5.48373600	-1.40880600
H	-5.59425900	-3.83357300	-1.83820200
C	-0.21107400	-1.04541500	-2.08944500
O	-0.28301300	-1.29581000	-3.22736000

TS5
Energy: -4370.381113 Hartree

Ni	-0.13487600	0.01205700	0.02132100
P	-2.14105500	-0.76201800	-0.01310200
P	1.79105300	-1.07173400	-0.11139000
C	-2.68283100	-0.49988500	1.68326300
C	-3.97469100	-0.29512800	2.15028500
C	-4.17726200	-0.06864400	3.51482600
C	-3.07462100	-0.06029900	4.37386400
C	-1.77865500	-0.27066300	3.89943600
C	-1.54145600	-0.49455700	2.52502400
C	-0.23105000	-0.59314500	1.86801400
C	0.76444400	-1.52711100	2.34523000
C	0.71462300	-2.22823600	3.58282700
C	1.75652900	-3.04091900	3.99890800
C	2.90839800	-3.21847400	3.21438900
C	2.96693300	-2.59429500	1.97383900
C	1.90976700	-1.78970700	1.53353500
C	-2.11229500	-2.56538100	-0.29121900
C	-2.09630300	-3.44840900	0.79516500
C	-1.95941700	-4.81919700	0.57874100
C	-1.84482200	-5.31807400	-0.71821300
C	-1.85120400	-4.44029500	-1.80347600
C	-1.96878900	-3.06984700	-1.59169200
C	-3.44932900	-0.10232600	-1.10221900
C	-4.41603300	-0.90280500	-1.72617300
C	-5.39625300	-0.32185700	-2.53198000
C	-5.42557300	1.06093900	-2.71749200
C	-4.47446200	1.86590100	-2.08633800
C	-3.48995700	1.28876500	-1.28700300
C	1.55353500	-2.44969400	-1.30436500
C	1.21770500	-2.13463600	-2.63104400
C	1.07659000	-3.13985100	-3.58539300
C	1.24106900	-4.47680400	-3.21916000
C	1.53422300	-4.79899100	-1.89442500
C	1.69321500	-3.79336700	-0.94105800
C	3.48465000	-0.48140500	-0.50056800
C	4.21951200	0.13796900	0.52047400
C	5.51047600	0.59846700	0.28216400
C	6.08710800	0.45507600	-0.98060900
C	5.35572400	-0.14072100	-2.00722800
C	4.06437900	-0.61021300	-1.76925400
C	0.55248100	1.39401000	2.19845800

C	0.57348500	2.27523200	1.09301000
C	-0.61865900	3.03933500	0.72677200
C	-0.62279100	4.00825500	-0.30472600
C	-1.77029800	4.73056200	-0.63181400
C	-2.97887300	4.55031400	0.04967900
C	-2.98187500	3.60384900	1.08666200
C	-1.85334400	2.86489200	1.40728600
C	-4.21942400	5.33279700	-0.29911700
C	1.91023500	2.69124700	0.57721000
C	2.28511400	2.70544900	-0.77859000
C	3.53085800	3.16710400	-1.18920300
C	4.47372300	3.63728400	-0.26875400
C	4.11819400	3.61957200	1.08311600
C	2.86925500	3.16100200	1.49368300
C	5.82764700	4.11567900	-0.72454400
H	-4.81004700	-0.29825700	1.45613700
H	-5.17638300	0.10444200	3.90113200
H	-3.22288800	0.13419900	5.43260800
H	-0.94432900	-0.20961200	4.58902900
H	-0.15989000	-2.13398300	4.21401000
H	1.67624300	-3.55109300	4.95548200
H	3.72484900	-3.84359700	3.56035100
H	3.83186200	-2.73260000	1.33096200
H	-2.18581300	-3.06226700	1.80380300
H	-1.94335400	-5.49650000	1.42707100
H	-1.74008700	-6.38593000	-0.88414800
H	-1.73950000	-4.82005500	-2.81316000
H	-1.94598400	-2.38987300	-2.43692800
H	-4.40178200	-1.97750500	-1.58424400
H	-6.14038300	-0.95137400	-3.01061400
H	-6.19026000	1.50925100	-3.34481400
H	-4.49479500	2.94421800	-2.20820900
H	-2.76527400	1.91930900	-0.78925500
H	1.06915500	-1.09655100	-2.91229600
H	0.83165600	-2.88109200	-4.61151400
H	1.12709800	-5.26286700	-3.95961200
H	1.64058900	-5.83838300	-1.59910700
H	1.92098500	-4.04945800	0.08715500
H	3.77874400	0.25424100	1.50311100
H	6.06344100	1.07727100	1.08347800
H	7.09895700	0.80483700	-1.16184200
H	5.79558100	-0.25764600	-2.99342400
H	3.51994600	-1.09712100	-2.56988900
H	-0.17632100	1.52944100	2.99172000
H	1.51427700	1.03025900	2.54462100
H	0.29678400	4.21648100	-0.83919600
H	-1.71550100	5.47385200	-1.42604300
H	-3.89974900	3.41885300	1.64004600
H	-1.93642600	2.12508100	2.19220400
H	-4.07401300	5.92528800	-1.20781700
H	-4.50163700	6.02719100	0.50209400
H	-5.08049800	4.67341600	-0.46297300
H	1.59900300	2.32624100	-1.52153000
H	3.78595600	3.14167500	-2.24594000
H	4.82532700	3.98245900	1.82542700
H	2.61058400	3.19030000	2.54780100
H	6.41038500	3.28937700	-1.14831100
H	6.40161400	4.53910200	0.10451300
H	5.74410800	4.88345800	-1.50181500
N	-0.27910800	0.85955500	-1.62377200
C	-0.63531100	1.51657000	-2.50855500

C	-1.12161700	2.39243000	-3.55911800
H	-0.35459100	2.55280600	-4.32149000
H	-2.01451700	1.95903600	-4.01725800
H	-1.38885700	3.34889800	-3.09971200

TS6
Energy: -4347.119694 Hartree

Ni	0.43477900	-0.18634700	-0.83170700
P	0.46509300	-2.26097500	-0.24463600
P	2.13482800	1.02798000	-0.37160500
N	-0.90754900	0.26118100	-1.78286100
N	-2.08653800	0.41256900	-1.63734100
C	0.77150600	-2.02162300	1.52897000
C	1.40013400	-2.97064600	2.34473200
C	1.68212600	-2.68364100	3.67678700
C	1.31226800	-1.44329100	4.19982600
C	0.67313200	-0.50406300	3.39393100
C	0.39454600	-0.76531200	2.04214600
C	-0.31208800	0.32039800	1.22536100
C	0.25783600	1.69456200	1.53004600
C	-0.36562900	2.56184400	2.43792000
C	0.18693400	3.79988900	2.75620400
C	1.39405000	4.19883600	2.18318100
C	2.03288400	3.35112300	1.28061600
C	1.47424000	2.11354900	0.94944400
C	1.61014600	-3.59566000	-0.73170700
C	1.18862700	-4.83167200	-1.23360000
C	2.13042300	-5.78804400	-1.61881200
C	3.49391000	-5.52223400	-1.49468100
C	3.91852400	-4.29072200	-0.98753100
C	2.98400200	-3.33011200	-0.61586600
C	-1.21354300	-2.99100300	-0.32039900
C	-1.91837000	-2.91910700	-1.53105500
C	-3.24229400	-3.34459600	-1.60296500
C	-3.88584500	-3.82670100	-0.46279200
C	-3.19188300	-3.90432200	0.74454900
C	-1.86029100	-3.49507200	0.81626500
C	3.66212300	0.27768100	0.29982400
C	4.65616400	-0.18009700	-0.57984200
C	5.73119500	-0.92827900	-0.10353600
C	5.81646500	-1.24936400	1.25327900
C	4.82999600	-0.79982500	2.13206300
C	3.76535700	-0.03241300	1.66239300
C	2.74273500	2.20113800	-1.64256400
C	3.95838300	2.89250800	-1.53587000
C	4.32390100	3.82715200	-2.50331600
C	3.47997200	4.08160700	-3.58648400
C	2.27529900	3.38833400	-3.70691900
C	1.91257500	2.44703200	-2.74402700
C	-1.72088800	0.21758800	1.23148500
C	-2.87136700	0.98870900	-0.67996500
C	-4.19888300	0.33875700	-0.53927200
C	-4.88482800	0.28452700	0.68575600
C	-6.08541500	-0.41043700	0.80531100
C	-6.65401700	-1.07861900	-0.28412500
C	-5.97766300	-1.01431400	-1.50712200
C	-4.77781000	-0.32299500	-1.63430100
C	-7.93170200	-1.86634000	-0.13781500
C	-2.63338800	2.42760500	-0.41846500
C	-1.46046500	3.05826400	-0.87881900

C	-1.15104100	4.35920800	-0.51230200
C	-1.99384700	5.10035700	0.32506700
C	-3.18437300	4.49646900	0.73973400
C	-3.50968400	3.19277600	0.36973600
C	-1.61103800	6.48759500	0.77009700
H	1.69400800	-3.92434700	1.91909400
H	2.18489100	-3.41733300	4.29925500
H	1.52082600	-1.20423100	5.23863200
H	0.39939100	0.45785900	3.81236700
H	-1.30221400	2.26733300	2.89714200
H	-0.33028500	4.45476300	3.45094600
H	1.82763000	5.16389800	2.42708000
H	2.95489200	3.66842000	0.80540200
H	0.12852500	-5.04513800	-1.32178700
H	1.79609900	-6.74424800	-2.01037800
H	4.22305300	-6.26997100	-1.79189700
H	4.97731500	-4.07290300	-0.88452500
H	3.31479400	-2.37440300	-0.22707500
H	-1.43584700	-2.49477300	-2.40596300
H	-3.78290600	-3.26854100	-2.54051200
H	-4.92886700	-4.12241200	-0.51257400
H	-3.68933200	-4.27631600	1.63525900
H	-1.32828200	-3.54385800	1.76026600
H	4.58066300	0.04209400	-1.63958000
H	6.49637200	-1.27045100	-0.79410700
H	6.64590400	-1.84526600	1.62146800
H	4.88334100	-1.04793400	3.18756400
H	3.00179600	0.30574600	2.35208300
H	4.62377800	2.68879500	-0.70303700
H	5.26735800	4.35715500	-2.41222600
H	3.76611400	4.81097800	-4.33821100
H	1.62173500	3.57200700	-4.55415400
H	0.98838800	1.88521000	-2.83997600
H	-2.18091300	-0.76100900	1.30507700
H	-2.31588000	1.04603300	1.58197600
H	-4.46719500	0.75994000	1.56485800
H	-6.58482700	-0.44167500	1.77037200
H	-6.39909100	-1.51268000	-2.37683600
H	-4.26937400	-0.28840700	-2.59115400
H	-8.58444000	-1.43433400	0.62655600
H	-7.72751100	-2.90311700	0.15915400
H	-8.48902100	-1.90257300	-1.07854400
H	-0.77528400	2.52252100	-1.51947300
H	-0.22290500	4.79783500	-0.86839500
H	-3.87655600	5.05643300	1.36373100
H	-4.45203900	2.77062700	0.69585500
H	-1.34037100	7.12325100	-0.08000400
H	-0.73960400	6.45337700	1.43534000
H	-2.42675400	6.97602600	1.31031100

TS7
Energy: -4347.07215 Hartree

Ni	-0.76551300	-0.50084100	-0.73204200
P	0.71249900	-2.01410900	-0.48726300
P	1.62093200	1.28583900	0.24548100
N	-1.89090200	-1.68626900	-1.60707400
N	-2.97308800	-1.46091600	-1.10861000
C	0.66395700	-2.21898900	1.32168800
C	1.32904400	-3.25680900	1.99020400
C	1.25833500	-3.35613400	3.37516700

C	0.49452700	-2.42683500	4.08711600
C	-0.19772200	-1.42151700	3.41664700
C	-0.14197100	-1.29249700	2.01725500
C	-0.90647400	-0.19191800	1.27519600
C	-0.57919600	1.10962200	1.97583100
C	-1.36995900	1.61409900	3.02794500
C	-0.97206000	2.69626000	3.80657600
C	0.26541500	3.30144200	3.58308300
C	1.05668000	2.83966900	2.53695300
C	0.62482800	1.79965700	1.69842500
C	2.42953100	-1.71296700	-1.03206900
C	3.55335300	-2.04516600	-0.26997200
C	4.83409100	-1.83063900	-0.77878600
C	5.00218800	-1.29276400	-2.05333200
C	3.88338800	-0.96548100	-2.82366500
C	2.60298700	-1.17005000	-2.31399100
C	0.36328500	-3.69602600	-1.10691900
C	1.17476200	-4.34188200	-2.04717200
C	0.83333200	-5.61078000	-2.51566800
C	-0.32149600	-6.24218300	-2.05489200
C	-1.13787100	-5.60086000	-1.12127900
C	-0.80110600	-4.33461600	-0.65249500
C	3.21574200	0.89224800	1.10447000
C	4.47185300	1.27260000	0.61666900
C	5.64067100	0.83381200	1.23921100
C	5.57847100	0.00088400	2.35574600
C	4.33096700	-0.37660200	2.85656100
C	3.16312800	0.06710100	2.24179000
C	2.07481900	2.84138200	-0.64462100
C	1.77713100	4.15275900	-0.25069100
C	2.24113900	5.24162900	-0.98895200
C	3.00053000	5.04367900	-2.14229800
C	3.26294500	3.74259100	-2.57383900
C	2.79074900	2.65405700	-1.84059100
C	-2.43348800	-0.51068000	1.19107900
C	-3.09027100	-0.12566500	-0.19750600
C	-4.57514700	0.10141300	-0.08100900
C	-5.37268100	-0.87411200	0.53612400
C	-6.74316400	-0.69100100	0.67882400
C	-7.37138500	0.47075800	0.20927600
C	-6.57623700	1.43335500	-0.41746800
C	-5.20047600	1.25279000	-0.56431200
C	-8.85486400	0.67522600	0.39078400
C	-2.32407500	0.99151200	-0.88737600
C	-1.94836100	0.92513200	-2.25667600
C	-1.33907400	2.03022900	-2.87976800
C	-1.12071200	3.22163700	-2.20661100
C	-1.54284000	3.30597200	-0.86063100
C	-2.13786300	2.23520100	-0.22870400
C	-0.50219900	4.39935900	-2.90990400
H	1.89361600	-3.98798800	1.42003000
H	1.78559300	-4.14979800	3.89522000
H	0.42899600	-2.49465700	5.16934400
H	-0.78596900	-0.71074400	3.98673600
H	-2.31287400	1.13300800	3.26389800
H	-1.61332500	3.04325700	4.61209900
H	0.60792200	4.11560900	4.21420000
H	2.03084800	3.28552700	2.36582800
H	3.43578700	-2.43680600	0.73154500
H	5.69732500	-2.06418600	-0.16531600
H	6.00031800	-1.12110900	-2.44440300

H	4.00820500	-0.54638000	-3.81768500
H	1.72967800	-0.89777500	-2.89976800
H	2.07606900	-3.85980700	-2.40804500
H	1.47365800	-6.10599000	-3.23945000
H	-0.58588000	-7.22933400	-2.42170900
H	-2.04095800	-6.08476100	-0.76245900
H	-1.43792800	-3.84125900	0.07352600
H	4.54625600	1.91500000	-0.25243000
H	6.60400700	1.14626500	0.84611800
H	6.48993000	-0.34185400	2.83637500
H	4.26115700	-1.01693100	3.73107600
H	2.20535800	-0.22933900	2.65178100
H	1.17954100	4.32910900	0.63407800
H	2.00611500	6.24973300	-0.65993900
H	3.36611800	5.89369200	-2.71011700
H	3.83071100	3.57307400	-3.48407700
H	2.99388200	1.64475900	-2.18615200
H	-2.57274500	-1.58963900	1.30947200
H	-3.03785700	-0.05864500	1.98694100
H	-4.91451600	-1.79256800	0.88759700
H	-7.33937100	-1.46405100	1.15735200
H	-7.03820600	2.33884800	-0.80237100
H	-4.61374100	2.01726700	-1.06077000
H	-9.23118600	1.46659500	-0.26329000
H	-9.09415900	0.95942500	1.42295000
H	-9.41418400	-0.23997200	0.17136800
H	-2.27761000	0.11348700	-2.88757700
H	-1.08368100	1.95587600	-3.93330900
H	-1.41948900	4.24097200	-0.32288300
H	-2.49028600	2.34821000	0.78694100
H	0.56979900	4.24449300	-3.05767000
H	-0.62403000	5.31963800	-2.33416500
H	-0.95496800	4.54822500	-3.89573500

TS8

Energy: -4237.53244 Hartree

Ni	-1.33518700	1.08597500	-0.29803700
P	3.11028100	-0.85602300	-0.67444800
P	-3.11102300	-0.04813800	-0.61435900
C	2.12222300	-1.82121600	0.54095300
C	2.80342400	-2.52447900	1.55657800
C	2.16452700	-3.42964600	2.38889300
C	0.79359600	-3.66704300	2.18770500
C	0.10066000	-2.97746800	1.21500600
C	0.71063800	-1.99755500	0.37348000
C	-0.14545500	-1.23857100	-0.51362200
C	-1.17413600	-1.98217600	-1.26515900
C	-0.85577900	-3.25643800	-1.79827600
C	-1.76523000	-4.01494600	-2.52167300
C	-3.05429300	-3.53274100	-2.77090700
C	-3.41780900	-2.29706100	-2.24489800
C	-2.51718300	-1.53936000	-1.48573500
C	4.55534300	-1.99274200	-0.89519500
C	5.84657900	-1.75047100	-0.41018400
C	6.87774000	-2.66159500	-0.64982500
C	6.63373600	-3.82870300	-1.37143700
C	5.34891200	-4.08042600	-1.85934300
C	4.32371900	-3.16770300	-1.62904400
C	3.87716300	0.50190500	0.31405700
C	3.68172300	0.68094300	1.68881500

C	4.28142300	1.74892400	2.35937900
C	5.09273700	2.64736400	1.66824200
C	5.28977100	2.48270500	0.29528200
C	4.67771900	1.42787100	-0.37536100
C	-4.49363200	0.58842900	-1.62037900
C	-4.18782800	1.48224800	-2.65673500
C	-5.19418000	1.96853900	-3.48725100
C	-6.51920800	1.57670600	-3.28288300
C	-6.83190200	0.69376900	-2.24923300
C	-5.82412900	0.19702900	-1.42206800
C	-3.87839700	-0.66227200	0.92306200
C	-3.72239100	-1.98723000	1.34445400
C	-4.26032000	-2.40084700	2.56328000
C	-4.97177900	-1.50228800	3.35857200
C	-5.13586900	-0.18002900	2.93787500
C	-4.57808100	0.24300600	1.73440500
C	0.28045900	0.10523300	-1.00914100
H	3.87413100	-2.37621300	1.65987500
H	2.71630000	-3.95860300	3.15924800
H	0.26644200	-4.38185200	2.81457800
H	-0.84650400	0.59458000	-1.64641800
H	-0.96678500	-3.13460000	1.11060000
H	0.14936600	-3.63212700	-1.63908600
H	-1.45952400	-4.97934900	-2.91839900
H	-3.76387400	-4.11264000	-3.35210100
H	-4.42507100	-1.92425900	-2.40249700
H	6.04847700	-0.85117300	0.16087900
H	7.87281200	-2.45881400	-0.26372600
H	5.14836500	-4.98694900	-2.42313700
H	3.32653600	-3.36853000	-2.01230200
H	3.06360000	-0.01986600	2.23708700
H	4.11745400	1.87277800	3.42617100
H	5.90824600	3.18589800	-0.25469600
H	4.81455200	1.32023900	-1.44764200
H	-3.15784200	1.79799000	-2.79992900
H	-4.94741100	2.65887200	-4.28794700
H	-7.86106000	0.38821800	-2.08672100
H	-6.06989600	-0.48994800	-0.61936700
H	-3.18864000	-2.68880900	0.71423600
H	-4.13097000	-3.42938500	2.88572200
H	-5.68892400	0.52293800	3.55315700
H	-4.68961200	1.27502000	1.41620900
H	0.84054200	0.15322300	-1.94539500
H	7.43621000	-4.53701900	-1.55445600
H	-7.30514300	1.96244800	-3.92487100
H	5.56142700	3.47553900	2.19151000
H	-5.39926400	-1.82957200	4.30145800
C	0.57127300	1.24081700	-0.10294800
C	1.31980800	2.42200900	-0.57144000
C	1.54661500	2.68312600	-1.93427000
C	1.85304600	3.32511500	0.36248500
C	2.28631300	3.78907300	-2.33605400
H	1.73166600	3.12466000	1.41962300
C	2.58188300	4.43467100	-0.04663700
H	1.14216200	2.02011300	-2.69065600
C	2.82338900	4.68370400	-1.40155100
H	2.99813700	5.10232700	0.70242500
H	2.44924200	3.96353800	-3.39622800
C	-0.06067800	1.27141300	1.23775300
C	-0.00237500	0.28909900	2.25806600
C	-1.03676400	2.32289200	1.37954200

C	-0.83198500	0.36725500	3.35536800
H	0.71579700	-0.51141800	2.18298400
C	-1.85217900	2.37815500	2.53368400
H	-0.94967000	3.21924400	0.77073900
C	-1.78251900	1.40512300	3.51272500
H	-0.75470300	-0.39717900	4.12358400
H	-2.53466100	3.21507200	2.65284300
C	3.67457200	5.84856300	-1.83663800
H	3.43870300	6.15935700	-2.85834300
H	3.54065700	6.71134400	-1.17725000
H	4.73963300	5.58502600	-1.81288100
C	-2.64881600	1.44697200	4.74311100
H	-3.22777300	0.52321200	4.83924900
H	-2.04192500	1.55000100	5.64999700
H	-3.35126500	2.28387900	4.70970100

TS9
Energy: -4237.572681 Hartree

Ni	0.19411800	-0.49063500	-0.54984600
P	2.12932600	0.01121400	0.03903100
P	-1.17914100	1.09196700	-0.32279300
C	2.18438000	-0.30040500	1.84054500
C	3.40157100	-0.13385300	2.52935900
C	3.53655400	-0.43252200	3.87709800
C	2.43473700	-0.94812600	4.55773000
C	1.23937200	-1.14517600	3.88053600
C	1.05974600	-0.81623100	2.51919400
C	-0.41372300	-0.99324600	2.05773900
C	-1.19958500	0.24754200	2.42499600
C	-1.61048300	0.40003500	3.75713500
C	-2.26751800	1.54294800	4.19881600
C	-2.51753600	2.57957200	3.30122000
C	-2.13321600	2.43737900	1.97188800
C	-1.49631600	1.27613300	1.50045400
C	2.72167400	1.73394400	-0.24125400
C	3.33637200	2.56115700	0.70563900
C	3.79263000	3.83097200	0.34757200
C	3.64733900	4.28980400	-0.96060500
C	3.00525400	3.48747700	-1.90462100
C	2.53177100	2.23068800	-1.54214700
C	3.61452500	-0.93313400	-0.54212500
C	4.22251300	-0.60041700	-1.76218500
C	5.29314400	-1.34225600	-2.25606300
C	5.77561800	-2.44336100	-1.54541200
C	5.17070800	-2.79323300	-0.33974000
C	4.09894600	-2.04910700	0.15420500
C	-0.70058700	2.81624500	-0.77242500
C	0.06869700	3.60528400	0.09453700
C	0.45759000	4.89063000	-0.27175400
C	0.11008800	5.40133600	-1.52266000
C	-0.62854100	4.61322700	-2.40503100
C	-1.03529000	3.33281600	-2.03121800
C	-2.87515100	1.03713100	-1.08021800
C	-4.03433300	1.61837200	-0.55108300
C	-5.25449900	1.50430500	-1.21351500
C	-5.33901200	0.81142500	-2.42248600
C	-4.19089700	0.23773500	-2.96666400
C	-2.97124700	0.35144500	-2.29900100
C	-0.88030200	-2.38804100	2.41693200
C	-1.02314100	-2.01919500	0.96315100

C	-0.10133500	-2.46100000	-0.13861100
C	-0.45657200	-2.12628800	-1.49788000
C	0.42237800	-2.54364300	-2.53170100
C	1.54391200	-3.32039000	-2.29105700
C	1.78021900	-3.76243400	-0.96797900
C	0.98924000	-3.34126600	0.07896000
C	2.48736200	-3.71508200	-3.39720100
C	-2.49888700	-2.03230000	0.57820000
C	-3.50604100	-1.21210800	1.10045300
C	-4.83850600	-1.36901000	0.72494000
C	-5.23694500	-2.35698600	-0.17475900
C	-4.25077200	-3.23782300	-0.62914200
C	-2.92128900	-3.09027200	-0.24900600
C	-6.66087300	-2.44675100	-0.65658700
H	4.26914500	0.20009400	1.97144500
H	4.48825800	-0.29154800	4.37982100
H	2.50726200	-1.21285300	5.60826800
H	0.40830300	-1.57402200	4.42707600
H	-1.41224600	-0.40116800	4.45975000
H	-2.57247700	1.62613800	5.23748800
H	-3.01316300	3.48806900	3.62916500
H	-2.32176300	3.24978200	1.27998900
H	3.45337300	2.22588300	1.72876200
H	4.26632500	4.46047800	1.09551100
H	4.00676200	5.27642800	-1.23661500
H	2.84341200	3.85386000	-2.91316600
H	1.98048300	1.63231700	-2.26177900
H	3.87108200	0.25576900	-2.32550900
H	5.75503400	-1.05553300	-3.19637300
H	6.61198900	-3.02011800	-1.92832600
H	5.53090200	-3.65014900	0.22248900
H	3.63952800	-2.34044100	1.09010700
H	0.36209000	3.21390100	1.06127200
H	1.05078700	5.48535200	0.41492700
H	0.41831600	6.40224900	-1.80940800
H	-0.90221100	4.99941800	-3.38274800
H	-1.63048000	2.73796200	-2.71601100
H	-3.99730100	2.13918400	0.39746700
H	-6.14441200	1.95304900	-0.78140400
H	-6.29197200	0.72135800	-2.93540000
H	-4.24309300	-0.30335900	-3.90672400
H	-2.07831400	-0.09934700	-2.72009000
H	-0.09972400	-3.07881000	2.71753600
H	-1.81982600	-2.52328800	2.94395400
H	-1.48035100	-1.90511600	-1.76611400
H	0.17560900	-2.27539000	-3.55654300
H	2.62037000	-4.42167400	-0.77506400
H	1.21045100	-3.67531700	1.08777700
H	2.08061900	-3.45753200	-4.37930900
H	2.69063900	-4.79206000	-3.38582500
H	3.44882800	-3.20256900	-3.28519100
H	-3.27506800	-0.42399700	1.79649500
H	-5.57753300	-0.68251400	1.12786800
H	-4.52388700	-4.05465700	-1.29238700
H	-2.19441300	-3.80857100	-0.60674600
H	-6.82889500	-1.74476000	-1.48289400
H	-7.37115500	-2.19014300	0.13561500
H	-6.90182400	-3.44995500	-1.02005500

TS10
Energy: -4370.366263 Hartree

Ni	-0.15050200	0.29183500	-0.42586500
P	-1.94577200	-0.70882300	-0.09015600
P	1.48712200	-1.01853700	-0.28005200
C	-2.36057400	-0.46647300	1.67718400
C	-3.62156900	-0.81035500	2.18778700
C	-3.95335600	-0.58560600	3.51838000
C	-3.00986900	0.00941100	4.35480200
C	-1.76883000	0.37782200	3.84751300
C	-1.40342800	0.14779200	2.50666900
C	0.01837600	0.59093500	2.10683600
C	1.09208700	-0.45429300	2.43578500
C	1.35635900	-0.65252600	3.80384400
C	2.28874300	-1.57902800	4.25364800
C	3.00370000	-2.34413800	3.33188600
C	2.76535000	-2.15906000	1.97663600
C	1.83046300	-1.21745400	1.51122700
C	-2.10894200	-2.52480100	-0.33021800
C	-2.44852700	-3.44013600	0.67130400
C	-2.62720500	-4.79134900	0.36496700
C	-2.46477000	-5.24267200	-0.94274000
C	-2.09819000	-4.34008400	-1.94442600
C	-1.91759300	-2.99649000	-1.63904600
C	-3.52059200	-0.19378400	-0.93152500
C	-4.58859500	-1.06127900	-1.20661500
C	-5.74377800	-0.59309600	-1.83393700
C	-5.85324700	0.74947200	-2.19883500
C	-4.79555500	1.62073600	-1.93460800
C	-3.63956400	1.14923700	-1.31427800
C	1.36017400	-2.78969400	-0.80264700
C	0.86140500	-3.76264700	0.07595200
C	0.75986500	-5.09435400	-0.31614100
C	1.13135200	-5.48080500	-1.60366500
C	1.59756100	-4.51781500	-2.49722000
C	1.71346400	-3.18645200	-2.09981000
C	3.10534400	-0.61246100	-1.07933400
C	4.37495700	-0.75771100	-0.51231300
C	5.52173700	-0.48828900	-1.26017700
C	5.41896800	-0.07210900	-2.58684100
C	4.15623100	0.10161500	-3.15660000
C	3.01276300	-0.15341900	-2.40357200
C	0.36044000	1.92124400	2.72551100
C	0.57287500	2.06087500	1.24779000
C	-0.39334500	3.06086100	0.65480400
C	0.05534100	4.22888000	0.01933300
C	-0.83813400	5.18781900	-0.46059500
C	-2.21860400	5.04588500	-0.30600700
C	-2.66561100	3.91572400	0.38854600
C	-1.77966900	2.95382400	0.85995100
C	-3.18966700	6.05677100	-0.86220800
C	2.01611200	2.11564700	0.80513000
C	2.37486900	2.46422500	-0.51076000
C	3.69385200	2.69422200	-0.87322800
C	4.74267400	2.52768800	0.03792500
C	4.40583800	2.10299500	1.32260800
C	3.07794400	1.91242800	1.70127400
C	6.17049700	2.79350400	-0.35993600
H	-4.35612700	-1.24232100	1.51652900
H	-4.93685100	-0.85277400	3.89274000
H	-3.24427900	0.20785000	5.39635200
H	-1.07284800	0.87762000	4.51056500

H	0.81322500	-0.06820700	4.53660400
H	2.45199200	-1.70532400	5.31977600
H	3.73098200	-3.07790500	3.66570400
H	3.28844200	-2.77554500	1.25436000
H	-2.58370700	-3.10119600	1.69188600
H	-2.89689100	-5.48923800	1.15269900
H	-2.60365200	-6.29313700	-1.17981600
H	-1.93928600	-4.68888200	-2.95984800
H	-1.62473500	-2.29654700	-2.41612500
H	-4.51528200	-2.10928100	-0.93783700
H	-6.56036100	-1.27999500	-2.03775300
H	-6.75327500	1.11107500	-2.68746900
H	-4.86797800	2.66969300	-2.21042600
H	-2.81974800	1.82418400	-1.11854800
H	0.55321500	-3.47917900	1.07465500
H	0.37248800	-5.82815400	0.38306600
H	1.04961700	-6.51967200	-1.90905900
H	1.88626400	-4.80278600	-3.50505700
H	2.10444000	-2.45824000	-2.80071300
H	4.47668200	-1.06199200	0.52145000
H	6.49966000	-0.60085800	-0.80098200
H	6.31443500	0.12751200	-3.16809800
H	4.06327000	0.44688700	-4.18243800
H	2.02832000	0.02402300	-2.82843000
H	-0.47690400	2.49923800	3.10073500
H	1.22192800	1.98898100	3.38008800
H	1.11458800	4.40938500	-0.09716500
H	-0.44242000	6.07390800	-0.95173600
H	-3.73047800	3.76918500	0.54966800
H	-2.18587200	2.09593400	1.37609600
H	-2.68504200	6.98895800	-1.13165800
H	-3.97723600	6.29537000	-0.14004800
H	-3.68840000	5.68014000	-1.76456000
H	1.60688100	2.56050100	-1.26229100
H	3.91736400	2.96979200	-1.89946500
H	5.19171100	1.92274600	2.05149300
H	2.89490700	1.58208900	2.71401100
H	6.36446500	2.44795100	-1.37900300
H	6.87127500	2.28563500	0.30882800
H	6.39985500	3.86593900	-0.32437500
N	-0.36476800	1.45188900	-1.82355500
C	-0.71958100	2.18973100	-2.65525000
C	-1.31000300	3.13033300	-3.59387700
H	-2.31927800	2.80103100	-3.86421800
H	-1.37861700	4.11353200	-3.11671600
H	-0.71282500	3.22007600	-4.50654200

TS11

Energy: -4237.58229 Hartree

Ni	-0.02180600	-0.45327900	0.29853500
P	1.27669400	1.16515200	-0.53115900
P	-2.21581000	-0.17714200	0.38442200
C	2.17704400	1.52875800	1.00561300
C	3.46773700	2.03257400	1.11020800
C	4.03142900	2.23988800	2.37234700
C	3.27617800	1.95359800	3.51291300
C	1.97828700	1.45281600	3.41192700
C	1.39090900	1.21752400	2.14859600
C	0.08993900	0.61110400	1.91675000
C	-1.11948300	1.05996400	2.52132900

C	-1.20207700	1.93684600	3.64821600
C	-2.41677200	2.26401100	4.21896200
C	-3.63334000	1.76560900	3.71149300
C	-3.59723700	0.97389400	2.57240000
C	-2.37573600	0.65189000	1.96511500
C	2.59558300	1.04803400	-1.80014600
C	2.73096400	1.92494400	-2.88441800
C	3.75841600	1.74431300	-3.81201800
C	4.66629500	0.69545700	-3.66653000
C	4.54838400	-0.17198800	-2.57859100
C	3.52257800	0.00207100	-1.65410700
C	0.35073800	2.69607800	-0.91136300
C	0.20392900	3.70540300	0.04787600
C	-0.58581400	4.82274000	-0.22462400
C	-1.22091400	4.95114000	-1.45874800
C	-1.08337300	3.94531800	-2.41687600
C	-0.32062200	2.81536400	-2.13735500
C	-3.45362800	-1.53558200	0.46831400
C	-3.50465200	-2.29655800	1.64667100
C	-4.34847300	-3.39921000	1.74624600
C	-5.16536600	-3.75748200	0.67080700
C	-5.13748700	-2.99450700	-0.49595600
C	-4.28716500	-1.89217800	-0.59747100
C	-2.90352600	0.88862300	-0.93937100
C	-2.71271000	0.49016300	-2.27200700
C	-3.28309800	1.21750700	-3.31462100
C	-4.02362700	2.36855500	-3.03809900
C	-4.17927900	2.79191000	-1.71823700
C	-3.62716300	2.05437700	-0.67086500
C	0.49077700	-1.55456000	2.19169500
H	4.03827000	2.24655100	0.21195300
H	5.04349600	2.62050500	2.46455600
H	3.71158500	2.10065000	4.49758300
H	-0.52721700	-1.85307600	2.41589200
H	1.43612000	1.18724500	4.31256300
H	-0.29434300	2.35706600	4.06249200
H	-2.42941500	2.92381800	5.08292400
H	-4.57614000	2.01551400	4.18623600
H	-4.51982100	0.60583900	2.13102800
H	2.04554800	2.75579800	-3.00121300
H	3.85185400	2.43299500	-4.64663900
H	5.25122800	-0.98780500	-2.44093000
H	3.45445800	-0.66415500	-0.80486000
H	0.70483900	3.61481600	1.00446200
H	-0.69905200	5.59525700	0.52994600
H	-1.59321800	4.02600900	-3.37047700
H	-0.25307400	2.01919800	-2.87109800
H	-2.88816200	-2.00818300	2.49188700
H	-4.37487200	-3.97591800	2.66603300
H	-5.78245000	-3.25284300	-1.33085000
H	-4.27990200	-1.30718800	-1.50943700
H	-2.12037600	-0.39529000	-2.48428300
H	-3.14270200	0.89202000	-4.34134100
H	-4.73391000	3.69964700	-1.50087700
H	-3.75230900	2.38325500	0.35460200
H	1.08458400	-1.31103500	3.06449100
H	5.46321800	0.55933700	-4.39138900
H	-5.82602500	-4.61549600	0.74831700
H	-1.82995400	5.82463700	-1.67096700
H	-4.46481000	2.94006900	-3.84922600
C	1.15161100	-2.10017500	1.06523400

C	0.28630100	-2.56590700	-0.04351300
C	0.50634600	-2.09255900	-1.36561300
C	-0.80450900	-3.44973100	0.16240900
C	-0.37377300	-2.45657900	-2.40310700
H	1.42039900	-1.57040700	-1.61293300
C	-1.63957200	-3.80787800	-0.87212500
H	-0.97558200	-3.84303000	1.15789300
C	-1.45514300	-3.29645500	-2.17724800
H	-0.18098100	-2.08567400	-3.40635700
H	-2.47436000	-4.47362100	-0.67691200
C	2.60983800	-2.18785000	0.94369100
C	3.19840900	-3.07028400	0.01404600
C	3.48783700	-1.42405800	1.74109300
C	4.57876200	-3.13421200	-0.15192800
H	2.56289600	-3.71023500	-0.58771300
C	4.86333700	-1.49556300	1.56829000
H	3.09267000	-0.72850300	2.46875400
C	5.44229300	-2.33610700	0.60694600
H	4.99320600	-3.82064800	-0.88634400
H	5.49978400	-0.86168800	2.18004600
C	-2.41486500	-3.66728600	-3.27635600
H	-2.49575900	-4.75437700	-3.38519000
H	-3.42011800	-3.29311000	-3.05246500
H	-2.10323100	-3.25229300	-4.23835300
C	6.93212600	-2.35027300	0.37904900
H	7.48373100	-2.26817500	1.32102300
H	7.25157600	-3.26909800	-0.12142000
H	7.24577100	-1.50767600	-0.25098700

TS12
Energy: -4350.975859 Hartree

Ni	0.00890800	-0.02947700	0.00283300
P	1.95437000	0.94578300	-0.18842200
P	-2.01497100	0.81978100	0.01820800
C	2.54273000	0.98687300	1.52358900
C	3.86850700	1.05505700	1.93864400
C	4.17164500	0.96463600	3.29863100
C	3.13232300	0.77739200	4.21327000
C	1.80481900	0.70100700	3.79525600
C	1.46220400	0.82261500	2.42824200
C	0.12263500	0.65237300	1.86715800
C	-1.05109800	1.22460500	2.47322200
C	-1.12647500	1.85461400	3.74958000
C	-2.33973100	2.24325100	4.29381100
C	-3.55322100	2.05486100	3.60971400
C	-3.51014200	1.53355500	2.32202900
C	-2.28697300	1.15988200	1.75430100
C	1.73439900	2.67766400	-0.73121400
C	1.13889200	3.58320000	0.15956100
C	0.86846500	4.88625100	-0.24741200
C	1.19033700	5.30006300	-1.54105400
C	1.77054100	4.40015200	-2.43373200
C	2.03195800	3.08916200	-2.03661300
C	3.32299200	0.29563700	-1.20315200
C	4.46818300	1.07144000	-1.45481700
C	5.53131100	0.54200400	-2.18198500
C	5.46068100	-0.76431500	-2.67276100
C	4.32674300	-1.53707700	-2.42979000
C	3.26309800	-1.01253500	-1.69603600
C	-2.08749700	2.44467900	-0.84073000

C	-1.52250100	2.58316900	-2.11623100
C	-1.61520900	3.79190800	-2.80171800
C	-2.25280700	4.88360200	-2.21271900
C	-2.79281600	4.76135500	-0.93306300
C	-2.71298800	3.54914100	-0.24889400
C	-3.46483500	-0.08423000	-0.62317200
C	-4.12793100	-0.99330200	0.21074500
C	-5.21999900	-1.71364700	-0.26618900
C	-5.65073700	-1.54879600	-1.58231700
C	-4.97980500	-0.66104000	-2.42451000
C	-3.89562100	0.07258600	-1.94801400
C	-0.18997100	-1.46830800	2.02256500
C	-0.01009400	-2.30624200	0.88653400
C	1.32660000	-2.85824600	0.59216200
C	1.53586300	-3.91417400	-0.31948600
C	2.81243600	-4.41053300	-0.58204400
C	3.94775500	-3.89921500	0.05263000
C	3.74524800	-2.87145700	0.98390200
C	2.48179100	-2.36482000	1.24431800
C	5.33376000	-4.38930600	-0.27663500
C	-1.22170800	-3.00204800	0.35675700
C	-1.47757300	-3.20982900	-1.01049300
C	-2.58857800	-3.92094700	-1.44819600
C	-3.50220800	-4.47285000	-0.54252000
C	-3.25088100	-4.28753500	0.81963100
C	-2.13834100	-3.57331700	1.25802900
C	-4.72118000	-5.21276800	-1.02842900
H	4.66344300	1.15119500	1.20659800
H	5.20164800	1.01424500	3.63592800
H	3.36023100	0.66152300	5.26927000
H	1.03456100	0.48756100	4.52593000
H	-0.22063000	2.04167700	4.31037300
H	-2.34770700	2.70461600	5.27793700
H	-4.49637000	2.34264900	4.06149200
H	-4.42080100	1.43383400	1.73795700
H	0.87680200	3.25821600	1.16038900
H	0.39774800	5.57719500	0.44433100
H	0.97714400	6.31709800	-1.85485400
H	2.01491500	4.71422700	-3.44409000
H	2.47595500	2.39183800	-2.73848900
H	4.52051600	2.09239600	-1.09115100
H	6.41198200	1.14885600	-2.36873100
H	6.28808400	-1.17525400	-3.24334000
H	4.26456900	-2.55595800	-2.79622300
H	2.40434200	-1.63804000	-1.48864900
H	-1.00324900	1.75042900	-2.57574600
H	-1.16956900	3.88532300	-3.78658300
H	-2.31523600	5.82868000	-2.74351100
H	-3.27796600	5.61149000	-0.46282800
H	-3.12766400	3.46249200	0.74845000
H	-3.78743900	-1.13669000	1.22877500
H	-5.72592100	-2.41361800	0.39009500
H	-6.50458600	-2.10935900	-1.95065100
H	-5.30805500	-0.52925500	-3.45103900
H	-3.39616700	0.77848300	-2.60235200
H	0.54418700	-1.49386300	2.82238300
H	-1.21094800	-1.33538000	2.36255100
H	0.68904600	-4.36662100	-0.82002300
H	2.92279000	-5.22302100	-1.29689500
H	4.59813700	-2.43933300	1.50177300
H	2.39649700	-1.55330100	1.95261100

H	5.88073800	-3.65169100	-0.87815900
H	5.30320500	-5.32298400	-0.84596500
H	5.92503700	-4.56583100	0.62843400
H	-0.79287100	-2.81019700	-1.74624600
H	-2.75479600	-4.04146600	-2.51564000
H	-3.93013800	-4.71856200	1.55129500
H	-1.95749700	-3.47863300	2.32412300
H	-5.22990700	-5.72775500	-0.20866200
H	-4.46381900	-5.95873600	-1.78783100
H	-5.43972200	-4.52203800	-1.48618500
C	-0.03052700	-0.50768000	-1.67635500
O	-0.00721700	-0.67695400	-2.81837100

CO

Energy: -113.3769844 Hartree

O	0.00000000	0.00000000	0.48768700
C	0.00000000	0.00000000	-0.65024900

MeCN

Energy: -132.7921677 Hartree

N	0.00000000	0.00000000	1.43964000
C	0.00000000	0.00000000	0.27952800
C	0.00000000	0.00000000	-1.18016800
H	0.00000000	1.02592700	-1.55788000
H	0.88847900	-0.51296300	-1.55788000
H	-0.88847900	-0.51296300	-1.55788000

N_2C(p-tol$_2$)

Energy: -689.6049849 Hartree

C	-0.00041600	1.06819400	-0.00093500
C	-1.31449700	0.40024900	-0.00796000
C	-1.47186900	-0.86582100	-0.59570500
C	-2.44884700	1.01617400	0.54649200
C	-2.71508700	-1.49036400	-0.61153900
H	-0.61698100	-1.35703700	-1.04625100
C	-3.68904300	0.38859000	0.51126500
H	-2.35624900	1.99024700	1.01764000
C	-3.84867200	-0.87778900	-0.06462300
H	-2.80803500	-2.47168000	-1.06944800
H	-4.54871000	0.88688200	0.95157800
C	1.31500900	0.40290000	0.00891300
C	2.44874600	1.01930900	-0.54562000
C	1.47580300	-0.85730100	0.60886000
C	3.69143600	0.39679100	-0.50149700
H	2.35582200	1.99355900	-1.01634200
C	2.72111600	-1.47679000	0.63346900
H	0.62325000	-1.34360200	1.06911700
C	3.85297200	-0.86677500	0.07952600
H	4.55201000	0.89852100	-0.93601300
H	2.81829000	-2.45043100	1.10672300
C	-5.20018100	-1.54427000	-0.11921900
H	-5.81655600	-1.26871300	0.74160500
H	-5.75152500	-1.25008600	-1.02100900
H	-5.10797700	-2.63402200	-0.13570300
C	5.19327600	-1.55740600	0.08545600
H	5.32864000	-2.16997300	-0.81467900

H	6.01461800	-0.83546500	0.11312200
H	5.29591500	-2.22182400	0.94842500
N	-0.00203100	2.37608800	-0.00377800
N	-0.00382500	3.52383700	-0.00623200

N_2

Energy: -109.5862792 Hartree

N	0.00000000	0.00000000	0.55274900
N	0.00000000	0.00000000	-0.55274900

1,1-di(p-tolyl)ethylene

Energy: -619.4035723 Hartree

C	-0.00060900	1.43158000	-0.00346800
C	-1.27473900	0.66673600	-0.01383900
C	-1.40296400	-0.51453700	-0.76152600
C	-2.39269600	1.11013200	0.70790700
C	-2.60841300	-1.20818500	-0.80144900
H	-0.54913200	-0.88494600	-1.31906800
C	-3.59592900	0.41129800	0.66660800
H	-2.30433300	1.99778800	1.32607900
C	-3.72868200	-0.75735500	-0.09224400
H	-2.68241200	-2.11685800	-1.39335200
H	-4.44344500	0.77118400	1.24420800
C	1.27500200	0.66940000	0.01218200
C	2.39370400	1.11210400	-0.70888500
C	1.40632100	-0.50383900	0.77187200
C	3.59987700	0.41922900	-0.65730900
H	2.30603500	1.99908700	-1.32816000
C	2.61481400	-1.19152900	0.82209300
H	0.55482800	-0.86793900	1.33714100
C	3.73359300	-0.74532600	0.10771000
H	4.45012800	0.78219800	-1.22899200
H	2.69341800	-2.08989600	1.42894300
C	-5.04034900	-1.49868800	-0.15855600
H	-5.63802100	-1.33497600	0.74270200
H	-5.64062700	-1.16341800	-1.01347400
H	-4.88601100	-2.57562700	-0.27370300
C	5.02967200	-1.51607100	0.13275600
H	5.04506700	-2.29048800	-0.64421700
H	5.88759000	-0.86102000	-0.04401200
H	5.17691100	-2.01814800	1.09350200
C	-0.00248400	2.77660900	-0.00834700
H	0.91958700	3.34349500	0.05899200
H	-0.92628900	3.34021900	-0.07960800

p-fluorostyrene

Energy: -408.982507 Hartree

C	0.98028700	-1.22138800	-0.00000900
C	-0.39898000	-1.05234800	0.00002100
C	-0.97746000	0.22986100	0.00002700
C	-0.11674900	1.33968500	0.00001100
C	1.26806800	1.19051400	-0.00002100
C	1.79544000	-0.09333600	-0.00003200
H	1.43252300	-2.20674200	-0.00001400
H	-1.03432200	-1.93143700	0.00004400
H	-0.54249100	2.33899200	0.00001900
H	1.93449600	2.04550200	-0.00003400
F	3.13499600	-0.25506200	-0.00005200

C	-2.42961000	0.45467300	0.00005500
H	-2.72331100	1.50352800	0.00012500
C	-3.39603200	-0.47024200	0.00000700
H	-3.19001300	-1.53620400	-0.00006400
H	-4.44162500	-0.18259900	0.00004400

6. Literature references

[1] C. Wu, F. Hu, Z. Liu, G. Deng, F. Ye, Y. Zhang, J. Wang, *Tetrahedron* **2015**, *71*, 9196–9201.
[2] J. B. Miller, *J. Org. Chem.* **1959**, *24*, 560–561.
[3] B. W. H. Saes, D. G. A. Verhoeven, M. Lutz, R. J. M. Klein Gebbink, M. E. Moret, *Organometallics* **2015**, *34*, 2710–2713.
[4] W. Chen, E. B. Twum, L. Li, B. D. Wright, P. L. Rinaldi, Y. Pang, *J. Org. Chem.* **2012**, *77*, 285–290.
[5] J. Wienand, "Error Propagation Calculator", can be found under https://www.julianibus.de, **2021**.
[6] B. J. Barrett, V. M. Iluc, *Organometallics* **2017**, *36*, 730–741.
[7] A. M. M. Schreurs, X. Xian, L. M. J. Kroon-Batenburg, *J. Appl. Crystallogr.* **2010**, *43*, 70–82.
[8] G. M. Sheldrick (2014). SADABS and TWINABS. Universität Göttingen, Germany.
[9] G. M. Sheldrick, *Acta Crystallogr. Sect. A Found. Crystallogr.* **2015**, *71*, 3–8.
[10] G. M. Sheldrick, *Acta Crystallogr. Sect. C Struct. Chem.* **2015**, *71*, 3–8.
[11] A. L. Spek, *Acta Crystallogr. Sect. D Biol. Crystallogr.* **2009**, *65*, 148–155.
[12] A. L. Spek, *Acta Crystallogr. Sect. C Struct. Chem.* **2015**, *71*, 9–18.
[13] R. Herbst-Irmer, G. M. Sheldrick, *Acta Crystallogr. Sect. B Struct. Sci.* **1998**, *54*, 443–449.
[14] M. J. Frisch, G. W. Trucks, H. B. Schlegel, G. E. Scuseria, M. a. Robb, J. R. Cheeseman, G. Scalmani, V. Barone, G. a. Petersson, H. Nakatsuji, et al., **2016**, Gaussian 16, Revision C.01, Gaussian, Inc., Wallin.
[15] D. J. Mindiola, G. L. Hillhouse, *J. Am. Chem. Soc.* **2002**, *124*, 9976–9977.
[16] V. M. Iluc, G. L. Hillhouse, *J. Am. Chem. Soc.* **2014**, *136*, 6479–6488.
[17] N. D. Harrold, A. R. Corcos, G. L. Hillhouse, *J. Organomet. Chem.* **2016**, *813*, 46–54.
[18] R. H. Grubbs, A. Miyashita, *J. Am. Chem. Soc.* **1978**, *100*, 7418–7420.
[19] A. Miyashita, R. H. Grubbs, *Tetrahedron Lett.* **1981**, *22*, 1255–1256.
[20] A. Miyashita, M. Ohyoshi, H. Shitara, H. Nohira, *J. Organomet. Chem.* **1988**, *338*, 103–111.

7. Author Contributions

Dr. M.-E. Moret initiated and supervised the project. M. L. G. Sansores-Paredes and S. van der Voort performed the synthesis and characterization of Ligand 1. M. L. G. Sansores-Paredes performed the remainder of the experiments and DFT calculations. Dr. M Lutz oversaw the acquisition, solving and interpretation of X-ray diffraction crystal structures. M. L. G. Sansores-Paredes and Dr. M.-E. Moret wrote the manuscript with the contributions of S. van der Voort and Dr. M. Lutz. All authors approved the final manuscript.
